# Invasive vs Conservative Strategy for Frail Older Patients With Myocardial Infarction: A Secondary Analysis of the SENIOR-RITA Randomized Clinical Trial

**DOI:** 10.1001/jamanetworkopen.2026.7316

**Published:** 2026-04-21

**Authors:** Francesca Rubino, Helen Mossop, David P. Ripley, Justin Carter, Darragh Twomey, Justin Cooke, David Austin, Murugapathy Veerasamy, Damian Kelly, Dirk Felmeden, Atul Anand, David E. Newby, Robert F. Storey, Keith A. A. Fox, Stuart J. Pocock, Vijay Kunadian

**Affiliations:** 1 Translational and Clinical Research Institute, Faculty of Medical Sciences, Newcastle University, Newcastle upon Tyne, United Kingdom; 2 Population Health Sciences Institute, Newcastle University, Newcastle upon Tyne, United Kingdom; 3 Cardiology Department, Northumbria Healthcare NHS Foundation Trust, Newcastle upon Tyne, United Kingdom; 4 North Tees and Hartlepool NHS Foundation Trust, Stockton-on-Tees, United Kingdom; 5 County Durham and Darlington NHS Trust, Darlington, United Kingdom; 6 Chesterfield Royal Hospital, Chesterfield, United Kingdom; 7 Academic Cardiovascular Unit, The James Cook University Hospital, South Tees NHS Trust, Middlesbrough, United Kingdom; 8 Population Health Sciences Institute, Newcastle University, Newcastle upon Tyne, United Kingdom; 9 Leeds Teaching Hospital NHS Trust, Leeds, United Kingdom; 10 Royal Derby Hospital, Derby, United Kingdom; 11 Torbay and South Devon NHS Foundation Trust, Torquay, United Kingdom; 12 Centre for Cardiovascular Science, University of Edinburgh, Edinburgh, United Kingdom; 13 NIHR Sheffield Biomedical Research Centre, Sheffield University, Sheffield, United Kingdom; 14 University of Edinburgh, Edinburgh, United Kingdom; 15 London School of Hygiene and Tropical Medicine, London, United Kingdom; 16 Cardiothoracic Centre, Freeman Hospital, Newcastle upon Tyne Hospitals NHS Foundation Trust, Newcastle upon Tyne, United Kingdom

## Abstract

**Importance:**

Frail older patients with non–ST-elevation myocardial infarction (NSTEMI) experience an increased risk of major adverse cardiovascular events. The beneficial role of an invasive strategy over a conservative strategy among frail patients with NSTEMI is unclear.

**Objective:**

To compare the clinical outcomes of an invasive strategy with those of a conservative strategy among older patients with NSTEMI stratified by frailty status.

**Design, Setting, and Participants:**

In this prespecified exploratory subgroup analysis from the SENIOR-RITA randomized clinical trial, patients were screened across 48 National Health Service trusts in England and Scotland from November 1, 2016, through March 31, 2023. The SENIOR-RITA trial included patients with NSTEMI aged 75 years or older, randomized to an invasive strategy with coronary angiography, revascularization if needed, and optimal medical therapy vs a conservative strategy with optimal medical therapy only. In this analysis, frailty status was defined using the Fried frailty criteria (frail, ≥3 criteria present). Statistical analysis was performed from March through November 2025.

**Interventions:**

Invasive vs conservative strategy.

**Main Outcomes and Measures:**

The primary composite outcome was the time to cardiovascular death or nonfatal myocardial infarction. All participants were analyzed according to the intention-to-treat principle.

**Results:**

Fried frailty criteria were available for 1446 of the 1518 randomized patients (95.3%), of whom 469 (32.4%; median age, 83 years [IQR, 80-86 years]; 240 women [51.2%]) met criteria for frailty. The primary outcome among frail patients occurred among 87 of 231 patients (37.7%) in the invasive group and 70 of 238 patients (29.4%) in the conservative group (hazard ratio [HR], 1.21; 95% CI, 0.88-1.67) over a median follow-up of 4.1 years (IQR, 2.8-4.6 years). When frailty was analyzed as a continuous variable, there was a significant interaction with treatment such that patients at the highest levels of frailty had a potential signal for harm with routine invasive strategy. There were no significant treatment differences across frailty categories for cardiovascular death (HR, 1.44; 95% CI, 0.97-2.10) or nonfatal myocardial infarction (HR, 1.00; 95% CI, 0.61-1.63).

**Conclusions and Relevance:**

In this subgroup analysis of a randomized clinical trial, an invasive strategy did not reduce the risk of a composite outcome of cardiovascular death or nonfatal myocardial infarction compared with a conservative strategy, with a potential signal for increased risk of harm among those at the highest levels of frailty. These findings underscore the need for individualized, frailty-informed treatment strategies.

**Trial Registration:**

isrctn.org Identifier: ISRCTN11343602

## Key Points

**Question:**

Among frail older adults with non–ST-elevation myocardial infarction (NSTEMI), does an invasive strategy reduce the risk of cardiovascular death or nonfatal myocardial infarction (MI) compared with a conservative strategy?

**Findings:**

In this subgroup analysis of 469 frail patients from the SENIOR-RITA randomized clinical trial, an invasive strategy did not reduce the risk of cardiovascular death or nonfatal MI. A significant interaction was found between treatment and frailty severity such that patients at the highest levels of frailty had a potential signal for harm with routine invasive strategy.

**Meaning:**

Among frail older adults with NSTEMI, an invasive strategy provided no benefit over conservative management and may be associated with worse outcomes at higher levels of frailty, highlighting the importance of incorporating frailty assessment into clinical decision-making.

## Teaser

This secondary analysis of the SENIOR-RITA randomized clinical trial compares the clinical outcomes of an invasive strategy with those of a conservative strategy among older patients with non–ST-elevation myocardial infarction stratified by frailty status.

## Introduction

Frailty is a status characterized by a diminished biological reserve, resulting in the failure of homeostatic mechanisms in response to stressors, such as acute coronary syndrome (ACS).^1^ As the global population continues to age, the issue of frailty becomes increasingly more significant.^2^ Frailty increases the risk of cardiovascular disease among older patients regardless of classical cardiovascular risk factors.^3^ Frail patients with non–ST-elevation ACS present with more complex coronary disease, have longer hospital stays, and less frequently receive invasive management and optimal medical therapy.^4,5^ In addition, frailty is considered a negative prognostic factor among older patients with ACS, associated with an increased risk of all-cause mortality, myocardial infarction, stroke, unplanned revascularization, and major bleeding.^6,7^ The European Society of Cardiology guidelines recommend a holistic and personalized approach to managing frail patients with ACS, emphasizing the importance of carefully balancing risks and benefits.^8^

Frail patients with ACS are underrepresented in randomized clinical trials.^9,10^ The MOSCA-FRAIL trial enrolled 167 older frail patients with non–ST-elevation myocardial infarction (NSTEMI), comparing routine invasive management vs conservative management, and showed no difference between the 2 groups in terms of number of days alive out of the hospital from discharge to 1 year.^9^ To our knowledge, the SENIOR-RITA trial is the first large, randomized clinical trial to study older patients with NSTEMI, including frail patients, testing an invasive vs conservative strategy in their management.^11^ This prespecified subgroup analysis of the SENIOR-RITA trial focuses on frail patients and seeks to provide valuable insights to support clinical practice and enhance the management of frail older patients with NSTEMI.

## Methods

The design and methods of the SENIOR-RITA trial have been published previously.^11^ In brief, the SENIOR-RITA trial was a prospective, multicenter, open-label, randomized clinical trial that recruited patients at least 75 years of age with NSTEMI to test an invasive vs conservative treatment strategy. All patients provided written informed consent (versions of trial protocol and statistical analysis plan in Supplement 1). A written consultee declaration was obtained from a family member or a caregiver for patients with cognitive impairment. The trial was funded by the British Heart Foundation and sponsored by Newcastle upon Tyne Hospitals NHS Foundations Trust. The Newcastle Clinical Trials Unit managed and coordinated the trial. The protocol was approved by the UK Health Research Authority. This analysis follows the Consolidated Standards of Reporting Trials (CONSORT) reporting guideline.

### Trial Population

Patients with a clinical diagnosis of type 1 NSTEMI aged 75 years or older were enrolled from November 1, 2016, through March 31, 2023, across 48 National Health Service trusts in England and Scotland. Exclusion criteria included diagnosis of ST-elevation myocardial infarction, unstable angina, cardiogenic shock, or life expectancy of less than 1 year. Detailed inclusion and exclusion criteria are listed in the eMethods in Supplement 2.

We used the Fried frailty criteria in this analysis (frail: ≥3 criteria present; intermediate or prefrail: 1 or 2 criteria present; robust [nonfrail]: 0 criteria present), assessed at the baseline.^12^ The Charlson Comorbidity Index^13^ was used to define the degree of coexisting conditions, and the Montreal Cognitive Assessment was used to assess cognitive impairment.^14^ A detailed description of these scores is available in the eMethods in Supplement 2.

### Randomization and Treatment

All patients were randomly assigned in a 1:1 ratio to an invasive treatment with revascularization if appropriate, plus optimal medical therapy (the invasive strategy group), or to conservative management with optimal medical therapy alone (conservative strategy group). Randomization was on a 1:1 basis using a variable-length block-stratified method with randomly selected block sizes of 2, 4, 6, and 8. Randomization was performed at each site using a secure web-based system.

Optimal medical therapy included aspirin (75 mg daily), a P2Y12 receptor (P2Y purinergic receptor 12) antagonist, a β-blocker (target heart rate, 60-70 beats/min), statin therapy, and an angiotensin-converting enzyme inhibitor or angiotensin II receptor blocker. Comorbidities such as hypertension, diabetes, and hypercholesterolemia were managed according to the clinical practice guidelines.^8^

Patients assigned to the invasive group underwent coronary angiography with coronary revascularization if appropriate and received optimal medical therapy. Coronary angiography was conducted following local guidelines. Coronary revascularization was carried out within 3 to 7 days, when appropriate, through either percutaneous coronary intervention or coronary artery bypass grafting, as determined by the attending cardiologist and the multidisciplinary team. In the conservative strategy group, coronary angiography was permitted in cases of clinical deterioration when deemed necessary by the treating physicians.

### Outcomes and Follow-Up

The primary composite outcome was time to cardiovascular death or nonfatal myocardial infarction, defined by the fourth universal definition of myocardial infarction.^15^ Secondary outcome measures were a composite of death from any cause or myocardial infarction, death from any cause, cardiovascular death, noncardiovascular death, recurrent myocardial infarction, subsequent coronary angiography, subsequent coronary revascularization, and hospitalization for heart failure, stroke, transient ischemic attack, and bleeding (as per the Bleeding Academic Research Consortium criteria).^16^ Safety outcomes included procedural and in-hospital complications occurring among patients assigned to the invasive strategy. The outcomes were analyzed based on the Fried frailty status. Site level follow-up was performed at 6 months, 1 year, and then yearly until 5 years either via telephone or in-person visit.

### Statistical Analysis

Statistical analysis was performed from March through November 2025. Baseline characteristics were compared using the χ^2^ test, *t* test, or Mann-Whitney test as appropriate. A 2-sided *P* < .05 was considered statistically significant. Cumulative incidence was assessed using the Kaplan-Meier method, and the treatment strategies were compared using the log-rank test. The treatment effect was calculated using the Cox proportional hazards regression model with associated 95% CIs within the subgroups. When the proportional hazard assumption was violated, the difference in the restricted mean event-free time was estimated.

A competing-risk analysis for the primary end point was performed, considering noncardiovascular death as a competing event. Treatment effects were evaluated using the Fine-Gray subdistribution hazards model. A sensitivity analysis was performed using the Fried frailty score as a continuous variable to assess whether the observed categorical trends persisted on a continuous scale. All participants were analyzed according to the intention-to-treat principle. Analyses were conducted using R, version 4.3.1 and R Studio, version 1.4.1106 (R Project for Statistical Computing).

## Results

### Patients

The Fried frailty criteria were available for 1446 of the 1518 randomized patients (95.3%) (eFigure 1 in Supplement 2). The frail group included 469 patients (32.4%; median age, 83 years [IQR, 80-86 years]; 240 women [51.2%] and 229 men [48.8%]) (Table 1). Frail patients were older compared with prefrail and robust patients. The median Montreal Cognitive Assessment score was lower among frail patients compared with prefrail and robust patients. Frail patients presented a higher GRACE (Global Registry of Acute Coronary Events) risk score compared with prefrail and robust patients. In addition, frail older patients had a higher burden of comorbidities compared with prefrail and robust patients, particularly a history of diabetes, kidney disease, previous myocardial infarction, previous percutaneous coronary intervention, peripheral vascular disease, chronic obstructive pulmonary disease, and congestive heart failure. Among frail older patients, the invasive treatment group comprised 231 patients and the conservative treatment group comprised 238 patients. Baseline characteristics of frail patients were generally similar between the 2 arms, as shown in eTable 1 in Supplement 2. There were no differences in medical therapy at hospital discharge between the invasive and conservative strategies among frail patients, except for a higher proportion of patients receiving aspirin at hospital discharge in the invasive group (eTable 1 in Supplement 2). No differences were observed in medical therapy at follow-up. Procedural characteristics are shown in Table 2. Among the 231 frail older patients randomized to the invasive strategy group, 192 (83.1%) underwent coronary angiography, with radial access used in 174 of 192 cases (90.6%). Reasons for not performing coronary angiography are shown in Table 2. Analysis of robust and prefrail patients are shown in Table 1, Table 2, and eTable 1 in Supplement 2.

**Table 1.** Baseline Characteristics of Robust, Prefrail, and Frail Patients

Characteristic	Patients^a^			*P* value
	Robust (n = 303)	Prefrail (n = 674)	Frail (n = 469)	
Sex				
Female	117 (38.6)	291 (43.2)	240 (51.2)	<.001
Male	186 (61.4)	383 (56.8)	229 (48.8)	
Age, y				
Median (IQR)	80 (78-83)	82 (79-86)	83 (80-86)	<.001
Distribution				
≥75 to <80	125 (41.3)	204 (30.3)	104 (22.2)	<.001
≥80 to <85	131 (43.2)	261 (38.7)	178 (38.0)	
≥85 to <90	40 (13.2)	159 (23.6)	136 (29.0)	
≥90 to <95	7 (2.3)	46 (6.8)	41 (8.7)	
≥95	0	4 (0.6)	10 (2.1)	
Days from admission to randomization, median (IQR)	2 (1-2.5)	2 (1-3)	2 (1-3)	<.001
MoCA score, median (IQR)	26 (23-27)	24 (21-27)	23 (19-26)	<.001
Impaired (MoCA score <26)	145/300 (48.3)	405/661 (61.3)	325/447 (72.7)	
Age-adjusted Charlson Comorbidity Index, median (IQR)	5 (4-6)	5 (4-6)	6 (5-7)	<.001
GRACE risk score, median (IQR)	130 (120-144)	135.5 (123-148)	139 (128-157)	<.001
Smoking status				
Current smoker	15/301 (5.0)	34/668 (5.1)	25/465 (5.4)	.79
Ex-smoker	131/301 (43.2)	318/668 (47.6)	210/465 (45.2)	
Never smoked	155/301 (51.2)	316/668 (47.3)	230/465 (49.5)	
Hypertension	198 (65.3)	431 (63.9)	315 (67.2)	.53
Diabetes	71 (23.4)	212 (31.5)	157 (33.5)	.009
Hypercholesterolemia	96/303 (31.7)	223/673 (33.1)	139/469 (29.6)	.46
History of kidney disease	49 (16.2)	123 (18.2)	121 (25.8)	<.001
Previous myocardial infarction	75 (24.8)	198 (29.4)	177 (37.7)	<.001
Previous PCI	54/303 (17.8)	120/674 (17.8)	111/468 (23.7)	.03
Previous CABG	32 (10.6)	76 (11.3)	64 (13.6)	.34
History of peripheral vascular disease	17 (5.6)	44 (6.5)	47 (10.0)	.03
History of TIA or stroke	42 (13.9)	94 (13.9)	82 (17.5)	.21
History of COPD	33 (10.9)	95 (14.1)	93 (19.8)	.002
History of congestive heart failure	19 (6.3)	40 (5.9)	75 (16.0)	<.001

Abbreviations: CABG, coronary artery bypass graft; COPD, chronic obstructive pulmonary disease; GRACE, Global Registry of Acute Coronary Events; MoCA, Montreal Cognitive Assessment; PCI, percutaneous coronary intervention; TIA, transient ischemic attack.

^a^ Data are presented as number (percentage) or number/total number (percentage) for sample sizes differing from the total for each column, unless otherwise indicated.

**Table 2.** Medical Therapy and Procedural Characteristics of Robust, Prefrail, and Frail Patients

Characteristic	Patients^a^			*P* value
	Robust	Prefrail	Frail	
**Discharge medical therapy**				
Patients, No.	303	674	469	NA
Antiplatelet therapies				
Aspirin	278 (91.7)	602 (89.3)	400 (85.3)	.01
P2Y12 receptor antagonist				
Total	280 (92.4)	625 (92.7)	426 (90.8)	.40
Clopidogrel	119 (39.3)	323 (47.9)	271 (57.8)	
Ticagrelor	160 (52.8)	301 (44.7)	153 (32.6)	
Prasugrel	1 (0.3)	1 (0.1)	2 (0.4)	
Antiplatelet therapy				
None	6(2.0)	16 (2.4)	5 (1.1)	<.001
Single	36 (11.9)	85 (12.6)	102 (21.7)	
Dual	261 (86.1)	571 (84.7)	362 (77.2)	
Anticoagulant				
Total	63 (20.8)	155 (23.0)	120 (25.6)	.29
Apixaban	24 (7.9)	54 (8.0)	38 (8.1)	
Rivaroxaban	15 (5.0)	32 (4.7)	29 (6.2)	
Edoxaban	10 (3.3)	13 (1.9)	8 (1.7)	
Dabigatran	1 (0.3)	4 (0.6)	1 (0.2)	
Warfarin	5 (1.7)	25 (3.7)	29 (6.2)	
Other	8 (2.6)	28 (4.2)	15 (3.2)	
Triple therapy	36 (11.9)	87 (12.9)	59 (12.6)	.89
Lipid-lowering therapy				
Total	280 (92.4)	618 (91.7)	411 (87.6)	.37
Atorvastatin	252 (83.2)	540 (80.1)	360 (76.8)	
Simvastatin	13 (4.3)	42 (6.2)	24 (5.1)	
Rosuvastatin	10 (3.3)	25 (3.7)	17 (3.6)	
Pravastatin	3 (1.0)	9 (1.3)	7 (1.5)	
Ezetimibe	2 (0.7)	2 (0.3)	3 (0.6)	
**Procedural characteristics**				
Invasive strategy				
Patients, No.	150	335	231	NA
Angiography performed				
Total	146 (97.3)	310 (92.5)	192 (83.1)	<.001
Radial access	128/146 (87.7)	277/310 (89.3)	174/192 (90.6)	.68
Time from admission to angiography, median (IQR), d	4 (3-7)	5 (4-7)	5 (3-9)	.03
Time from randomization to angiography, median (IQR), d	2 (1-4)	3 (1-4)	3 (1-5)	.50
Reason not performed				
Clinical decision	2/4 (50.0)	9/25 (36.0)	23/39 (59.0)	<.001
Participant decision	2/4 (50.0)	10/25 (40.0)	6/39 (15.4)	
Participant too unwell	0	4/25 (16.0)	8/39 (20.5)	
Participant died	0	1/25 (4.0)	2/39 (5.1)	
Not known	0	1/25 (4.0)	0	
Revascularization performed				
PCI	80/146 (54.8)	166/310 (53.5)	91/192 (47.4)	<.001
CABG	7/146 (4.8)	12/310 (3.9)	3/192 (1.6)	<.001
Time from admission to PCI, median (IQR), d	4 (2-6)	5 (3-7)	5 (3-8)	.04
Time from randomization to PCI, median (IQR), d	2 (1-4)	3 (1-4)	3 (1-5)	.85
Time from admission to CABG, median (IQR), d	19 (15-23)	14 (13-21)	16 (13-29)	.56

Abbreviations: CABG, coronary artery bypass surgery; NA, not applicable; PCI, percutaneous coronary intervention; P2Y12, P2Y purinergic receptor 12.

^a^ Data are presented as number (percentage) or number/total number (percentage) for sample sizes differing from the total for each column, unless otherwise indicated.

### Primary Outcome Among Frail Patients

**Figure 1.**
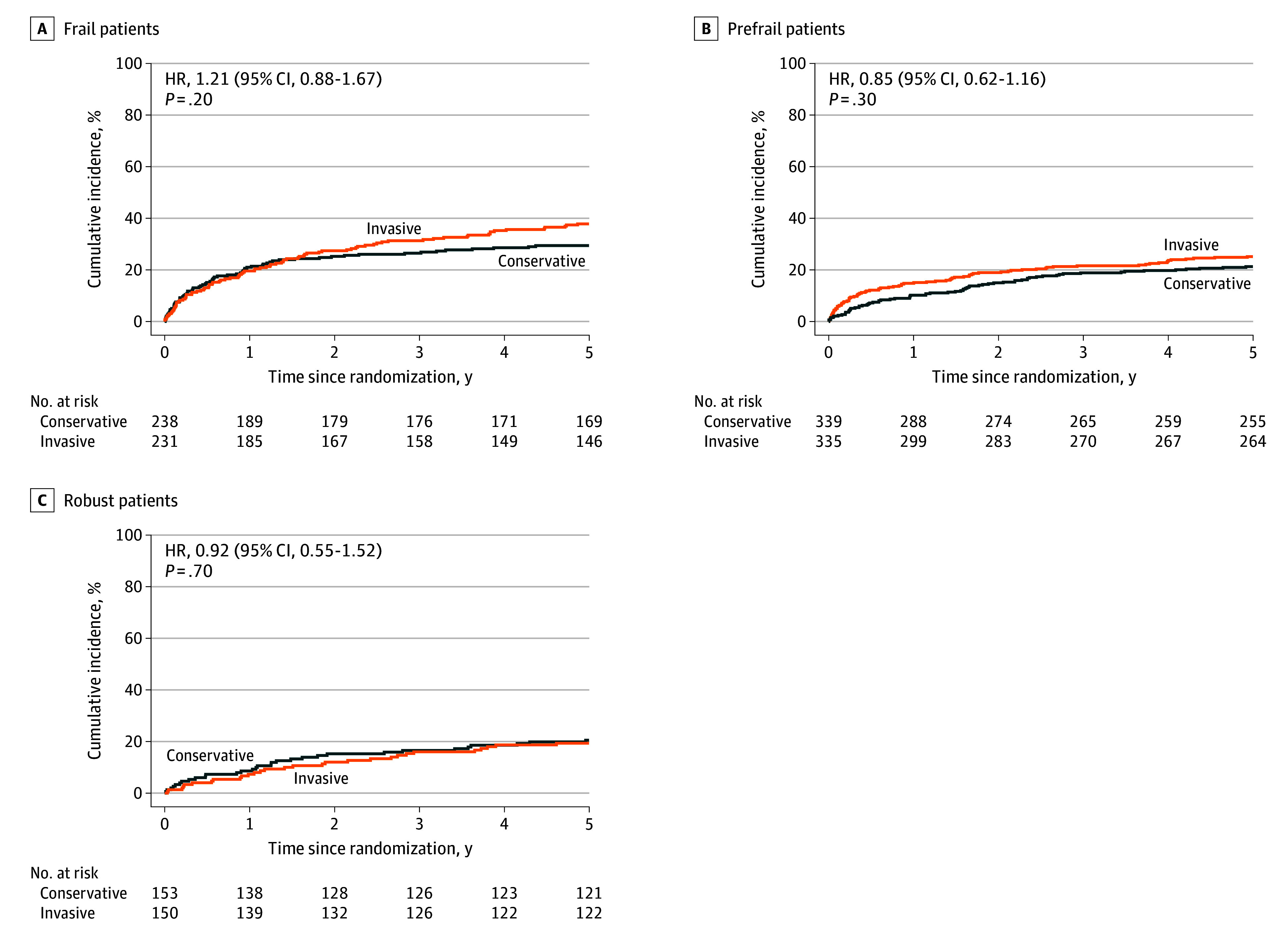
Survival Plots of Cumulative Incidence of Primary Outcome Among Frail, Prefrail, and Robust Patients Incidence of cardiovascular death or nonfatal myocardial infarction (the primary outcome) among frail (A), prefrail (B), and robust patients (C). Cumulative incidence was estimated with the Kaplan-Meier method. Hazard ratios (HRs) were estimated with Cox proportional hazards regression models. *P* values were calculated with the use of a log-rank test.

The primary outcome among frail patients occurred in 87 of 231 patients (37.7%) in the invasive group and in 70 of 238 patients (29.4%) in the conservative group (hazard ratio [HR], 1.21; 95% CI, 0.88-1.67; *P* = .20) over a median follow-up of 4.1 years (IQR, 2.8-4.6 years) (Table 3, Figure 1A; eTables 2 and 3 and eFigure 4 in Supplement 2). Cardiovascular death occurred in 59 of 231 patients (25.5%) in the invasive group and 44 of 238 patients (18.5%) in the conservative group (HR, 1.44; 95% CI, 0.97-2.10; *P* = .07) (Table 3; eTable 3 and eFigures 2 and 4 in Supplement 2). Nonfatal myocardial infarction occurred in 34 of 231 patients (14.7%) in the invasive group and in 33 of 238 patients (13.9%) in the conservative group (HR, 1.00; 95% CI, 0.61-1.63; *P* > .99) (Table 3; eTable 3 and eFigures 3 and 4 in Supplement 2).

**Table 3.** Primary and Secondary Outcomes of Frail, Prefrail, and Robust Patients

Outcome variable	Patients, No. (%)		Treatment effect, HR (95% CI)	*P* value^a^	*P* value for interaction^b^
	Invasive strategy	Conservative strategy			
**Frail patients**					
No.	231	238	NA	NA	NA
Primary outcome and its components					
Cardiovascular death or nonfatal MI^c^	87 (37.7)	70 (29.4)	1.21 (0.88-1.67)	.20	.04
Cardiovascular death^d^	59 (25.5)	44 (18.5)	1.44 (0.97-2.10)	.07	.24
Nonfatal MI^d^	34 (14.7)	33 (13.9)	1.00 (0.61-1.63)	>.99	.13
Secondary outcomes					
Death from any cause and nonfatal MI^d^	135 (58.4)	131 (55.0)	1.05 (0.82-1.33)	.70	.25
Death from any cause^d^	122 (52.8)	112 (47.1)	1.14 (0.88-1.16)	.30	.86
Noncardiovascular death^d^	63 (27.3)	69 (30.0)	1.07 (0.76-1.52)	.70	.57
Fatal and nonfatal MI^d^	42 (18.2)	37 (15.5)	1.11 (0.71-1.75)	.60	.08
Coronary angiography^c^	8 (3.5)	52 (21.8)	0.14 (0.07-0.23)	<.001	.14
Coronary revascularization^d^	6 (2.6)	28 (11.8)	0.17 (0.07-0.45)	<.001	.37
Stroke^d^	15 (6.5)	14 (5.9)	1.19 (0.60-2.51)	.60	.13
Transient ischemic attack^d^	4 (1.7)	2 (0.8)	2.07 (0.40-11.30)	.40	.53
Hospitalization for heart failure^d^	31 (13.4)	29 (12.2)	1.14 (0.68-1.90)	.60	.87
Bleeding (BARC type ≥2)^d^	26 (11.3)	16 (6.7)	1.64 (0.86-3.15)	.10	.95
**Prefrail patients**					
No.	335	339	NA	NA	NA
Primary outcome and its components					
Cardiovascular death and nonfatal MI^d^	72 (21.5)	86 (25.4)	0.85 (0.62-1.16)	.30	NA
Cardiovascular death^d^	42 (12.5)	46 (13.6)	0.96 (0.63-1.47)	.90	NA
Nonfatal MI^d^	37 (11.0)	51 (15.0)	0.75 (0.49-1.14)	.20	NA
Secondary outcomes					
Death from any cause and nonfatal MI^d^	136 (40.6)	130 (38.3)	1.09 (0.85-1.38)	.50	NA
Death from any cause^d^	112 (33.4)	99 (29.2)	1.25 (0.95-1.64)	.10	NA
Noncardiovascular death^d^	70 (20.9)	53 (15.6)	1.44 (1.00-2.06)	.05	NA
Fatal and nonfatal MI^d^	39 (11.6)	54 (15.9)	0.74 (0.49-1.12)	.20	NA
Coronary angiography^c^	18 (5.4)	77 (22.7)	0.19 (0.11-0.32)	<.001	NA
Coronary revascularization^d^	13 (3.9)	41 (12.1)	0.26 (0.13-0.50)	<.001	NA
Stroke^d^	12 (3.6)	18 (5.3)	0.87 (0.40-1.88)	.70	NA
Transient ischemic attack^d^	12 (3.6)	4 (1.2)	4.08 (1.15-14.40)	.02	NA
Hospitalization for heart failure^d^	43 (12.8)	36 (10.6)	1.33 (0.72-1.77)	.60	NA
Bleeding (BARC type ≥2)^d^	25 (7.5)	24 (7.1)	1.10 (0.62-1.94)	.70	NA
**Robust patients**					
No.	150	153	NA	NA	NA
Primary outcome and its components					
Cardiovascular death and nonfatal MI^d^	30 (20.0)	31(20.3)	0.92 (0.55-1.52)	.70	NA
Cardiovascular death^d^	15 (10.0)	10 (6.5)	1.45 (0.64-3.25)	.40	NA
Nonfatal MI^d^	16 (10.7)	24 (15.7)	0.68 (0.36-1.30)	.20	NA
Secondary outcomes					
Death from any cause and nonfatal MI^d^	38 (25.3)	43 (28.1)	0.85 (0.55-1.32)	.50	NA
Death from any cause^d^	29 (19.3)	22 (14.4)	1.30 (0.74-2.28)	.40	NA
Noncardiovascular death^d^	14 (9.3)	12 (7.8)	1.38 (0.62-3.04)	.40	NA
Fatal and nonfatal MI^d^	18 (12.0)	25 (16.3)	0.73 (0.40-1.35)	.30	NA
Coronary angiography^d^	15 (10.0)	47 (30.7)	0.28 (0.16-0.51)	<.001	NA
Coronary revascularization^d^	9 (6.0)	29 (19.0)	0.32 (0.15-0.67)	.002	NA
Stroke^d^	4 (2.7)	6 (3.9)	0.33 (0.07-1.64)	.20	NA
Transient ischemic attack^d^	2 (1.3)	3 (2.0)	0.68 (0.11-4.07)	.70	NA
Hospitalization for heart failure^d^	7 (4.7)	6 (3.9)	0.70 (0.25-1.98)	.50	NA
Bleeding (BARC type ≥2)^d^	11 (7.3)	6 (3.9)	2.26 (0.80-6.51)	.10	NA

Abbreviations: BARC, Bleeding Academic Research Consortium; HR, hazard ratio; MI, myocardial infarction; NA, not applicable.

^a^ *P* values were calculated using a log-rank test.

^b^ *P* values for interaction between continuous frailty score and treatment.

^c^ Analyses did not satisfy the proportional hazards assumption of the Cox model. Analyses of the restricted mean event-free time were also performed and gave consistent interpretation.

^d^ Analyses satisfied the proportional hazards assumption of the Cox model.

### Secondary Outcomes Among Frail Patients

No differences were found in secondary outcomes between the invasive and conservative groups among frail patients. However, there was a reduced risk of coronary angiography and revascularization in the invasive group (coronary angiography: HR, 0.14; 95% CI, 0.07-0.23; *P* < .001; coronary revascularization: HR, 0.17, 95% CI, 0.07-0.45; *P* < .001) (Table 3; eTable 2 in Supplement 2).

### Primary and Secondary Outcomes Among Robust and Prefrail Patients

The primary outcome and its components did not differ between the invasive and conservative groups among prefrail and robust patients (Table 3, Figure 1B and C; eTable 3 and eFigure 2B and C, eFigure 3B and C, and eFigure 4 in Supplement 2). The risk of coronary angiography and revascularization was significantly reduced among robust patients treated with the invasive approach (coronary angiography: HR, 0.28; 95% CI, 0.16-0.51; *P* < .001; coronary revascularization: HR, 0.32; 95% CI, 0.15-0.67; *P* = .002) (Table 3). Similarly, prefrail patients in the invasive group demonstrated a significant risk reduction of coronary angiography and revascularization (coronary angiography: HR, 0.19; 95% CI, 0.11-0.32; *P* < .001; coronary revascularization: HR, 0.26; 95% CI, 0.13-0.50; *P* < .001). Risk of transient ischemic attack was higher among prefrail patients managed with the invasive strategy compared with the conservative strategy (HR, 4.08; 95% CI, 1.15-14.40; *P* = .02).

**Figure 2.**
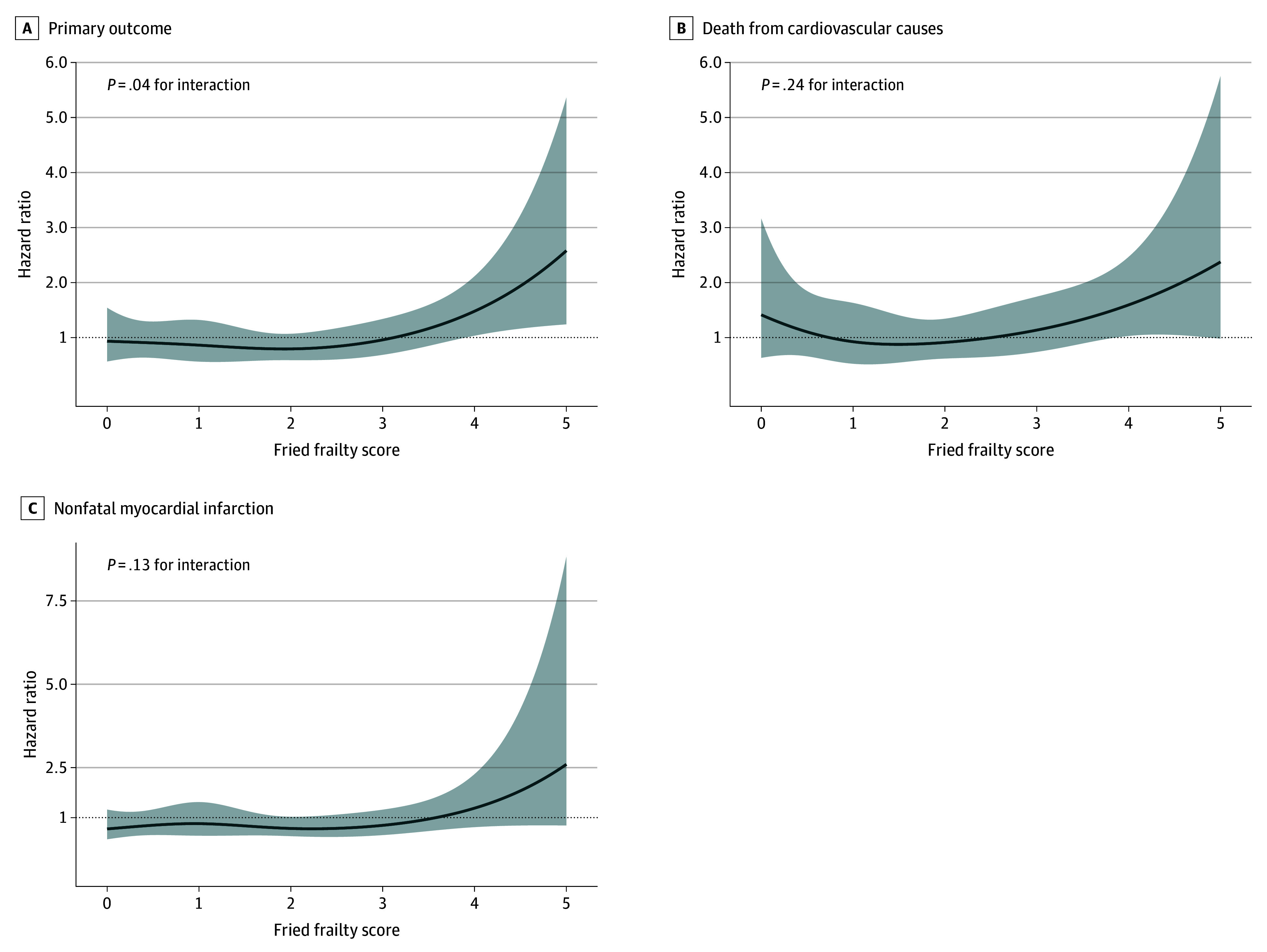
Line Graphs of Treatment Effect on the Primary Outcome and Its Components Across the Fried Frailty Score, With Tests for Interaction Between Frailty and Treatment Strategy Cardiovascular death or nonfatal myocardial infarction (the primary outcome) (A), death from cardiovascular cause (B), and nonfatal myocardial infarction (C) across Fried frailty scores. The *P* value for interaction refers to the interaction between the Fried frailty score and treatment strategy. Shading indicates 95% CIs.

The effect of treatment varied across levels of frailty. In the categorical subgroup analysis, the *P* value for interaction for the primary end point was .07, which did not reach statistical significance (eFigure 4 in Supplement 2). However, when frailty was examined as a continuous variable, a statistically significant interaction emerged for the primary end point (*P* = .04 for interaction), suggesting that the treatment effect may differ across the frailty spectrum (Figure 2A). For cardiovascular death and nonfatal myocardial infarction, no significant interaction was observed across levels of frailty either categorically or continuously (Figure 2B and C; eFigure 4B and C in Supplement 2), indicating that the treatment effect for these outcomes did not vary by frailty status.

### Procedural Complications

Frail patients showed a numerically higher rate of procedural complications compared with prefrail and robust patients, although this did not reach statistical significance (frail, 16 of 192 patients [8.3%] vs prefrail, 12 of 310 [3.9%] and robust, 5 of 146 [3.4%]). However, the incidence of each individual procedural complication was below 1% in the 3 groups except for type 2 bleeding among frail patients (Bleeding Academic Research Consortium [BARC] criteria; 5 of 192 patients [2.6%]) and increases in serum creatinine concentration among frail patients (3 of 192 patients [1.6%]).

## Discussion

This exploratory subgroup analysis of the SENIOR-RITA trial examined the outcomes of frail older patients with NSTEMI managed with an invasive vs a conservative strategy. Our findings suggest that, among frail patients, an invasive approach was not associated with a statistically significant reduction in the primary composite outcome or its individual components. Conversely, there was a numerical increase in adverse events, particularly cardiovascular death, in the invasive group, although this did not reach statistical significance.

In our analysis, frail patients randomized to an invasive strategy had numerically higher rates of the primary outcome (37.7% vs 29.4%), driven by increased cardiovascular death (25.5% vs 18.5%), compared with those managed conservatively. Although not statistically significant, these findings raise concerns regarding the role of invasive management in this vulnerable subgroup. Moreover, a significant interaction was found between frailty status as a continuous variable and the risk of the primary outcome. This result highlights that invasive management may be harmful among older patients at the highest levels of frailty with NSTEMI. The observed trend toward worse outcomes among the frail patients in the invasive group may reflect several factors: increased vulnerability to procedural complications and potentially limited capacity to benefit from revascularization due to a higher burden of comorbidities or limited life expectancy. These considerations support the European Society of Cardiology guidelines’ recommendation for a personalized and holistic approach in managing frail patients with ACS, emphasizing shared decision-making, and weighing the risks and benefits of invasive interventions.^8^ Our study endorses the evidence that frailty should be systematically assessed and incorporated into clinical decision-making. It also highlights the need for frailty-specific clinical trials, as frail patients remain underrepresented in cardiovascular research.

The primary results of the SENIOR-RITA trial demonstrated a reduced risk of nonfatal myocardial infarction with an invasive treatment strategy (HR, 0.75; 95% CI, 0.57-0.99).^10^ Although a difference was observed in the overall cohort (1518 patients), no significant effect emerged in the frailty subgroup analysis. This finding may reflect higher competing cardiovascular risks and greater vulnerability to complications among frail patients. Otherwise, the result may suggest that the effect is not primarily driven by the subgroup under investigation or, alternatively, that the analysis was underpowered to detect a meaningful difference within that subgroup.

Our results are consistent with the findings of the MOSCA-FRAIL trial, which also failed to demonstrate a clinical benefit from a routine invasive approach among frail older adults with NSTEMI.^9^ In particular, the trial did not find a significant difference between the 2 strategy groups but highlighted an increased risk of bleeding requiring hospitalization in the invasive strategy arm.^9^ However, our study extends these observations to a larger cohort, approximately 3 times the size of the MOSCA-FRAIL trial, supporting that frailty may attenuate or even reverse the expected benefits of early invasive management in NSTEMI. In our study, a trend toward a higher bleeding rate was observed in the invasive group. This finding may be explained not only by the increased vulnerability of frail older patients receiving antiplatelet therapy but also by the higher proportion of patients in the invasive group receiving aspirin at hospital discharge.^17^

Frailty is increasingly recognized as a major determinant of clinical outcomes among older patients with cardiovascular disease.^6^ Although invasive management is standard care in the general population of patients with NSTEMI, its role among frail patients remains controversial.^8,10^ Among frail patients randomized to the invasive arm, 16.9% did not undergo coronary angiography, primarily due to clinical decisions. This finding reflects clinical decision-making in a highly vulnerable population. Frailty is often accompanied by clinical complexity and rapid changes in a patient’s condition, leading clinicians to reasonably defer invasive procedures even when initially planned.

Secondary outcomes demonstrated a significantly lower risk of coronary angiography and revascularization in the invasive group, while procedural complications did not differ significantly between frail and nonfrail patients, except for BARC type 2 bleeding and increases in serum creatinine concentration. This finding suggests that frailty alone may not dramatically increase procedural risk, but it could influence the clinical benefit of invasive strategies.

### Strengths and Limitations

This study has some strengths. SENIOR-RITA is the first randomized clinical trial to date to include such a high proportion of frail patients, with 32.4% of participants classified as frail. This represents a key strength of the study, allowing for a more representative assessment of treatment strategies in this high-risk population. We have previously described that the revascularization rate in SENIOR-RITA is consistent with previous studies. The median time from admission to angiography in SENIOR-RITA was 5 days (IQR, 3-7 days), as was the aim in the trial protocol. The reason for this was due to a longer time required to recruit patients with frailty and cognitive impairment. It was crucial that SENIOR-RITA adequately represented a contemporary older population; this meant more time was required to explain the trial to patients and give them more time to decide whether to participate.^18^

However, our analysis also has limitations. The lack of detailed information on the specific causes of cardiovascular death limited our ability to explore the underlying mechanisms driving these events. Patients with more advanced frailty were less likely to be considered candidates for randomization, which restricts generalizability. Although only 1 in 5 screened patients was ultimately enrolled in SENIOR-RITA, reflecting the known challenges of including highly vulnerable older adults in clinical research, the clinical and demographic similarity between randomized and nonrandomized patients supports the representativeness of our trial population.^11^ The size of the frail subgroup may limit the statistical power to detect modest but clinically relevant differences; therefore, the findings should be interpreted with caution. The median delay to angiography (3 days after randomization among frail patients) may have attenuated the potential benefit of the invasive strategy, particularly for frail older adults among whom delays are frequent, as also noted in contemporary evidence.^18^ Landmark or time-varying analyses were not performed and represent a limitation of this exploratory subgroup analysis. In our work, frailty was assessed using the Fried frailty criteria, while other instruments, such as the Rockwood Clinical Frailty Scale, capture different dimensions of vulnerability. Because these tools reflect distinct conceptual models (physical performance vs deficit accumulation), they may classify patients differently and lead to different estimates of treatment effect. Therefore, our findings should be interpreted in the context of the frailty measure used. The SENIOR-RITA randomization was stratified by Rockwood frailty^2^ and not by the Fried criteria used in this analysis; residual imbalances between treatment groups persist within the Fried frailty subgroups, and confounding cannot be excluded. In addition, as this was an exploratory subgroup analysis, no adjustment for multiple comparisons was applied; therefore, the findings should be interpreted as hypothesis generating rather than confirmatory. Nevertheless, our analysis provides valuable insights into the optimal care of frail older adults with NSTEMI. Patients with more advanced frailty were less likely to be considered candidates for randomization, which may limit generalizability. Although only 1 in 5 screened patients was ultimately enrolled in SENIOR-RITA, reflecting the known challenges of including highly vulnerable older adults in clinical research, the clinical and demographic similarity between randomized and nonrandomized patients supports the representativeness of our trial population.

## Conclusions

In this prespecified subgroup analysis of frail older patients with NSTEMI, an invasive strategy did not reduce the risk of cardiovascular death or nonfatal myocardial infarction compared with a conservative strategy. Although the study may be underpowered, the results indicate that invasive management may not provide clinical benefit for older populations at the highest levels of frailty and underscore the need for individualized, frailty-informed treatment strategies.

## Notes

Supplement 1.
Trial Protocol and Statistical Analysis Plan

Supplement 2.
eMethods.
**eTable 1.** Baseline Demographics, Clinical Characteristics and Medical Therapy of Frail, Pre-Frail and Robust Patients as Per Randomized Strategy **eTable 2.** Restricted Mean Event-Free Time to Account for Non-Proportional Hazards in Frail and Pre-Frail Patients **eTable 3.** Primary Outcome Accounting for Competing Risk In Frail, Pre-Frail and Robust Patients—Fine and Gray Regression Model **eFigure 1.** Subgroup-Study Flow Chart **eFigure 2.** Cumulative Incidence of Cardiovascular Death in Frail, Pre-Frail and Robust Patients **eFigure 3.** Cumulative Incidence of Non-Fatal Myocardial Infarction in Frail, Pre-Frail and Robust Patients **eFigure 4.** Treatment Effect for Primary Outcome and components of Primary Outcome With Interaction Between Frailty and Strategy Arm in Robust, Pre-Frail and Frail Patients

Supplement 3.
Data Sharing Statement
