# Supplementary material for: Invasive vs Conservative Strategy for Frail Older Patients With Myocardial Infarction: A Secondary Analysis of the SENIOR-RITA Randomized Clinical Trial
Source: JAMA Netw Open. 2026 Apr 21;9(4):e267316. doi: 10.1001/jamanetworkopen.2026.7316 (PMC13100840; doi:10.1001/jamanetworkopen.2026.7316)
Supplement: Supplement 1. — Trial Protocol and Statistical Analysis Plan [file jamanetwopen-e267316-s001.pdf]

Table of contents

1. **Trial protocol version 1.0**
2. **Trial protocol version 3.0**
3. **Trial protocol summary of changes**
4. **SAP version 1.0**
5. **SAP version 2.0**
6. **SAP summary of changes**

**Full Title:**	The British Heart Foundation older patients with non-ST SEgmeNt elevatIOn myocaRdial infarction Randomized Interventional TreAtment Trial
**Short Title/Acronym:**	The BHF SENIOR-RITA Trial
**Protocol Version Number & Date:**	Version 1.0<br>14 June 2016

Statement: This protocol has regard for the HRA guidance.

RESEARCH REFERENCE NUMBERS

**IRAS Number:**	**204031**
**NHS REC Reference:**	**16/NE/0238**
**Research Registry & References:**	**TBC**

RESEARCH SPONSOR

**Sponsor Name:**	Newcastle upon Tyne Hospitals NHS Foundations Trust
**Sponsor Reference:**	**TBC**

RESEARCH FUNDER

**Funder Name:**	British Heart Foundation
**Funder Reference:**	CS/15/7/31679

SIGNATURE PAGE

The undersigned confirm that the following protocol has been agreed and accepted. The Chief Investigator agrees to conduct the trial in compliance with the approved protocol and will adhere to the Research Governance Framework, Good Clinical Practice (GCP) guidelines, the relevant Standard Operating Procedures and other regulatory requirements as applicable.

I agree to ensure that the confidential information contained in this document will not be used for any other purpose other than the evaluation or conduct of the investigation without the prior written consent of the Sponsor.

Representative of the Research Sponsor

Name: **Ms. Stephanie Blacklock**

Position: **RM&G Manger**

Signature: **Date:**

Chief Investigator

Name: **Dr. Vijay Kunadian**

Position: **Senior Lecturer, Newcastle University and Honorary Consultant Interventional Cardiologist, Newcastle upon Tyne Hospitals**

Signature: **Date:**

Statistician

Name: **Dr. Deborah Stocken**

Position: **Head of Statistics, Institute of Health and Society & Deputy Director, Newcastle Clinical Trials Unit, Newcastle University**

Signature: **Date:**

Senior Trial Manager

Name: **Dr Lesley Hall**

Position: **Senior Trial Manager, NCTU**

Signature: **Date:**

Trial Manager

Name: **Miss Jaki Begum**

Position **Trial Manager, NCTU**

Signature: **Date**

Database Manager

Name: **Mr Jonathan Prichard**

Position: **Database manager, NCTU**

Signature: **Date:**

KEY TRIAL CONTACTS

**Chief Investigator**	Dr. Vijay Kunadian<br>Senior Lecturer/Honorary Consultant Interventional Cardiologist<br>M3.131 3rd Floor William Leech Building<br>Medical School<br>Newcastle upon Tyne<br>NE2 4HH<br>United Kingdom<br>Tel: +44 (0) 191 208 5797<br>E-mail: vijay.kunadian@newcastle.ac.uk
**Senior Trial Manager**	Dr. Lesley Hall<br>Newcastle Clinical Trials Unit<br>Newcastle University<br>1-4 Claremont Terrace<br>Newcastle upon Tyne<br>NE2 4AE<br>United Kingdom<br>Telephone: +44 (0) 191 208 4584<br>Email: lesley.hall@ncl.ac.uk
**Trial Manager**	Miss Jaki Begum<br>Newcastle Clinical Trials Unit<br>Newcastle University<br>1-4 Claremont Terrace<br>Newcastle upon Tyne<br>NE2 4AE<br>United Kingdom<br>Telephone: +44 (0) 191 208 8753<br>Email: jaki.begum@newcastle.ac.uk
**Database Manager**	Mr. Jonathan Pritchard<br>Newcastle Clinical Trials Unit<br>Faculty of Medical Sciences<br>Newcastle University<br>1-4 Claremont Terrace<br>Newcastle upon Tyne<br>NE2 4AE<br>Telephone: +44 (0) 191 208 2518<br>Email: jonathan.prichard@newcastle.ac.uk
**Trial Statistician**	Dr. Gillian Libby<br>Institute of Health and Society<br>Baddiley Clark Building<br>Richardson Road<br>Newcastle upon Tyne<br>NE2 4AX<br>Email: Gillian.Libby@newcastle.ac.uk

**Sponsor**	Ms. Stephanie Blacklock<br>RM&G Manager<br>Newcastle upon Tyne Hospitals NHS Foundations Trust<br>Level 1, Regent Point<br>Regent Farm Road<br>Gosforth<br>Newcastle upon Tyne<br>NE3 3HD<br>Tel: +44 (0) 191 2825969<br>Email: Andrew.Johnston@nuth.nhs.uk
**Funder(s)**	Mrs. Alex Mazzetta<br>Head of Research Funds<br>British Heart Foundation<br>Greater London House, 180 Hampstead Road<br>London<br>NW1 7AW<br>Tel: 020 7554 0434<br>Email: research@bhf.org.uk
**Collaborators/Co-Investigators**	Professor David Newby<br>BHF Professor of Cardiology<br>Centre for Cardiovascular Science<br>Queen's Medical Research Institute<br>49 Little France Crescent<br>University of Edinburgh<br>Edinburgh<br>EH16 4TJ<br>Tel: +44 (0) 131 242 6515<br>Email: d.e.newby@ed.ac.uk<br><br>Professor Elaine McColl<br>Director Newcastle Clinical Trials Unit<br>Newcastle University<br>1-2 Claremont Terrace<br>Newcastle upon Tyne<br>NE2 4AE<br>Tel: +44 (0) 191 222 7260<br>E-mail: elaine.mccoll@newcastle.ac.uk<br><br>Professor Luke Vale<br>Health Foundation Chair in Health Economics<br>Institute of Health & Society<br>Newcastle University<br>Baddiley-Clark Building<br>Richardson Road<br>Newcastle upon Tyne<br>NE2 4AA<br>Tel: +44 (0) 191 2085590<br>Email: luke.vale@newcastle.ac.uk

	Professor Stuart Parker<br>William Leech Professor of Geriatric Medicine<br>Institute for Ageing and Health<br>Newcastle University, Campus for Ageing and Vitality<br>Newcastle upon Tyne<br>NE4 5PL<br>Tel: +44 (0) 191 2081215<br>Email: stuart.parker@ncl.ac.uk<br><br>Dr. Deborah Stocken<br>Senior Lecturer in Clinical Trials and Biostatistics<br>Institute of Health & Society<br>Baddiley Clark Building<br>Richardson Road<br>Newcastle upon Tyne<br>NE2 4AX<br>Tel: +44 (0) 191 208 3410<br>Email: deborah.stocken@newcastle.ac.uk<br><br>Ms. Jennifer Hislop<br>Research Associate<br>Health Economics Group<br>Institute of Health & Society<br>Baddiley-Clark Building<br>Richardson Road<br>Newcastle-upon-Tyne<br>NE2 4AX<br>Tel: +44 (0) 191 208 7040<br>Email: jenni.hislop@newcastle.ac.uk<br><br>Professor Robert Storey<br>Professor and Honorary Consultant in Cardiology<br>Department of Cardiovascular Science<br>University of Sheffield<br>Medical School<br>Beech Hill Road<br>Sheffield<br>S10 2RX<br>Tel: +44 (0) 114 226 1124<br>Email: r.f.storey@sheffield.ac.uk<br><br>Professor Colin Berry<br>Professor of Cardiology and Imaging<br>RC309 Level C3<br>Institute of C&MS<br>BHF GCRC<br>Glasgow G12 8TA<br>Tel: +44 (0) 1413301671<br>Email: Colin.Berry@glasgow.ac.uk

	Dr. Mark de Belder<br>Consultant Interventional Cardiologist<br>The James Cook University Hospital<br>Marton Road<br>Middlesbrough<br>TS4 3BW<br>Tel: +44 (0) 1642 854620<br>Email: mark.debelder@stees.nhs.uk
**Committees**	**Trial Steering Committee (Independent)**<br>Professor Keith Fox (Chair)<br>Emeritus Professor of Cardiology<br>Centre for Cardiovascular Science<br>Queen's Medical Research Institute<br>49 Little France Crescent, University of Edinburgh<br>Edinburgh, EH16 4TJ<br>Email: k.a.a.fox@ed.ac.uk<br><br>Professor Gary Ford<br>Chief Executive Officer of the Oxford AHSN<br>Oxford Academic Health Science Network<br>Magdalen Centre North, Oxford Science Park<br>OX4 4GA<br>Tel: +44 (0) 1865 784957<br>Email: Gary.Ford@ouh.nhs.uk<br><br>Professor Marcus Flather<br>Professor of Medicine and Honorary Consultant Cardiologist<br>Norwich Medical School<br>University of East Anglia Norwich NR4 7TJ<br>Tel: +44 (0) 1603 591062<br>Email: marcus.flather@nnuh.nhs.uk<br><br>Professor Rajesh Kharbanda<br>Associate Professor of Cardiovascular Medicine, Consultant Cardiologist<br>University of Oxford, Headley Way,<br>Oxford, OX3 9DU.<br>Tel: +44 (0) 1865 220325<br>Email: Rajesh.Kharbanda@ouh.nhs.uk<br><br>Mr. David Inness (Lay member)<br>VoiceNorth<br>Faculty of Medical Sciences<br>Biomedical Research Building<br>Newcastle University, Campus for Ageing and Vitality<br>Newcastle upon Tyne<br>NE4 5PL<br>Tel: +44 (0) 191 208 1287<br>Email: dbi@talktalk.net

	Dr. Shannon Amoils (BHF Representative)<br>British Heart Foundation<br>Greater London House<br>180 Hampstead Road<br>London NW1 7AW<br>Tel: 020 7554 0360<br>Email: amoilss@bhf.org.uk<br><br>**Data Monitoring Committee**<br>Professor Stuart Pocock (Chair)<br>Room, G34a, Medical Statistics Unit<br>LSHTM, Keppel Street<br>London<br>WC1E 7HT<br>Tel: +44 (0)20 7927 2413<br>Fax: 020 7637 2853<br>Email: stuart.pocock@lshtm.ac.uk<br><br>Professor Ajay Shah<br>King's College London<br>The James Black Centre<br>125 Coldharbour Lane<br>London SE5 9NU<br>Tel: +44 (0)20 7848 5189<br>Email: ajay.shah@kcl.ac.uk<br><br>Professor Robert Guigliano<br>TIMI Study Group<br>Office Level One, 350 Longwood Avenue<br>Boston, MA 02115<br>Tel: (617) 278-0145 (800) 385-4444<br>Fax: (617) 734-7329 (888) 249-5261<br>E-mail: RGIUGLIANO@partners.org<br><br>Dr. Ian Reeves<br>Consultant Geriatrician<br>Greater Glasgow and Clyde Valley<br>Glasgow<br>United Kingdom<br>Email: i.reeves@nhs.net<br><br>Professor Stephen Leslie<br>Consultant Cardiologist (NHS Highland)<br>Associate Director Research, Development & Innovation<br>Cardiac Unit<br>Raigmore Hospital, Old Perth Road<br>Inverness, IV2 3UJ<br>Tel: 01463 705459<br>Email: stephen.leslie@nhs.net

	**Clinical Events Committee**<br>Dr. Martin Denvir<br>Consultant Cardiologist<br>Centre for Cardiovascular Science<br>Queen's Medical Research Institute<br>49 Little France Crescent<br>University of Edinburgh<br>Edinburgh<br>EH16 4TJ<br>Tel: 0131 242 9236<br>E-mail: martin.denvir@ed.ac.uk<br><br>Dr. Anoop Shah<br>Clinical Lecturer<br>Centre for Cardiovascular Science<br>Queen's Medical Research Institute<br>49 Little France Crescent<br>University of Edinburgh<br>Edinburgh<br>EH16 4TJ<br>Tel: 0131 242 6537<br>E-mail: Anoop.Shah@ed.ac.uk<br><br>Dr. Ifti Haq<br>Consultant Cardiologist<br>Newcastle upon Tyne Hospitals NHS Foundations Trust<br>Newcastle upon Tyne<br>NE7 7NDN<br>Tel: 0191 2336161<br>E-mail: ifti.haq@nuth.nhs.uk<br><br>**Trial Management Group**<br>Dr. Vijay Kunadian<br>Professor Dave Newby<br>Professor Elaine McColl<br>Professor Luke Vale<br>Dr. Deborah Stocken<br>Professor Stuart Parker<br>Dr. Lesley Hall<br>Mr. Jon Prichard<br>Mrs. Joan Farrington<br>Ms. Jenni Hislop<br>Mrs. Laura Robertson<br>Dr. Gillian Libby
**Trial Website**	TBC

TRIAL SUMMARY

**Trial Title**	The British Heart Foundation older patients with non-ST SEgmeNt elevatIOn myocaRdial infarction Randomized Interventional TreAtment Trial
**Acronym**	SENIOR-RITA
**Summary of Trial Design**	SENIOR-RITA is a multicentre prospective open-label trial randomizing patients presenting with type 1 NSTEMI aged ≥75 years between invasive and conservative treatment strategies, to compare time to cardiovascular death or non-fatal MI within one year from randomization.
**Participant Population**	Older patients aged ≥75 years presenting with type 1 NSTEMI
**Planned Sample Size**	2300
**Number of Sites**	Approximately 30 centres
**Intervention Duration**	During index presentation with type 1 NSTEMI
**Follow Up Duration**	1 Year
**Planned Trial Period**	5 Years
**Primary Objective**	To determine the impact of a routine invasive strategy on one-year cardiovascular death and non-fatal myocardial infarction (MI) compared with a conservative treatment strategy in older patients (≥75 years) with NSTEMI.
**Secondary Objectives**	To determine the impact of a routine invasive strategy compared with a conservative strategy on:<br>1. All-cause death<br>2. Cardiovascular or non-cardiovascular death<br>3. Recurrent myocardial infarction<br>4. Urgent coronary revascularisation<br>5. Recurrent hospitalisation for myocardial infarction<br>6. Hospitalization for heart failure<br>7. Stroke<br>8. Bleeding (BARC ≥2)<br>9. Procedural and in-hospital complications<br>10. The length of time spent at home<br>11. Frailty and quality of life<br>12. Cost-effectiveness
**Intervention**	Coronary angiography ± revascularisation and optimal medical therapy *versus* optimal medical therapy alone

Contents

RESEARCH REFERENCE NUMBERS 2
SIGNATURE PAGE 3
KEY TRIAL CONTACTS 5
TRIAL SUMMARY 11
GLOSSARY OF ABREVIATIONS 15
RESPONSIBILITIES 16
1. BACKGROUND and RATIONALE 18
2. OBJECTIVES AND OUTCOME MEASURES 22
2.1. Primary Objective 22
2.2. Secondary Objectives 22
2.3. Outcome Measures 22
3. TRIAL DESIGN 24
4. STUDY SETTING 25
5. ELIGIBILITY CRITERIA 26
5.1. Inclusion Criteria 26
5.2. Exclusion Criteria 26
6. TRIAL PROCEDURES 27
6.1. Patient Identification and Screening 27
6.2. Consent 27
6.3. Randomization 27
6.4. Data collection 28
6.5. Follow-up 29
6.6. Schedule of events 30
6.7 Withdrawal Criteria 31
6.8 End of Trial 31
7. TRIAL INTERVENTIONS 32
7.1. Treatment Strategy 32
7.1.1. Schedule & Modifications 32
7.1.2. Known Risks 32
7.2. Concomitant Medications & Therapies 32
8. SAFETY REPORTING 33
8.1. Definitions 33
8.2. Severity (Intensity) of Adverse Events and Reactions 33

8.3. Assessment of Causality 34
8.4. Recording and Reporting AEs and SAEs 34
8.5. Responsibilities 35
8.6. Notification of Deaths 36
8.7. Reporting Urgent Safety Measures 36
9. STATISTICAL CONSIDERATIONS 37
9.1. Analysis Populations 37
9.2. Statistical Analyses 37
9.2.1. Analyses of the Primary Outcome Measure 37
9.2.2. Analyses of Secondary Outcome Measures 37
9.2.3. Subgroup Analyses 38
9.2.4. Planned Additional Analyses 38
9.2.5. Interim Analyses and Criteria for the Premature Termination of the Trial 38
9.3. Sample Size Calculations 39
10. ECONOMIC EVALUATION 40
10.1. Assessment of Cost 40
10.2. Economic Analysis 40
11. DATA HANDLING 42
11.1. Data Collection Tools and Source Document Identification 42
11.2. Data Handling and Record Keeping 42
11.3. Access to Data 42
11.4 Archiving 42
12. MONITORING, AUDIT & INSPECTION 43
13. ETHICAL AND REGULATORY CONSIDERATIONS 45
13.1. Research Ethics Committee Review and Reports 45
13.2. Public and Patient Involvement 45
13.3. Regulatory Compliance 45
13.4. Protocol Compliance 45
13.5. Notification of Serious Breaches to GCP and/or the Protocol 45
13.6. Data Protection and Patient Confidentiality 46
13.7. Indemnity 46
13.8. Amendments 46
14. DISSEMINATION POLICY 47
15. REFERENCES 48

16. APPENDICES....51
16.1. Definitions....51
16.2. Safety Reporting Diagram....53
16.3. Amendment History....54
16.4. Fried Frailty Index....55
16.5. Rockwood Frailty Index....56
16.6. Bleeding Academic Research Consortium definition for bleeding....57
16.7. EQ-5D-5L....58

GLOSSARY OF ABREVIATIONS

ABBREVIATION	DEFINITION
ACS	Acute coronary syndrome
AE	Adverse Event
AR	Adverse Reaction
CABG	Coronary artery bypass surgery
CAD	Coronary artery disease
CEAC	Cost-effectiveness acceptability curve
CEC	Clinical events committee
CHD	Coronary heart disease
CI	Chief Investigator
CRF	Case Report Form
DMC	Data Monitoring Committee
GBP	Pound Sterling
GCP	Good Clinical Practice
HRA	Health Research Authority
HR	Hazard Ratio
ICF	Informed Consent Form
IRMER	Ionising Radiation (Medical Exposure) Regulations
ISF	Investigator Site File
ISRCTN	International Standard Randomized Controlled Trials Number
MI	Myocardial infarction
MoCA	Montreal Cognitive Assessment
NCTU	Newcastle Clinical Trials Unit
NHS	National Health Service
NSTEACS	Non ST elevation acute coronary syndrome
NSTEMI	Non ST elevation myocardial infarction
PCI	Percutaneous coronary intervention
PI	Principal Investigator
PIS	Participant Information Sheet
QA	Quality Assurance
QALY	Quality-Adjusted Life Year
QC	Quality Control
QOL	Quality of Life
R&D	Research & Development
RCT	Randomized Control Trial
REC	Research Ethics Committee
SAE	Serious Adverse Event
SAR	Serious Adverse Reaction
SDV	Source Data Verification
SOP	Standard Operating Procedure
SSI	Site Specific Information
USAR	Unexpected Serious Adverse Reaction
TMG	Trial Management Group
TSC	Trial Steering Committee
TMF	Trial Master File

RESPONSIBILITIES

Sponsor: The nominated Sponsor for the study is The Newcastle upon Tyne Hospitals NHS Foundation Trust who will undertake a Research Governance Risk Assessment prior to commencement of the study.

Funder: The British Heart Foundation, Greater London House, 180 Hampstead Road, London, NW1 7AW

Trial Management: The study will be managed through the UKCRC registered Newcastle Clinical Trials Unit (Unit number 22). A Trial Management Group (TMG) will be responsible for overseeing the progress of the trial. The full list of TMG members is shown above.

Chief Investigator: The Chief Investigator will have overall responsibility for the conduct of the study.

Principal Investigator responsibility:

- Study conduct and the welfare of study subjects
- Compliance with the protocol, documentation of any protocol deviations and reporting of all serious adverse events
- Screening and recruitment of subjects
- Ensuring all trial-related medical decisions are made by a qualified physician, who is an investigator or co-investigator for the trial.
- Provision of adequate medical care in the event of an adverse event
- Obtaining local approval and abiding by the policies of Research Governance
- Compliance with the Principles of GCP, the Research Governance Framework for Health and Social Care, and the Data Protection Act
- Ensuring that no participant is recruited into the study until all relevant local regulatory permissions and approvals have been obtained.
- Obtaining written informed consent from participants prior to any study specific procedures
- The Principal Investigator shall be qualified by education, training and experience to assume responsibility for the proper conduct of the trial. S/he shall provide a current signed & dated curriculum vitae as evidence for the Trial Master File (TMF).
- The Principal Investigator shall ensure that site personnel are suitably trained for any task that they have delegated, and are named on the delegation log. A copy of the delegation log should be sent to the Trial Manager NCTU for inclusion in the TMF
- Ensuring Study Site team members are appropriately qualified by education, training and experience to undertake the conduct of the study.
- Availability for monitoring visits and in the case of an audit
- Maintaining study documentation and compliance with reporting requests
- Maintaining a site file, including copies of study approval, list of subjects and their signed informed consent forms
- Documenting appropriate delegation of tasks to study personnel e.g. Research Nurse, Investigator(s)
- Ensuring data collected is accurate, timely & complete
- Ensuring subject confidentiality is maintained during the project and archival period

- Ensuring archival of study documentation for a minimum of 15 years following the end of the study, unless local arrangements require a longer period

1. BACKGROUND and RATIONALE

Our population is ageing. Age is a powerful predictor of adverse events following acute myocardial infarction and percutaneous coronary intervention (PCI): adjusted odds for in-hospital death increase by 70% for each 10-year increase in age. Older patients are often frail with up to a half of older patients (≥75 years) admitted with non-ST segment elevation myocardial infarction (NSTEMI) being severely frail. These frail older patients have a 4-fold increased risk of death at 1-year and yet are often denied coronary revascularisation because of fear of complications and causing harm. The goals of this study are to determine the benefits and risks of invasive coronary angiography and coronary revascularisation in older patients presenting with NSTEMI and receiving optimal medical therapy.

Invasive Coronary Angiography and Coronary Revascularisation

A strategy of routine invasive coronary angiography with a view to coronary revascularisation reduces recurrent myocardial infarction (MI) and cardiovascular death among patients with NSTEMI (hazard ratio [HR]: 0.81, 95% confidence interval [CI]: 0.71-0.93; p=0.002).[1] In the British Heart Foundation RITA-3 trial (mean age 62 years),[2] a composite outcome of death, myocardial infarction (MI) and refractory angina was markedly reduced in the intervention arm compared with the conservative arm at 4 months (9.6% vs. 14.5%, p=0.001).

Acute Coronary Syndrome (ACS) in Older Patients

Although most developed countries have accepted the chronological age of 65 years as a definition of “elderly” or “older person”,[3] based on comprehensive analysis of data from a survey on public attitudes, surveys on older patients requiring nursing care, longitudinal studies of functional independence in the elderly and clinical and pathological data, it has been suggested to change the definition of elderly to those ≥75 years of age instead of the current 65 years.[4] We therefore refer to those aged ≥75 years as “older patients”.

In the UK, more than twice as many individuals ≥75 years of age (n=55,028) die from coronary heart disease (CHD) than younger individuals <75 years (n=25,540).[5] In the Global Registry of Acute Coronary Events (GRACE), increasing age was associated with increased incidence of NSTEMI.[6] Despite this, older patients were less likely to receive pharmacological therapies such as aspirin, statins, beta-blockers and angiotensin-converting enzyme inhibitors, and less likely to undergo invasive investigation and treatment such as angiography and PCI.[7, 8] Rates of major bleeding were more than twice as high in patients aged ≥85 years compared to <65 years (p<0.0001). Each 10-year increase in age resulted in 75% increase in in-hospital mortality.[9] Despite the fact that older patients constitute a significant proportion (30-40%) of the NSTEMI population, and have the greatest potential to benefit from intervention, older patients are under-represented and often excluded from clinical trials.[10, 11]

Older patients presenting with acute coronary syndrome (ACS) are at higher risk of poor outcomes even after adjustment for confounding factors, such as co-morbidities.[12] However, the rate of invasive angiography in patients with non-ST segment elevation ACS (NSTEACS) declines with age.[13] This has led to an apparent treatment paradox, whereby the highest risk patients are the least likely to undergo invasive management despite having the most potential to gain from it.[14]

Frailty in Patients with NSTEMI

Frailty is often cited as a reason not to undertake invasive coronary angiography. It can be evaluated using the Fried and Rockwood scores. Fried frailty score requires three or more of the following criteria to be present: unintentional weight loss (10 pounds in past year), self-reported exhaustion, weakness (grip strength), slow walking speed, and low physical activity.[15] Rockwood frailty score[16] consists of 7 categories: **(1) Very fit:** Robust, active, energetic, well-motivated and fit; these people commonly exercise regularly and are in the most fit group for their age. **(2) Well:** Without active disease, but less fit than people in category 1. **(3) Well**: With treated co-morbid disease - disease symptoms are well controlled compared with those in category 4. **(4) Apparently vulnerable**: Although not frankly dependent, these people commonly complain of being "slowed up" or have disease symptoms. **(5) Mildly frail:** With limited dependence on others for instrumental activities of daily living. **(6) Moderately frail**: Help is needed with both instrumental and non-instrumental activities of daily living. **(7) Severely frail**: Completely dependent on others for the activities of daily living, or terminally ill.

Approximately one half of all patients hospitalised with NSTEMI who are ≥75 years of age are frail by the Rockwood 7-point frailty criteria.[16] Frailty is independently associated with increased 1-year mortality (frail: 49% vs. non-frail: 13%) after adjusting for cardiovascular risk and co-morbid conditions in the setting of NSTEMI (HR 4.3; 95% CI 2.4-7.8).[17] However, fewer frail older patients undergo coronary angiography compared to non-frail patients. In the study by Ekerstad et al, only 15% of older frail patients underwent coronary angiography and only 7% underwent a PCI procedure.[18] On the other hand, frailty is also independently associated with the risk of major adverse cardiovascular outcomes or complications (composite of death from any cause, MI, revascularization due to ischemia, hospitalization for any cause, major bleeding, stroke/transient ischemic attack, and need for dialysis) at 30-days (odds ratio [OR] 2.2; 95% CI 1.3-3.7).[18] Given the fact that fewer frail patients underwent coronary angiography compared to non-frail patients in previous studies, the risk-benefit of invasive procedures in these high-risk frail older patients presenting with NSTEMI remains unclear.[18]

Coronary Revascularisation in Frail Older Patients

To date, there has been only one published modest sized randomized controlled trial to determine the benefits of invasive treatment specifically in older patients presenting with NSTEMI and unstable angina: the After 80 study.[19] This Norwegian study randomized 457 patients to invasive or conservative strategies and demonstrated a halving in the composite of myocardial infarction, need for urgent revascularisation, stroke, and death (41% vs. 61%, p=0.0001). This reduction in events was predominantly driven by fewer recurrent myocardial infarctions and urgent coronary revascularisations. There were no major differences in the rates of stroke, death, or minor or major bleeding. However, the study recruited ~10% of potential patients, did not formally assess frailty and was underpowered to assess individual clinical endpoints or subgroups who may benefit most. Importantly, there were also no assessments of subsequent quality of life, frailty or living independence.

Other previous studies have been non-randomized retrospective sub-group analyses or in the setting of chronic stable angina.[20] Two published studies have thus far evaluated the benefit of invasive strategy versus conservative treatment in older (≥75 years) patients with ACS without stratification for frailty status and recruiting selected patient cohorts with no significant co-morbidities. In the first registry study, 1005 patients underwent coronary

angiography and revascularisation, and 931 patients received conservative treatment. The mean age was 79 and 82 years (p<0.0001) respectively. In-hospital mortality and the combined endpoint of death or non-fatal re-infarction were lower in patients undergoing invasive management compared to those being managed conservatively (6.0% vs. 12.5%, p<0.0001 and 9.6% vs. 17.3%, p<0.0001 respectively). There was a marked reduction in one-year mortality in the invasive treatment group compared to conservative treatment group (OR 0.56; 95% CI 0.38-0.81).[21] The second study randomized 313 patients ≥75 years old with NSTEMI or unstable angina to an early invasive approach (n=154) or initial conservative approach (n=159).[22] In the early invasive treatment group, 88% of patients underwent coronary angiography and 55% had PCI, and in the initial conservative group it was 29% and 23% respectively. In patients with NSTEMI, there was a reduction in the primary end-point (composite of death, MI, stroke and rehospitalisation for CV causes) in the early invasive treatment group compared to conservative approach at 1 year (HR 0.43; 95% CI 0.23-0.80, Log rank p<0.05). There was no difference noted between the two groups with unstable angina.

Evidence from clinical trials to inform the management of ACS in older patients is limited. We have reviewed this topic in detail in previous publications.[19, 23] More than half of all trials for coronary disease in the past decade failed to enrol patients ≥75 years of age, with this subgroup accounting for just 9% of all patients enrolled in trials.[24] Evidence-based recommendations from trials do not account for the age-related differences in physiology and disease that may alter these relationships. The age gap between trial and community populations begins at age 75 years and widens with age.[10] Even the older patients included in trials are different from the older patients in the community. Trial populations have lower rates of traditional cardiovascular risk factors, less co-morbidity and better renal function in each age subgroup than do community populations.[25] As older patients are at increased risk from cardiac events, the absolute benefit of treatment should increase if treatment risks can be balanced against benefits.[26] Risks and benefits derived from trials cannot always be extrapolated to older patients in daily clinical practice due to the differences between the patient groups.[27] More specifically, previous studies did not stratify patients based on frailty status and have not evaluated the risk-benefit of revascularisation in this cohort. The only published previous randomized controlled trials of older patients[22, 28] did not account for frailty status, included patients without NSTEMI and had limited power to draw definitive conclusions. Moreover, the mortality and composite ischaemic endpoint rates were low in the Italian study suggesting inclusion of highly selected low-risk older patients who were not frail.

Whether the beneficial effects of revascularisation will be demonstrated in older patients with co-morbidities presenting with NSTEMI is unknown and will be investigated in the present study. This is particularly important as this potentially frail older cohort is becoming more prevalent. SENIOR-RITA specifically aims to investigate these high-risk co-morbid frail older patients who according to our national survey (responded to by 100 Cardiologists throughout the UK) are being denied advanced cardiovascular care due to fear of complications and perception of futility. We will utilise frailty score to identify, to characterise and to investigate at-risk older patients presenting with NSTEMI. We will also include all-comer older patients with NSTEMI including those with co-morbidities and those with cognitive impairment who normally would be denied invasive care due to underlying co-

morbidity and in whom there is lack of evidence currently in the management of NSTEMI. Previous studies have shown that older patients undergoing PCI experience significant improvements in health-rated QOL.[29, 30] We will assess whether any gains in quality of life represent best use of NHS resources by conducting an economic evaluation of coronary revascularisation compared with conservative management in this population.

2. OBJECTIVES AND OUTCOME MEASURES

2.1. Primary Objective

To determine the impact of a routine invasive strategy on one-year cardiovascular death and non-fatal myocardial infarction (MI) compared with a conservative treatment strategy in older patients (≥75 years) with NSTEMI.

2.2. Secondary Objectives

To determine the impact of a routine invasive strategy compared with a conservative strategy on:

- All-cause death
- Cardiovascular or non-cardiovascular death
- Recurrent myocardial infarction
- Urgent coronary revascularisation
- Recurrent hospitalisation for myocardial infarction
- Hospitalization for heart failure
- Stroke
- Bleeding (BARC ≥2)
- Procedural and in-hospital complications
- Length of time spent at home
- Frailty and quality of life
- Cost-effectiveness

2.3. Outcome Measures

In this randomized controlled trial, we will determine the risks and benefits of invasive versus conservative management on the following variables in older patients presenting with NSTEMI:

2.3.1. Primary Outcome Measure

Time to cardiovascular death or non-fatal MI (defined by the third universal definition[36] [see Section 16.1]) within 1 year from randomization

2.3.2. Secondary Outcome Measures (within 1 year and annually thereafter)

- All-cause, cardiovascular and non-cardiovascular death rates
- Recurrent myocardial infarction
- Hospitalisation for heart failure
- Urgent coronary revascularisation
- Recurrent hospitalisation for myocardial infarction
- Stroke
- Bleeding (BARC ≥2)
- Procedural complications (including death, MI, major bleeding (BARC definition), ≥25% increase in serum creatinine concentration from baseline, need for renal replacement therapy, stroke)

- Length of time spent at home
- Fried and Rockwood frailty scores
- Quality of Life using EQ-5D-5L and quality adjusted life years (QALY)
- Costs to the NHS and personal social services
- Incremental cost per QALY gained at 1 year

3. TRIAL DESIGN

SENIOR-RITA is a multicentre prospective open-label trial randomizing patients presenting with type 1 NSTEMI aged ≥75 years between invasive and conservative treatment strategies, to compare time to cardiovascular death or non-fatal MI within one year from randomization. The trial flow chart is shown in **Figure 1.**

Figure 1: Trial Flow diagram

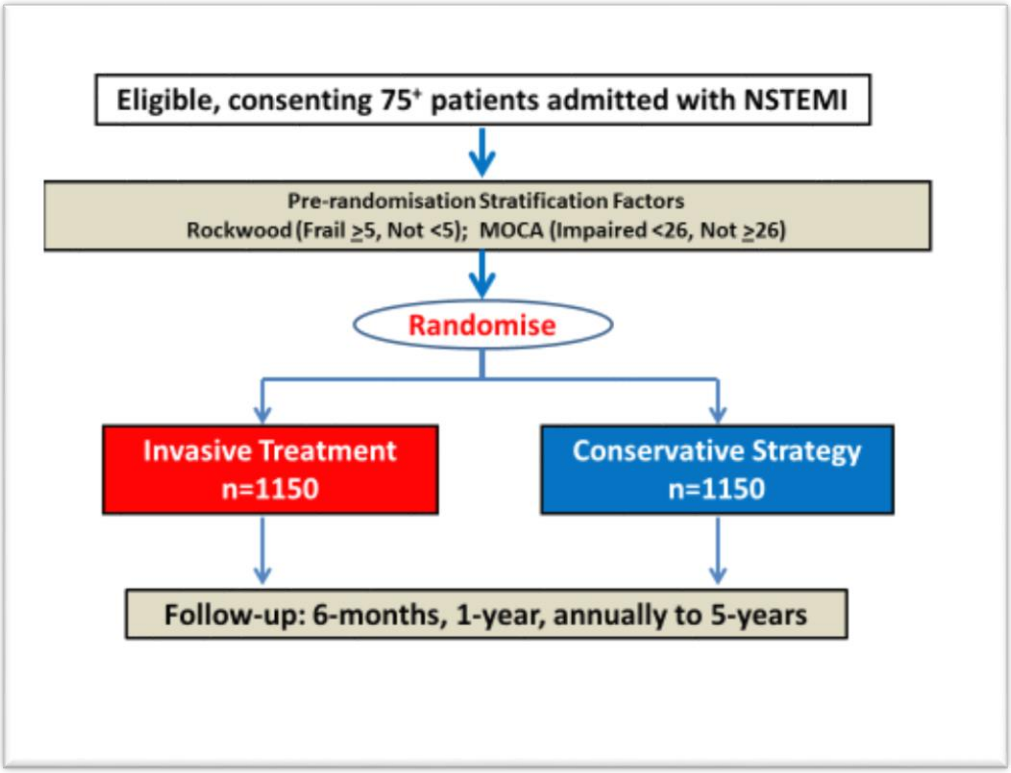

4. STUDY SETTING

This will be a broad and inclusive trial that will include participation of frail older patients with co-morbidities. Patients will be recruited from approximately 30 NHS centres throughout the UK. Patients will be recruited from centres with both on and offsite cardiac catheterisation facilities. Those patients randomized to invasive management in centres without cardiac catheterisation facilities will be transferred to a tertiary care centre within a recommended time frame of 7 days of randomization for angiography with a view to revascularisation. The study will be included as a UK Clinical Research Network study on the Central Portfolio Management System (CPMS) as a ‘Portfolio’ study. This will provide access to NIHR Clinical Research Network (CRN) service support and infrastructure in England. Inclusion on the Portfolio will also facilitate working with the Scottish Clinical Research Network and Health and Care Research Networks in Wales.

5. ELIGIBILITY CRITERIA

5.1. Inclusion Criteria

- Aged ≥75 years
- Type 1 NSTEMI during index hospitalisation

5.2. Exclusion Criteria

- Patients presenting with STEMI or unstable angina
- Patients with cardiogenic shock
- Patients with known life expectancy <1 year
- Patients in whom neither the patient nor the consultee are able and willing to provide written informed consent
- Previous inclusion in the BHF SENIOR-RITA trial
- Inability to undergo invasive coronary angiography, such as no vascular access site, or absolute contraindication to coronary revascularisation

6. TRIAL PROCEDURES

6.1. Patient Identification and Screening

Patients will be identified at tertiary centres and district hospitals based on eligibility criteria. Given the study will be on the NIHR portfolio, local research teams at individual sites that have agreed to participate in the study will screen for ≥75 year old patients with type 1 NSTEMI based on history (cardiac sounding chest pain), ECG changes (ST-T changes) and troponin elevation. Details of patients that are eligible but not randomized will be captured in the study using anonymised screening logs at all participating centres.

6.2. Consent

Patients with ability to provide informed consent: Patients will be provided with the SENIOR-RITA patient information sheet to read. Written informed consent will be obtained from patients with capacity. Patients who have capacity to consent but are unable to write will provide oral consent in the presence of an independent witness who will initial, sign and date the consent form on their behalf. Four copies of the consent form will be produced; one will be sent to NCTU for central monitoring of consent; one will be filed in the patient's medical record; one will be filed in the Investigator Site File; one will be given to the patient.

Patients lacking capacity to provide informed consent: Some patients with cognitive impairment will be able to provide informed consent. In patients who lack the capacity to provide informed consent, we will identify and approach a consultee as defined by the Mental Capacity Act (2005) England and Wales and the Adults with Incapacity (Scotland) Act 2000 and subsequent amendments. In England and Wales the consultee will be provided with the PIS and an information sheet for consultees and where appropriate, asked to sign the consultee declaration form for the patient's participation in SENIOR-RITA. In Scotland the nearest relative/guardian or Welfare Attorney will be provided with a participant information sheet and asked to consent on the participant's behalf which is permissible under the Adults with Incapacity (Scotland) Act 2000. Four copies of the form will be produced; one will be sent to NCTU for central monitoring of consent; one will be filed in the patient's medical record; one will be filed in the Investigator Site File; one will be given to the patient. Consent will include allowing the Newcastle Clinical Trials Unit to access patients' contact details in order to administer the EQ-5D-5L questionnaires.

6.3. Randomization

Following consent, patients will be randomized to an invasive strategy (coronary angiography and, if appropriate, coronary revascularisation [PCI or CABG] plus optimal medical therapy) or to conservative management (optimal medical therapy alone). Randomization will be on 1:1 per patient basis using a variable-length block stratified method derived by the statistics team and based on two stratification factors:

- Frailty score: frail (Rockwood Frailty Score ≥5) and not frail (Rockwood Frailty Score <5) (**Appendix 16.5**); and
- Cognitive impairment: impaired (Montreal Cognitive Assessment (MoCA) score <26) and not impaired (MoCA ≥26)

Randomization will be performed at site using a secure web-based system, to ensure concealment of allocation and minimise selection bias.

6.4. Data collection

All baseline data required for the study including baseline demographics, medical history/co-morbidities (Charlson Index), risk factors, current admission details, medications history will be collected during hospitalisation. Results of investigations performed as part of routine care of patients including full blood count, serum urea, creatinine concentrations, lipids, glucose and peak troponin concentration will be recorded. Data from baseline ECG will be collected for information on ST-T changes. Study related data including MoCA scores (baseline and 1-year), frailty scores, quality of life (EQ-5D-5L), use of health services and patient costs will also be collected. Echocardiographic data will be collected where available. Angiographic and procedural data will be collected from those that undergo invasive care. Detailed angiographic analysis will be carried out in Newcastle.

Frailty Scores: Rockwood and Fried frailty score will be calculated for all patients at baseline, 6 months, 1 year and yearly thereafter for 5 years.

Quality of Life: Quality of life (QOL) assessments using the EQ-5D-5L will be requested at baseline (pre-randomisation), 30 days, 3 months, 6 months, and at 1 year. Data will be collected during research clinic follow-up visits where feasible. Where this is not feasible, patients will be given the questionnaires to take home at the appropriate trial visits or questionnaires will be posted out to patients. Patients will be asked to complete the questionnaires at home after the trial visit and return the completed questionnaires back to the trial office. If questionnaires cannot be returned by post or are not returned after 2 weeks, data will be collected by telephone interviews with the patient or with their carer, where possible.

Given the increasing age of this population over the course of the trial, it is important to consider those who may struggle to self-complete quality of life instruments (e.g. due to pre-existing cognitive impairment or onset during the course of the trial). In addition, for the EQ-5D-5L, a proxy-rated version is available for relatives/carers to rate how they expect the patient views their current quality of life. We will strive to collect self-reported quality of life data where possible. It will not be possible at baseline to predict the patients who will go on to develop dementia during the course of the trial, and within this group, who will deteriorate to the extent that it will become no longer be possible to collect self-reported quality of life data. Thus, we aim to collect proxy data (EQ-5D-5L proxy version 2) for all participants at all time points in order to be consistent.

Health and personal social service resource use and patient costs: As part of our assessment of endpoints, we will quantify the cost and treatment in both trial arms by collecting data on the use of secondary, primary and personal social services (PSS) at baseline (and at each follow-up period) in a bespoke health and PSS utilisation questionnaire. These data will be combined with unit costs (obtained from routine sources or from study specific estimates) to calculate costs (see Section 10).

Consent will include access to patients' and relative/carer contact details to allow the Newcastle Clinical Trials Unit to centrally organise distribution of further postal questionnaire versions of the self-complete and proxy version 2 EQ-5D-5L to participants and their relative/carer at 30 days, and 3 months. Returned questionnaires will be used to provide additional data on how quality of life for patients over the course of the trial is characterised. It is pertinent to do this to account for possibly significant differences in quality of life for those in the intervention arm who undergo CABG as the revascularisation intervention.

6.5. Follow-up

Patients will be invited to attend research clinics for follow-up visits where feasible. Where this is not feasible, telephone follow-up will be carried out. If required, home visits by the research team will be organised in order to obtain follow-up data from patients. Follow-up information will be gathered for up to 10 years from ONS and GP databases; patient (or consultee) consent for this level of data access will be sought.

6.6. Schedule of events

	During Hospitalisation								
	V1	Postal	Postal	V2	V3	V4	V5	V6	V7
Event	In-patient with NSTEMI	30 days ±14 days	3-months ±14 days	6-month ±14 days	1 year ±14 days	2 year ± 28 days	3 year ±28 days	4 year ± 28 days	5 year ± 28 days
Eligibility	X								
Informed Consent	X								
Fried and Rockwood Frailty Score	X Required pre-randomisation			X	X	X	X	X	X
Montreal Cognitive Assessment	X Required pre-randomisation				X				
Randomize (Web)	X								
Patient Demographics, Baseline Admission Data	X								
Medical History, Risk Factors, Co-morbidities	X								
Baseline ECG	X								
Baseline Bloods (FBC, urea, creatinine, glucose, lipids*, peak troponin)	X								
Echocardiography*	X								
Angiography/Procedural Data	X								
Concomitant Medications	X			X	X				
Evaluate End-points Either Clinic or Post or Telephone or ONS, HES	X			X	X	X	X	X	X
EQ-5D-5L Patient and Proxy	X	X	X	X	X	X	X	X	X
NHS & PSS Utilisation†	X†			X†	X†	X‡	X‡	X‡	X‡
Time and Travel Questions				X					

† 6-month recall period; ‡ 12-month recall period; PSS- Personal social services; ONS- Office of National Statistics; Hospital Episode Statistics

*This information is collected where available from routine clinical practise

6.7 Withdrawal Criteria

Participants have the right to withdraw from the trial at any time without having to give a reason. Investigator sites should try to ascertain the reason for withdrawal and document this reason within the Case Report Form and participant's medical notes. Participants may:

- Withdraw from further study related procedures but continue to have contact from the study team for follow-up
- Have no further contact from the study team but allow continued use of routinely collected data
- Withdraw from any further involvement but allow use of all data up to the point of withdrawal of consent

All patients who withdraw from this trial will be requested to have follow-up data collected as per point 1

6.8 End of Trial

Completion of last patient last follow-up either by phone call or visit at 5-year follow-up. Patients will continue to receive care that is offered through the NHS for ongoing care of these patients following completion of the trial.
Collection of final follow-up information from ONS and GP databases will end at 10 years after enrolment in the study (patient consent for this level of data access will be obtained at baseline).

7. TRIAL INTERVENTIONS

7.1. Treatment Strategy

Invasive coronary angiography ± coronary revascularisation plus optimal medical therapy versus optimal medical therapy alone. Coronary angiography will be performed as per local practice patterns. Based on angiographic findings, revascularisation by PCI or CABG will be performed at the discretion of attending Cardiologist and the multidisciplinary team.

7.1.1. Schedule & Modifications

All invasive procedures will be performed according to local hospital protocols. It is recommended that patients undergo coronary angiography with a view to revascularisation (PCI/CABG) within 3-7 days after randomization and during their index hospitalisation. Where possible, coronary revascularisation should be completed within 7 days or as soon as practically possible.

7.1.2. Known Risks

Invasive coronary angiography, percutaneous coronary intervention and coronary artery bypass graft surgery are established treatment strategies for the management of coronary artery disease. The coronary angiography and coronary angioplasty procedures use x-rays, so there is a small risk from the exposure to this radiation. The amount of radiation involved is equivalent to less than three years of exposure to the average natural background radiation in the UK. Risks and benefits that are quoted during normal routine consent procedures are applicable to patients that will be recruited into the SENIOR-RITA trial. In addition, the procedure-related risk of in-hospital death (<1%), myocardial infarction (<1%), stroke (<1%), renal replacement therapy (<1%) and any bleeding (2%) will be quoted to the patients.

7.2. Concomitant Medications & Therapies

Optimal Contemporary Medical Therapy: In the absence of contraindications, all patients will receive aspirin 75 mg once daily; P2Y12 receptor antagonist in line with European Society of Cardiology guidelines; statin therapy, a beta-blocker (to target a heart rate of 60-70 beats per minute); an ACE inhibitor or ARB. If the patient continues to experience hypertension with blood pressure >140/85 mmHg, additional blood pressure lowering treatments will be considered at the discretion of the attending cardiologist and as tolerated by the patient. If the patient has marked left ventricular dysfunction (left ventricular ejection fraction <40%), an aldosterone antagonist will be considered. If the patient is diabetic, the goal will be to maintain fasting blood glucose levels between 4.4-7.5 mmol/L and haemoglobin A1c levels <7.0%, in accordance with published recommendations of the American Diabetes Association and the DCCT Consensus Report. In patients with poorly controlled glucose levels, insulin will be strongly considered.

8. SAFETY REPORTING

8.1. Definitions

Term	Definition
**Adverse Event (AE)**	Any untoward medical occurrence in a participant, including occurrences which are not necessarily caused by or related to the intervention under study. Medical conditions/diseases present before starting study treatment are only considered AEs if they worsen after starting study treatment.
**Adverse Reaction (AR)**	An untoward or unintended response in a participant to which is related to the intervention under study i.e. that a causal relationship between the trial intervention and an AE is at least a reasonable possibility and the relationship cannot be ruled out. All cases judged by either the reporting medically qualified professional or the Sponsor as having a reasonable suspected causal relationship to the trial intervention qualify as adverse reactions.
**Unexpected Adverse Reaction:**	An adverse reaction, the nature and severity of which is not consistent with the information about the intervention under study.
**Serious Adverse Event (SAE)**	A serious adverse event is any untoward medical occurrence that:<br>• Results in death<br>• Is life-threatening*<br>• Requires inpatient hospitalisation or prolongation of existing hospitalisation<br>• Results in persistent or significant disability/incapacity<br>• Consists of a congenital anomaly or birth defect<br>• Other important medical events that jeopardise the participant or require intervention to prevent one of the above consequences<br>*Life-threatening refers to an event in which the participant was at immediate risk of death at the time of the event; it does not refer to an event which hypothetically might have caused death if it were more severe.
**Serious Adverse Reaction (SAR)**	An adverse event that is both serious and, in the opinion of the reporting Investigator, believed with reasonable probability to be due to the trial intervention, based upon the information provided.
**Unexpected Serious Adverse Reaction (USAR)**	A serious adverse reaction, the nature and severity of which is not consistent with the known information about the intervention under study.

8.2. Severity (Intensity) of Adverse Events and Reactions

Severity of all AEs and ARs will be graded on a three-point scale of intensity (mild, moderate, severe):

- **Mild:** Discomfort is noticed, but there is no disruption of normal daily activities
- **Moderate:** Discomfort is sufficient to reduce or affect normal daily activities
- **Severe:** Discomfort is incapacitating, with inability to work or to perform normal daily activities

An AE or AR may be severe but not serious.

8.3. Assessment of Causality

Each AE should be clinically assessed for causality based on the information available, i.e., the relationship of the AE to the intervention under study should be established. The assignment of the causality will be made by the Principal Investigator responsible for the care of the participant using the definitions in the table below. All adverse events judged as having a reasonable suspected causal relationship to the intervention under study (i.e. definitely, probably or possibly related) are considered to be adverse reactions. If any doubt about the causality exists, the case will be reviewed and adjudicated by the clinical events committee (CEC). In the case of discrepant views on causality between the Principal Investigator and others, all parties will discuss the case and will refer as necessary to the TSC. In the event that no agreement is reached the main REC and other bodies will be informed of both points of view.

Relationship	Description
**Unrelated**	There is no evidence of any causal relationship. The clinical event has an incompatible time relationship to the intervention under study, and could be explained by underlying disease, or other drugs or chemicals.
**Unlikely**	There is little evidence to suggest there is a causal relationship (e.g. the event did not occur within a reasonable time after the intervention under study). There is another reasonable explanation for the event (e.g. the participant's clinical condition).
**Possible**	There is some evidence to suggest a causal relationship (e.g. the event occurs within a reasonable time after the intervention under study). However the influence of other factors may have contributed to the event (e.g. the participant's clinical condition).
**Probable**	There is evidence to suggest a causal relationship, including a reasonable time relationship with the intervention under study, and the influence of other factors is unlikely.
**Definitely**	There is clear evidence to suggest a causal relationship and other possible contributing factors can be ruled out.
**Not assessable**	There is insufficient or incomplete evidence to make a clinical judgement of the casual relationship.

8.4. Recording and Reporting AEs and SAEs

Research team staff at individual sites will complete the adverse events section of the relevant case report forms and input the details of the AEs into the MACRO database hosted by NCTU via the secure study website. The system will automatically alert the study NCTU Trial Manager if any new AEs are added or any amendments are made to

the data in existing ones. The trial management office in Newcastle in conjunction with the CI will have the responsibility for any decision making and forward reporting of AEs. Procedure related adverse events occurring within the first 7 days will be reported.

Serious Adverse Event (SAEs): All unexpected SAEs that are related to study participation shall be reported to NCTU through the study website within 24 hours of the site learning of its occurrence. The initial report can be made by secure fax which will also generate an email copy to the Chief Investigator, local Principal Investigator Senior Trial Manager and Trial Manager. In the case of incomplete information at the time of initial reporting, all appropriate information should be provided as follow-up as soon as this becomes available. Relationship of the SAE to trial participation should be assessed by the Principal Investigator at site, as should the expected or unexpected nature of the AE.
For each SAE the following information will be collected:

- Full details in medical terms and case description
- Event duration (start and end dates, if applicable)
- Action taken
- Outcome
- Seriousness criteria
- Causality in the opinion of the investigator
- Whether the event is considered expected or unexpected

The SAE form should be transmitted by fax to the NCTU on 0191 5800866 (SoHo 66).

For the purposes of this protocol

- SAEs exclude any pre-planned hospitalisations not associated with clinical deterioration.
- SAEs exclude routine treatment or monitoring of the studied indication, not associated with any deterioration in condition.
- Cardiovascular death and other events that are primary or secondary outcome measures are not considered to be SAEs and should be reported in the normal way on the appropriate CRF.

The main RECs will be notified by NCTU (on behalf of the Sponsor) of all USARs within 15 days of NCTU becoming aware of the USAR. USARs will be reported using the NRES Report of Serious Events Form, version 3, April 2007. The NCTU will ensure that The Newcastle upon Tyne Hospitals NHS Foundation Trust as Sponsor is notified of any USARs in accordance with local trust policy. Local principal investigators should report any USARs as required by their local Research & Development Office.

8.5. Responsibilities

Principal Investigator

- Checking for AEs when participants attend for treatment or follow-up
- Using medical judgement in assigning seriousness and causality and providing an opinion on expectedness of events

- Ensuring that all SAEs and SARs, including USARs, are recorded and reported to the Sponsor within 24 hours of becoming aware of the event and provide further follow-up information as soon as available
- Ensuring that AEs and ARs are recorded and reported to the Sponsor in line with the requirements of the protocol

Chief Investigator

- Clinical oversight of the safety of trial participants, including an ongoing review of the risk/benefit profile
- Using medical judgement in assigning seriousness, causality and expectedness of SAEs where it has not been possible to obtain local medical assessment.
- Using medical judgement in assigning expectedness to SARs.
- Immediate review of all USARs
- Review of specific SAEs and SARs in accordance with the trial risk assessment and protocol

Sponsor

- Assessment of expectedness of any USARs
- Expedited reporting of USARs to the REC within required timelines
- Notification of all investigator sites of any USAR that occurs

8.6. Notification of Deaths

Procedure related deaths will be notified to the sponsor using the study SAE form.

8.7. Reporting Urgent Safety Measures

An Urgent Safety Measure (USM) is an action that the Sponsor or an Investigator may take in order to protect the subjects of a trial against any immediate hazard to their health or safety. Upon implementation of an USM by an Investigator, the Sponsor must be notified immediately and details of the USM given. The Sponsor must inform the NHS REC within 3 days of the USM taking place in accordance with the Sponsor's standard operating procedures.

9. STATISTICAL CONSIDERATIONS

9.1. Analysis Populations

All data from trial participants will be included in the primary analysis conducted on an intention to treat (ITT) basis. Sensitivity analyses may be conducted on a per-protocol subset. Intervention related AEs and SAEs will be reported for the ITT and per-protocol sets. All statistical analyses will follow a fully detailed predefined statistical analysis plan, written prior to any comparative interim analysis (i.e. for DMC reporting).

9.2. Statistical Analyses

9.2.1. Analyses of the Primary Outcome Measure

The primary outcome is time to cardiovascular death or non-fatal MI as time to first event of a composite outcome at 1-year follow-up from randomization. Alive patients will be censored in the analysis at the time last seen alive MI free or time of death (for non-CV deaths). Event-free rates will be estimated using the method of Kaplan and Meier, and reported as 6-month and 1-year CV death and non-fatal MI composite rates, with the associated hazard ratio (HR) comparing risk of invasive treatment with conservative therapy (with 95% confidence intervals). Event-free estimates will be statistically compared using log-rank analyses and stratified log-rank analyses (based on stratification factors of levels of frailty and cognitive impairment groups at randomization). Multivariable regression will investigate and present the treatment effect adjusted by baseline stratification factors (levels of frailty and cognitive impairment at randomization) analysed using Cox proportional regression or parametric regression modelling, depending on validation of statistical assumptions. Further multivariable models will investigate other demographic (e.g. sex, age) and clinical covariates including baseline disease severity, co-morbidities and cardiovascular risk factors. Competing risks analyses will be carried out and reported based on time to event analysis of each event type of the composite (CV death and non-fatal MI) given patients may experience both events of interest and acknowledging a death event prevents further outcome events of interest.

9.2.2. Analyses of Secondary Outcome Measures

Time to Event: Analyses of secondary time to event outcomes will be analysed and reported using similar Kaplan Meier time to event analyses based on all-cause, cardiovascular and non-cardiovascular deaths. Unadjusted and adjusted HR of the risk of invasive treatment compared with conservative therapy (with 95% confidence intervals) will be reported.

Numbers and rates of recurrent myocardial infarction, hospitalisation and recurrent hospitalisation (for heart failure), coronary revascularisation and length of time spent at home will be reported descriptively.

Safety data: Including the numbers and rates of procedural complications (death, MI), major bleeding including gastrointestinal and intracranial bleeding [BARC definition], ≥25% increase in creatinine from baseline, need for renal replacement therapy, incidence of stroke) will be reported descriptively summarising continuous measures

as means and proportions of the total number randomized (with 95% confidence intervals). Missing data are expected to be minimal and will be reported.

Quality of Life: QoL according to EQ-5D-5L will be graphically summarised over the 12-month period by randomized treatment group. The dimensions of the EQ-5D-5L will be investigated. Questionnaire responses are transformed into utility scores according to the developers' instructions and these will be presented longitudinally. Standardised area under the curve summary statistics will be reported as a conditional analysis on survival within 12 months and will be compared across treatment groups. EQ-5D utility score (see Section 10 for scoring of EQ-5D-5L) will be used in a quality-adjusted survival analysis to analyse QoL and survival simultaneously. Primary QoL analysis will be based on patient reported scores. Comparison of proxy reported and patient reported scores will be undertaken.

Frailty Scores:
Scores will be calculated according to the developers' instructions and will be graphically summarised over time by randomized treatment group. Standardised area under the curve summary statistics will be used to report frailty scores over time conditional on survival and will be compared across treatment groups. The association between Rockwood and Fried frailty scores will be investigated using rank correlation.

9.2.3. Subgroup Analyses

Planned subgroup analysis will present hazard ratios for individual subgroups using Forest plots. Pre-planned sub-group analysis (according to a predefined statistical analysis plan) will be based on the following sub-groups:

- Frail vs. not frail patients (Rockwood score ≥5 vs. <5).
- Cognitively impaired vs. not impaired (Montreal Cognitive Assessment (MoCA) score <26 vs. ≥26).
- Frail and cognitively impaired patients vs. non-frail, non-cognitively impaired patients
- Co-morbid vs. non co-morbid patients

Hazard Ratios of the treatment effect will be calculated within identified subgroups and plotted on a Forest plot with associated 95% confidence intervals. Tests of heterogeneity of the difference in the estimated treatments effects across subgroups will be calculated and reported.

9.2.4. Planned Additional Analyses

Time to event analyses will be reported descriptively using unadjusted and adjusted HR (95% CI) for longer term follow up of patients to 5 and 10 years.

9.2.5. Interim Analyses and Criteria for the Premature Termination of the Trial

The trial will be monitored by an external Data Monitoring Committee (DMC) that will meet at the start of the trial and annually thereafter. Interim meetings may be convened should the DMC have any safety concerns. Accumulating patient data will be reported to the DMC but interim analyses will not be undertaken until 50% of patients are recruited. The DMC will make recommendations to the Trial Steering Committee as to whether to stop or continue recruitment based on all available

evidence and with attention to the Peto-Haybittle boundary when interpreting significance levels, as specified in the statistical analysis plan and DMC charter.

9.3. Sample Size Calculations

The recruitment target for this trial is a total of 2300 patients (1150 in each arm).

In previous studies, the all-cause death rates in frail and non-frail patients presenting with NSTEMI was approximately 30% at one year,[17] which equates to an assumed 12-month 'event-free survival' rate on the conservative therapy arm of 70% at 12-months. In our trial, the estimated reduction in event rate due to including only CV deaths is anticipated to be counter balanced by an increase in event rate due to the inclusion of non-fatal MI. A clinically relevant effect size is assumed to be a 20% reduction in the overall event rate. To detect a clinically relevant reduction in cardiovascular death and MI from 30% to 24% equates to a clinically relevant increase in event free survival from 70% to 76% and a Hazard Ratio=0.77. To detect this increase in event free survival rate at 12 months, requires a minimum of 620 events to be observed. Therefore these events will be observed for at least 12 months and if 620 events has not been reached at 12 months this will be extended. It is estimated that this trial will need to recruit 1149 patients to each randomised arm to observe this number of events. With 2300 patients and 620 events, this trial will be able to reject the null hypothesis that the invasive treatment and conservative therapy survival estimates are equal with probability (power) of 90%, and associated type I error (false positive) probability (alpha) of 5%[31]. The analysis of the primary outcome measure will be conducted when the number of events has been reached.

If the true size of difference is smaller, similar to that found by Fox et al,[1] then this trial would have 78% power to detect the HR=0.81 reported. MINAP database reported an incremental reduction from intensive management with increasing age.[32] The adjusted HR for all-cause mortality comparing invasive to conservative management was HR=0.53 (reported inversely as HR=1.90 95% CI 1.77, 2.04) in NSTEMI patients aged >85 years. If the true size of difference is larger, similar to that found by Zaman et al,[32] then this trial would have >99% power to detect a larger difference of HR=0.53.

Underlying assumptions pertaining to the sample size calculations, including overall numbers of CV deaths and non-fatal MI, will be monitored by the independent DMC.

10. ECONOMIC EVALUATION

10.1. Assessment of Cost

For the main analysis we will conduct a micro-costing exercise to estimate the cost of the interventions (costed on a per-patient basis), as well as the use of secondary, primary and personal social services (PSS) during the follow up period. This is because the perspective for the main analysis will be the NHS and PSS. However, we will conduct further analyses that take a societal perspective by incorporating the time and travel costs and out of pocket expenses for health care borne by patients and their families. Details of resources used and patient costs will be obtained from bespoke patient completed questionnaires. For resources used, this will be completed at each face to face data collection time period, using a modified version of the Client Service Receipt Inventory (CSRI). The CSRI is a validated instrument designed to be adapted to the specific study population of interest[33, 34]. Patient time and travel costs will be collected at one time point (six months). Unit costs will be based on nationally available data from routine sources and study-specific estimates. Where appropriate, discounting will be applied to costs and outcomes.

10.2. Economic Analysis

A cost-utility analysis will use the responses to the within-trial assessment of quality of life using the EQ-5D-5L, combined with each study participant's mortality to estimate QALYs. This measure provides a profile of quality of life over time. The results of the analyses will be presented as point estimates of mean incremental costs and QALYs. We will undertake sensitivity analyses to test key assumptions made during data collection, including for example, the accuracy of any proxy-reported EQ-5D-5L values. Techniques such as bootstrapping will be used alongside deterministic sensitivity analyses to address uncertainty, and will be presented as cost-effectiveness acceptability curves (CEACs) for each trial arm, for different levels of society's willingness-to-pay for a QALY. An economic model will then be developed to assess the cost and health consequences measured in terms of QALYs over the patients remaining lifetime. This will allow us to consider the longer-term effects of the treatments received. We will develop a range of plausible care pathways, using the literature and expert advice on the natural course of this illness for this population, along with expert advice on typical care routes. The level of detail involved in modelling care routes will to some extent depend on the extent of variation in the kinds of care being received. The data from the trial will be the main source of data for this model but further data with which to model outcomes beyond the follow-up will be systematically derived from the literature and other existing data sources following guidance for best practice.[35] These data will include information on factors such as the incidence of hospitalisation beyond the trial follow-up period. Sensitivity analysis will be applied to the model using probabilistic and deterministic sensitivity analyses to address parameter and other forms of uncertainty.

We will identify from existing and possibly ongoing studies health related quality of life as measured by EQ-5D-5L instruments that have been collected over the course of one year following revascularisation interventions. If sufficient applicable data are

available from the existing evidence base, we will seek to obtain the individual level data and information of the characteristics of the individuals revascularised and explore whether such data could be used to impute EQ-5D responses during the recovery period following revascularisation. If this is possible and can be completed in a timely manner we will consider reducing the response burden for the EQ-5D-5L on participants in this trial.

11. DATA HANDLING

11.1. Data Collection Tools and Source Document Identification

All data for an individual patient will be collected by the PI or their delegated nominees and recorded in the web-based electronic case report form (CRF) for the study delivered via Elsevier's MACRO. All investigators and study teams will be provided with secure logins to access the ECRF to input patient data. Patient identification on the CRF will be through a unique study identifier number allocated by a web based randomization system. The NCTU will audit completeness and quality of data recording in CRFs and will correspond regularly with the PI (or their delegated assistants) with the aim of capturing any missing data where possible, and ensuring continuous high quality of data.

11.2. Data Handling and Record Keeping

All data will be stored in password-protected databases. Any paper documents will be stored in study related locked filing cabinets. Clinical information will not be released without the written permission of the participant, except as necessary for monitoring and auditing by the Sponsor, its designee, Regulatory Authorities, the Trial Steering Committee (TSC), the Data Monitoring Committee (DMC) or the REC. Secure anonymized electronic data will be released to the Trial Statisticians for interim and final analyses. The PI and study site staff involved with this study may not disclose or use for any purpose other than performance of the study, any data, record, or other unpublished, confidential information disclosed to those individuals for the purpose of the study. Prior written agreement from the Sponsor or its designee must be obtained for the disclosure of any said confidential information to other parties. Data will be anonymised by coding in order to maintain confidentiality of the study participant but the research team will be able to identify participants in the event of clinical need. Any data recorded electronically will be onto a password protected NHS computer in the research centre. Data received at NCTU will be processed as per the NCTU SOPs, including entering the data into a secure central database. Responsibility for maintenance of the database will rest with the NCTU. The NCTU SOPs utilized in this protocol are listed at the following web page:
http://www.ncl.ac.uk/nctu/activities/sop/library.htm

11.3. Access to Data

The Investigators will have access to their own patient data during the trial. Accumulating trial data sets will be accessible by Chief Investigator and NCTU.

11.4 Archiving

The site will construct an Investigator Site File (ISF) and will maintain all study records according to GCP and the applicable regulatory requirements. The trial master file (TMF) will be held by the NCTU and the essential documents that make up the file will be listed in a SOP. On completion of the trial, the TMF and study data will be archived by the Sponsor according to the applicable regulatory requirements and for up to 15 years.

12. MONITORING, AUDIT & INSPECTION

The trial may be subject to audit by representatives of the Sponsor or inspection by HRA. Each investigator site will permit trial-related monitoring, audits and regulatory inspection including access to essential and source data relating to the trial. The trial may be prematurely discontinued on the basis of new safety information, or for other reasons given by the Data Monitoring Committee (DMC) and/or Trial Management Group (TMG), Sponsor, regulatory authority or ethics committee concerned.

Monitoring, quality control and assurance: The trial will be managed by NCTU in collaboration with the CI and Co-PIs.

The Trial Management Group (TMG): The Principal Investigators will be responsible for the day-to-day study conduct at site. The NCTU trial management team will provide day-to-day support for the sites and will provide training through Investigator meetings, site initiation visit and routine monitoring visits. Quality control will be maintained through adherence to NCTU SOPs, study protocol, the principles of GCP, the Research Governance Framework for Health and Social Care, the European Directive (2001/20/EC), the Medicines for Human Use (Clinical Trials) Regulations 2004 (SI 1031) and all subsequent amendments thereof, and the Declaration of Helsinki (1996).

Trial Steering Committee (TSC): TSC will be convened to undertake independent review. The committee will meet at least once a year. The role of the TSC is to provide the overall supervision of the trial and to ensure that the trial is conducted to the rigorous standards set out in the Department of Health's Research Governance Framework for Health and Social Care and Guidelines for Good Clinical Practice. TSC members consist of individuals who are independent of the investigators, their employing organisations, and sponsors. The TSC will monitor trial progress and conduct and advise on scientific credibility. The TSC will consider and act, as appropriate, upon the recommendations of the Data Monitoring Committee (DMC).

Data Monitoring Committee (DMC): The DMC will assess at annual intervals at least, the progress of the clinical trial, the safety data, and the critical efficacy endpoints, and will recommend to the sponsor whether to continue, modify, or stop the trial. If the study is prematurely discontinued, participants enrolled to date will be informed and no further participants will be recruited. For participants already enrolled data may still continue to be collected.

Clinical Events Adjudication Committee (CEC): The CEC will adjudicate important clinical events including those included in the primary outcome measure events.

Trial monitoring: Monitoring of study conduct and data collected will be performed by a combination of central review and site monitoring visits to ensure the study is conducted in accordance with GCP. Study site monitoring will be undertaken by NCTU, following a risk-based assessment approved by sponsor. The main areas of focus will include consent, serious adverse events, and review of essential documents in study files.

Site monitoring will include: Original consent forms will be reviewed as part of the study file. The presence of a copy in the patient hospital notes will be confirmed for participants. Reported serious adverse events will be verified against treatment notes/medical records (source data verification). The presence of essential documents in the investigator site file and study files will be checked. Source data verification of primary endpoint data and eligibility data for participants entered in the study will be performed.

Central monitoring will include: All applications for study authorisations and submissions of progress/safety reports will be reviewed for accuracy and completeness, prior to submission. All documentation essential for study initiation will be reviewed prior to site authorisation. All monitoring findings will be reported and followed up with the appropriate persons in a timely manner. The study may be subject to inspection and audit by NUTH under their remit as sponsor, and other regulatory bodies to ensure adherence to GCP. The investigator(s) / institutions will permit trial-related monitoring, audits, REC review and regulatory inspection(s), providing direct access to source data/documents.

Serious Breaches: It is the responsibility of the Chief Investigator (CI) and Co-Investigators to ensure that the clinical trial is run in accordance with GCP and the protocol. Given this is a multicentre trial, this task is delegated to the local Principal Investigators at each site. Any actual or suspected breaches must be reported to the Research Governance Manager (RGM) and/or Quality Assurance Manager (QAM) within 24 hours of identification. The breach will also need to be reported to the REC. Deviations from the protocol and GCP occur in clinical trials and the majority of these events are technical deviations that are not serious breaches. These events should be documented in the Case Report Form (CRF) or by the completion of a File Note, in order for Corrective and Preventative Actions (CAPA) to be taken.

Discontinuation rules: The trial may be prematurely discontinued on the basis of new safety information, or for other reasons given by the Trial Steering Committee (TSC), Data Monitoring Committee (DMC) and/or Sponsor, or ethics committee concerned.

13. ETHICAL AND REGULATORY CONSIDERATIONS

13.1. Research Ethics Committee Review and Reports

The NCTU will obtain a favourable ethical opinion from an NHS Research Ethics Committee (REC) in England and Scotland prior to the start of the trial. All parties will conduct the trial in accordance with this ethical opinion. The NCTU will notify the REC of all required substantial amendments to the trial and those non-substantial amendments that result in a change to trial documentation (e.g. protocol or patient information sheet). Substantial amendments that require a REC favourable opinion will not be implemented until this REC favourable opinion is obtained. The NCTU will notify the REC of any serious breaches of GCP or the protocol, urgent safety measures or USARs that occur during the trial. An annual progress report will be submitted each year to the REC by the NCTU until the end of the trial. This report will be submitted within 30 days of the anniversary date on which the original favourable ethical opinion was granted. The NCTU will notify the REC of the early termination or end of trial in accordance with the required timelines.

13.2. Public and Patient Involvement

We have included patient group involvement in SENIOR-RITA through VOICENorth (http://www.ncl.ac.uk/ageing/innovation/engagement/voicenorth/). VOICENorth is linked in to our Newcastle NIHR Biomedical Research Centre in ageing and chronic disease and Faculty of Medical Sciences, Newcastle University. The lay summary of the SENIOR-RITA protocol was presented to the VOICENorth Research Support Group. One member of VOICENorth has been invited to participate as a Trial Steering Committee lay member. Once the study is completed, the patient group will also support with the dissemination of study findings to the lay public and patients.

13.3. Regulatory Compliance

The trial will be conducted in accordance with the Health Research Authority guidance. Before any site can enrol patients into the trial, that site must have received NHS permission from the site's Research & Development department.

13.4. Protocol Compliance

All investigators and study teams must adhere to the protocol, in particular the inclusion and exclusion criteria of the study. Waivers of the eligibility criteria are not permitted. Any deviation from the protocol must be notified to the NCTU trial manager for assessment and onward reporting to the sponsor if required.

13.5. Notification of Serious Breaches to GCP and/or the Protocol

A serious breach is a breach which is likely to affect (to a significant degree) the safety or physical or mental integrity of the subjects of the trial; or the scientific value of the trial. The sponsor must be notified immediately of any incident that may be classified

as a serious breach. The NCTU will notify the NHS REC within the required timelines in accordance with the NCTU SOP.

13.6. Data Protection and Patient Confidentiality

All personal data will be regarded as strictly confidential. The study will comply with the Data Protection Act, 1998. All study records and Investigator Site Files will be kept at site in a locked filing cabinet with restricted access.

13.7. Indemnity

The Newcastle upon Tyne Hospitals NHS Foundations Trust is Sponsor and through the Sponsor, NHS indemnity is provided in respect of potential liability and negligent harm arising from study management. Indemnity in respect of potential liability arising from negligent harm related to study design is provided by the substantive employers of protocol authors (HEIs/NHS). All study sites are NHS organisations and indemnity in respect of potential liability arising from negligent harm related to study conduct at individual sites will be provided via NHS schemes.

13.8. Amendments

It is the responsibility of the Research Sponsor to determine if an amendment is substantial or not and study procedures must not be changed without the mutual agreement of the Chief Investigator, Co-Investigators, Sponsor, Trial Management Group and Trial Steering Committee. Substantial amendments will be submitted to the REC and will not be implemented until this approval is in place. It is the responsibility of the NCTU to submit substantial amendments. Non-substantial amendments may be made at any time with a record of the amendment held in the Trial Master File. Any non-substantial amendment that requires an update to the trial documentation will be submitted to the NHS REC for acknowledgement of the revised version of the document. Substantial amendments and those minor amendments which may impact sites will be submitted to the relevant NHS R&D Departments for notification to determine if the amendment affects the NHS permission for that site. Amendment documentation will be provided to sites by the NCTU.

14. DISSEMINATION POLICY

The data will be the property of the Chief Investigator and Co-Investigators. Publication will be the responsibility of the Chief Investigator and the Trial Management Group and published under the authorship agreed with all the Co-Investigators who fulfil the ICMJE criteria for authorship. It is planned to publish this trial in peer-reviewed journals and to present data at national and international meetings. Results of the study will also be reported to the Sponsor and Funder. Participants will be informed about their treatment and their contribution to the study at the end of the trial, including a lay summary of the results.

15. REFERENCES

1. Fox KA, Clayton TC, Damman P, Pocock SJ, de Winter RJ, Tijssen JG, Lagerqvist B, Wallentin L. Long-term outcome of a routine versus selective invasive strategy in patients with non-st-segment elevation acute coronary syndrome a meta-analysis of individual patient data. *J Am Coll Cardiol*. 2010;55:2435-2445
2. Fox KA, Poole-Wilson PA, Henderson RA, Clayton TC, Chamberlain DA, Shaw TR, Wheatley DJ, Pocock SJ. Interventional versus conservative treatment for patients with unstable angina or non-st-elevation myocardial infarction: The british heart foundation rita 3 randomised trial. Randomized intervention trial of unstable angina. *Lancet*. 2002;360:743-751
3. Organisation WH. Http://www.Who.Int/healthinfo/survey/ageingdefnolder/en/.
4. Orimo H, Ito H, Suzuki T, Araki A, Hosoi T, Sawabe M. Reviewing the definition of "elderly". *Geriatrics & Gerontology International*. 2006;6:149-158
5. Townsend N, Wickramasinghe K, Bhatnagar P, Smolina K, Nichols M, Leal J, Luengo-Fernandez R, Rayner M. Coronary heart disease statistics 2012 edition. 2012
6. Goodman SG, Huang W, Yan AT, Budaj A, Kennelly BM, Gore JM, Fox KA, Goldberg RJ, Anderson FA, Jr., Expanded Global Registry of Acute Coronary Events I. The expanded global registry of acute coronary events: Baseline characteristics, management practices, and hospital outcomes of patients with acute coronary syndromes. *American Heart Journal*. 2009;158:193-201 e191-195
7. Devlin G, Gore JM, Elliott J, Wijesinghe N, Eagle KA, Avezum A, Huang W, Brieger D, Investigators G. Management and 6-month outcomes in elderly and very elderly patients with high-risk non-st-elevation acute coronary syndromes: The global registry of acute coronary events. *European Heart Journal*. 2008;29:1275-1282
8. Nguyen HL, Goldberg RJ, Gore JM, Fox KA, Eagle KA, Gurfinkel EP, Spencer FA, Reed G, Quill A, Anderson FA, Jr. Age and sex differences, and changing trends, in the use of evidence-based therapies in acute coronary syndromes: Perspectives from a multinational registry. *Coronary Artery Disease*. 2010;21:336-344
9. Avezum A, Makdisse M, Spencer F, Gore JM, Fox KA, Montalescot G, Eagle KA, White K, Mehta RH, Knobel E, Collet JP, Investigators G. Impact of age on management and outcome of acute coronary syndrome: Observations from the global registry of acute coronary events (grace). *American Heart Journal*. 2005;149:67-73
10. Alexander KP, Newby LK, Cannon CP, Armstrong PW, Gibler WB, Rich MW, Van de Werf F, White HD, Weaver WD, Naylor MD, Gore JM, Krumholz HM, Ohman EM. Acute coronary care in the elderly, part i: Non-st-segment-elevation acute coronary syndromes: A scientific statement for healthcare professionals from the american heart association council on clinical cardiology: In collaboration with the society of geriatric cardiology. *Circulation*. 2007;115:2549-2569
11. Hordijk-Trion M, Lenzen M, Wijns W, de Jaegere P, Simoons ML, Scholte op Reimer WJ, Bertrand ME, Mercado N, Boersma E. Patients enrolled in coronary intervention trials are not representative of patients in clinical practice: Results from the euro heart survey on coronary revascularization. *European Heart Journal*. 2006;27:671-678
12. Granger CB, Goldberg RJ, Dabbous O, Pieper KS, Eagle KA, Cannon CP, Van De Werf F, Avezum A, Goodman SG, Flather MD, Fox KA, Global Registry of Acute Coronary Events I. Predictors of hospital mortality in the global registry of acute coronary events. *Archives of Internal Medicine*. 2003;163:2345-2353
13. Bagnall AJ, Goodman SG, Fox KA, Yan RT, Gore JM, Cheema AN, Huynh T, Chauret D, Fitchett DH, Langer A, Yan AT, Canadian Acute Coronary Syndrome Registry I, Investigators II, Canadian Global Registry of Acute Coronary Events I. Influence of age on use of cardiac catheterization

and associated outcomes in patients with non-st-elevation acute coronary syndromes. *The American Journal of Cardiology*. 2009;103:1530-1536
14. Fox KAA, Anderson FA, Dabbous OH, Steg PG, López-Sendón J, Van de Werf F, Budaj A, Gurfinkel EP, Goodman SG, Brieger D. Intervention in acute coronary syndromes: Do patients undergo intervention on the basis of their risk characteristics? The global registry of acute coronary events (grace). *Heart*. 2007;93:177-182
15. Fried LP, Tangen CM, Walston J, Newman AB, Hirsch C, Gottdiener J, Seeman T, Tracy R, Kop WJ, Burke G, McBurnie MA. Frailty in older adults: Evidence for a phenotype. *J Gerontol A Biol Sci Med Sci*. 2001;56:M146-156
16. Rockwood K, Song X, MacKnight C, Bergman H, Hogan DB, McDowell I, Mitnitski A. A global clinical measure of fitness and frailty in elderly people. *CMAJ*. 2005;173:489-495
17. Ekerstad N, Swahn E, Janzon M, Alfredsson J, Lofmark R, Lindenberger M, Andersson D, Carlsson P. Frailty is independently associated with 1-year mortality for elderly patients with non-st-segment elevation myocardial infarction. *Eur J Prev Cardiol*. 2014;21:1216-1224
18. Ekerstad N, Swahn E, Janzon M, Alfredsson J, Lofmark R, Lindenberger M, Carlsson P. Frailty is independently associated with short-term outcomes for elderly patients with non-st-segment elevation myocardial infarction. *Circulation*. 2011;124:2397-2404
19. Veerasamy M, Edwards R, Ford G, Kirkwood T, Newton J, Jones D, Kunadian V. Acute coronary syndrome among older patients: A review. *Cardiology in Review*. 2014;23:26-32
20. Pfisterer M, Buser P, Osswald S, Allemann U, Amann W, Angehrn W, Eeckhout E, Erne P, Estlinbaum W, Kuster G, Moccetti T, Naegeli B, Rickenbacher P. Outcome of elderly patients with chronic symptomatic coronary artery disease with an invasive vs optimized medical treatment strategy: One-year results of the randomized time trial. *JAMA : the Journal of the American Medical Association*. 2003;289:1117-1123
21. Bauer T, Koeth O, Junger C, Heer T, Wienbergen H, Gitt A, Zahn R, Senges J, Zeymer U. Effect of an invasive strategy on in-hospital outcome in elderly patients with non-st-elevation myocardial infarction. *European Heart Journal*. 2007;28:2873-2878
22. Savonitto S, Cavallini C, Petronio AS, Murena E, Antonicelli R, Sacco A, Steffenino G, Bonechi F, Mossuti E, Manari A, Tolaro S, Toso A, Daniotti A, Piscione F, Morici N, Cesana BM, Jori MC, De Servi S, Italian Elderly ACSTI. Early aggressive versus initially conservative treatment in elderly patients with non-st-segment elevation acute coronary syndrome: A randomized controlled trial. *JACC. Cardiovascular Interventions*. 2012;5:906-916
23. Sinclair H, Kunadian V. Coronary revascularisation in older patients with non-st elevation acute coronary syndromes. *Heart*. 2016; 102(6):416-24.
24. Lee PY, Alexander KP, Hammill BG, Pasquali SK, Peterson ED. Representation of elderly persons and women in published randomized trials of acute coronary syndromes. *JAMA : the Journal of the American Medical Association*. 2001;286:708-713
25. Kandzari DE, Roe MT, Chen AY, Lytle BL, Pollack CV, Jr., Harrington RA, Ohman EM, Gibler WB, Peterson ED. Influence of clinical trial enrollment on the quality of care and outcomes for patients with non-st-segment elevation acute coronary syndromes. *American Heart Journal*. 2005;149:474-481
26. Alter DA, Manuel DG, Gunraj N, Anderson G, Naylor CD, Laupacis A. Age, risk-benefit trade-offs, and the projected effects of evidence-based therapies. *The American Journal of Medicine*. 2004;116:540-545
27. Tinetti ME, Bogardus ST, Jr., Agostini JV. Potential pitfalls of disease-specific guidelines for patients with multiple conditions. *The New England Journal of Medicine*. 2004;351:2870-2874
28. Tegn N, Abdelnoor M, Aaberge L, Endresen K, Smith P, Aakhus S, Gjertsen E, Dahl-Hofseth O, Ranhoff AH, Gullestad L, Bendz B. Invasive versus conservative strategy in patients aged 80 years or older with non-st-elevation myocardial infarction or unstable angina pectoris (after eighty study): An open-label randomised controlled trial. *Lancet*. 2016

29. Seto TB, Taira DA, Berezin R, Chauhan MS, Cutlip DE, Ho KK, Kuntz RE, Cohen DJ. Percutaneous coronary revascularization in elderly patients: Impact on functional status and quality of life. *Annals of Internal Medicine*. 2000;132:955-958
30. Chait R, Zad O, Ramineni R, Shukla A, Mitchell A. Midterm outcomes and quality of life following percutaneous coronary intervention in nonagenarians. *The American Journal of Cardiology*. 2011;107:1609-1612
31. Machin D, Campbell MJ, Tan SB, Tan SH. Comparing survival curves. *Sample size tables for clinical studies*. Wiley-Blackwell; 2009:84-101.
32. Zaman MJ, Stirling S, Shepstone L, Ryding A, Flather M, Bachmann M, Myint PK. The association between older age and receipt of care and outcomes in patients with acute coronary syndromes: A cohort study of the myocardial ischaemia national audit project (minap). *European Heart Journal*. 2014;35:1551-1558
33. Beecham J KM. Costing psychiatric interventions. In: Thornicroft, graham, ed. Measuring mental health needs (second edition). Royal college of psychiatrists, london, 2001, 200-224. *Measuring Mental Health Needs* 2001;Second Edition 24
34. Patel A RA, Moran S, et al. A comparison of two methods of collecting economic data in primary care. *Family Practice 2005;22:323-7*
35. Caro JJ, Briggs AH, Siebert U, Kuntz KM. Modeling good research practices--overview: A report of the ispor-smdm modeling good research practices task force. *Value in health : the Journal of the International Society for Pharmacoeconomics and Outcomes Research*. 2012;15:796-803
36. Thygesen K, Alpert JS, Jaffe AS, Simoons ML, Chaitman BR, White HD, Thygesen K, Alpert JS, White HD, Jaffe AS, Katus HA, Apple FS, Lindahl B, Morrow DA, Chaitman BA, Clemmensen PM, Johanson P, Hod H, Underwood R, Bax JJ, Bonow RO, Pinto F, Gibbons RJ, Fox KA, Atar D, Newby LK, Galvani M, Hamm CW, Uretsky BF, Steg PG, Wijns W, Bassand JP, Menasche P, Ravkilde J, Ohman EM, Antman EM, Wallentin LC, Armstrong PW, Simoons ML, Januzzi JL, Nieminen MS, Gheorghiade M, Filippatos G, Luepker RV, Fortmann SP, Rosamond WD, Levy D, Wood D, Smith SC, Hu D, Lopez-Sendon JL, Robertson RM, Weaver D, Tendera M, Bove AA, Parkhomenko AN, Vasilieva EJ, Mendis S. Third universal definition of myocardial infarction. *European Heart Journal*. 2012;33:2551-2567
37. Hochman JS, Sleeper LA, Webb JG, Sanborn TA, White HD, Talley JD, Buller CE, Jacobs AK, Slater JN, Col J, McKinlay SM, LeJemtel TH. Early revascularization in acute myocardial infarction complicated by cardiogenic shock. Shock investigators. Should we emergently revascularize occluded coronaries for cardiogenic shock. *The New England Journal of Medicine*. 1999;341:625-634
38. Alexander JH, Reynolds HR, Stebbins AL, Dzavik V, Harrington RA, Van de Werf F, Hochman JS. Effect of tilarginine acetate in patients with acute myocardial infarction and cardiogenic shock: The triumph randomized controlled trial. *JAMA : the Journal of the American Medical Association*. 2007;297:1657-1666
39. Sacco RL, Kasner SE, Broderick JP, Caplan LR, Connors JJ, Culebras A, Elkind MS, George MG, Hamdan AD, Higashida RT, Hoh BL, Janis LS, Kase CS, Kleindorfer DO, Lee JM, Moseley ME, Peterson ED, Turan TN, Valderrama AL, Vinters HV. An updated definition of stroke for the 21st century: A statement for healthcare professionals from the american heart association/american stroke association. *Stroke; a Journal of Cerebral Circulation*. 2013;44:2064-2089

16. APPENDICES

16.1. Definitions

Myocardial Infarction:

Myocardial infarction (MI) is defined by the third universal definition.[36]

Type 1 MI: Spontaneous MI

This is an event related to atherosclerotic plaque rupture, ulceration, fissuring, erosion, or dissection with resulting intraluminal thrombus in one or more of the coronary arteries, leading to decreased myocardial blood flow or distal platelet emboli with ensuing myocyte necrosis.

Type 2 MI: Secondary to an ischaemic imbalance

In instances of myocardial injury with necrosis where a condition other than CAD contributes to an imbalance between myocardial oxygen supply and/or demand, e.g. coronary endothelial dysfunction, coronary artery spasm, coronary embolism, tachy-/brady-arrhythmias, anaemia, respiratory failure, hypotension, and hypertension with or without LVH

Type 3 MI: Cardiac death due to myocardial infarction

Cardiac death with symptoms suggestive of myocardial ischaemia and presumed new ischaemic ECG changes or new LBBB, but death occurring before blood samples could be obtained, before cardiac biomarker could rise, or in rare cases cardiac biomarkers were not collected.

Type 4a: MI related to PCI

Myocardial infarction associated with PCI is arbitrarily defined by elevation of cTn values >5 x 99th centile URL in patients with normal baseline values (<99th percentile URL) or a rise of cTn values >20% if the baseline values are elevated and are stable or falling. In addition, either (i) symptoms suggestive of myocardial ischemia, or (ii) new ischemic ECG changes or new LBBB, or (iii) angiographic loss of patency of a major coronary artery or a side branch or persistent slow- or no-flow or embolization, or (iv) imaging demonstration of new loss of viable myocardium or new regional wall motion abnormality are required.

Type 4b: MI related to stent thrombosis

Myocardial infarction associated with stent thrombosis is detected by coronary angiography or autopsy in the setting of myocardial ischemia and with a rise and/ or fall of cardiac biomarkers values with at least one value above the 99th centile URL.

Type 5: MI related to coronary artery bypass grafting (CABG)

Myocardial infarction associated with CABG is arbitrarily defined by elevation of cardiac biomarker values >10 x 99th centile URL in patients with normal baseline cTn values (<99th centile URL). In addition, either (i) new pathological Q waves or new LBBB, or (ii) angiographic documented new graft or new native coronary artery occlusion, or (iii) imaging evidence of new loss of viable myocardium or new regional

wall motion abnormality.

Cardiogenic shock: Cardiogenic shock describes the physiological state in which reduced cardiac output (CO) and resultant tissue hypoxia occur in the presence of adequate intravascular volume. Haemodynamically, this is defined as systolic blood pressure below 90 mmHg sustained for at least 30 minutes in the absence of hypovolaemia, with a cardiac index below 1.8 Litres/minute/metre^2 without support or 2.0-2.2 Litres/minute/metre^2 with support, in the presence of a raised pulmonary capillary wedge pressure (>15 mmHg).[37, 38]

Stroke

Stroke is defined according to the American Heart Association/American Stroke Association updated definition of stroke for the 21st century.[39]

Definition of ischaemic stroke: An episode of neurological dysfunction caused by focal cerebral, spinal, or retinal infarction.

Definition of intracerebral hemorrhage: A focal collection of blood within the brain parenchyma or ventricular system that is not caused by trauma.

16.2. Safety Reporting Diagram

The diagram may require editing depending upon the requirements of the trial and the sponsor

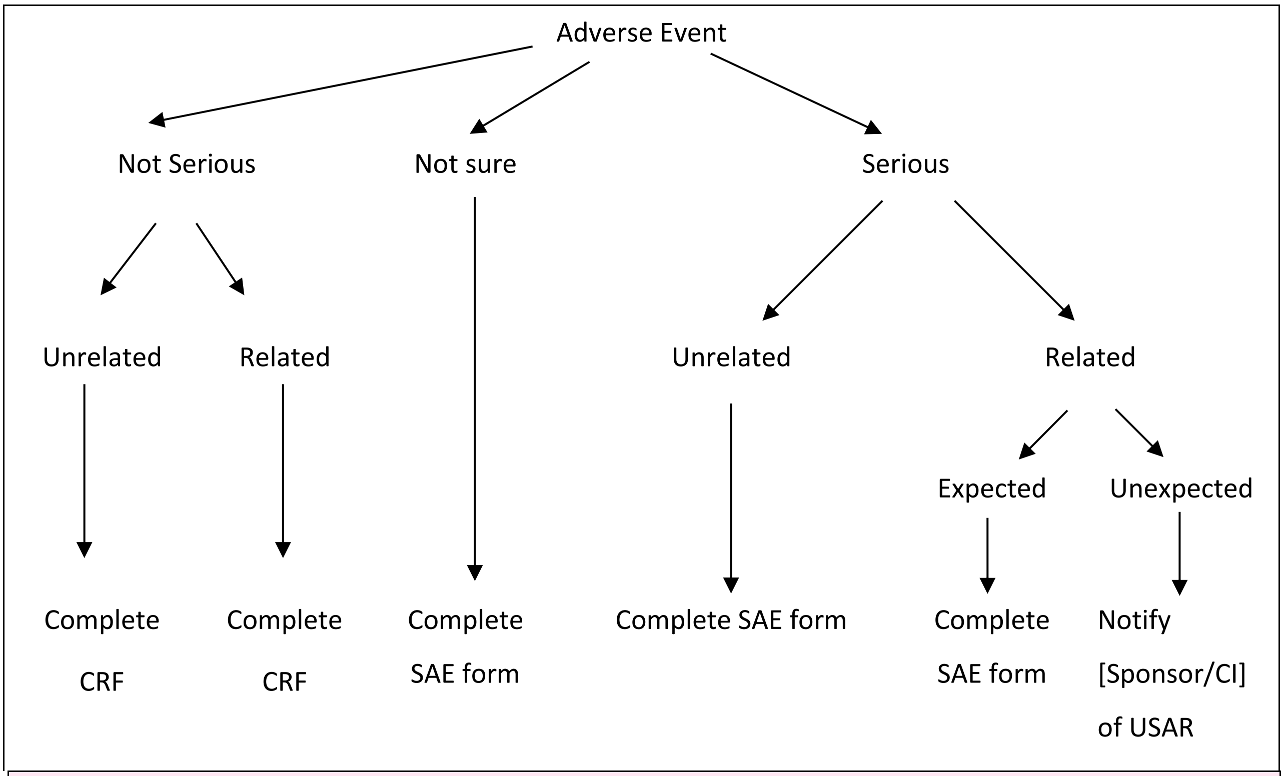

Contact details for reporting SAEs and USARs

Please send form(s) via fax to ***0191 5800866***

or

Contact Trial Administrator **0191 208 8932**

16.3. Amendment History

Amendment Number	Protocol version no.	Date issued	Author(s) of changes	Details of changes made

16.4. Fried Frailty Index

Criterion	Frailty Status
**Shrinking**	**Frailty cut point:**<br>**Baseline:** Self reported unintentional weight loss ≥10lb in previous year<br>**Follow-up:** Unintentional weight loss ≥5% of previous year's body weight<br><u>*OR*</u><br>BMI <18.5kg/m^2
**Physical endurance/energy**	*Geriatric Depression Scale:*<br>1. *Do you feel full of energy?*<br>2. *During the last 4 weeks how often you rested in bed during day?*<br><br><u>Response options:</u> Every day, every week, once, not at all.<br><br>**Frailty cut point:**<br>No to 1 and every day or every week to 2.
**Low physical activity**	*Frequency of mildly energetic, moderately energetic and very energetic physical activity.*<br><br><u>Response options:</u> ≥3 times per week, 1-2 times per week, 1-3 times per month, hardly ever/never<br><br>**Frailty cut point:**<br>Hardly ever/never for very energetic physical activity AND for moderately energetic physical activity.
**Weakness**	Hand grip strength in Kg: GRIP-D hand held dynamometer, dominant hand, average of 3 measures.<br><br>**Frailty cut point:**<br>**Grip strength:** lowest 20% (by gender, body mass index)<br>*Men*<br>BMI ≤24 ≤29<br>BMI 24.1–26 ≤30<br>BMI 26.1–28 ≤30<br>BMI >28 ≤32<br>*Women*<br>BMI ≤23 ≤17<br>BMI 23.1–26 ≤17.3<br>BMI 26.1–29 ≤18<br>BMI >29 ≤21
**Slow walking speed**	Walking time in seconds (usual pace) over 15 feet<br><br>**Frailty cut point:**<br>Slowest 20%, stratified by gender and median standing height.<br>*Men*<br>Height ≤173 cm ≥7 seconds<br>Height >173 cm ≥6 seconds<br>*Women*<br>Height ≤159 cm ≥7 seconds<br>Height >159 cm ≥6 seconds<br><br><u>*OR*</u><br>Time to complete "timed up and go test" (TUG)<br><br>**Frailty cut point:**<br>TUG time ≥19 seconds

Frail: ≥3 criteria present; **Intermediate or Pre-Frail:**1 or 2 criteria present; **Robust :** 0 criteria present

Adapted from Fried et al, Cardiovascular Health Study Collaborative Research G. Frailty in older adults: Evidence for a phenotype. The Journals of Gerontology. Series A, Biological sciences and medical sciences. 2001;56:M146-156.

16.5. Rockwood Frailty Index

1	Very fit – robust, active, energetic, well-motivated and fit; these people commonly exercise regularly and are in the most fit group for their age
2	Well – without active disease, but less fit than people in category 1.
3	Well, with treated co-morbid disease – disease symptoms are well controlled compared with those in category 4
4	Apparently vulnerable – although not frankly dependent, these people commonly complain of being “slowed up” or have disease symptoms.
5	Mildly frail – with limited dependence on others for instrumental activities of daily living
6	Moderately frail – help is needed with both instrumental and non-instrumental activities of daily living
7	Severely frail – completely dependent on others for the activities of daily living, or terminally ill.

Adapted from Rockwood et al, A global clinical measure of fitness and frailty in elderly people. Canadian Medical Association Journal 2005;173:489-495

16.6. Bleeding Academic Research Consortium definition for bleeding

Type 0	No bleeding
Type 1	Bleeding that is not actionable and does not cause the patient to seek unscheduled performance of studies, hospitalization, or treatment by a healthcare professional. May include episodes leading to self-discontinuation of medical therapy by the patient without consulting a healthcare professional.
Type 2	Any overt, actionable sign of haemorrhage (e.g. more bleeding than would be expected for a clinical circumstance, including bleeding found by imaging alone) that does not fit the criteria for Type 3, 4 or 5 but does meet at least one of the following criteria: (1) requiring non-surgical, medical intervention by a healthcare professional, (2) leading to hospitalization or increased level of care, or (3) prompting evaluation.
Type 3a	Overt bleeding plus haemoglobin drop of 3 to <5g/dl* (provided haemoglobin drop is due to bleed)<br>Any transfusion with overt bleeding
Type 3b	Overt bleeding plus haemoglobin drop ≥5g/dl* (provided haemoglobin drop is due to bleed)<br>Cardiac tamponade<br>Bleeding requiring surgical intervention for control (excluding dental/ nasal/ skin/ haemorrhoid)<br>Bleeding requiring intravenous vasoactive agents
Type 3c	Intracranial haemorrhage (does not include micro-bleeds or haemorrhagic transformation, does include intraspinal)<br>Subcategories confirmed by autopsy or imaging or lumbar puncture<br>Intraocular bleed compromising vision
Type 4:	CABG-related bleeding<br>Perioperative intracranial bleeding within 48 hours<br>Reoperation following closure of sternotomy for the purpose of controlling bleeding<br>Transfusion of ≥5 units of whole blood or packed red blood cells within a 48-hour period†<br>Chest tube output ≥ 2 litres within a 24-hour period<br>If a CABG-related bleed is not adjudicated as at least a Type 3 severity event, it will be classified as 'not a bleeding event'.
Type 5a	Probable fatal bleeding; no autopsy or imaging confirmation, but clinically suspicious
Type 5b	Definite fatal bleeding; overt bleeding or autopsy or imaging confirmation

**Corrected for transfusion (1 U packed red blood cells or 1 U whole blood1 g/dL haemoglobin).*
†Cell saver products are not counted.

Adapted from Mehran et al, Standardized bleeding definitions for cardiovascular clinical trials: A consensus report from the bleeding academic research consortium. Circulation. 2011;123:2736-274

16.7. EQ-5D-5L

A sample English (UK) version of the EQ-5D-5L, to be completed by trial participants is available to view at the link below:

http://www.euroqol.org/fileadmin/user_upload/Documenten/PDF/Products/Sample_UK__English__EQ-5D-5L_Paper_Self_complete_v1.0__ID_24700_.pdf

In addition to the participant completed version a proxy version of the EQ-5D-5L will be completed for all participants at all time points. Please note that an online sample for this version of the EQ-5D-5L is not available, but more information can be found at the link below:

http://www.euroqol.org/eq-5d-products/eq-5d-5l/proxy-paper.html

We anticipate that a proportion of participants will not be able to complete the paper version. For these participants the telephone version will be completed: Please note that an online sample for this version of the EQ-5D-5: is not available, but more information can be found at the link below

http://www.euroqol.org/eq-5d-products/eq-5d-5l/telephone.html

**Full Title:**	The British Heart Foundation older patients with non-ST SEgmeNt elevatIOn myocaRdial infarction Randomized Interventional TreAtment Trial
**Short Title/Acronym:**	The BHF SENIOR-RITA Trial
**Protocol Version Number & Date:**	Version 3.0<br>02 July 2020

Statement: This protocol has regard for the HRA guidance.

RESEARCH REFERENCE NUMBERS

**IRAS Number:**	**204031**
**NHS REC Reference:**	**16/NE/0238**
**ClinicalTrials.gov Identifier:**	**NCT03052036**

RESEARCH SPONSOR

**Sponsor Name:**	Newcastle upon Tyne Hospitals NHS Foundation Trust
**Sponsor Reference:**	**7910**

RESEARCH FUNDER

**Funder Name:**	British Heart Foundation
**Funder Reference:**	CS/15/7/31679

SIGNATURE PAGE

The undersigned confirm that the following protocol has been agreed and accepted. The Chief Investigator agrees to conduct the trial in compliance with the approved protocol and will adhere to the Research Governance Framework, Good Clinical Practice (GCP) guidelines, the relevant Standard Operating Procedures and other regulatory requirements as applicable.

I agree to ensure that the confidential information contained in this document will not be used for any other purpose other than the evaluation or conduct of the investigation without the prior written consent of the Sponsor.

Representative of the Research Sponsor

Name: **Mr. Sean Scott**

Position: **Regulatory Compliance Manager**

Signature: **Date:**

Chief Investigator

Name: **Dr. Vijay Kunadian**

Position: **Academic Consultant Interventional Cardiologist, Newcastle University**

Signature: **Date:**

Senior Statistician

Name: **Prof Dawn Teare**

Position: **Professor of Biostatistics, Population Health Sciences Institute, Newcastle University**

Signature: **Date:**

Senior Trial Manager

Name: **Michelle Bardgett**

Position: **Senior Trial Manager, NCTU**

Signature: **Date:**

Trial Managers

Name: **Philippa Watts and Vicky Wheeldon**

Position **Trial Manager, NCTU**

Signature: **Date**

Database Manager

Name: **Mr Jonathan Prichard**

Position: **Database Manager, NCTU**

Signature: **Date:**

Principal Investigator Signature

I confirm that I have read and understood protocol version 3.0 dated 02 July 2020. I agree to comply with the study protocol, the principles of Good Clinical Practice (GCP), research governance, clinical trial regulations and appropriate reporting requirements.

Signature …………………………….……….. Date ………………..…

Print Name ……………………………..…….…

Site Name/I.D. ……………………………....

KEY TRIAL CONTACTS

**Chief Investigator**	Dr. Vijay Kunadian<br>Academic Consultant Interventional Cardiologist<br>M4:146 4th Floor William Leech Building<br>Medical School<br>Newcastle upon Tyne<br>NE2 4HH<br>Tel: +44 (0) 191 208 5797<br>E-mail: vijay.kunadian@newcastle.ac.uk
**Senior Trial Manager**	Michelle Bardgett<br>Newcastle Clinical Trials Unit<br>Newcastle University<br>1-4 Claremont Terrace<br>Newcastle upon Tyne<br>NE2 4AE<br>Telephone: +44 (0) 191 208 2597<br>Email: michelle.bardgett@newcastle.ac.uk
**Trial Managers**	Philippa Watts and Vicky Wheeldon<br>Newcastle Clinical Trials Unit<br>Newcastle University<br>1-4 Claremont Terrace<br>Newcastle upon Tyne<br>NE2 4AE<br>Telephone: +44 (0) 191 208 4591<br>Email: philippa.watts@newcastle.ac.uk<br>victoria.wheeldon@newcastle.ac.uk
**Database Manager**	Jonathan Prichard<br>Newcastle Clinical Trials Unit<br>Newcastle University<br>1-4 Claremont Terrace<br>Newcastle upon Tyne<br>NE2 4AE<br>Telephone: +44 (0) 191 208 2518<br>Email: jonathan.prichard@newcastle.ac.uk
**Senior Statistician**	Prof Dawn Teare<br>Professor of Biostatistics<br>Population Health Sciences Institute<br>Newcastle University<br>Baddiley-Clark Building<br>Richardson Road<br>Newcastle upon Tyne<br>NE2 4AX<br>Email: dawn.teare@newcastle.ac.uk

**Trial Statistician**	Helen Mossop<br>Population Health Sciences Institute<br>Newcastle University<br>Baddiley-Clark Building<br>Richardson Road<br>Newcastle upon Tyne<br>NE2 4AX<br>Email: helen.mossop@newcastle.ac.uk
**Sponsor**	Sean Scott<br>Regulatory Compliance Manager<br>Newcastle upon Tyne Hospitals NHS Foundation Trust<br>Level 1, Regent Point<br>Regent Farm Road<br>Gosforth<br>Newcastle upon Tyne<br>NE3 3HD<br>Tel: +44 (0)191 282 4461<br>Email: sean.scott@nhs.net
**Funder(s)**	Mrs. Alex Mazzetta<br>Head of Research Funds<br>British Heart Foundation<br>Greater London House, 180 Hampstead Road<br>London<br>NW1 7AW<br>Tel: 020 7554 0434<br>Email: research@bhf.org.uk
**Collaborators/Co-Investigators**	Professor David Newby<br>BHF Duke of Edinburgh Chair of Cardiology<br>Centre for Cardiovascular Science<br>The Queen's Medical Research Institute<br>49 Little France Crescent<br>University of Edinburgh<br>Edinburgh EH16 4SB<br>Tel: +44 (0) 131 242 6515<br>Email: d.e.newby@ed.ac.uk<br><br>Professor Helen Hancock<br>Director, Newcastle Clinical Trials Unit<br>Newcastle University<br>1-2 Claremont Terrace<br>Newcastle upon Tyne<br>NE2 4AE<br>Tel: +44 (0) 191 208 2516<br>E-mail: helen.hancock@newcastle.ac.uk

	Professor Luke Vale<br>Professor of Health Economics<br>Population Health Sciences Institute<br>Newcastle University<br>Baddiley-Clark Building<br>Richardson Road<br>Newcastle upon Tyne<br>NE2 4AA<br>Tel: +44 (0) 191 2085590<br>Email: luke.vale@newcastle.ac.uk<br><br>Professor Stuart Parker<br>William Leech Professor of Geriatric Medicine<br>Institute for Ageing and Health<br>Newcastle University, Campus for Ageing and Vitality<br>Newcastle upon Tyne<br>NE4 5PL<br>Tel: +44 (0) 191 2081215<br>Email: stuart.parker@newcastle.ac.uk<br><br>Professor Robert Storey<br>Professor and Honorary Consultant in Cardiology<br>Dept of Infection, Immunity & Cardiovascular Disease<br>University of Sheffield<br>Medical School<br>Beech Hill Road<br>Sheffield<br>S10 2RX<br>Tel: +44 (0) 114 215 9554<br>Email: r.f.storey@sheffield.ac.uk<br><br>Professor Colin Berry<br>Professor of Cardiology and Imaging<br>RC309 Level C3<br>Institute of C&MS<br>BHF GCRC<br>Glasgow G12 8TA<br>Tel: +44 (0) 1413 301671<br>Email: colin.berry@glasgow.ac.uk<br><br>Professor Mark de Belder MA MD FRCP<br>National Institute for Cardiovascular Outcomes Research<br>Barts Health NHS Trust<br>2nd Floor, 1 St Martin's le Grand<br>London EC1A 4NP<br>Email: mark.debelder@nhs.net
**Committees**	**Trial Steering Committee (Independent)**

	Professor Keith Fox (Chair)<br>Emeritus Professor of Cardiology<br>Centre for Cardiovascular Science<br>The Queen's Medical Research Institute<br>47 Little France Crescent<br>University of Edinburgh<br>Edinburgh EH16 4TJ<br>Email: k.a.a.fox@ed.ac.uk<br><br>Professor Gary Ford<br>Chief Executive Officer of the Oxford AHSN<br>Oxford Science Park<br>Oxford OX4 4GA<br>Tel: +44 (0) 1865 784957<br>Email: gary.ford@ouh.nhs.uk<br><br>Professor Marcus Flather<br>Clinical Professor in Medicine<br>Norwich Medical School<br>University of East Anglia<br>Norwich NR4 7TJ<br>Tel: +44 (0) 1603 591420<br>Email: m.flather@uea.ac.uk<br><br>Professor Rajesh Kharbanda<br>Associate Professor of Cardiovascular Medicine, Consultant Cardiologist<br>Oxford University Hospitals<br>Headley Way<br>Oxford OX3 9DU<br>Tel: +44 (0) 1865 220325<br>Email: Rajesh.Kharbanda@ouh.nhs.uk<br><br>Professor Toby Prevost<br>Nightingale-Saunders Chair in Complex Clinical Trials and Statistics<br>Director, Nightingale-Saunders Clinical Trials and Epidemiology Unit @ King's CTU<br>Florence Nightingale Faculty of Nursing Midwifery and Palliative Care<br>King's College London<br>James Clerk Maxwell Building,<br>57 Waterloo Road, London SE1 8WA<br>and Cicely Saunders Institute, Bessemer Road, Denmark Hill, London SE5 9PJ<br>Tel: +44 (0) 7557 308168<br>Email: toby.1.prevost@kcl.ac.uk<br><br>Mr. David Inness (Lay member)<br>VOICE<br>UK National Innovation Centre for Ageing

	The Catalyst, 3 Science Square,<br>Newcastle Helix<br>Newcastle upon Tyne NE4 5TG<br>Tel: +44 (0) 191 208 2503<br>Email: dbi@talktalk.net<br><br>Dr. Shannon Amoils (BHF Representative)<br>British Heart Foundation<br>Greater London House<br>180 Hampstead Road<br>London NW1 7AW<br>Tel: 020 7554 0360<br>Email: amoilss@bhf.org.uk<br><br>**Data Monitoring Committee**<br>Professor Stuart Pocock (Chair)<br>Room, G34a, Medical Statistics Unit<br>LSHTM, Keppel Street<br>London WC1E 7HT<br>Tel: +44 (0)20 7927 2413<br>Email: stuart.pocock@lshtm.ac.uk<br><br>Professor Ajay Shah<br>King's College London<br>The James Black Centre<br>125 Coldharbour Lane<br>London SE5 9NU<br>Tel: +44 (0)20 7848 5189<br>Email: ajay.shah@kcl.ac.uk<br><br>Professor Robert Guigliano, MD<br>Professor of Medicine<br>TIMI Study Group<br>Cardiovascular Medicine<br>Brigham and Women's Hospital<br>Boston, MA 02115, USA<br>Tel: +1 617 278-0145<br>E-mail: rgiugliano@bwh.harvard.edu<br><br>Dr. Ian Reeves<br>Consultant Geriatrician<br>Greater Glasgow and Clyde Valley<br>Glasgow<br>Email: i.reeves@nhs.net<br><br>Professor Stephen Leslie<br>Consultant Cardiologist (NHS Highland)<br>Associate Director Research, Development & Innovation<br>Cardiac Unit<br>Raigmore Hospital, Old Perth Road

	Inverness, IV2 3UJ<br>Tel: 01463 705459<br>Email: stephen.leslie@nhs.net<br>**Clinical Events Committee**<br>Dr. Martin Denvir<br>Consultant Cardiologist<br>Centre for Cardiovascular Science<br>The Queen's Medical Research Institute<br>47 Little France Crescent<br>University of Edinburgh<br>Edinburgh EH16 4TJ<br>Tel: 0131 242 9236<br>E-mail: martin.denvir@ed.ac.uk<br><br>Dr. Anoop Shah<br>London School of Hygiene & Tropical Medicine<br>Keppel Street<br>London<br>WC1E 7HT<br>E-mail: anoop.shah@lshtm.ac.uk<br><br>Dr. Ifti Haq<br>Consultant Cardiologist<br>Newcastle upon Tyne Hospitals NHS Foundation Trust<br>Newcastle upon Tyne NE7 7ND<br>Tel: 0191 2336161<br>E-mail: ifti.haq@nhs.net<br><br><br>Dr. Atul Anand<br>Centre for Cardiovascular Science<br>The Queen's Medical Research Institute<br>47 Little France Crescent<br>University of Edinburgh<br>Edinburgh EH16 4TJ<br>Tel: 0131 242 6537<br>E-mail: atal.anand@ed.ac.uk<br><br>**Trial Management Group**<br>Dr. Vijay Kunadian<br>Professor Dave Newby<br>Professor Helen Hancock<br>Professor Luke Vale<br>Professor Stuart Parker<br>Professor Dawn Teare<br>Michelle Bardgett<br>Philippa Watts<br>Vicky Wheeldon<br>Jon Prichard<br>Carol Shields<br>Helen Mossop

	Janet Jobling
**Trial Website**	https://research.ncl.ac.uk/seniorrita/

TRIAL SUMMARY

**Trial Title**	The British Heart Foundation older patients with non-ST SEgmeNt elevatIOn myocaRdial infarction Randomized Interventional TreAtment Trial
**Acronym**	SENIOR-RITA
**Summary of Trial Design**	SENIOR-RITA is a multicentre prospective open-label trial randomizing patients presenting with type 1 NSTEMI aged ≥75 years between invasive and conservative treatment strategies, to compare time to cardiovascular death or non-fatal MI
**Participant Population**	Older patients aged ≥75 years presenting with type 1 NSTEMI
**Planned Sample Size**	1668
**Number of Sites**	Circa 40 sites
**Intervention Duration**	During index presentation with type 1 NSTEMI
**Follow Up Duration**	A minimum follow-up of 1year for the primary endpoint (with additional 5 and 10 year follow-up)
**Planned Trial Period**	7 Years (for primary endpoint)
**Primary Objective**	To determine the impact of a routine invasive strategy on cardiovascular death and non-fatal myocardial infarction (MI) compared with a conservative treatment strategy in older patients (≥75 years) with NSTEMI.
**Secondary Objectives**	To determine the impact of a routine invasive strategy compared with a conservative strategy on:<br>1. All-cause death<br>2. Cardiovascular and non-cardiovascular death<br>3. Recurrent myocardial infarction<br>4. Coronary angiography and coronary revascularisation<br>5. Hospitalization for heart failure<br>6. Stroke<br>7. TIA<br>8. Bleeding (BARC ≥2)<br>9. Procedural and in-hospital complications<br>10. The length of time spent at home<br>11. Frailty and quality of life<br>12. Cost-effectiveness
**Intervention**	Coronary angiography ± revascularisation and optimal medical therapy *versus* optimal medical therapy alone

Contents

RESEARCH REFERENCE NUMBERS 2
SIGNATURE PAGE 3
Principal Investigator Signature 4
KEY TRIAL CONTACTS 5
TRIAL SUMMARY 11
GLOSSARY OF ABREVIATIONS 15
RESPONSIBILITIES 17
1. BACKGROUND and RATIONALE 19
2. OBJECTIVES AND OUTCOME MEASURES 22
2.1. Primary Objective 22
2.2. Secondary Objectives 22
2.3. Outcome Measures 22
3. TRIAL DESIGN 24
4. STUDY SETTING 25
5. ELIGIBILITY CRITERIA 25
5.1. Inclusion Criteria 25
5.2. Exclusion Criteria 25
6. TRIAL PROCEDURES 26
6.1. Patient Identification and Screening 26
6.2. Consent 26
6.3. Randomisation 27
6.4. Data collection 27
6.5. Long-term follow-up 29
6.6. Schedule of events 30
6.7 Withdrawal Criteria 31
6.8 End of Trial 31
7. TRIAL INTERVENTIONS 32
7.1. Treatment Strategy 32
7.1.1. Schedule & Modifications 32
7.1.2. Known Risks 32
7.2. Concomitant Medications & Therapies 32
8. SAFETY REPORTING 33

8.1. Standard Definitions 33
8.2. Severity (Intensity) of Serious Adverse Events and Reactions 34
8.3. Assessment of Causality 34
8.4. Recording and Reporting AEs and SAEs 35
8.5. Responsibilities 38
8.6. Reporting Urgent Safety Measures 39
9. STATISTICAL CONSIDERATIONS 39
9.1. Analysis Populations 39
9.2. Statistical Analyses 39
9.2.1. Analyses of the Primary Outcome Measure 39
9.2.2. Analyses of Secondary Outcome Measures 39
9.2.3. Subgroup Analyses 40
9.2.4. Planned Additional Analyses 38
9.2.5. Interim Analyses and Criteria for the Premature Termination of the Trial 38
9.3. Sample Size Calculations 39
10. ECONOMIC EVALUATION 42
10.1. Assessment of Cost 42
10.2. Economic Analysis 43
11. DATA HANDLING 44
11.1. Data Collection Tools and Source Document Identification 44
11.2. Data Handling and Record Keeping 44
11.3. Access to Data 44
11.4 Archiving 44
12. MONITORING, AUDIT & INSPECTION 45
13. ETHICAL AND REGULATORY CONSIDERATIONS 47
13.1. Research Ethics Committee Review and Reports 47
13.2. Public and Patient Involvement 47
13.3. Regulatory Compliance 47
13.4. Protocol Compliance 47
13.5. Notification of Serious Breaches to GCP and/or the Protocol 48
13.6. Data Protection and Patient Confidentiality 48
13.7. Indemnity 48
13.8. Amendments 48
14. DISSEMINATION POLICY 49

15. REFERENCES 48
16. APPENDICES 53
16.1. Definitions 53
16.2. Amendment History 54
16.3. Fried Frailty Index 57
16.4. Rockwood Frailty Index 58
16.5. Bleeding Academic Research Consortium definition for bleeding 59
16.6. EQ-5D-5L 58
16.7. Further discussion on sample size and power 58

GLOSSARY OF ABREVIATIONS

**ABBREVIATION**	**DEFINITION**
ACS	Acute coronary syndrome
AE	Adverse Event
AR	Adverse Reaction
CABG	Coronary artery bypass surgery
CAG	Confidentiality Advisory Group
CAD	Coronary artery disease
CEAC	Cost-effectiveness acceptability curve
CEC	Clinical events committee
CHD	Coronary heart disease
CI	Chief Investigator
CRF/eCRF	Case Report Form/electronic Case Report Form
DMC	Data Monitoring Committee
GBP	Pound Sterling
GCP	Good Clinical Practice
HRA	Health Research Authority
HR	Hazard Ratio
ICF	Informed Consent Form
IRMER	Ionising Radiation (Medical Exposure) Regulations
ISF	Investigator Site File
ISRCTN	International Standard Randomized Controlled Trials Number
MI	Myocardial infarction
MoCA	Montreal Cognitive Assessment
NCTU	Newcastle Clinical Trials Unit
NHS	National Health Service
NSTEACS	Non-ST elevation acute coronary syndrome
NSTEMI	Non-ST elevation myocardial infarction
PBPP	Public Benefit and Privacy Panel for Health and Social Care
PCI	Percutaneous coronary intervention
PHS	Public Health Scotland
PI	Principal Investigator
PIS	Participant Information Sheet
QA	Quality Assurance
QALY	Quality-Adjusted Life Year
QC	Quality Control
QOL	Quality of Life
R&D	Research & Development
RCT	Randomized Control Trial
REC	Research Ethics Committee
SAE	Serious Adverse Event
SAR	Serious Adverse Reaction
SDV	Source Data Verification
SOP	Standard Operating Procedure
SSI	Site Specific Information
USAR	Unexpected Serious Adverse Reaction
TMG	Trial Management Group
TSC	Trial Steering Committee

TMF	Trial Master File

RESPONSIBILITIES

Sponsor: The nominated Sponsor for the study is The Newcastle upon Tyne Hospitals NHS Foundation Trust who will undertake a Research Governance Risk Assessment prior to commencement of the study.

Funder: The British Heart Foundation, Greater London House, 180 Hampstead Road, London, NW1 7AW

Trial Management: The study will be managed through the UKCRC registered Newcastle Clinical Trials Unit (Unit number 22). A Trial Management Group (TMG) will be responsible for overseeing the progress of the trial. The full list of TMG members is shown above.

Chief Investigator: The Chief Investigator will have overall responsibility for the conduct of the study.

Principal Investigator responsibility:

- Study conduct and the welfare of study subjects
- Compliance with the protocol, documentation of any protocol deviations and reporting of all serious adverse events
- Screening and recruitment of subjects
- Ensuring all trial-related medical decisions are made by a qualified physician, who is an investigator or co-investigator for the trial.
- Provision of adequate medical care in the event of an adverse event
- Obtaining local approval and abiding by the policies of Research Governance
- Compliance with the Principles of GCP, the Research Governance Framework for Health and Social Care, and the Data Protection Act
- Ensuring that no participant is recruited into the study until all relevant local regulatory permissions and approvals have been obtained.
- Obtaining written informed consent from participants prior to any study specific procedures
- The Principal Investigator shall be qualified by education, training and experience to assume responsibility for the proper conduct of the trial. S/he shall provide a current signed & dated curriculum vitae as evidence for the Trial Master File (TMF).
- The Principal Investigator shall ensure that site personnel are suitably trained for any task that they have delegated, and are named on the delegation log. A copy of the delegation log should be sent to the Trial Manager NCTU for inclusion in the TMF
- Ensuring Study Site team members are appropriately qualified by education, training and experience to undertake the conduct of the study.
- Availability for monitoring visits and in the case of an audit
- Maintaining study documentation and compliance with reporting requests
- Maintaining a site file, including copies of study approval, list of subjects and their signed informed consent forms
- Documenting appropriate delegation of tasks to study personnel e.g. Research Nurse, Investigator(s)
- Ensuring data collected is accurate, timely and complete

- Ensuring subject confidentiality is maintained during the project and archival period
- Ensuring archival of study documentation for a minimum of 5 years following the end of the study, unless local arrangements require a longer period

1. BACKGROUND and RATIONALE

Our population is ageing. Age is a powerful predictor of adverse events following acute myocardial infarction and percutaneous coronary intervention (PCI): adjusted odds for in-hospital death increase by 70% for each 10-year increase in age. Older patients are often frail with up to a half of older patients (≥75 years) admitted with non-ST segment elevation myocardial infarction (NSTEMI) being severely frail. These frail older patients have a 4-fold increased risk of death at 1-year and yet are often denied coronary revascularisation because of fear of complications and causing harm. The goals of this study are to determine the benefits and risks of invasive coronary angiography and coronary revascularisation in older patients presenting with NSTEMI and receiving optimal medical therapy.

Invasive Coronary Angiography and Coronary Revascularisation

A strategy of routine invasive coronary angiography with a view to coronary revascularisation reduces recurrent myocardial infarction (MI) and cardiovascular death among patients with NSTEMI (hazard ratio [HR]: 0.81, 95% confidence interval [CI]: 0.71-0.93; p=0.002).(1) In the British Heart Foundation RITA-3 trial (mean age 62 years),(2) a composite outcome of death, myocardial infarction (MI) and refractory angina was markedly reduced in the intervention arm compared with the conservative arm at 4 months (9.6% vs. 14.5%, p=0.001).

Acute Coronary Syndrome (ACS) in Older Patients

Although most developed countries have accepted the chronological age of 65 years as a definition of “elderly” or “older person”,(3) based on comprehensive analysis of data from a survey on public attitudes, surveys on older patients requiring nursing care, longitudinal studies of functional independence in the elderly and clinical and pathological data, it has been suggested to change the definition of elderly to those ≥75 years of age instead of the current 65 years.(4) We therefore refer to those aged ≥75 years as “older patients”.

In the UK, more than twice as many individuals ≥75 years of age (n=55,028) die from coronary heart disease (CHD) than younger individuals <75 years (n=25,540).(5) In the Global Registry of Acute Coronary Events (GRACE), increasing age was associated with increased incidence of NSTEMI.(6) Despite this, older patients were less likely to receive pharmacological therapies such as aspirin, statins, beta-blockers and angiotensin-converting enzyme inhibitors, and less likely to undergo invasive investigation and treatment such as angiography and PCI.(7, 8) Rates of major bleeding were more than twice as high in patients aged ≥85 years compared to <65 years (p<0.0001). Each 10-year increase in age resulted in 75% increase in in-hospital mortality.(9) Despite the fact that older patients constitute a significant proportion (30-40%) of the NSTEMI population, and have the greatest potential to benefit from intervention, older patients are under-represented and often excluded from clinical trials.(10, 11)

Older patients presenting with acute coronary syndrome (ACS) are at higher risk of poor outcomes even after adjustment for confounding factors, such as co-morbidities.(12) However, the rate of invasive angiography in patients with non-ST segment elevation ACS (NSTEACS) declines with age.(13) This has led to an apparent treatment paradox, whereby the highest risk patients are the least likely to undergo invasive management despite having the most potential to gain from it.(14)

Frailty in Patients with NSTEMI

Frailty is often cited as a reason not to undertake invasive coronary angiography. It can be evaluated using the Fried and Rockwood scores. Fried frailty score requires three or more of the following criteria to be present: unintentional weight loss (10 pounds in past year), self-reported exhaustion, weakness (grip strength), slow walking speed, and low physical activity.(15) Rockwood frailty score(16) consists of 7 categories: **(1) Very fit:** Robust, active, energetic, well-motivated and fit; these people commonly exercise regularly and are in the most fit group for their age. **(2) Well:** Without active disease, but less fit than people in category 1. **(3) Well**: With treated co-morbid disease - disease symptoms are well controlled compared with those in category 4. **(4) Apparently vulnerable**: Although not frankly dependent, these people commonly complain of being "slowed up" or have disease symptoms. **(5) Mildly frail:** With limited dependence on others for instrumental activities of daily living. **(6) Moderately frail**: Help is needed with both instrumental and non-instrumental activities of daily living. **(7) Severely frail**: Completely dependent on others for the activities of daily living, or terminally ill.

Approximately one half of all patients hospitalised with NSTEMI who are ≥75 years of age are frail by the Rockwood 7-point frailty criteria.(16) Frailty is independently associated with increased 1-year mortality (frail: 49% vs. non-frail: 13%) after adjusting for cardiovascular risk and co-morbid conditions in the setting of NSTEMI (HR 4.3; 95% CI 2.4-7.8).(17) However, fewer frail older patients undergo coronary angiography compared to non-frail patients. In the study by Ekerstad et al, only 15% of older frail patients underwent coronary angiography and only 7% underwent a PCI procedure.(18) On the other hand, frailty is also independently associated with the risk of major adverse cardiovascular outcomes or complications (composite of death from any cause, MI, revascularization due to ischemia, hospitalization for any cause, major bleeding, stroke/transient ischemic attack, and need for dialysis) at 30-days (odds ratio [OR] 2.2; 95% CI 1.3-3.7).(18) Given the fact that fewer frail patients underwent coronary angiography compared to non-frail patients in previous studies, the risk-benefit of invasive procedures in these high-risk frail older patients presenting with NSTEMI remains unclear.(18)

Coronary Revascularisation in Frail Older Patients

To date, there has been only one published modest sized randomized controlled trial to determine the benefits of invasive treatment specifically in older patients presenting with NSTEMI and unstable angina: the After 80 study.[19] This Norwegian study randomized 457 patients to invasive or conservative strategies and demonstrated a halving in the composite of myocardial infarction, need for urgent revascularisation, stroke, and death (41% vs. 61%, p=0.0001). This reduction in events was predominantly driven by fewer recurrent myocardial infarctions and urgent coronary revascularisations. There were no major differences in the rates of stroke, death, or minor or major bleeding. However, the study recruited ~10% of potential patients, did not formally assess frailty and was underpowered to assess individual clinical endpoints or subgroups who may benefit most. Importantly, there were also no assessments of subsequent quality of life, frailty or living independence.

Other previous studies have been non-randomized retrospective sub-group analyses or in the setting of chronic stable angina.(20) Two published studies have thus far evaluated the benefit of invasive strategy versus conservative treatment in older (≥75 years) patients with ACS without stratification for frailty status and recruiting selected patient cohorts with no significant co-morbidities. In the first registry study, 1005 patients underwent coronary angiography and revascularisation, and 931 patients received conservative treatment. The mean age was 79 and 82 years ($p<0.0001$) respectively. In-hospital mortality and the combined endpoint of death or non-fatal re-infarction were lower in patients undergoing invasive management compared to those being managed conservatively (6.0% vs. 12.5%, $p<0.0001$ and 9.6% vs. 17.3%, $p<0.0001$ respectively). There was a marked reduction in one-year mortality in the invasive treatment group compared to conservative treatment group (OR 0.56; 95% CI 0.38-0.81).(21) The second study randomized 313 patients ≥75 years old with NSTEMI or unstable angina to an early invasive approach (n=154) or initial conservative approach (n=159).(22) In the early invasive treatment group, 88% of patients underwent coronary angiography and 55% had PCI, and in the initial conservative group it was 29% and 23% respectively. In patients with NSTEMI, there was a reduction in the primary endpoint (composite of death, MI, stroke and rehospitalisation for CV causes) in the early invasive treatment group compared to conservative approach at 1 year (HR 0.43; 95% CI 0.23-0.80, Log rank $p<0.05$). There was no difference noted between the two groups with unstable angina.

Evidence from clinical trials to inform the management of ACS in older patients is limited. We have reviewed this topic in detail in previous publications.(19, 23) More than half of all trials for coronary disease in the past decade failed to enrol patients ≥75 years of age, with this subgroup accounting for just 9% of all patients enrolled in trials.(24) Evidence-based recommendations from trials do not account for the age-related differences in physiology and disease that may alter these relationships. The age gap between trial and community populations begins at age 75 years and widens with age.(10) Even the older patients included in trials are different from the older patients in the community. Trial populations have lower rates of traditional cardiovascular risk factors, less co-morbidity and better renal function in each age subgroup than do community populations.(25) As older patients are at increased risk from cardiac events, the absolute benefit of treatment should increase if treatment risks can be balanced against benefits.(26) Risks and benefits derived from trials cannot always be extrapolated to older patients in daily clinical practice due to the differences between the patient groups.(27) More specifically, previous studies did not stratify patients based on frailty status and have not evaluated the risk-benefit of revascularisation in this cohort. The only published previous randomized controlled trials of older patients(22, 28) did not account for frailty status, included patients without NSTEMI and had limited power to draw definitive conclusions. Moreover, the mortality and composite ischaemic endpoint rates were low in the Italian study suggesting inclusion of highly selected low-risk older patients who were not frail.

Whether the beneficial effects of revascularisation will be demonstrated in older patients with co-morbidities presenting with NSTEMI is unknown and will be investigated in the present study. This is particularly important as this potentially frail older cohort is becoming more prevalent. SENIOR-RITA specifically aims to investigate these high-risk co-morbid frail

older patients who according to our national survey (responded to by 100 Cardiologists throughout the UK) are being denied advanced cardiovascular care due to fear of complications and perception of futility. We will utilise frailty score to identify, to characterise and to investigate at-risk older patients presenting with NSTEMI. We will also include all-comer older patients with NSTEMI including those with co-morbidities and those with cognitive impairment who normally would be denied invasive care due to underlying co-morbidity and in whom there is lack of evidence currently in the management of NSTEMI. Previous studies have shown that older patients undergoing PCI experience significant improvements in health-rated QOL.(29, 30) We will assess whether any gains in quality of life represent best use of NHS resources by conducting an economic evaluation of coronary revascularisation compared with conservative management in this population.

2. OBJECTIVES AND OUTCOME MEASURES

2.1. Primary Objective

To determine the impact of a routine invasive strategy on cardiovascular death and non-fatal myocardial infarction (MI) compared with a conservative treatment strategy in older patients (≥75 years) with NSTEMI.

2.2. Secondary Objectives

To determine the impact of a routine invasive strategy compared with a conservative strategy on:

- All-cause death
- Cardiovascular and non-cardiovascular death
- Recurrent myocardial infarction
- Coronary angiography and coronary revascularisation
- Hospitalization for heart failure
- Stroke
- TIA
- Bleeding (BARC ≥2)
- Procedural and in-hospital complications
- Length of time spent at home
- Frailty and quality of life
- Cost-effectiveness

2.3. Outcome Measures

In this randomized controlled trial, we will determine the risks and benefits of invasive versus conservative management on the following variables in older patients presenting with NSTEMI:

2.3.1. Primary Outcome Measure

Time to cardiovascular death or non-fatal MI (defined by the fourth universal definition(31) [see Section 16.1])

2.3.2. Secondary Outcome Measures

- All-cause, cardiovascular and non-cardiovascular death rates
- Recurrent myocardial infarction
- Hospitalisation for heart failure
- Coronary angiography and coronary revascularisation
- Stroke
- TIA
- Bleeding (BARC ≥2)
- Procedural complications (including death, MI, major bleeding (BARC definition), ≥25% increase in serum creatinine concentration from baseline, need for renal replacement therapy, stroke)
- Length of time spent at home
- Fried and Rockwood frailty scores
- Quality of Life using EQ-5D-5L and quality adjusted life years (QALY)
- Costs to the NHS and personal social services
- Incremental cost per QALY gained

3. TRIAL DESIGN

SENIOR-RITA is a multicentre prospective open-label trial randomising patients presenting with type 1 NSTEMI aged ≥75 years between invasive and conservative treatment strategies, to compare time to cardiovascular death or non-fatal MI. Patients will be followed up for at least one year after the last patient is recruited.

The trial flow chart is shown in **Figure 1.**

Figure 1: Trial Flow diagram

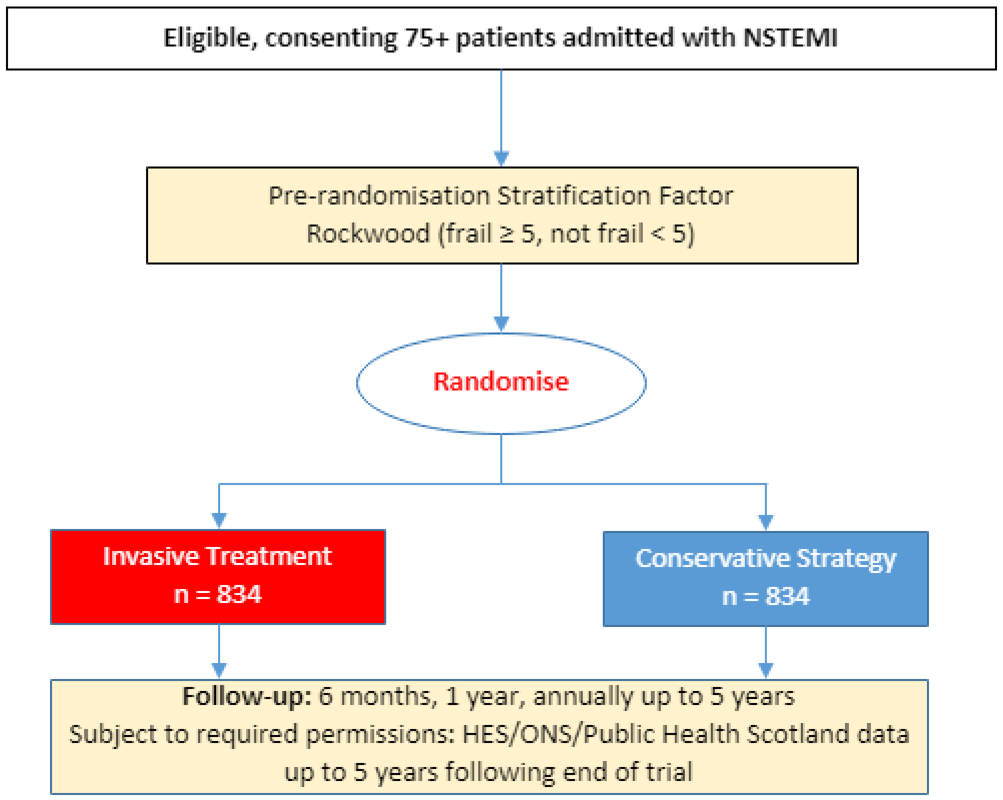

4. STUDY SETTING

This will be a broad and inclusive trial that will include participation of frail older patients with co-morbidities. Patients will be recruited throughout the UK. Patients will be recruited from centres with both on and offsite cardiac catheterisation facilities. Those patients randomized to invasive management in centres without cardiac catheterisation facilities will be transferred to a tertiary care centre within a recommended time frame of 7 days of randomization for angiography with a view to revascularisation. The study will be included as a UK Clinical Research Network study on the Central Portfolio Management System (CPMS) as a 'Portfolio' study. This will provide access to NIHR Clinical Research Network (CRN) service support and infrastructure in England. Inclusion on the Portfolio will also facilitate working with the Scottish Clinical Research Network and Health and Care Research Networks in Wales.

5. ELIGIBILITY CRITERIA

5.1. Inclusion Criteria

- Aged ≥75 years
- Type 1 NSTEMI during index hospitalisation

5.2. Exclusion Criteria

- Patients presenting with STEMI or unstable angina
- Patients with cardiogenic shock
- Patients with known life expectancy <1 year
- Patients in whom neither the patient nor the consultee are able and willing to provide written informed consent
- Previous inclusion in the BHF SENIOR-RITA trial
- Inability to undergo invasive coronary angiography, such as no vascular access site, or absolute contraindication to coronary revascularisation

6. TRIAL PROCEDURES

6.1. Patient Identification and Screening

Patients will be identified at tertiary centres and district hospitals based on eligibility criteria. Given the study will be on the NIHR portfolio, local research teams at individual sites that have agreed to participate in the study will screen for ≥75 year old patients with type 1 NSTEMI based on history (cardiac sounding chest pain), ECG changes (ST-T changes) and troponin elevation.

Screening data will be collected for all patients meeting the inclusion criteria and will be captured in the study screening logs at all participating centres. Each person eligible for SENIOR-RITA, whether or not recruited, must be recorded on the MACRO Screening log https://macro.infermed.com/NewcastleCTU/. The screening log will capture patient's age, gender, eligibility and recruitment status. For those patients who are screened but not recruited, the reason for non-participation, treatment strategy received and vital status (alive or dead) during index hospitalisation will be captured, subject to Health Research Authority (HRA) Confidentiality Advisory Group (CAG) permissions (England and Wales) and Public Benefit and Privacy Panel for Health and Social Care (PBPP) permissions (Scotland). Screening numbers are 6 digits and in the following format: the first two digits being site number and the next four digits following sequentially starting with 0001. Recruited participants' NHS/CHI numbers will be captured in the screening log.

6.2. Consent

Patients with ability to provide informed consent: Patients will be provided with the SENIOR-RITA patient information sheet to read. Written informed consent will be obtained from patients with capacity. Patients who have capacity to consent but are unable to write will provide oral consent in the presence of an independent witness who will initial, sign and date the consent form on their behalf. Two copies of the consent form will be produced: one will be filed in the patient's medical record and one will be given to the patient. The original will be filed in the Investigator Site File. The consent form will be securely faxed or emailed to NCTU for central monitoring of consent.

Should the patient lose capacity during follow up, the research team will continue to collect only routine data and the proxy EQ-5D-5L questionnaires to be completed by the relative or carer.

Patients lacking capacity to provide informed consent: Some patients with cognitive impairment will be able to provide informed consent. In patients who lack the capacity to provide informed consent, we will identify and approach a consultee as defined by the Mental Capacity Act (2005) England and Wales and subsequent amendments. In England and Wales, the consultee will be provided with the PIS and an information sheet for consultees and where appropriate, asked to sign the consultee declaration form for the patient's participation in SENIOR-RITA. In Scotland only patients with mental capacity will be identified and approached to participate in the study. Two copies of the informed consent form will be produced and the original filed in the

Investigator Site File. One copy will be filed in the patient's medical record and one will be given to the patient/consultee. The form will be securely faxed or emailed to NCTU for central monitoring purposes.

6.3. Randomisation

Following consent, patients will be randomised to an invasive strategy (coronary angiography and, if appropriate, coronary revascularisation [PCI or CABG] plus optimal medical therapy) or to conservative management (optimal medical therapy alone). Randomization will be on 1:1 per patient basis using a variable-length block stratified method specified by the statistics team. Stratification will be by site and Frailty score: frail (Rockwood Frailty Score ≥5) and not frail (Rockwood Frailty Score <5) (**Appendix 16.4)**

Randomisation will be performed at site using a secure web-based system. This system will produce a randomisation number which will need to be recorded on MACRO. Sites must complete the Patient Enrolment log and Patient Identification Code List.

For each randomised participant, the following information will be collected:

- Participant or Consultee Consent form
- Eligibility criteria form

The above documents should be transmitted by secure fax to the NCTU on 0191 5800987 (SoHo 66), or by email to nctu.seniorrita.conf@nhs.net using an NHS.net email. If the site does not use nhs.net email, the documents should be sent as an encrypted, password-protected file (with the password sent in a separate email) to seniorrita.support@newcastle.ac.uk.

6.4. Data collection

See schedule of events.

Baseline data

All baseline data required for the study including baseline demographics, medical history/co-morbidities (Charlson Index), risk factors, current admission details and discharge medications will be collected during hospitalisation and recorded on the electronic CRF. Results of investigations performed as part of routine care of patients including full blood count, serum urea, creatinine concentrations, lipids, glucose and peak troponin concentration will be recorded. Data from baseline ECG will be collected for information on ST-T changes. Study related data including MoCA scores, frailty scores, quality of life (EQ-5D-5L), use of health services and patient costs will also be collected. Echocardiographic data will be collected where available.

Angiographic and procedural data will be collected from participants that are randomised to invasive care. Sites will be asked to save to CD the anonymised angiography and angioplasty images of participants' index procedures. The CDs will be supplied to them by Newcastle CTU,

and sites will be asked to return them for analysis. Participants' full angiography and angioplasty image data will be identified by their SENIOR-RITA randomisation number.

5-Year follow-up: 6-Month, 1-year follow up and annually up to 5 years

Participants should be encouraged to attend in person for each of these follow-up appointments. However, due to the nature of this patient population, this will not always be possible. Where it is not possible for the participant to attend a research appointment the research nurse should contact the participant (or carer) by phone to obtain as much of the follow up information as possible including the completion of the relevant questionnaires and the identification of any complications or clinical endpoints. If it is not possible to contact the patient (or carer) by phone, then the questionnaires should be sent by post from the site. These should be returned to the site and recorded in the eCRF. If follow-up is carried out by telephone or post, it will not be possible to perform full frailty assessments. At each of these assessment points the research nurse will be required to check for any complications or clinical endpoints in the participants' medical records or by contacting the GP.

The duration of the follow up period for participants is dependent upon when they were recruited into the study. All participants will be followed up until one year after the last patient is recruited, up to a maximum of 5 years.

Frailty Scores: Rockwood Scores must be calculated for all patients prior to randomisation as this is used to stratify recruitment. The Fried Frailty Score should also be completed at baseline, and both the Rockwood and Fried frailty scores should be completed where possible at 6 months and annually up to 5 years. Completed Fried Frailty score and Rockwood frailty score worksheets must be filed in the patient notes labelled with the randomisation number and the date of completion.

Cognitive impairment: The MoCA should be completed for all participants at baseline and 1 year. Completed MoCA score must be filed in patient notes.

Quality of Life: Quality of life (QOL) assessments using the EQ-5D-5L will be requested at baseline (during hospitalisation), 6 months, and at 1 year for all participants, and then annually up to 5 years as applicable. Further long-term follow-up beyond 5 years using ONS/HES/PHS data may be considered subject to additional funding and CAG/PBPP approvals. Data will be collected during research clinic follow-up visits where feasible. Where this is not feasible, patients will be given the questionnaires to take home at the appropriate trial visits or questionnaires will be posted out to patients. Patients will be asked to complete the questionnaires at home after the trial visit and return the completed questionnaires back to the trial office. If questionnaires cannot be returned by post or are not returned after 2 weeks, data will be collected by telephone interviews with the patient or with their carer, where possible.

Given the increasing age of this population over the course of the trial, it is important to consider those who may struggle to self-complete quality of life instruments (e.g. due to pre-existing cognitive impairment or onset during the course of the trial). In addition, for the EQ-5D-5L, a proxy-rated version is available for relatives/carers to rate how they expect the

patient views their current quality of life. We will strive to collect self-reported quality of life data where possible. It will not be possible at baseline to predict the patients who will go on to develop dementia during the course of the trial, and within this group, who will deteriorate to the extent that it will become no longer possible to collect self-reported quality of life data. Thus, whilst not mandatory to the trial, all participants regardless of their capacity should be approached to ask a relative or carer to complete the Proxy EQ-5D-5L questionnaire during the index visit. Thereafter the relative or carer will only be asked to complete a proxy EQ-5D-5L if the patient is no longer able to self-report.

Relative or carer contact form to be completed and signed before obtaining proxy questionnaire.

Health and personal social service resource use and patient costs: As part of our assessment of endpoints, we will quantify the cost and treatment in both trial arms by collecting data on the use of secondary, primary and personal social services (PSS) at baseline (and at each follow-up period) in a bespoke health and PSS utilisation questionnaire. These data will be combined with unit costs (obtained from routine sources or from study specific estimates) to calculate costs (see Section 10).

Medical events/outcomes: All instances of the following clinical outcomes which occur following randomisation up to 5 years should be obtained from medical records/GP records and reported on the appropriate pages of the eCRF:

- Death
- Recurrent myocardial infarction
- Stroke
- TIA
- Bleeding (BARC≥2)
- Coronary angiography and coronary revascularisation
- Hospitalisation for heart failure

6.5. Long-term follow-up

Subject to additional funding, follow-up information will be gathered from ONS, HES data (England) and PHS data (Scotland) health records for 5 years after the end of the study period. Patient (or consultee) consent for this level of data access will be sought. Further long-term follow-up beyond 5 years using ONS/HES/PHS data may be considered subject to additional funding and CAG (England and Wales)/PBPP (Scotland) approvals.

6.6. Schedule of events

	During Hospitalisation						
	V1	V2	V3	V4	V5	V6	V7
**Event**	**In-patient with NSTEMI**	**6-months ±28 days**	**1 year ±28 days**	**2 year ± 56 days**	**3 year ±56 days**	**4 year ± 56 days**	**5 year ± 56 days**
**Eligibility**	X						
**Informed Consent**	X						
**Rockwood Frailty Score**	Required pre-randomisation	X	X	X	X	X	X
**Fried Frailty Score**	X	X	X	X	X	X	X
**Montreal Cognitive Assessment**	X		X				
**Randomise (Web)**	X						
**Patient Demographics, Baseline Admission Data**	X						
**Medical History, Risk Factors, Co-morbidities**	X						
**Baseline ECG**	X						
**Baseline Bloods (FBC, urea, creatinine, glucose, lipids*, peak troponin)**	X						
**Echocardiography***	X						
**Angiography/Procedural Data**	X						
**Concomitant Medications**	X	X	X				
**Evaluate Endpoints[1] Either Clinic (hospital or GP records) or Post or Telephone or ONS, HES, PHS data**	X	X	X	X	X	X	X
**EQ-5D-5L Patient or Proxy[2]**	X	X	X	X	X	X	X
**NHS & PSS Utilisation† (Resource Use Questionnaire)**	X†	X†	X†	X‡	X‡	X‡	X‡
**Time and Travel Questions**		X					

† 6-month recall period
‡ 12-month recall period
PSS - Personal social services
ONS - Office of National Statistics
HES - Hospital Episode Statistics
PHS – Public Health Scotland

*This information is collected where available from routine clinical practice

[1] Recurrent MI, Hospitalisation for heart failure, coronary revascularisation and coronary angiography, Stroke, TIA Bleeding BARC ≥ 2, Death
[2] Participant and relative or carer to be approached to complete EQ-5D-5L Self Complete and proxy EQ-5D-5L at baseline and follow-ups.

6.7 Withdrawal Criteria

Participants have the right to withdraw from the trial at any time without having to give a reason. Investigator sites should try to ascertain the reason for withdrawal and document this reason within the Case Report Form and participant's medical notes. Participants may:

1.Withdraw from the trial with no further contact from the study team but allow the continued use of routinely collected data. Routinely collected data includes hospital medical notes, GP records, ONS (Office of National Statistics), HES (Health Episode Statistics) or PHS (Public Health Scotland) data. 2. Withdraw from any further involvement in the trial and not allow the continued use of routinely collected data

- As follow-up for clinical events in this study is critical, all participants who no longer wish to take part in the study will be encouraged to have follow-up data collected as per point 1.
- A withdrawal form will need to be reviewed and signed off by the Principal Investigator.
- If the participant is unable to sign off the form themselves, it should detail how the withdrawal was communicated to the site team.
- In all withdrawals data collected up to the point of withdrawal of consent will be retained and used in analysis

Participants may not receive their allocated trial treatment due to patient preference or clinician decision. Participants may also not prefer to attend clinic follow-up visits due to personal or social reasons. This is not considered a withdrawal from the trial. All such participants will continue to be followed up as normal as per protocol.

As the trial will not be approaching patients lacking capacity in Scotland, should patients randomised to the study in Scotland lose capacity, only routine data regarding hospital episodes and death data will be captured for these participants. Scottish centres can obtain these data through PHS data review.

6.8 End of Trial

Participants will be followed up for at least one year after the last patient is recruited. The primary analysis will take place once all participants have been followed for at least one year. Direct participant contact for the purposes of the trial will cease after their 5 year follow up.

Beyond this, longer term outcome data, up to 10 years post randomisation, may be obtained from electronic health records data (ONS, HES, PHS) subject to additional funding and approvals. Participant consent for this level of data collection will be obtained at baseline, however HRA Confidential Advisory Group (CAG) and Scottish PBPP approvals will be sought where appropriate.

Participants will continue to receive care that is offered through the NHS for ongoing care of these patients.

7. TRIAL INTERVENTIONS

7.1. Treatment Strategy

Invasive coronary angiography ± coronary revascularisation plus optimal medical therapy versus optimal medical therapy alone. Coronary angiography will be performed as per local practice patterns. Based on angiographic findings, revascularisation by PCI or CABG will be performed at the discretion of attending Cardiologist and the multidisciplinary team.

7.1.1. Schedule & Modifications

All invasive procedures will be performed according to local hospital protocols. It is recommended that patients undergo coronary angiography with a view to revascularisation (PCI/CABG) within 3-7 days after randomization and during their index hospitalisation. Where possible, coronary revascularisation should be completed within 7 days or as soon as practically possible.

7.1.2. Known Risks

Invasive coronary angiography, percutaneous coronary intervention and coronary artery bypass graft surgery are established treatment strategies for the management of coronary artery disease. The coronary angiography and coronary angioplasty procedures use x-rays, so there is a small risk from the exposure to this radiation. The amount of radiation involved is equivalent to less than three years of exposure to the average natural background radiation in the UK. Risks and benefits that are quoted during normal routine consent procedures are applicable to patients that will be recruited into the SENIOR-RITA trial. In addition, the procedure-related risk of in-hospital death (<1%), myocardial infarction (<1%), stroke (<1%), renal replacement therapy (<1%) and any bleeding (2%) will be quoted to the patients.

7.2. Concomitant Medications & Therapies

Optimal Contemporary Medical Therapy: In the absence of contraindications, all patients will receive aspirin 75 mg once daily; P2Y12 receptor antagonist in line with European Society of Cardiology guidelines; statin therapy, a beta-blocker (to target a heart rate of 60-70 beats per minute); an ACE inhibitor or ARB. If the patient continues to experience hypertension with blood pressure >140/85 mmHg, additional blood pressure lowering treatments will be considered at the discretion of the attending cardiologist and as tolerated by the patient. If the patient has marked left ventricular dysfunction (left ventricular ejection fraction <40%), an aldosterone antagonist will be considered. If the patient is diabetic, the goal will be to maintain fasting blood glucose levels between 4.4-7.5 mmol/L and haemoglobin A1c levels <7.0%, in accordance with published recommendations of the American Diabetes Association and the DCCT Consensus Report. In patients with poorly controlled glucose levels, insulin will be strongly considered.

8. SAFETY REPORTING

8.1. Standard Definitions

Term	Definition
**Adverse Event (AE)**	Any untoward medical occurrence in a participant, including occurrences which are not necessarily caused by or related to the intervention under study. Medical conditions/diseases present before starting study treatment are only considered AEs if they worsen after starting study treatment.
**Adverse Reaction (AR)**	An untoward or unintended response in a participant to which is related to the intervention under study i.e. that a causal relationship between the trial intervention and an AE is at least a reasonable possibility and the relationship cannot be ruled out. All cases judged by either the reporting medically qualified professional or the Sponsor as having a reasonable suspected causal relationship to the trial intervention qualify as adverse reactions.
**Unexpected Adverse Reaction:**	An adverse reaction, the nature and severity of which is not consistent with the information about the intervention under study.
**Serious Adverse Event (SAE)**	A serious adverse event is any untoward medical occurrence that:<br>• Results in death<br>• Is life-threatening*<br>• Requires inpatient hospitalisation or prolongation of existing hospitalisation<br>• Results in persistent or significant disability/incapacity<br>• Consists of a congenital anomaly or birth defect<br>• Other important medical events that jeopardise the participant or require intervention to prevent one of the above consequences<br>*Life-threatening refers to an event in which the participant was at immediate risk of death at the time of the event; it does not refer to an event which hypothetically might have caused death if it were more severe.
**Serious Adverse Reaction (SAR)**	An adverse event that is both serious and, in the opinion of the reporting Investigator, believed with reasonable probability to be due to the trial intervention, based upon the information provided.
**Unexpected Serious Adverse Reaction (USAR)**	A serious adverse reaction, the nature and severity of which is not consistent with the known information about the intervention under study.

8.2. Severity (Intensity) of Serious Adverse Events and Reactions

Severity of all SAEs and SARs will be graded on a three-point scale of intensity (mild, moderate, severe):

- **Mild:** Discomfort is noticed, but there is no disruption of normal daily activities
- **Moderate:** Discomfort is sufficient to reduce or affect normal daily activities
- **Severe:** Discomfort is incapacitating, with inability to work or to perform normal daily activities

8.3. Assessment of Causality

Each AE should be clinically assessed for causality based on the information available, i.e., the relationship of the event to the intervention under study should be established. The assignment of causality will be made by the Principal Investigator responsible for the care of the participant using the definitions in the table below. All serious adverse events judged by the local investigator as having a reasonable suspected causal relationship to the intervention under study (i.e. definitely, probably or possibly related) are considered to be serious adverse reactions. All SAE's will be reviewed for causality by the Chief Investigator. In the event of a discrepancy between PI and CI assessment of causality, the Clinical Endpoints Committee (CEC) will adjudicate and make the final decision.

Relationship	Description
**Unrelated**	There is no evidence of any causal relationship. The clinical event has an incompatible time relationship to the intervention under study, and could be explained by underlying disease, or other drugs or chemicals.
**Unlikely**	There is little evidence to suggest there is a causal relationship (e.g. the event did not occur within a reasonable time after the intervention under study). There is another reasonable explanation for the event (e.g. the participant's clinical condition).
**Possible**	There is some evidence to suggest a causal relationship (e.g. the event occurs within a reasonable time after the intervention under study). However, the influence of other factors may have contributed to the event (e.g. the participant's clinical condition).
**Probable**	There is evidence to suggest a causal relationship, including a reasonable time relationship with the intervention under study, and the influence of other factors is unlikely.
**Definitely**	There is clear evidence to suggest a causal relationship and other possible contributing factors can be ruled out.
**Not assessable**	There is insufficient or incomplete evidence to make a clinical judgement of the casual relationship.

8.4. Recording and Reporting AEs and SAEs

Due to the nature of the study population (high risk older patients with multiple co-morbidities) only serious adverse events which are causally (possible, probable or definitely) related to the study intervention (coronary angiography and/or PCI) occurring within 7 days of the procedure will be reported in patients randomised to the invasive arm only.

Events occurring in participants randomised to the optimal medical therapy arm will not be reported as SAEs but will be recorded as an outcome measure in the ECRF.

Complications associated with CABG will not be reported as SAEs.

Events occurring in patients randomised to the optimal medical therapy arm subsequently undergoing angiography procedure will also be not reported as SAEs.

Some related serious adverse events occurring in this trial will be expected as a consequence of the underlying disease, study related investigational procedures or routine treatments/diagnostic tests. Expected serious adverse events are listed below. Should any of these events occur within 7 days of the invasive procedure they are to be captured on the relevant eCRFs and are required to undergo separate expedited reporting as an SAE (see figure below).

Expected serious adverse events within 7 days of angiography and/or PCI:

- Death
- Myocardial Infarction
- Minor (BARC<2) and major bleeding (BARC ≥2)
- >25% increase in serum creatinine concentration from baseline
- Need for renal replacement therapy
- Coronary Dissection
- Aortic Dissection
- Perforation
- Stroke
- TIA
- Cardiac tamponade
- Emergency repeat angiography or PCI procedure
- Procedure related pulmonary oedema
- Vascular complications needing intervention

Events which meet the definition of medical events/outcomes as per Section 6.4 should also be reported on the appropriate pages of the eCRF as a clinical endpoint.

All unexpected, related serious adverse events undergo expedited reporting by submitting an SAE form to the NCTU within 24 hours of the site becoming aware of the incident. The initial report can be made by secure fax or password encrypted

email. In the case of incomplete information at the time of initial reporting, all appropriate information should be provided as follow-up as soon as this becomes available. For each event the following information will be collected:

- Full details in medical terms and case description
- Event duration (start and end dates, if applicable)
- Action taken
- Outcome
- Seriousness criteria
- Causality in the opinion of the investigator
- Whether the event is considered expected or unexpected

The SAE form should be transmitted by fax to the NCTU on 0191 5800866 (SoHo 66).

Should participating centres not have access to a fax machine, these documents will need to be sent as a password encrypted file to soho66seniorrita@newcastle.ac.uk.

Unrelated AEs and SAEs e.g. pneumonia, cancer, liver failure, knee and/or hip replacements will not be collected. Should there be any queries from site regarding the reporting requirements for SAEs, the site will need to contact the NCTU who will seek clarification from the Chief Investigator.

In the case of incomplete information at the time of initial reporting, all appropriate information should be provided as follow-up as soon as this becomes available. Relationship of the SAE to trial participation should be assessed by the Principal Investigator at site, as should the expected or unexpected nature of the AE. The trial Chief Investigator will review all SAEs to provide their assessment of the events reported by the site.

For the purposes of this protocol

- SAEs exclude any pre-planned hospitalisations not associated with clinical deterioration.
- SAEs exclude routine treatment or monitoring of the studied indication, not associated with any deterioration in condition.
- Cardiovascular death and other events that are primary or secondary outcome measures are not considered to be SAEs if they occur more than seven days after the trial intervention (angiography/PCI) and should be reported in the normal way on the appropriate CRF.

The main REC will be notified by NCTU (on behalf of the Sponsor) of all USARs within 15 days of NCTU becoming aware of the USAR. USARs will be reported by email to REC using the Non-CTIMP Safety Report to REC form, and a copy of the report filed in the Trial Master File. The NCTU will ensure that The Newcastle upon Tyne Hospitals NHS Foundation Trust as Sponsor is notified of any USARs in accordance with local trust policy. Local principal investigators should report any USARs as required by their local Research & Development Office.

Safety Reporting Diagram
A flowchart is given below to aid reporting procedures

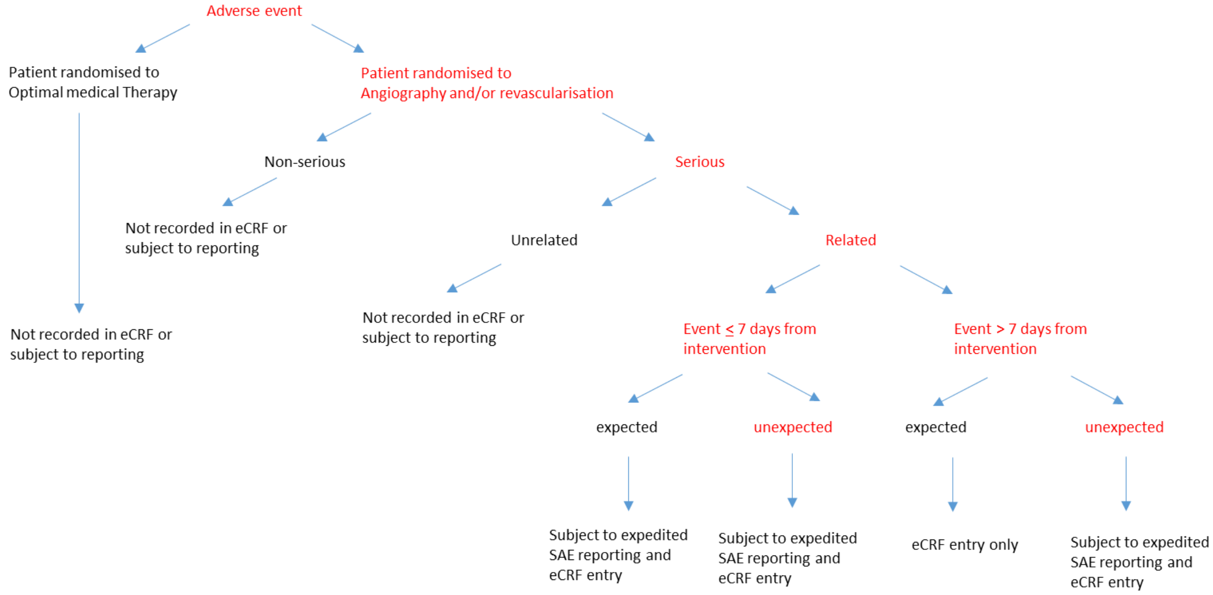

Note – Recording in eCRF is only required for Adverse Events that meet Clinical End Point definition (see section 6.4)

8.5. Responsibilities

Principal Investigator

- Checking for related AEs when participants attend for treatment or follow-up
- Using medical judgement in assigning seriousness and causality and providing an opinion on expectedness of events
- Ensuring that all SAEs occurring within 7 days of the intervention, including USARs, are recorded and reported to the Sponsor's representative (NCTU) within 24 hours of becoming aware of the event and provide further follow-up information as soon as available

Chief Investigator

- Clinical oversight of the safety of trial participants, including an ongoing review of the risk/benefit profile
- Using medical judgement in assigning seriousness, causality and expectedness of SAEs where it has not been possible to obtain local medical assessment.
- Using medical judgement in assigning expectedness to SARs.
- Immediate review of all USARs
- Review of specific SAEs and SARs in accordance with the trial risk assessment and protocol

Sponsor

- Assessment of expectedness of any USARs
- Expedited reporting of USARs to the REC within required timelines
- Notification of all investigator sites of any USAR that occurs

8.6. Reporting Urgent Safety Measures

An Urgent Safety Measure (USM) is an action that the Sponsor or an Investigator may take in order to protect the subjects of a trial against any immediate hazard to their health or safety. Upon implementation of an USM by an Investigator, the Sponsor must be notified immediately and details of the USM given. The Sponsor must inform the NHS REC within 3 days of the USM taking place in accordance with the Sponsor's standard operating procedures.

9. STATISTICAL CONSIDERATIONS

9.1. Analysis Populations

All data from trial participants will be included in the primary analysis which will be conducted on an intention to treat (ITT) basis. Sensitivity analyses may be conducted on a per-protocol subset, which, if used will be pre-specified in the statistical analysis plan and agreed by a statistician blind to treatment allocation. Intervention related SAEs will be reported for those randomised to the invasive strategy and undergoing their randomised angiography and intervention procedure. All statistical analyses will follow a fully detailed predefined statistical analysis plan, written prior to any comparative interim analysis (i.e. for DMC reporting).

9.2. Statistical Analyses

9.2.1. Analyses of the Primary Outcome Measure

The primary outcome is time to cardiovascular death or non-fatal MI as time to first event of a composite outcome. Analyses will utilise all data collected. Participants alive and free from recurrent MI will be censored in the analysis at the time last seen alive and MI free or time of non-CV death. Event-free rates will be estimated using the method of Kaplan and Meier, and reported as 6-month and 1-year CV death and non-fatal MI composite rates. Event-free estimates will be statistically compared using a non-parametric stratified log-rank test (based on stratification factor of level of frailty at randomization). An adjusted Cox proportional hazards regression model (adjusted for the stratification factor of level of frailty at randomization) will be used to estimate the treatment effect. The hazard ratio (HR) will be reported along with a 95% confidence interval). A sensitivity analysis of the primary outcome will also adjust for other demographic (e.g. sex, age) and clinical covariates including baseline disease severity, co-morbidities and cardiovascular risk factors. The proportional hazards assumption of the Cox model will be investigated and alternative methods such as flexible parametric survival models used if the assumptions are not found to hold. Competing risks analyses will be carried out and reported based on time to event analysis of the composite (CV death and non-fatal MI) given patients may experience a non-CV death which would prevent further outcome events of interest.

9.2.2. Analyses of Secondary Outcome Measures

Time to Event: Analyses of secondary time to event outcomes will be analysed and reported using similar Kaplan Meier time to event analyses based on all-cause, cardiovascular and non-cardiovascular deaths. HRs of the risk of invasive treatment

compared with conservative therapy, adjusted for the stratification factor for level of frailty at randomisation, will be reported (with 95% confidence intervals).

Numbers and rates of recurrent myocardial infarction, hospitalisation (for heart failure), stroke, TIA, coronary angiography and coronary revascularisation and length of time spent at home will be reported descriptively.

Safety data: Comprising the numbers and rates of procedural complications (e.g. death, MI, major bleeding including gastrointestinal and intracranial bleeding [BARC definition], ≥25% increase in creatinine from baseline, need for renal replacement therapy, incidence of stroke or TIA) will be reported descriptively as frequency and proportions of the total number randomised to the invasive strategy and undergoing their allocated procedure (with 95% confidence intervals). Missing data are expected to be minimal and will be reported.

Frailty Scores:
Scores will be calculated according to the developers' instructions and will be graphically summarised over time by randomized treatment group. Standardised area under the curve summary statistics will be used to report frailty scores over time conditional on survival and will be compared across treatment groups. The association between Rockwood and Fried frailty scores will be investigated using rank correlation.

9.2.3. Subgroup Analyses

Planned subgroup analysis will present hazard ratios for individual subgroups using Forest plots. Pre-planned sub-group analysis (according to a predefined statistical analysis plan) will be based on the following sub-groups:

- Frail vs. not frail patients (Rockwood score ≥5 vs. <5).
- Cognitively impaired vs. not impaired (Montreal Cognitive Assessment (MoCA) score <26 vs. ≥26).
- Frail and cognitively impaired patients vs. non-frail, non-cognitively impaired patients
- Co-morbid vs. non co-morbid patients

Hazard Ratios of the treatment effect will be calculated within identified subgroups and plotted on a Forest plot with associated 95% confidence intervals. Tests of heterogeneity of the difference in the estimated treatments effects across subgroups will be calculated and reported.

9.2.4. Planned Additional Analyses

Subject to additional funding time to event analyses will be reported for longer term follow up of patients to 5 and 10 years.

9.2.5. Interim Analyses and Criteria for the Premature Termination of the Trial

The trial will be monitored by an external Data Monitoring Committee (DMC) that will meet at the start of the trial and annually thereafter. Interim meetings may be convened should the DMC have any safety concerns. Accumulating patient data will be reported to the DMC, but interim analyses will not be undertaken until 50% of patients are recruited. The DMC will make recommendations to the Trial Steering

Committee as to whether to stop or continue recruitment based on all available evidence and with attention to the Peto-Haybittle boundary when interpreting significance levels, as specified in the statistical analysis plan and DMC charter.

9.3. Sample Size Calculations

Original sample size calculation

The original recruitment target for this trial was a total of 2300 patients (1150 in each arm).

In previous studies, the all-cause death rates in frail and non-frail patients presenting with NSTEMI was approximately 30% at one year,(17) which equates to an assumed 12-month 'event-free survival' rate on the conservative therapy arm of 70% at 12-months. In our trial, the estimated reduction in event rate due to including only CV deaths was anticipated to be counter balanced by an increase in event rate due to the inclusion of non-fatal MI. A clinically relevant effect size is assumed to be a 20% reduction in the overall event rate. To detect a clinically relevant reduction in cardiovascular death and MI from 30% to 24% equates to a clinically relevant increase in event free survival from 70% to 76% and a Hazard Ratio=0.77. To detect this increase in event free survival rate at 12 months, requires a minimum of 620 events to be observed for 90% power and 5% two-sided type I error rate. It was estimated that this trial would need to recruit 1149 patients to each randomised arm to observe this number of events with patients observed for 12 months. It was anticipated that events would be observed for at least 12 months and if 620 events had not been reached at 12 months this would be extended. With 2300 patients and 620 events, this trial would be able to reject the null hypothesis that the invasive treatment and conservative therapy survival estimates are equal with probability (power) of 90%, and associated type I error (false positive) probability (alpha) of 5%(32). The analysis of the primary outcome measure would have been conducted when the desired number of events had been reached.

If the true size of difference was smaller, similar to that found by Fox et al,(1) then the original trial sample size would have had 78% power to detect the HR=0.81 reported. MINAP database reported an incremental reduction from intensive management with increasing age.(33) The adjusted HR for all-cause mortality comparing invasive to conservative management was HR=0.53 (reported inversely as HR=1.90 95% CI 1.77, 2.04) in NSTEMI patients aged >85 years. If the true size of difference was larger, similar to that found by Zaman et al,[(33)] then this original trial sample size would have >99% power to detect a larger difference of HR=0.53.

Updated Sample size calculation

In late 2018, an assessment of study progress was made by the trial management group. It was observed that the recruitment rate was much lower than originally projected, at around 32 participants/month, and based on this recruitment rate the study would require an extension to recruitment of around 3.5 years in order to reach the target sample size of 2300. Furthermore, a blind review of available primary outcome data (unadjudicated) to December 2018 suggested the probability of an

event in the first year (aggregated across treatment groups) was lower than originally anticipated at approximately 0.2.

The original sample size calculation effectively assumes patients are only followed-up for 12 months. However, in reality, patients are followed up throughout the trial, not just to 12 months, and the analysis will make use of all data available on all patients. This allows events to accrue over a longer period, so fewer patients would need to be recruited to achieve the required number of events.

A more realistic approach to sample size estimation, as detailed in Barthel *et al(34),* allows events beyond one-year to be incorporated by assuming the event rate follows an exponential distribution. The recruitment rate across the trial can also be taken into account as this will inform the duration of follow up available for each patient at the time of the primary analysis. This approach can be implemented using the Stata function ARTSURV (version 1.1.0)(35).

Assuming an event rate of 20% in the conservative therapy arm at 12 months and maintaining the original clinically relevant effect size of a 20% reduction equates to an event rate of 16% in the invasive therapy arm and a HR of 0.78. To detect a HR of 0.78 with 90% power and 5% two-sided type I error requires around 700 events to be observed.

If recruitment is extended for 24 months (i.e. a total recruitment period of 5 years) and a recruitment rate of 32 patients/month can be maintained then the study will recruit 1668 patients and have 92.5% power (5% two-sided type I error) to observe a HR of 0.78 assuming an event rate in the control group of 0.2 with the analysis taking place 1 year after the last patient is recruited and utilising all available follow-up. This assumes a total of 752 events have been observed at the time of analysis. Further information and power calculations under alternative scenarios are given in Appendix 16.7.

Underlying assumptions pertaining to the sample size calculations, including overall numbers of CV deaths and non-fatal MI, will be monitored by the independent DMC.

10. ECONOMIC EVALUATION

10.1. Assessment of Cost

For the main analysis we will conduct a micro-costing exercise to estimate the cost of the interventions (costed on a per-patient basis), as well as the use of secondary, primary and personal social services (PSS) during the follow up period. This is because the perspective for the main analysis will be the NHS and PSS. However, we will conduct further analyses that take a societal perspective by incorporating the time and travel costs and out of pocket expenses for health care borne by patients and their families. Details of resources used and patient costs will be obtained from bespoke patient completed questionnaires. For resources used, this will be completed at each face to face data collection time period, using a modified version of the Client Service

Receipt Inventory (CSRI). The CSRI is a validated instrument designed to be adapted to the specific study population of interest(36, 37). Patient time and travel costs will be collected at one time point (six months). Unit costs will be based on nationally available data from routine sources and study-specific estimates. Where appropriate, discounting will be applied to costs and outcomes.

10.2. Economic Analysis

A cost-utility analysis will use the responses to the within-trial assessment of quality of life using the EQ-5D-5L, combined with each study participant's mortality to estimate QALYs. This measure provides a profile of quality of life over time. The results of the analyses will be presented as point estimates of mean incremental costs and QALYs. We will undertake sensitivity analyses to test key assumptions made during data collection, including for example, the accuracy of any proxy-reported EQ-5D-5L values. Techniques such as bootstrapping will be used alongside deterministic sensitivity analyses to address uncertainty, and will be presented as cost-effectiveness acceptability curves (CEACs) for each trial arm, for different levels of society's willingness-to-pay for a QALY. An economic model will then be developed to assess the cost and health consequences measured in terms of QALYs over the patients remaining lifetime. This will allow us to consider the longer-term effects of the treatments received. We will develop a range of plausible care pathways, using the literature and expert advice on the natural course of this illness for this population, along with expert advice on typical care routes. The level of detail involved in modelling care routes will to some extent depend on the extent of variation in the kinds of care being received. The data from the trial will be the main source of data for this model but further data with which to model outcomes beyond the follow-up will be systematically derived from the literature and other existing data sources following guidance for best practice.(38) These data will include information on factors such as the incidence of hospitalisation beyond the trial follow-up period. Sensitivity analysis will be applied to the model using probabilistic and deterministic sensitivity analyses to address parameter and other forms of uncertainty.

We will use data from the EQ-5D-5L collected at 1 month and 3 months on a sub-sample of trial participants to estimate the utility for all participants for the recovery period after initial intervention. Only a sub-sample of data is required in order to reduce response burden to trial participants and trial staff in each centre. We will identify from existing and possibly ongoing studies health related quality of life as measured by EQ-5D-5L instruments that have been collected over the course of one year following revascularisation interventions. If sufficient applicable data are available from the existing evidence base, we will seek to obtain the individual level data and information of the characteristics of the individuals revascularised and explore whether such data could be used to impute EQ-5D responses during the recovery period following revascularisation. If this is possible and can be completed in a timely manner, we will consider reducing the response burden for the EQ-5D-5L on participants in this trial.

11. DATA HANDLING

11.1. Data Collection Tools and Source Document Identification

All data for an individual patient will be collected by the PI or their delegated nominees and recorded in the web-based electronic case report form (CRF) for the study delivered via Elsevier's MACRO. All investigators and study teams will be provided with secure logins to access the ECRF to input patient data. Patient identification on the CRF will be through a unique study identifier number allocated by a web-based randomization system. The NCTU will audit completeness and quality of data recording in CRFs and will correspond regularly with the PI (or their delegated assistants) with the aim of capturing any missing data where possible, and ensuring continuous high quality of data.

11.2. Data Handling and Record Keeping

All data will be stored in password-protected databases. Any paper documents will be stored in study related locked filing cabinets. Clinical information will not be released without the written permission of the participant, except as necessary for monitoring and auditing by the Sponsor, its designee, Regulatory Authorities, the Trial Steering Committee (TSC), the Data Monitoring Committee (DMC) or the REC. Secure anonymized electronic data will be released to the Trial Statisticians for interim and final analyses. The PI and study site staff involved with this study may not disclose or use for any purpose other than performance of the study, any data, record, or other unpublished, confidential information disclosed to those individuals for the purpose of the study. Prior written agreement from the Sponsor or its designee must be obtained for the disclosure of any said confidential information to other parties. Data will be anonymised by coding in order to maintain confidentiality of the study participant, but the research team will be able to identify participants in the event of clinical need. Any data recorded electronically will be onto a password protected NHS computer in the research centre. Data received at NCTU will be processed as per the NCTU SOPs, including entering the data into a secure central database. Responsibility for maintenance of the database will rest with the NCTU.

11.3. Access to Data

The Investigators will have access to their own patient data during the trial. Accumulating trial data sets will be accessible by Chief Investigator and NCTU. The data may also be used to support students/Fellows wishing to undertake research projects and write up their dissertation. Data sharing will be in alignment with the Sponsor's data sharing SOP.

11.4 Archiving

The site will construct an Investigator Site File (ISF) and will maintain all study records according to GCP and the applicable regulatory requirements. On completion of the

trial the site ISF and study data can be archived for a minimum of 5 years following the end of the study, unless local arrangements require a longer period.

The trial master file (TMF) will be held by the NCTU and the essential documents that make up the file will be listed in a SOP. On completion of the trial, the TMF and study data will be archived by the Sponsor according to the applicable regulatory requirements and for up to 15 years.

12. MONITORING, AUDIT & INSPECTION

The trial may be subject to audit by representatives of the Sponsor or inspection by HRA. Each investigator site will permit trial-related monitoring, audits and regulatory inspection including access to essential and source data relating to the trial. The trial may be prematurely discontinued on the basis of new safety information, or for other reasons given by the Data Monitoring Committee (DMC) and/or Trial Management Group (TMG), Sponsor, regulatory authority or ethics committee concerned.

Monitoring, quality control and assurance: The trial will be managed by NCTU in collaboration with the CI and Co-PIs.

The Trial Management Group (TMG): The Principal Investigators will be responsible for the day-to-day study conduct at site. The NCTU trial management team will provide day-to-day support for the sites and will provide training through Investigator meetings, site initiation visits and routine monitoring visits. Quality control will be maintained through adherence to NCTU SOPs, study protocol, the principles of GCP, the Research Governance Framework for Health and Social Care, the European Directive (2001/20/EC), the Medicines for Human Use (Clinical Trials) Regulations 2004 (SI 1031) and all subsequent amendments thereof, and the Declaration of Helsinki (1996).

Trial Steering Committee (TSC): TSC will be convened to undertake independent review. The committee will meet at least once a year. The role of the TSC is to provide the overall supervision of the trial and to ensure that the trial is conducted to the rigorous standards set out in the Department of Health's Research Governance Framework for Health and Social Care and Guidelines for Good Clinical Practice. TSC members consist of individuals who are independent of the investigators, their employing organisations, and sponsors. The TSC will monitor trial progress and conduct and advise on scientific credibility. The TSC will consider and act, as appropriate, upon the recommendations of the Data Monitoring Committee (DMC).

Data Monitoring Committee (DMC): The DMC will assess at annual intervals at least, the progress of the clinical trial, the safety data, and the critical efficacy endpoints, and will recommend to the sponsor whether to continue, modify, or stop the trial. If the study is prematurely discontinued, participants enrolled to date will be informed and no further participants will be recruited. For participants already enrolled data may still continue to be collected.

Clinical Endpoints Committee (CEC): The CEC will adjudicate important clinical events including those listed as primary outcome measures.

Trial monitoring: Monitoring of study conduct and data collected will be performed by a combination of central review and site monitoring visits to ensure the study is conducted in accordance with GCP. Study site monitoring will be undertaken by NCTU, following a risk-based assessment approved by sponsor. The main areas of focus will include consent, serious adverse events, and review of essential documents in study files.

Site monitoring will include: Original consent forms will be reviewed as part of the study file. The presence of a copy in the patient hospital notes will be confirmed for participants. Reported serious adverse events will be verified against treatment notes/medical records (source data verification). The presence of essential documents in the investigator site file and study files will be checked. Source data verification of primary endpoint data and eligibility data for participants entered in the study will be performed.

Central monitoring will include: All applications for study authorisations and submissions of progress/safety reports will be reviewed for accuracy and completeness, prior to submission. All documentation essential for study initiation will be reviewed prior to site authorisation. All monitoring findings will be reported and followed up with the appropriate persons in a timely manner. The study may be subject to inspection and audit by NUTH under their remit as sponsor, and other regulatory bodies to ensure adherence to GCP. The investigator(s) / institutions will permit trial-related monitoring, audits, REC review and regulatory inspection(s), providing direct access to source data/documents.

Serious Breaches: It is the responsibility of the Chief Investigator (CI) and Co-Investigators to ensure that the clinical trial is run in accordance with GCP and the protocol. Given this is a multicentre trial, this task is delegated to the local Principal Investigators at each site. Any actual or suspected breaches must be reported to the Research Governance Manager (RGM) and/or Quality Assurance Manager (QAM) within 24 hours of identification. The breach will also need to be reported to the REC. Deviations from the protocol and GCP occur in clinical trials and the majority of these events are technical deviations that are not serious breaches. Deviations from allocated treatment should be recorded in the eCRF. Other deviations from protocol or GCP should be recorded on a Deviation Log, in order for Corrective and Preventative Actions (CAPA) to be taken.

Discontinuation rules: The trial may be prematurely discontinued on the basis of new safety information, or for other reasons given by the Trial Steering Committee (TSC), Data Monitoring Committee (DMC) and/or Sponsor, or ethics committee concerned.

13. ETHICAL AND REGULATORY CONSIDERATIONS

13.1. Research Ethics Committee Review and Reports

The NCTU will obtain a favourable ethical opinion from an NHS Research Ethics Committee (REC) in England prior to the start of the trial. All parties will conduct the trial in accordance with this ethical opinion. The NCTU will notify the REC of all required substantial amendments to the trial and those non-substantial amendments that result in a change to trial documentation (e.g. protocol or patient information sheet). Substantial amendments that require a REC favourable opinion will not be implemented until this REC favourable opinion is obtained. The NCTU will notify the REC of any serious breaches of GCP or the protocol, urgent safety measures or USARs that occur during the trial. An annual progress report will be submitted each year to the REC by the NCTU until the end of the trial. This report will be submitted within 30 days of the anniversary date on which the original favourable ethical opinion was granted. The NCTU will notify the REC of the early termination or end of trial in accordance with the required timelines.

13.2. Public and Patient Involvement

We have included patient group involvement in SENIOR-RITA through VOICE (http://www.ncl.ac.uk/nica/voice/). VOICE is linked to our UK National Innovation Centre of Ageing, Newcastle University. The lay summary of the SENIOR-RITA protocol was presented to the VOICE Research Support Group. One member of VOICE has been invited to participate as a Trial Steering Committee lay member. Once the study is completed, the patient group will also support with the dissemination of study findings to the lay public and patients.

13.3. Regulatory Compliance

The trial will be conducted in accordance with the Health Research Authority guidance. Before any site can enrol patients into the trial, that site must have received NHS permission from the site's Research & Development department.

13.4. Protocol Compliance

All investigators and study teams must adhere to the protocol, in particular the inclusion and exclusion criteria of the study. Waivers of the eligibility criteria are not permitted. Any deviation from the protocol must be notified to the NCTU trial manager on a deviation log for assessment and onward reporting to the sponsor.

13.5. Notification of Serious Breaches to GCP and/or the Protocol

A serious breach is a breach which is likely to affect (to a significant degree) the safety or physical or mental integrity of the subjects of the trial; or the scientific value of the trial. The sponsor must be notified immediately of any incident that may be classified as a serious breach. The NCTU will notify the NHS REC within the required timelines in accordance with the NCTU SOP.

13.6. Data Protection and Patient Confidentiality

All personal data will be regarded as strictly confidential. The study will comply with the General Data Protection Regulation 2018. All study records and Investigator Site Files will be kept at site in a locked filing cabinet with restricted access.

13.7. Indemnity

The Newcastle upon Tyne Hospitals NHS Foundation Trust is Sponsor and through the Sponsor, NHS indemnity is provided in respect of potential liability and negligent harm arising from study management. Indemnity in respect of potential liability arising from negligent harm related to study design is provided by the substantive employers of protocol authors (HEIs/NHS). All study sites are NHS organisations and indemnity in respect of potential liability arising from negligent harm related to study conduct at individual sites will be provided via NHS schemes.

13.8. Amendments

It is the responsibility of the Research Sponsor to determine if an amendment is substantial or not and study procedures must not be changed without the mutual agreement of the Chief Investigator, Co-Investigators, Sponsor, Trial Management Group and Trial Steering Committee. Substantial amendments will be submitted to the REC and will not be implemented until this approval is in place. It is the responsibility of the NCTU to submit substantial amendments. Non-substantial amendments may be made at any time with a record of the amendment held in the Trial Master File. Any non-substantial amendment that requires an update to the trial documentation will be submitted to the NHS REC for acknowledgement of the revised version of the document. Substantial amendments and those minor amendments which may impact sites will be submitted to the relevant NHS R&D Departments for notification to determine if the amendment affects the NHS permission for that site. Amendment documentation will be provided to sites by the NCTU.

14. DISSEMINATION POLICY

The data will be the property of the Chief Investigator and Co-Investigators. Publication will be the responsibility of the Chief Investigator and the Trial Management Group and published under the authorship agreed with all the Co-Investigators who fulfil the ICMJE criteria for authorship. All sites, their Principal Investigators and their team members will be acknowledged as contributors in the main publication. It is planned to publish this trial in peer-reviewed journals and to present data at national and international meetings. Results of the study will also be reported to the Sponsor and Funder. Participants will be informed about their treatment and their contribution to the study at the end of the trial, including a lay summary of the results.

15. REFERENCES

1. Fox KA, Clayton TC, Damman P, Pocock SJ, de Winter RJ, Tijssen JG, et al. Long-term outcome of a routine versus selective invasive strategy in patients with non-ST-segment elevation acute coronary syndrome a meta-analysis of individual patient data. J Am Coll Cardiol. 2010;55(22):2435-45.
2. Fox KA, Poole-Wilson PA, Henderson RA, Clayton TC, Chamberlain DA, Shaw TR, et al. Interventional versus conservative treatment for patients with unstable angina or non-ST-elevation myocardial infarction: the British Heart Foundation RITA 3 randomised trial. Randomized Intervention Trial of unstable Angina. Lancet. 2002;360(9335):743-51.
3. Organisation WH. http://www.who.int/healthinfo/survey/ageingdefnolder/en/ [
4. Orimo H, Ito H, Suzuki T, Araki A, Hosoi T, Sawabe M. Reviewing the definition of “elderly”. Geriatrics & Gerontology International. 2006;6(3):149-58.
5. Townsend N, Wickramasinghe K, Bhatnagar P, Smolina K, Nichols M, Leal J, et al. Coronary heart disease statistics 2012 edition. London: British Heart Foundation; 2012.
6. Goodman SG, Huang W, Yan AT, Budaj A, Kennelly BM, Gore JM, et al. The expanded Global Registry of Acute Coronary Events: baseline characteristics, management practices, and hospital outcomes of patients with acute coronary syndromes. American heart journal. 2009;158(2):193-201 e1-5.
7. Devlin G, Gore JM, Elliott J, Wijesinghe N, Eagle KA, Avezum A, et al. Management and 6-month outcomes in elderly and very elderly patients with high-risk non-ST-elevation acute coronary syndromes: The Global Registry of Acute Coronary Events. European heart journal. 2008;29(10):1275-82.
8. Nguyen HL, Goldberg RJ, Gore JM, Fox KA, Eagle KA, Gurfinkel EP, et al. Age and sex differences, and changing trends, in the use of evidence-based therapies in acute coronary syndromes: perspectives from a multinational registry. Coronary artery disease. 2010;21(6):336-44.
9. Avezum A, Makdisse M, Spencer F, Gore JM, Fox KA, Montalescot G, et al. Impact of age on management and outcome of acute coronary syndrome: observations from the Global Registry of Acute Coronary Events (GRACE). American heart journal. 2005;149(1):67-73.
10. Alexander KP, Newby LK, Cannon CP, Armstrong PW, Gibler WB, Rich MW, et al. Acute coronary care in the elderly, part I: Non-ST-segment-elevation acute coronary syndromes: a scientific statement for healthcare professionals from the American Heart Association Council on Clinical Cardiology: in collaboration with the Society of Geriatric Cardiology. Circulation. 2007;115(19):2549-69.
11. Hordijk-Trion M, Lenzen M, Wijns W, de Jaegere P, Simoons ML, Scholte op Reimer WJ, et al. Patients enrolled in coronary intervention trials are not representative of patients in clinical practice: results from the Euro Heart Survey on Coronary Revascularization. European heart journal. 2006;27(6):671-8.
12. Granger CB, Goldberg RJ, Dabbous O, Pieper KS, Eagle KA, Cannon CP, et al. Predictors of hospital mortality in the global registry of acute coronary events. Archives of internal medicine. 2003;163(19):2345-53.
13. Bagnall AJ, Goodman SG, Fox KA, Yan RT, Gore JM, Cheema AN, et al. Influence of age on use of cardiac catheterization and associated outcomes in patients with non-ST-elevation acute coronary syndromes. The American journal of cardiology. 2009;103(11):1530-6.
14. Fox KAA, Anderson FA, Dabbous OH, Steg PG, López-Sendón J, Van de Werf F, et al. Intervention in acute coronary syndromes: do patients undergo intervention on the basis of their risk characteristics? The Global Registry of Acute Coronary Events (GRACE). Heart. 2007;93(2):177-82.
15. Fried LP, Tangen CM, Walston J, Newman AB, Hirsch C, Gottdiener J, et al. Frailty in older adults: evidence for a phenotype. J Gerontol A Biol Sci Med Sci. 2001;56(3):M146-56.

16. Rockwood K, Song X, MacKnight C, Bergman H, Hogan DB, McDowell I, et al. A global clinical measure of fitness and frailty in elderly people. CMAJ. 2005;173(5):489-95.
17. Ekerstad N, Swahn E, Janzon M, Alfredsson J, Lofmark R, Lindenberger M, et al. Frailty is independently associated with 1-year mortality for elderly patients with non-ST-segment elevation myocardial infarction. Eur J Prev Cardiol. 2014;21(10):1216-24.
18. Ekerstad N, Swahn E, Janzon M, Alfredsson J, Lofmark R, Lindenberger M, et al. Frailty Is Independently Associated With Short-Term Outcomes for Elderly Patients With Non-ST-Segment Elevation Myocardial Infarction. Circulation. 2011;124(22):2397-404.
19. Veerasamy M, Edwards R, Ford G, Kirkwood T, Newton J, Jones D, et al. Acute Coronary Syndrome among Older Patients: A Review. Cardiology in review. 2014;23(1):26-32.
20. Pfisterer M, Buser P, Osswald S, Allemann U, Amann W, Angehrn W, et al. Outcome of elderly patients with chronic symptomatic coronary artery disease with an invasive vs optimized medical treatment strategy: one-year results of the randomized TIME trial. JAMA : the journal of the American Medical Association. 2003;289(9):1117-23.
21. Bauer T, Koeth O, Junger C, Heer T, Wienbergen H, Gitt A, et al. Effect of an invasive strategy on in-hospital outcome in elderly patients with non-ST-elevation myocardial infarction. European heart journal. 2007;28(23):2873-8.
22. Savonitto S, Cavallini C, Petronio AS, Murena E, Antonicelli R, Sacco A, et al. Early aggressive versus initially conservative treatment in elderly patients with non-ST-segment elevation acute coronary syndrome: a randomized controlled trial. JACC Cardiovascular interventions. 2012;5(9):906-16.
23. Sinclair H, Kunadian V. Coronary revascularisation in older patients with non-ST elevation acute coronary syndromes. Heart. 2016.
24. Lee PY, Alexander KP, Hammill BG, Pasquali SK, Peterson ED. Representation of elderly persons and women in published randomized trials of acute coronary syndromes. JAMA : the journal of the American Medical Association. 2001;286(6):708-13.
25. Kandzari DE, Roe MT, Chen AY, Lytle BL, Pollack CV, Jr., Harrington RA, et al. Influence of clinical trial enrollment on the quality of care and outcomes for patients with non-ST-segment elevation acute coronary syndromes. American heart journal. 2005;149(3):474-81.
26. Alter DA, Manuel DG, Gunraj N, Anderson G, Naylor CD, Laupacis A. Age, risk-benefit trade-offs, and the projected effects of evidence-based therapies. The American journal of medicine. 2004;116(8):540-5.
27. Tinetti ME, Bogardus ST, Jr., Agostini JV. Potential pitfalls of disease-specific guidelines for patients with multiple conditions. The New England journal of medicine. 2004;351(27):2870-4.
28. Tegn N, Abdelnoor M, Aaberge L, Endresen K, Smith P, Aakhus S, et al. Invasive versus conservative strategy in patients aged 80 years or older with non-ST-elevation myocardial infarction or unstable angina pectoris (After Eighty study): an open-label randomised controlled trial. Lancet. 2016.
29. Seto TB, Taira DA, Berezin R, Chauhan MS, Cutlip DE, Ho KK, et al. Percutaneous coronary revascularization in elderly patients: impact on functional status and quality of life. Annals of internal medicine. 2000;132(12):955-8.
30. Chait R, Zad O, Ramineni R, Shukla A, Mitchell A. Midterm outcomes and quality of life following percutaneous coronary intervention in nonagenarians. The American journal of cardiology. 2011;107(11):1609-12.
31. Thygesen K, Alpert JS, Jaffe AS, Chaitman BR, Bax JJ, Morrow DA, et al. Fourth universal definition of myocardial infarction (2018). European heart journal. 2018;40(3):237-69.
32. Machin D, Campbell MJ, Tan SB, Tan SH. Comparing Survival Curves. Sample Size Tables for Clinical Studies: Wiley-Blackwell; 2009. p. 84-101.
33. Zaman MJ, Stirling S, Shepstone L, Ryding A, Flather M, Bachmann M, et al. The association between older age and receipt of care and outcomes in patients with acute coronary syndromes: a

cohort study of the Myocardial Ischaemia National Audit Project (MINAP). European heart journal. 2014;35(23):1551-8.
34. Barthel FM-S, Babiker A, Royston P, Parmar M K B Evaluation of sample size and power for multi-arm survival trials allowing for non-uniform accrual, non-proportional hazards, loss to follow-up and cross-over. . Statistics in Medicine. 2006;25:2521-42.
35. Barthel FM-S, P. Royston, and A. Babiker. A menu-driven facility for complex sample size calculation in randomized controlled trials with a survival or a binary outcome: Update. Stata Journal. 2005;5:123-9.
36. **Beecham J KM**. Costing psychiatric interventions. In: Thornicroft, Graham, ed. Measuring Mental Health Needs (Second Edition). Royal College of Psychiatrists, London, 2001, 200-224. Measuring Mental Health Needs **2001**;Second Edition 24.
37. Patel A RA, Moran S, et al. A comparison of two methods of collecting economic data in primary care. . Family Practice ;22:323-7. 2005(Family Practice ;22:323-7):5.
38. Caro JJ, Briggs AH, Siebert U, Kuntz KM. Modeling good research practices--overview: a report of the ISPOR-SMDM Modeling Good Research Practices Task Force--1. Value in health : the journal of the International Society for Pharmacoeconomics and Outcomes Research. 2012;15(6):796-803.
39. Hochman JS, Sleeper LA, Webb JG, Sanborn TA, White HD, Talley JD, et al. Early revascularization in acute myocardial infarction complicated by cardiogenic shock. SHOCK Investigators. Should We Emergently Revascularize Occluded Coronaries for Cardiogenic Shock. The New England journal of medicine. 1999;341(9):625-34.
40. Alexander JH, Reynolds HR, Stebbins AL, Dzavik V, Harrington RA, Van de Werf F, et al. Effect of tilarginine acetate in patients with acute myocardial infarction and cardiogenic shock: the TRIUMPH randomized controlled trial. JAMA : the journal of the American Medical Association. 2007;297(15):1657-66.
41. Sacco RL, Kasner SE, Broderick JP, Caplan LR, Connors JJ, Culebras A, et al. An updated definition of stroke for the 21st century: a statement for healthcare professionals from the American Heart Association/American Stroke Association. Stroke; a journal of cerebral circulation. 2013;44(7):2064-89.

16. APPENDICES

16.1. Definitions

Myocardial Infarction:

Myocardial infarction (MI) is defined by the fourth universal definition(31).

Universal definitions of myocardial injury and myocardial infarction

Criteria for myocardial injury

The term myocardial injury should be used when there is evidence of elevated cardiac troponin values (cTn) with at least one value above the 99th percentile upper reference limit (URL). The myocardial injury is considered acute if there is a rise and/or fall of cTn values.

Criteria for acute myocardial infarction (types 1, 2 and 3 MI)

The term acute myocardial infarction should be used when there is acute myocardial injury with clinical evidence of acute myocardial ischaemia and with detection of a rise and/or fall of cTn values with at least one value above the 99th percentile URL and at least one of the following:

- Symptoms of myocardial ischaemia;
- New ischaemic ECG changes;
- Development of pathological Q waves;
- Imaging evidence of new loss of viable myocardium or new regional wall motion abnormality in a pattern consistent with an ischaemic aetiology;
- Identification of a coronary thrombus by angiography or autopsy (not for types 2 or 3 MIs).

Post-mortem demonstration of acute athero-thrombosis in the artery supplying the infarcted myocardium meets criteria for *type 1 MI*.
Evidence of an imbalance between myocardial oxygen supply and demand unrelated to acute athero-thrombosis meets criteria for *type 2 MI*.
Cardiac death in patients with symptoms suggestive of myocardial ischaemia and presumed new ischaemic ECG changes before cTn values become available or abnormal meets criteria for *type 3 MI*.

Criteria for coronary procedure-related myocardial infarction (types 4 and 5 MI)

Percutaneous coronary intervention (PCI) related MI is termed *type 4a MI*.
Coronary artery bypass grafting (CABG) related MI is termed *type 5 MI*.
Coronary procedure-related MI ≤ 48 hours after the index procedure is arbitrarily defined by an elevation of cTn values > 5 times for *type 4a MI* and > 10 times for *type 5 MI* of the 99th percentile URL in patients with normal baseline values. Patients with elevated pre-procedural cTn values, in whom the pre-procedural cTn level are stable (≤ 20% variation) or falling, must meet the criteria for a > 5 or > 10 fold increase and manifest a change from the baseline value of > 20%. In addition with at least one of the following:

- New ischaemic ECG changes (this criterion is related to *type 4a MI* only);
- Development of new pathological Q waves;
- Imaging evidence of loss of viable myocardium that is presumed to be new and in a pattern consistent with an ischaemic aetiology;
- Angiographic findings consistent with a procedural flow-limiting complication such as coronary dissection, occlusion of a major epicardial artery or graft, side-branch occlusion-thrombus, disruption of collateral flow or distal embolization.

Isolated development of new pathological Q waves meets the *type 4a MI* or *type 5 MI* criteria with either revascularization procedure if cTn values are elevated and rising but less than the pre-specified thresholds for PCI and CABG.
Other types of 4 MI include *type 4b MI* stent thrombosis and *type 4c MI* restenosis that both meet *type 1 MI* criteria.
Post-mortem demonstration of a procedure-related thrombus meets the *type 4a MI* criteria or *type 4b MI* criteria if associated with a stent.

Criteria for prior or silent/unrecognized myocardial infarction

Any one of the following criteria meets the diagnosis for prior or silent/unrecognized MI:

- Abnormal Q waves with or without symptoms in the absence of non-ischaemic causes.
- Imaging evidence of loss of viable myocardium in a pattern consistent with ischaemic aetiology.
- Patho-anatomical findings of a prior MI.

Cardiogenic shock: Cardiogenic shock describes the physiological state in which reduced cardiac output (CO) and resultant tissue hypoxia occur in the presence of adequate intravascular volume. Haemodynamically, this is defined as systolic blood pressure below 90 mmHg sustained for at least 30 minutes in the absence of hypovolaemia, with a cardiac index below 1.8 Litres/minute/metre^2 without support or 2.0-2.2 Litres/minute/metre^2 with support, in the presence of a raised pulmonary capillary wedge pressure (>15 mmHg).(39, 40)

Stroke

Stroke is defined according to the American Heart Association/American Stroke Association updated definition of stroke for the 21st century.(41)

Definition of ischaemic stroke: An episode of neurological dysfunction caused by focal cerebral, spinal, or retinal infarction.

Definition of intracerebral hemorrhage: A focal collection of blood within the brain parenchyma or ventricular system that is not caused by trauma.

16.2. Amendment History

Amendment Number	Protocol version no.	Date issued	Author(s) of changes	Details of changes made
	3.0			Personnel changes
				Reduction in sample size from 2300 to 1668
				Increase in study duration due to increased recruitment period
				Removal of 'recurrent hospitalisation for MI' from secondary objectives
				Revised power calculation
				Clarification that routinely collected data includes medical records, GP records and ONS/HES/PHS data
				Clarification of primary outcome definition so analysis includes all events reported over the study period
				Change in primary outcome MI definition to using the fourth universal definition
				(section 3) Clarification that follow-up duration depends on when participant recruited
				Updated trial flow diagram
				Clarification of what data is collected from screened patients who are eligible but do not participate and subsequent CAG/PBPP applications to obtain this data
				Reduction in number of copies of consent form retained at site

				Clarification of procedure for transmission of patient documents to NCTU.
				Change in procedure for sending angio images to NCTU.
				Removal of 30-day and 3-month follow-up visits
				Removal of need to collect patient contact details for NCTU to conduct 30-day and 3-month follow ups.
				(section 6.6SoE) Increase in duration of window allowed for conducting 6-month and annual follow-up visits
				Clarification of withdrawal process and options
SA04	2.0	11/09/2017	Jaki Begum	Page 5-10: Key trial contact page updated
SA04	2.0	11/09/2017	Jaki Begum	Page 22, 2.2: TIA added to secondary objectives
SA04	2.0	11/09/2017	Jaki Begum	Page 24, 3. Study flow diagram updated. Removal of MoCA as stratification factor
SA04	2.0	11/09/2017	Jaki Begum	Page 27, 6.2: Consent text updated. If participant loses capacity, proxy to be asked to complete proxy EQ-5D-5L questionnaire
SA04	2.0	11/09/2017	Jaki Begum	Page 28; 6.3: Randomisation, MoCA removed as stratification factor.
SA04	2.0	11/09/2017	Jaki Begum	Page 29: Duration of QOL follow up updated
SA04	2.0	11/09/2017	Jaki Begum	Page 28-30, 6.4: Data Collection, Medical events/outcome listed
SA04	2.0	11/09/2017	Jaki Begum	Page 31-32, 6.6 Schedule of Events updated
SA04	2.0	11/09/2017	Jaki Begum	Page 33, Withdrawal criteria, wording updated
SA04	2.0	11/09/2017	Jaki Begum	Page 33, clarification if research team becomes aware of loss of capacity.

SA04	2.0	11/09/2017	Jaki Begum	Page 33, Last Patient Last Visit (LPLV) updated to 5 years
SA04	2.0	11/09/2017	Jaki Begum	Page 36, 8.1, Safety reporting definition table partially removed
SA04	2.0	11/09/2017	Jaki Begum	Page 37-41, 8.4 SAE reporting updated
SA04	2.0	11/09/2017	Jaki Begum	Page 42 Safety Reporting Diagram, flowchart updated
SA04	2.0	11/09/2017	Jaki Begum	Page 45, Subgroup analysis updated
Minor Amendment 2 (MA02)	1.2	30/09/2016	Jaki Begum	Deletion of the word 'Scotland' from text from section 13.1: *The NCTU will obtain a favourable ethical opinion from an NHS Research Ethics Committee (REC) in England and* ***Scotland*** *prior to the start of the trial"*
Minor Amendment 1 (MA01)	1.1	22/09/2016	Jaki Begum	Study not approaching participants lacking capacity in Scotland, protocol updated and to be reviewed by North Tyneside REC 2.

16.3. Fried Frailty Index

Criterion	Frailty Status
**Shrinking**	**Frailty cut point:**<br>**Baseline:** Self-reported unintentional weight loss ≥10lb in previous year<br>**Follow-up:** Unintentional weight loss ≥5% of previous year's body weight<br>*OR*<br>BMI <18.5kg/m^2
**Physical endurance/energy**	*Geriatric Depression Scale:*<br>1. *Do you feel full of energy?*<br>2. *During the last 4 weeks how often you rested in bed during day?*<br><br>Response options: Every day, every week, once, not at all.<br><br>**Frailty cut point:**<br>No to 1 and every day or every week to 2.
**Low physical activity**	*Frequency of mildly energetic, moderately energetic and very energetic physical activity.*<br><br>Response options: ≥3 times per week, 1-2 times per week, 1-3 times per month, hardly ever/never<br><br>**Frailty cut point:**<br>Hardly ever/never for very energetic physical activity AND for moderately energetic physical activity.
**Weakness**	Hand grip strength in Kg: GRIP-D handheld dynamometer, dominant hand, average of 3 measures.<br><br>**Frailty cut point:**<br>**Grip strength:** lowest 20% (by gender, body mass index)<br>*Men*<br>BMI ≤24 ≤29<br>BMI 24.1–26 ≤30<br>BMI 26.1–28 ≤30<br>BMI >28 ≤32<br>*Women*<br>BMI ≤23 ≤17<br>BMI 23.1–26 ≤17.3<br>BMI 26.1–29 ≤18<br>BMI >29 ≤21
**Slow walking speed**	Walking time in seconds (usual pace) over 15 feet<br><br>**Frailty cut point:**<br>Slowest 20%, stratified by gender and median standing height.<br>*Men*<br>Height ≤173 cm ≥7 seconds<br>Height >173 cm ≥6 seconds<br>*Women*<br>Height ≤159 cm ≥7 seconds<br>Height >159 cm ≥6 seconds<br><br>*OR*<br>Time to complete "timed up and go test" (TUG)<br><br>**Frailty cut point:**<br>TUG time ≥19 seconds

Frail: ≥3 criteria present; **Intermediate or Pre-Frail:**1 or 2 criteria present; **Robust:** 0 criteria present

Adapted from Fried et al, Cardiovascular Health Study Collaborative Research G. Frailty in older adults: Evidence for a phenotype. The Journals of Gerontology. Series A, Biological sciences and medical sciences. 2001;56:M146-156.

16.4. Rockwood Frailty Index

1	Very fit – robust, active, energetic, well-motivated and fit; these people commonly exercise regularly and are in the most fit group for their age
2	Well – without active disease, but less fit than people in category 1.
3	Well, with treated co-morbid disease – disease symptoms are well controlled compared with those in category 4
4	Apparently vulnerable – although not frankly dependent, these people commonly complain of being "slowed up" or have disease symptoms.
5	Mildly frail – with limited dependence on others for instrumental activities of daily living
6	Moderately frail – help is needed with both instrumental and non-instrumental activities of daily living
7	Severely frail – completely dependent on others for the activities of daily living, or terminally ill.

Adapted from Rockwood et al, A global clinical measure of fitness and frailty in elderly people. Canadian Medical Association Journal 2005; 173:489-495

16.5. Bleeding Academic Research Consortium definition for bleeding

Type 0	No bleeding
Type 1	Bleeding that is not actionable and does not cause the patient to seek unscheduled performance of studies, hospitalization, or treatment by a healthcare professional. May include episodes leading to self-discontinuation of medical therapy by the patient without consulting a healthcare professional.
Type 2	Any overt, actionable sign of haemorrhage (e.g. more bleeding than would be expected for a clinical circumstance, including bleeding found by imaging alone) that does not fit the criteria for Type 3, 4 or 5 but does meet at least one of the following criteria: (1) requiring non-surgical, medical intervention by a healthcare professional, (2) leading to hospitalization or increased level of care, or (3) prompting evaluation.
Type 3a	Overt bleeding plus haemoglobin drop of 3 to <5g/dl* (provided haemoglobin drop is due to bleed)<br>Any transfusion with overt bleeding
Type 3b	Overt bleeding plus haemoglobin drop ≥5g/dl* (provided haemoglobin drop is due to bleed)<br>Cardiac tamponade<br>Bleeding requiring surgical intervention for control (excluding dental/ nasal/ skin/ haemorrhoid)<br>Bleeding requiring intravenous vasoactive agents
Type 3c	Intracranial haemorrhage (does not include micro-bleeds or haemorrhagic transformation, does include intraspinal)<br>Subcategories confirmed by autopsy or imaging or lumbar puncture<br>Intraocular bleed compromising vision
Type 4:	CABG-related bleeding<br>Perioperative intracranial bleeding within 48 hours<br>Reoperation following closure of sternotomy for the purpose of controlling bleeding<br>Transfusion of ≥5 units of whole blood or packed red blood cells within a 48-hour period†<br>Chest tube output ≥ 2 litres within a 24-hour period<br>If a CABG-related bleed is not adjudicated as at least a Type 3 severity event, it will be classified as 'not a bleeding event'.
Type 5a	Probable fatal bleeding; no autopsy or imaging confirmation, but clinically suspicious
Type 5b	Definite fatal bleeding; overt bleeding or autopsy or imaging confirmation

**Corrected for transfusion (1 U packed red blood cells or 1 U whole blood1 g/dL haemoglobin).*
†Cell saver products are not counted.

Adapted from Mehran et al, Standardized bleeding definitions for cardiovascular clinical trials: A consensus report from the bleeding academic research consortium. Circulation. 2011; 123:2736-274

16.6. EQ-5D-5L

A sample English (UK) version of the EQ-5D-5L, to be completed by trial participants is available to view at the link below:

http://www.euroqol.org/fileadmin/user_upload/Documenten/PDF/Products/Sample_UK__English__EQ-5D-5L_Paper_Self_complete_v1.0__ID_24700_.pdf

In addition to the participant completed version a proxy version of the EQ-5D-5L will be completed for all participants at all time points. Please note that an online sample for this version of the EQ-5D-5L is not available, but more information can be found at the link below:

http://www.euroqol.org/eq-5d-products/eq-5d-5l/proxy-paper.html

We anticipate that a proportion of participants will not be able to complete the paper version. For these participants the telephone version will be completed: Please note that an online sample for this version of the EQ-5D-5: is not available, but more information can be found at the link below

http://www.euroqol.org/eq-5d-products/eq-5d-5l/telephone.html

16.7. Further discussion on sample size and power

The revised sample size of 1668 patients is based on a 24-month recruitment extension with a recruitment rate of 32 patients/month and that the primary analysis will take place once all patients have been followed up for at least a year.

The table below gives the power (and expected number of observed events) under various recruitment and minimum follow-up scenarios.

Table: Power (and expected number of events [E]) under various recruitment and follow-up scenarios (assuming a 20% control group event rate at 1 year and HR=0.78)

	12 month recruitment extension (total recruitment period of 4 years)			24 month recruitment extension (total recruitment period of 5 years)		
**Monthly accrual for remaining trial duration (from Jan 19)**	**28p/m**	**30p/m**	**32p/m**	**28p/m**	**30p/m**	**32p/m**
**Expected sample size achieved**	**1204[a]**	**1244[b]**	**1284[c]**	**1540[d]**	**1604[e]**	**1668[f]**
Follow-up for *at least* 1 year after last patient entered	0.784 (E=491)	0.795 (E=505)	0.804 (E=516)	0.909 (E=705)	0.919 (E=732)	0.925 (E=752)
Follow-up for *at least* 2 years after last patient entered	0.87 (E=619)	0.879 (E=637)	0.887 (E=654)	0.952 (E=854)	0.959 (E=888)	0.964 (E=915)

Assumed proportion of target sample size recruited per year:

[a] (10%; 33%; 29%; 28%)

[b] (10%; 32%; 29%; 29%)

[c] (10%; 30%; 30%; 30%)

[d] (8%; 26%; 22%; 22%; 22%)

[e] (8%; 25%; 23%; 22%; 22%)

[f] (7%; 24%; 23%; 23%; 23%)

While the chosen sample size (1668) will give a power higher than that for the original sample size calculation (90%), it will offer some protection against non-proportional hazards, treatment crossover, loss to follow-up and competing risks (non-CV deaths).

The calculations outlined above assume the treatment effect (HR=0.78) persists over time. In this patient population, it may be unrealistic to expect this to be the case and if the treatment effect diminishes over time then this would potentially lead to an under-powered study. An alternative assumption which serves as a sensitivity analysis to assess the likely effect of a diminishing treatment effect is to assume that the hazard ratio of 0.78 persists for the first two years after randomisation, but that it is attenuated thereafter to a value intermediate between 0.78 and 1 (see below **figure**). For example, if the HR increased to 0.90 after 2 years the power reduces to 80% (assuming an event rate of 20% in the control group, a total sample size of 1668 patients and an analysis that takes place 1 year after the last patient is recruited and utilises all available follow-up data).

Figure: Power allowing for non-proportional hazards

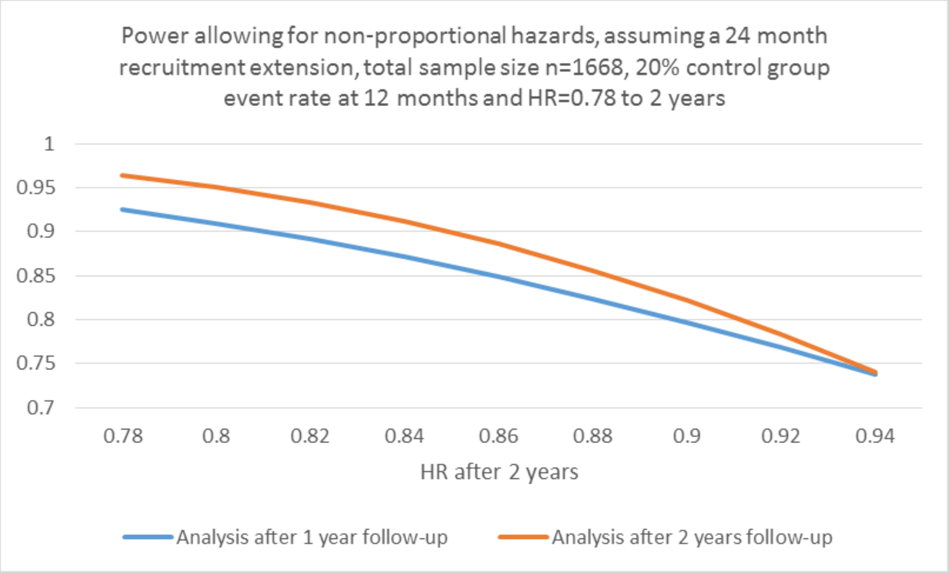

In the event that recruitment cannot be maintained at 32 patients/month alternative strategies could be employed, such as further reducing the sample size and extending the minimum follow-up to 2 years (see above **table**).

Protocol summary of changes

Amendment Number	Protocol version no.	Date issued	Author(s) of changes	Details of changes made
	3.0			Personnel changes
				Reduction in sample size from 2300 to 1668
				Increase in study duration due to increased recruitment period
				Removal of 'recurrent hospitalisation for MI' from secondary objectives
				Revised power calculation
				Clarification that routinely collected data includes medical records, GP records and ONS/HES/PHS data
				Clarification of primary outcome definition so analysis includes all events reported over the study period
				Change in primary outcome MI definition to using the fourth universal definition
				(section 3) Clarification that follow-up duration depends on when participant recruited
				Updated trial flow diagram
				Clarification of what data is collected from screened patients who are eligible but do not participate and subsequent CAG/PBPP applications to obtain this data
				Reduction in number of copies of consent form retained at site
				Clarification of procedure for transmission of patient documents to NCTU.
				Change in procedure for sending angio images to NCTU.
				Removal of 30-day and 3-month follow-up visits

				Removal of need to collect patient contact details for NCTU to conduct 30-day and 3-month follow ups.
				(section 6.6SoE) Increase in duration of window allowed for conducting 6-month and annual follow-up visits
				Clarification of withdrawal process and options
SA04	2.0	11/09/2017	Jaki Begum	Page 5-10: Key trial contact page updated
SA04	2.0	11/09/2017	Jaki Begum	Page 22, 2.2: TIA added to secondary objectives
SA04	2.0	11/09/2017	Jaki Begum	Page 24, 3. Study flow diagram updated. Removal of MoCA as stratification factor
SA04	2.0	11/09/2017	Jaki Begum	Page 27, 6.2: Consent text updated. If participant loses capacity, proxy to be asked to complete proxy EQ-5D-5L questionnaire
SA04	2.0	11/09/2017	Jaki Begum	Page 28; 6.3: Randomisation, MoCA removed as stratification factor.
SA04	2.0	11/09/2017	Jaki Begum	Page 29: Duration of QOL follow up updated
SA04	2.0	11/09/2017	Jaki Begum	Page 28-30, 6.4: Data Collection, Medical events/outcome listed
SA04	2.0	11/09/2017	Jaki Begum	Page 31-32, 6.6 Schedule of Events updated
SA04	2.0	11/09/2017	Jaki Begum	Page 33, Withdrawal criteria, wording updated
SA04	2.0	11/09/2017	Jaki Begum	Page 33, clarification if research team becomes aware of loss of capacity.
SA04	2.0	11/09/2017	Jaki Begum	Page 33, Last Patient Last Visit (LPLV) updated to 5 years
SA04	2.0	11/09/2017	Jaki Begum	Page 36, 8.1, Safety reporting definition table partially removed
SA04	2.0	11/09/2017	Jaki Begum	Page 37-41, 8.4 SAE reporting updated
SA04	2.0	11/09/2017	Jaki Begum	Page 42 Safety Reporting Diagram, flowchart updated
SA04	2.0	11/09/2017	Jaki Begum	Page 45, Subgroup analysis updated

Minor Amendment 2 (MA02)	1.2	30/09/2016	Jaki Begum	Deletion of the word 'Scotland' from text from section 13.1: *The NCTU will obtain a favourable ethical opinion from an NHS Research Ethics Committee (REC) in England and* ***Scotland*** *prior to the start of the trial"*
Minor Amendment 1 (MA01)	1.1	22/09/2016	Jaki Begum	Study not approaching participants lacking capacity in Scotland, protocol updated and to be reviewed by North Tyneside REC 2.

Biostatistics Research Group,
Population Health Sciences Institute,
Newcastle University

The British Heart Foundation older patients with non-ST segment elevation myocardial infarction randomised interventional treatment trial

Statistical Analysis Plan
SAP Version number: 1.0
SAP Date: 14/04/2021

This statistical analysis plan is based on protocol version 3.0 [02/07/2020]

ISRCTN Number: 11343602
REC Reference: 16/NE/0238

Sponsor: Newcastle upon Tyne Hospitals NHS Foundations Trust
Sponsor protocol number: 7910

Funder: British Heart Foundation
Funder reference number: CS/15/7/31679

Prepared by:

Name	Helen Mossop	Role	Trial Statistician
Signature		Date	14/04/2021

Authorised by:

Name	Prof Dawn Teare	Role	Senior Statistician
Signature		Date	14/04/2021
Name	Dr Vijay Kunadian	Role	Chief Investigator
Signature		Date	10/5/2021

This current version of the SAP and all preceding versions will be stored in the Statistical Section of the Trial Master File held by the Biostatistics Research Group.

This statistical analysis plan (SAP) provides a framework and guidelines for the statistical analysis and reporting of the BHF SENIOR-RITA trial. It has regard for published guidelines on the content of statistical analysis plans in clinical trials.[1]

This SAP applies to a clean and validated dataset. Detailed information on data collection tools, data validation, consistency and accuracy checks and data storage and archiving can be found in the current version of the Data Management Plan and Data Validation Plan.

Any deviation from the methods outlined in this SAP will be documented in the statistical end of trial report. Example Tables, Figures and Listings are for illustrative purposes only and are subject to change.

This SAP, along with all other documents relating to the analysis of this trial, will be stored in the 'Statistical Section' of the Trial Master File (TMF) held and maintained by the Biostatistics Research Group. The final signed SAP will also be stored in the main TMF.

Document history

Version	Date	Major changes made	Justification for change
0.1	20/05/2017	First draft	NA
0.2	15/06/2017	Safety reporting procedures clarified	Discussed and agreed at TMG meeting on 24/05/17. Protocol amendment to be made to clarify SAE reporting procedures.
0.3	03/12/2018	General formatting updates and changes to standard text in line with SAP template [dated 24/01/18]	To incorporate changes in made to the current SAP template
		Updates to stratification factors used for randomisation	In line with protocol amendment (v2.0)
		Analysis methods for safety data updated in line with protocol amendment to version 2.0	Due to protocol amendment (v 2.0) which clarified safety reporting requirements.
		Changes to Example Tables to reflect database updates and DMC suggestions	In line with database updates and DMC suggestions at 30th June 2017 meeting and 4th September 2018 meeting.
0.4	26/09/2019	Removed 'within one year' from the definition of the primary objective	In line with protocol version 3.0 [draft].
		Sample size calculation updated to incorporate events accruing over the whole follow-up period rather than just within 1 year from randomisation	For detailed justification see section 2.5.
		Addition of coronary angiography as an outcome measure and removal of recurrent hospitalisation for MI	Coronary angiography was added to the study database after the start of the trial. Recurrent hospitalisation for MI, specifically, was not collected in the study database. Recurrent MI's are collected.
		Clarified that the primary analysis method will be a stratified log-rank test and Cox regression model adjusted for baseline stratification factors	Primary analysis to account for frailty status (baseline stratification factor) as this should improve the precision of the estimated treatment effect.
		Removal of quality of life analyses assessed by EQ-5D-5L	Due to duplication with health economic analyses EQ-5D-5L data will only be analysed by the health economic team. Analysis methods are documented in the health economics analysis plan.
		Changes to study follow-up arrangements as per protocol version 3.0 [draft]	In line with protocol version 3.0 [draft].
		Addition of an exploratory analysis of the composite primary endpoint using the win-ratio method	To explore each component of the composite endpoint taking into account clinical priorities.

Version	Date	Major changes made	Justification for change
0.5	14/04/2021	Addition of statement to clarify how primary outcome events which cannot be adjudicated by the CEC will be handled in the analysis	Discussed and agreed with the TSC
		Definition of MI changed from 3rd to 4th Universal definition	In line with protocol version 3.0
1.0	14/04/2021	Version 0.5 made final version 1.0	NA

Abbreviations

BARC	Bleeding Academic Research Consortium
CABG	Coronary artery bypass surgery
CEC	Clinical Endpoints Committee
CI	Confidence interval
CV	Cardiovascular
Cx	Circumflex
DMC	Data monitoring committee
HR	Hazard Ratio
IQR	Interquartile range
LAD	Left anterior descending
MI	Myocardial infarction
MINAP	Myocardial ischaemia national audit project
NSTEMI	Non-ST elevation myocardial infarction
ONS	Office for National Statistics
PCI	Percutaneous coronary intervention
RCA	Right coronary artery
SAP	Statistical Analysis Plan
SAUC	Standardised area under the curve
SD	Standard deviation
SVG	Saphenous vein graft
TIA	Transient ischemic attack
TMF	Trial Master File
TMG	Trial Management Group
TSC	Trial Steering Committee
ULN	Upper limit of normal

CONTENTS

1. INTRODUCTION....6
1.1 Background and rational 6
1.2 Objectives 6

2. STUDY METHODS....7
2.1 Trial design 7
2.2 Study setting and patient population 7
2.3 Randomisation and blinding 8
2.4 Definition of outcome measures 9
2.5 Sample size and power 12

3. STATISTICAL CONSIDERATIONS....15
3.1 Timing of analyses 15
3.2 Interim analyses, data monitoring and stopping guidelines 15
3.3 Analysis populations 15

4. STUDY POPULATION....16
4.1 Participant flow through trial 16
4.1.1 Screening, eligibility and recruitment 18
4.2 Baseline characteristics 18
4.3 Treatment received 21
4.4 Follow-up and withdrawals 24
4.5 Protocol deviations 24

5. ANALYSIS METHODS....25
5.1 Analysis of primary outcome 25
5.2 Analysis of secondary outcomes 25
5.3 Additional and exploratory analyses 28
5.3.1 Sub-group analyses 28
5.3.2 Additional and exploratory analyses 28
5.4 Missing data 29

6. SAFETY....30
6.1 Procedure-related complications 30
6.2 Serious adverse events and reactions 31

7. STATISTICAL SOFTWARE....32

1. INTRODUCTION

1.1 Background and rational

Heart disease, in particular coronary artery disease, remains one of the leading causes of death in the UK. As our population ages, increasing numbers of older patients are presenting with coronary artery disease, including non-ST elevation myocardial infarction (NSTEMI).

Older patients admitted after an episode of NSTEMI are often frail, with up to half of older patients (≥75 years) being severely frail. These frail older patients are often denied routine invasive strategy (coronary angiography with a view to coronary revascularisation by percutaneous coronary intervention (PCI) or coronary artery bypass surgery (CABG)) due to fear of complications and causing harm. Whether these procedures are beneficial in older patients, in particular those with co-morbidities and those who are frail, is unknown.

This study aims to determine the benefit and risks of routine invasive strategy versus conservative management (treatment with medication only) in older patients presenting with NSTEMI.

1.2 Objectives

Primary

To determine the impact of a routine invasive strategy on cardiovascular death and non-fatal myocardial infarction (MI) compared with a conservative treatment strategy in older patients (≥75 years) with NSTEMI.

Secondary Objectives

To determine the impact of a routine invasive strategy compared with a conservative strategy on:

- All-cause death
- Cardiovascular and non-cardiovascular death
- Recurrent myocardial infarction
- Coronary angiography and coronary revascularisation
- Hospitalisation for heart failure
- Stroke
- Transient ischemic attack (TIA)
- Bleeding (Bleeding Academic Research Consortium (BARC) ≥2)
- Procedural and in-hospital complications
- Frailty
- Length of time spent at home
- Quality of life (outside the scope of this analysis plan*).
- Cost-effectiveness (outside the scope of this analysis plan*).

*Methods for the analysis and reporting of quality of life as assessed by EQ-5D-5L and cost-effectiveness will be documented in a Health Economics Analysis Plan.

2. STUDY METHODS

2.1 Trial design

SENIOR-RITA is a multicentre, prospective, phase III, open-label trial in older patients (aged ≥75 years) presenting with type 1 NSTEMI. The trial aims to assess the impact of a routine invasive strategy on cardiovascular death and non-fatal myocardial infarction (MI) compared with a conservative treatment strategy. All patients will be randomised (1:1) to receive invasive (coronary angiography ± coronary revascularisation plus optimal medical therapy) or conservative strategy (optimal medical therapy alone). For patients randomised to the invasive strategy, coronary angiography will be performed as per local practice with coronary revascularisation by PCI or CABG performed based on angiographic findings at the discretion of the attending cardiologist and the multidisciplinary team.

Patients will be followed-up for at least one year after the last patient has been recruited, up to a maximum of 5 years. Longer-term follow-up data may be collected up to 10 years using record linkage to electronic medical records (e.g. via ONS and HES data).

2.2 Study setting and patient population

This is a broad and inclusive trial that includes all-comer older patients (aged ≥75 years) with NSTEMI, including those with co-morbidities and/or cognitive impairment* in whom there is currently a lack of evidence relating to the management of NSTEMI. Patients are being recruited from approximately 40 NHS centres throughout the UK.

Inclusion Criteria

- Aged ≥75 years
- Type 1 NSTEMI during index hospitalisation

Exclusion Criteria

- Patients presenting with STEMI or unstable angina
- Patients with cardiogenic shock
- Patients with known life expectancy <1 year
- Patients in whom neither the patient nor the consultee are able and willing to provide written informed consent
- Previous inclusion in the BHF SENIOR-RITA trial
- Inability to undergo invasive coronary angiography, such as no vascular access site, or absolute contraindication to coronary revascularisation.

**Note that patients lacking capacity to provide informed consent will not be approached at Scottish sites.*

2.3 Randomisation and blinding

Patients are randomised to invasive or conservative management on a 1:1 basis using a variable-length block stratified method. Block lengths used are documented in the statistical section of the TMF. Randomisation is performed at site using a secure web-based system.

Up to 14th September 2018 stratification was based on three factors:

- Recruiting centre
- Frailty score: frail (Rockwood Frailty Score ≥5) and not frail (Rockwood Frailty Score <5)
- Cognitive impairment: impaired (Montreal Cognitive Assessment (MoCA) score <26) and not impaired (MoCA ≥26).

At the time data were first presented to the data monitoring committee (DMC) on 30th June 2017, a small proportion (~7%) of patients were found to have been randomised to the incorrect cognitive impairment strata. At the time of the next DMC meeting, this had increased to ~13%. On investigation, it became apparent that some sites found it logistically challenging to obtain a complete 30-item MoCA assessment from the participant prior to randomisation. So as not to delay treatment, randomisation was going ahead without a complete MoCA assessment being carried out, with cognitive impairment judged by the treating clinician for the purpose of randomisation and the MoCA assessment completed after randomisation. This was leading to a number of protocol deviations.

The decision was therefore made to remove cognitive impairment as a stratification factor. This was incorporated into protocol version 2.0 [14/12/17]. However due to a delay in updating the randomisation system, this was not implemented in practice until 14th September 2018, following the implementation of all other aspects of protocol version 2.0 on 20th February 2018. In total, 532 participants were recruited under the original allocation strategy.

The update to the randomisation system involved creating new blocks of participants, with variable block lengths as before. Incomplete blocks using the old strata were not used. The overall sample size should ensure this change has minimal or negligible effect on the overall balance between randomised groups at the end of the study.

Due to the nature of the intervention, it is not possible to blind patients or clinicians to trial treatment. The trial statistician (Helen Mossop) has been involved in preparing unblind reports to the DMC. The senior statistician responsible for approving this Statistical Analysis Plan has not reviewed any unblind data by randomised treatment group and will remain blind until the data are locked for the primary analysis.

2.4 Definition of outcome measures

Primary endpoint

The primary endpoint is time to cardiovascular (CV) death or non-fatal MI (defined by the fourth universal definition[2]). This will be measured as the time from randomisation to date of CV death or non-fatal MI (whichever comes first). Patients alive and free from recurrent MI at the time of analysis will be censored at the date they were last seen or assessed (latest date of follow-up / assessment on the Clinical Endpoint eCRF), up to a maximum of 5 years post randomisation. Patients experiencing non-CV death, without prior recurrent MI, will be censored at their date of death.

All death and recurrent MI events will be reviewed and adjudicated, in a blinded fashion, by an independent Clinical Endpoints Committee (CEC) and it will be this assessment which will be used for the main analysis. Further details of the adjudication process can be found in the Clinical Endpoints Committee Charter.

We do not anticipate there will be many deaths where the CEC cannot determine whether the participant died of a cardiovascular or non-cardiovascular cause (and will therefore be reported as undetermined). For the primary analysis these cases will be conservatively included as cardiovascular deaths, with sensitivity analyses excluding undetermined deaths.

For some non-fatal MI events, it is possible insufficient source data will be available to allow the CEC to adjudicate the event, however we do not anticipate this to affect many cases. In such cases the event will be counted as a non-fatal MI event, as per the local site assessment, and will be assumed to be Type 1. The number of MI events the CEC could not adjudicate will be reported.

Secondary endpoints

For all secondary endpoints, timing of analyses will coincide with the main analysis of the primary endpoint.

<u>All-cause death</u>

This will be measured as the time from randomisation to the date of death. Patients alive at the time of analysis will be censored at the date they were last seen or assessed.

<u>Cardiovascular death</u>

This will be measured as the time from randomisation to date of cardiovascular death. Patients alive at the time of analysis will be censored at the date they were last seen or assessed. Patients experiencing non-CV death will be censored at their date of death. Cause of death will be as adjudicated by the CEC.

<u>Non-cardiovascular death</u>

This will be measured as the time from randomisation to date of non-cardiovascular death. Patients alive at the time of analysis will be censored at the date they were last seen or assessed. Patients experiencing CV death will be censored at their date of death. Cause of death will be as adjudicated by the CEC.

<u>Recurrent MI</u>

Recurrent MIs are reported in the database by site staff and adjudicated by the CEC. A crude rate of recurrent MI will be calculated in each treatment group as the total number of recurrent MI events divided by the total patient follow-up time in each group.

Coronary angiography, coronary revascularisation, stroke, TIA

All events occurring from the time of randomisation will be obtained from medical records and reported by site staff in the study database. Crude rates will be calculated for each outcome measure as described above for recurrent MIs.

Bleeding

All bleeding events occurring from the time of randomisation will be reported if they are Type 2 or higher according to the BARC definition[3].

Time to first BARC Type 2 or higher event will be explored, as per the request of the DMC [at their meeting on 4th September 2018]. This will be measured as the time from randomisation to date of first BARC Type 2 event. Patients' event-free at the time of analysis will be censored at the date they were last seen or assessed, or their date of death.

A crude rate of recurrent bleeding events [Type 2 or higher] will be calculated as described above.

Procedural and in-hospital complications

Serious adverse events which are causally (possible, probable or definitely) related to the study angiography or PCI will be reported in patients randomised to the invasive arm undergoing their allocated coronary angiography procedure. Complications related to CABG will not be reported.

Expected complications include:

- Death
- Myocardial Infarction
- Minor (BARC<2) and major bleeding (BARC≥2)
- >25% increase in serum creatinine concentration from baseline
- Need for renal replacement therapy
- Coronary Dissection
- Aortic Dissection
- Coronary perforation
- Stroke
- TIA
- Cardiac tamponade
- Emergency repeat angiography or PCI procedure
- Procedure related pulmonary oedema
- Vascular complications needing intervention.

Frailty score

Frailty will be assessed using Fried and Rockwood scores at index hospitalisation, at 6 and 12 months after randomisation and annually up to 5 years. Scores at each time point will be calculated at site according to the developers' instructions, taking values 0-5 (Fried) and 1-7 (Rockwood).

Fried scores may be categorised as[4]

- Frail (score ≥3)
- Non-frail (score <3).

and

- Robust (score=0)

- Pre-frail (score=1, 2)
- Frail (score ≥3)

Similarly, Rockwood scores may be categorised as[5]

- Frail (Rockwood score ≥5)
- Non-frail (Rockwood score <5).

Standardised area under the curve (SAUC) scores will be calculated at a patient level from index hospitalisation to 12 months post randomisation conditional on patient survival[6,7] using both the Fried and Rockwood criteria. Analyses will also take place using available data up to 5 years.

Length of time spent at home

At index hospitalisation, 6 months and 12 months after randomisation and annually up to 5 years, participants are asked to complete a resource use questionnaire which asks the main place they have lived in the last 6 months:

- In their own home
- A relative or friend's home
- Sheltered housing
- Residential home
- Nursing home.

Length of time spent at home will be defined as the time from trial entry to first report of living in a residential or nursing home. The exact date will not be known so will be taken as the date at which the questionnaire was completed.

Hospitalisation for heart failure

All hospitalisations for heart failure occurring from the time of randomisation will be obtained from medical records and reported by site staff in the study database. A crude rate will be calculated in each treatment group as described above for recurrent MIs.

2.5 Sample size and power

Original calculation

The original recruitment target for this trial was a total of 2300 patients (1150 in each arm).

In previous studies, the all-cause death rates in frail and non-frail patients presenting with NSTEMI was approximately 30% at one year,[8] which equates to an assumed 12-month 'event-free survival' rate on the conservative therapy arm of 70% at 12-months. In our trial, the estimated reduction in event rate due to including only cardiovascular (CV) deaths was anticipated to be counter balanced by an increase in event rate due to the inclusion of non-fatal MI.

A clinically relevant effect size is assumed to be a 20% reduction in the overall event rate. To detect a clinically relevant reduction in CV death and MI from 30% to 24% at 12-months equates to a clinically relevant increase in event-free survival from 70% to 76% and a Hazard Ratio (HR) = 0.77. Standard sample size formula for a log-rank test[9] requires 620 events to be observed to detect a HR of 0.77 with 90% power and two-sided type I error of 5%. To observe 620 events with 12 month follow-up would require 620/(0.3+0.24) = 1149 patients to be recruited to each group[9]. The original recruitment target was therefore 2300 patients (1150 in each group).

Updated sample size calculation

In late 2018, an assessment of study progress was made by the trial management group. It was observed that recruitment was at a much slower rate than originally projected, at around 32 participants/month, and based on this recruitment rate the study would require an extension to recruitment of around 3.5 years in order to reach the target sample size of 2300. Furthermore, a blind review of available primary outcome data (unadjudicated) to December 2018 suggested the probability of an event in the first year (aggregated across treatment groups) was lower than originally anticipated at approximately 20%.

The original sample size calculation effectively assumes patients are only followed-up for 12 months. However, in reality, patients are followed up throughout the trial, not just to 12 months, and the analysis will make use of all data available on all patients. This allows events to accrue over a longer period, so fewer patients would need to be recruited to achieve the required number of events.

A more realistic approach to sample size estimation, as detailed in Barthel *et al*., 2006[10], allows events beyond one-year to be incorporated by assuming the event rate follows a piecewise exponential distribution; the recruitment rate across the trial can also be taken into account as this will inform the duration of follow up available for each patient at the time of the primary analysis. This approach can be implemented using the Stata function ARTSURV (version 1.1.0)[11].

Assuming an event rate of 20% in the conservative therapy arm at 12 months and maintaining the original clinically relevant effect size of a 20% reduction equates to an event rate of 16% in the invasive therapy arm and a HR of 0.78. To detect a HR of 0.78 with 90% power and 5% two-sided type I error requires around 700 events to be observed.

If the original recruitment period is extended for 24 months (i.e. a total recruitment period of 5 years) and a recruitment rate of 32 patients/month can be maintained then the study will recruit 1668 patients and have 92.5% power (5% two-sided type I error) to observe a HR of 0.78 assuming an event rate in the control group of 20% with the analysis taking place 1 year after the last patient is recruited and utilising all available follow-up data. This assumes a total of 752 events will have occurred at the time of the primary analysis. While this sample size gives a power higher than that for the original sample size calculation (90%), it will offer some protection against non-proportional hazards, treatment crossover, loss to follow-up and competing risks (non-CV deaths).

The table below gives the power (and expected number of observed events) under various recruitment and minimum follow-up scenarios.

Table: Power (and expected number of events [E]) under various recruitment and follow-up scenarios (assuming a 20% control group event rate at 1 year and HR=0.78)

	**12-month recruitment extension (total recruitment period of 4 years)**			**24-month recruitment extension (total recruitment period of 5 years)**		
**Monthly accrual for remaining trial duration (from Jan 19)**	**28p/m**	**30p/m**	**32p/m**	**28p/m**	**30p/m**	**32p/m**
**Expected sample size achieved**	**1204**[a]	**1244**[b]	**1284**[c]	**1540**[d]	**1604**[e]	**1668**[f]
Follow-up for *at least* 1 year after last patient entered	0.784 (E=491)	0.795 (E=505)	0.804 (E=516)	0.909 (E=705)	0.919 (E=732)	0.925 (E=752)
Follow-up for *at least* 2 years after last patient entered	0.87 (E=619)	0.879 (E=637)	0.887 (E=654)	0.952 (E=854)	0.959 (E=888)	0.964 (E=915)

Proportion of target sample size recruited per year:

[a] (10%; 33%; 29%; 28%)

[b] (10%; 32%; 29%; 29%)

[c] (10%; 30%; 30%; 30%)

[d] (8%; 26%; 22%; 22%; 22%)

[e] (8%; 25%; 23%; 22%; 22%)

[f] (7%; 24%; 23%; 23%; 23%)

The calculations outlined above assume the treatment effect (HR=0.78) persists over time. In this patient population, it may be unrealistic to expect this to be the case and if the treatment effect diminishes over time then this would potentially lead to an under-powered study. An alternative assumption, which serves as a sensitivity analysis to assess the likely effect of a diminishing treatment effect, is to assume that the hazard ratio of 0.78 persists for the first two years after randomisation, but that it is attenuated thereafter to a value intermediate between 0.78 and 1 (see below **figure**). For example, if the HR increased to 0.90 after 2 years, the power reduces to 80% (assuming an event rate of 20% in the control group, a total sample size of 1668 patients and an analysis that takes place 1 year after the last patient is recruited and utilises all available follow-up data).

Figure: Power allowing for non-proportional hazards

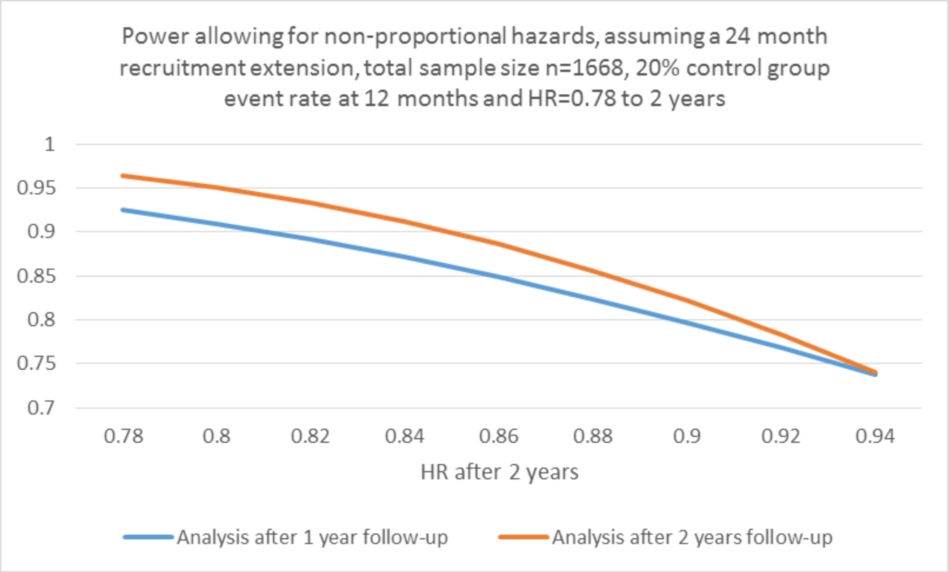

In the event that recruitment cannot be maintained at 32 patients/month, alternative strategies could be employed, such as further reducing the sample size and extending the minimum follow-up to 2 years (see above **table**).

3. STATISTICAL CONSIDERATIONS

3.1 Timing of analyses

The primary analysis is scheduled to take place once all participants have been followed-up for one year and will make use of all available follow-up data. The assumptions made in the revised sample size calculation are based on observed estimates of the event rate and recruitment rate and therefore the required number of primary endpoint events (752) should have been observed at this scheduled analysis time. The total (aggregate) number of primary endpoint events will be monitored periodically.

3.2 Interim analyses, data monitoring and stopping guidelines

The trial will be monitored by an independent data monitoring committee (DMC) that will meet at the start of the trial and at least annually thereafter, unless otherwise agreed. The DMC will review data summaries corresponding to the specific roles of the DMC charter [Version 1.0, 18/10/2016]; e.g. recruitment, data quality, adherence to protocol treatment and follow-up and descriptive summaries of safety data and main efficacy outcome measures. Underlying assumptions pertaining to the sample size calculations, including overall numbers of CV deaths and non-fatal MI, will be monitored by the DMC. The DMC will not be blind to treatment allocation.

Accumulating patient data will be reported to the DMC but (in addition to any summaries performed at the requested of the DMC) interim analyses of primary outcome data will not be undertaken until 50% of patients are recruited. While there are no formal stopping rules proposed for this trial, the DMC will make recommendations to the TSC as to whether to stop or continue recruitment based on all available evidence: when interpreting significance levels attention will be paid to Peto-Haybittle boundaries.

3.3 Analysis populations

Intention to treat (ITT): This population contains all patients randomised into the study (regardless of whether they were later found to be ineligible, a protocol violator, given the wrong treatment allocation, never treated etc.).

Safety population: This population contains patients in the intervention group undergoing their randomised coronary angiography.

Analysis of the primary endpoint and all secondary endpoints (apart from procedural and in-hospital complications) will be conducted in the ITT population. Procedural and in-hospital complications will be presented in the safety population.

There will be some participants allocated to the invasive strategy who do not undergo their allocated procedure and some participants allocated to conservative management who go on to receive coronary angiography. However, any analysis in a per-protocol population, of participants receiving their allocated treatment strategy as per-protocol, is likely to biased as treatment assignment would no longer be random and there would likely be an association between treatment switching and the risk of a clinical event. Furthermore, treatment switching reflects real-life clinical practice and a per-protocol analysis of participants receiving their allocated treatment as per-protocol would not estimate real-life clinical effectiveness. For these reasons, it is not planned to conduct any analyses in a per-protocol population of participants receiving their allocated treatment strategy.

A per-protocol analysis, excluding any participants found to be ineligible after randomisation, may be conducted as a sensitivity analysis if the number of ineligible patients is found to be excessive.

4. STUDY POPULATION

4.1 Participant flow through trial

Participant flow through the trial will be presented via a CONSORT diagram. Information will be provided on numbers and reasons (where appropriate) for: screened patients not being eligible; eligible patients not being randomised; participants found to be ineligible after randomisation; participants deviating from allocated treatment; participants not evaluable for the primary endpoints; withdrawal from follow-up; withdrawal of consent and any other major protocol deviations.

The number of ineligible patients and reasons for ineligibility will be reported.

Example Figure: CONSORT flow diagram

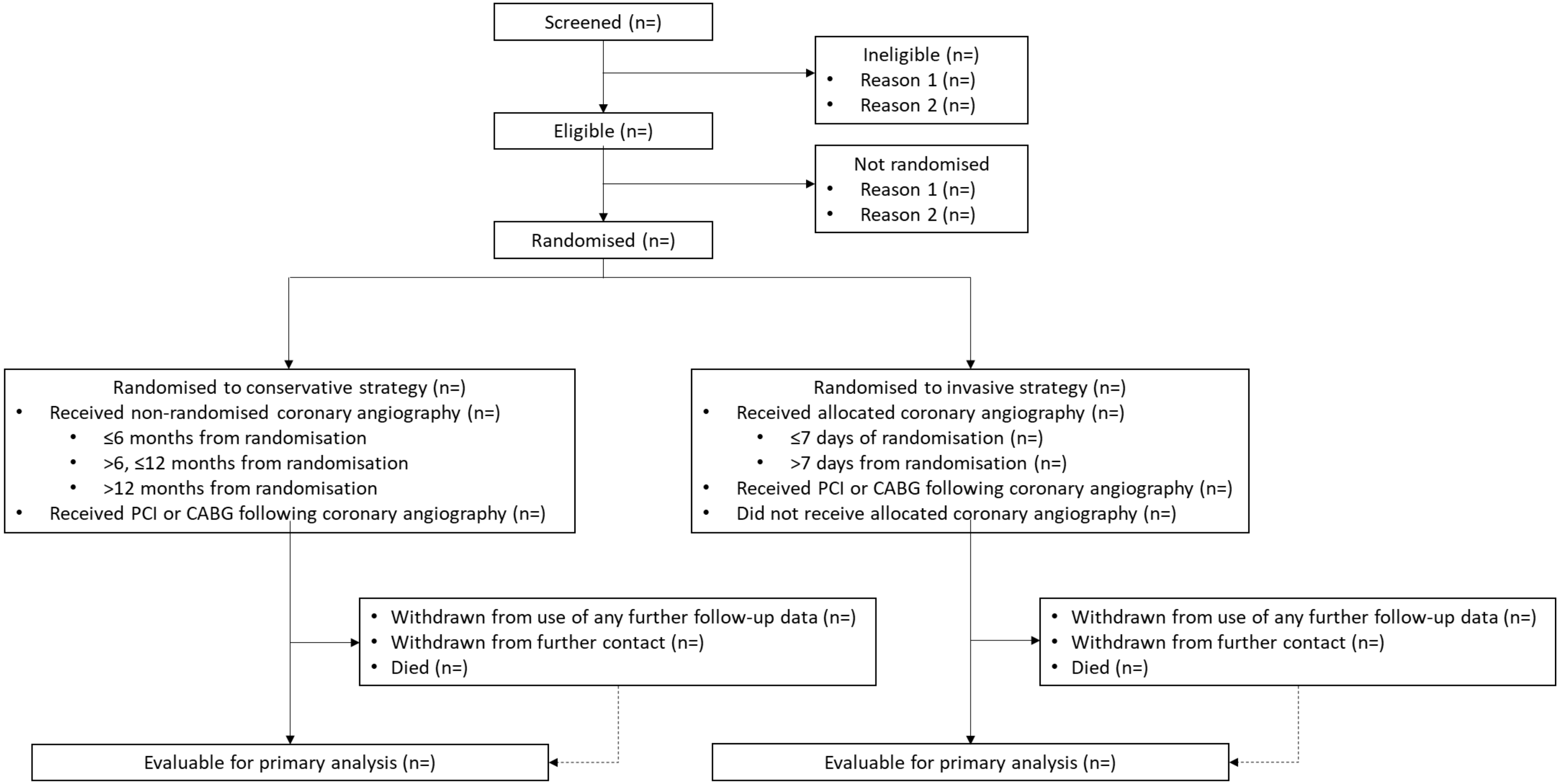

4.1.1 Screening, eligibility and recruitment

The representativeness of the study sample will be assessed using the following data:

- The number of patients identified at screening
- The number of patients excluded at screening due to ineligibility (with reasons)
- The number of eligible patients identified at screening
- The number of eligible patients not taking part in the study (with reasons)
- The treatment strategy chosen for those eligible patients not taking part in the study
- The number of eligible patients randomised into the study.

Observed and predicted cumulative recruitment will be presented graphically over time. Screening and recruitment data will be tabulated by site.

4.2 Baseline characteristics

Demographic, clinical and baseline characteristics and trial stratification factors at randomisation will be summarised by treatment groups and overall descriptively. For categorical variables, the frequency and percentage in each group will be reported and for continuous variables the mean, standard deviation (SD) and/or median, IQR and range will be reported.

Example Table: Patient demographics during index hospitalisation

Data are n; %, unless otherwise stated

	Conservative strategy (N=)	Invasive strategy (N=)	Overall (N=)
**Demographic**			
Gender<br>Male<br>Female			
Age years<br>Mean (SD)<br>Median (IQR); Range<br>≥75 to <80<br>≥80 to < 85<br>≥85 to < 90<br>≥90 to < 95<br>≥95			
**Stratification factors**			
Rockwood Frailty score<br>Frail (≥5)<br>Non Frail (<5)			
**Vital signs**			
Heart rate (bpm) Mean (SD)			
Systolic blood pressure (mmHg) Mean (SD)			
Diastolic blood pressure (mmHg) Mean (SD)			
Killip Class<br>I<br>II<br>III<br>IV			
Cardiac arrest at presentation*			

	Conservative strategy (N=)	Invasive strategy (N=)	Overall (N=)
Grace risk score			
Mean (SD)			
Median (IQR); Range			
Peak Troponin T (ng/L)‡			
Mean (SD)			
Median (IQR); Range			
Peak Troponin I (ng/L) ‡			
Mean (SD)			
Median (IQR); Range			
**Cognitive impairment, frailty, co-morbidity**			
MoCA assessment			
Median (IQR); Range			
Not impaired (≥26)			
Impaired (<26)			
Rockwood Frailty score			
Median (IQR); Range			
Frail (≥5)			
Non Frail (<5)			
Fried Frailty score			
Median (IQR); Range			
Frail (≥3)			
Pre-frail (1 or 2)			
Robust (0)			
Charlson co-morbidity index			
**ECG and Echo**			
ECG Change Present*			
ST Depression*			
T Wave Inversion*			
Transient ST Elevation*			
ST Elevation*			
Bundle Branch Block*			
Q Waves*			
Echo performed*			
LV function			
Normal			
Mild			
Moderate			
Severe			
Presence of severe aortic stenosis*			
Mitral regurgitation*			
Mild			
Moderate			
Severe			

**Number answering yes; †Exceeding 99th percentile of a normal population; ‡Will also be reported by assay type*

Example Table: History of comorbidities at baseline

Data are n; %, unless otherwise stated

	Conservative strategy (N=)	Invasive strategy (N=)	Overall (N=)
Charlson age-adjusted co-morbidity index<br>Mean (SD)<br>Median (IQR); Range			
Hypertension*			
Diabetes*			
Smoking*			
Hypercholesterolemia*			
Family History of Ischemic Heart Disease*			
Renal Disease*			
Previous Myocardial Infarction*			
Previous Angina*			
Previous PCI*			
Previous CABG*			
Peripheral Vascular Disease*			
TIA/Stroke*			
COPD*			
Malignancy*			
Congestive Heart Failure*			
Dementia*			
Liver Disease*			
Peptic Ulcer Disease*			
Bleeding History*			
Anaemia*			
Number of comorbidities<br>Mean (SD)<br>Median (IQR); Range			

**Number answering yes*

4.3 Treatment received

Patients are randomised to receive invasive coronary angiography ± coronary revascularisation plus optimal medical therapy versus optimal medical therapy alone.

Coronary angiography will be performed as per local practice. Based on angiographic findings, revascularisation by PCI or CABG will be performed at the discretion of the attending cardiologist and the multidisciplinary team. All invasive procedures will be performed according to local hospital protocols. It is recommended that patients undergo coronary angiography with a view to revascularisation (PCI/CABG) within 3-7 days after randomisation and during their index hospitalisation. Where possible, coronary revascularisation should be completed within 7 days or as soon as practically possible.

The number and proportion of patients undergoing coronary angiography and subsequent revascularisation by PCI or CABG during index hospitalisation will be reported in the intervention group. Compliance with treatment timelines will also be summarised and may also be explored by centre.

Example Table: Allocated angiography and revascularisation procedures performed in the invasive management group

Data are n; %, unless otherwise stated

	Invasive strategy (N=)
Number who received intended angiography	
Days from hospital admission	*Median (IQR); Range*
Days from randomisation	*Median (IQR); Range*
If angiography performed	
Arterial access	
Radial	
Femoral	
Brachial	
Other	
Culprit Vessel, if known	
LAD	
Cx	
RCA	
SVG	
Missing/unknown	
Imaging Used (IVUS/OCT)*	
Fractional Flow Reserve Used*	
Contrast Volume (ml)	
Mean; SD	
Median (IQR); Range	
Any revascularisation procedure from randomised angiography	
CABG	
Days from hospital admission	*Median (IQR); Range*
Days from randomisation	*Median (IQR); Range*
PCI	
Days from hospital admission	*Median (IQR); Range*
Days from randomisation	*Median (IQR); Range*
If PCI performed	
Single or multi-vessel PCI	

	Invasive strategy (N=)
Single vessel	
Multi-vessel	
Drug coated balloon used*	
Rotablation used*	
Balloon Angioplasty only	
Yes	
No	
If stent used: type	
Drug eluting stent	
Bare metal stent	
Bio-absorbable stent	
Other	
Number of stents used	
1	
2	
≥3	

**Number answering yes*

The number and proportion of patients undergoing coronary angiography and subsequent revascularisation will also be reported in both randomised treatment groups. Time to coronary angiography and time to revascularisation will be calculated and summarised using Kaplan-Meier curves. Patients not undergoing coronary angiography or revascularisation will be censored at their date of death or date last seen or assessed.

Example Table: Cardiac procedures since randomisation

Data are n; %, unless otherwise stated

	Invasive Strategy (N=)	Conservative Strategy (N=)	Overall (N=)
Number receiving coronary angiography			
Within 7 days			
Within 28 days			
Within 6 months			
Within 1 year			
Post-1 year			
Number receiving any revascularisation procedure			
Within 7 days			
Within 28 days			
Within 6 months			
Within 1 year			
Post-1 year			

Optimal medical therapy, in the absence of contraindications, will normally include aspirin 75 mg once daily; a P2Y12 receptor; statin therapy, a beta-blocker; an ACE inhibitor or ARB. Full details on optimal contemporary medical therapy is given in Section 7.2 of the protocol.

The use of optimal medical therapies will be tabulated by treatment group as reported at discharge, and 6-month and 12-month follow-up visits.

Example Table: Optimal medical therapies prescribed at discharge

Data are n; %, unless otherwise stated

	Invasive Strategy (N=)	Conservative Strategy (N=)	Overall (N=)
**Antiplatelet therapies**			
Aspirin			
P2Y12 Receptor Antagonist (Total)			
Clopidogrel			
Prasugrel			
Ticagrelor			
Antiplatelet therapy*			
None			
Single			
Dual			
**Anticoagulant* (Total)**			
Warfarin			
Rivaroxaban			
Apixaban			
Dabigatran			
Edoxaban			
Other			
**ACE inhibitor or ARB (Total)**			
Candesartan			
Lisinopril			
Perindopril			
Ramipril			
Other			
**Beta-blocker (Total)**			
Atenolol			
Bisoprolol			
Other			
**Statin (Total)**			
Atorvastatin			
Simvastatin			
Rosuvastatin			
Other			

**A cross-tabulation of antiplatelet (none, single, dual) and anticoagulant (yes, no) therapies will also be presented, as requested by the DMC*

4.4 Follow-up and withdrawals

Availability of follow-up data will be presented descriptively as frequency and percentages at each time-point and for each assessment in each randomised group.

Withdrawals from follow-up will be tabulated, noting whether the participant allowed continued use of routinely collected data or not and the time-point of withdrawal.

Example Table: Withdrawals from follow-up

Data are n; %, unless otherwise stated

	Conservative strategy (N=)	Invasive strategy (N=)	Overall (N=)
Withdrawn from follow up but allow the use of routinely collected data			
< 12 months follow up			
≥ 12 months follow up			
Withdrawn from follow up and not allowing the use of routinely collected data			
< 12 months follow up			
≥ 12 months follow up			

Duration of follow-up will be calculated using the reverse Kaplan-Meier method (the failure event will be 1 for those remaining in the study and 0 for those who have died or withdrawn from any further use of data), patients who have died will be censored at date of death and patients withdrawn from follow up and not allowing the use of routinely collected data censored at their last assessment date. Data will be presented as median (with 95% confidence interval) and interquartile range (IQR), both overall and within each randomised treatment group.

4.5 Protocol deviations

Protocol deviations will be reported overall and by randomised group. Details of protocol deviations will be reported in a line listing, sorted by type or summarised by frequency and percentage of patients reporting a particular type of deviation by randomised group if the number of that type of deviations is high and the impact low (e.g. for study visits taking place outside of allowable time-windows). Note that protocol deviations which are administrative in nature (e.g. patient identifiable data sent to an unsecure email address, incorrect versions of consent forms being signed etc.) will not be included as part of the statistical reporting.

5. ANALYSIS METHODS

5.1 Analysis of primary outcome

Event-free survival rates will be estimated using Kaplan-Meier methods and will be presented using Kaplan-Meier survival curves. Analyses will utilise all data collected, up to a maximum of 5 years post randomisation. Kaplan-Meier estimates of 6-month and 1-year event-free rates will be reported in each treatment group alongside 95% CI's.

The primary analysis will be conducted in the ITT population. The difference in survival between treatment groups will be compared using non-parametric stratified log-rank tests (stratified by Fried frailty status at randomisation*). An adjusted Cox proportional hazards regression model (adjusted by Fried frailty status at randomisation*) will be used to estimate treatment effect. A hazard ratio (HR) <1 will indicate a reduction in the event rate in the invasive treatment group and will be presented alongside a 95% CI. A further multivariable model will also be used, as a sensitivity analysis, to adjust for sex, age at trial entry, MoCA cognitive impairment status at baseline and recruiting centre (which will be added to the model as a shared frailty term).

The proportional hazards assumption of the Cox model will be investigated using Schoenfeld residuals and by testing the interaction of treatment with log(survival time), i.e. including treatment as a time dependent covariate. In the event that the statistical assumptions of the Cox model are not found to hold then the use flexible parametric survival models or restricted mean survival time will be explored.

Given that deaths from other non-CV causes would prevent the occurrence of the primary outcome events, a competing risk analysis with the competing event being non-CV death will be performed. Cumulative incidence of non-fatal MI or CV-death would be estimated using competing risks methodology and summarised by cumulative incidence curves. Estimates of 6-month and 1-year cumulative incidence would be reported in each treatment group (with 95% CI). Estimates of the treatment effect would be made using Fine and Gray's proportional regression model and presented as the sub-hazard ratio (sHR), with 95% CI. As above, if the proportional hazards assumption is not found to hold, the use of flexible parametric models would be explored.

**in the event of an error at randomisation, the correct frailty strata will be used, rather than that entered into the randomisation system.*

5.2 Analysis of secondary outcomes

<u>All-cause, CV death and non-CV death</u>

Event-free survival rates will be estimated using Kaplan-Meier methods, estimates of the treatment effect will be made using Cox regression models and tested using log-rank tests, as described above. The proportional hazards assumption will be assessed, and alternatives explored as previously described.

Competing risks analyses will be performed for the analysis of CV death, with non-CV death as the competing event, as per section 5.1.

Example Table: Primary and secondary event rates during follow-up

Endpoint		Number of events	Estimated proportion event-free by 12m	Comparison to conservative strategy (adjusted for frailty)		
		n/pts; %	%; 95% CI	HR	95% CI	p-value
**CV death or MI**	Conservative			1.00		
	Invasive					
**All cause death**	Conservative			1.00		
	Invasive					
**CV death**	Conservative			1.00		
	Invasive					
**MI**	Conservative					
	Invasive					

Example Figure (simulated data): Kaplan-Meier plot of CV death or MI

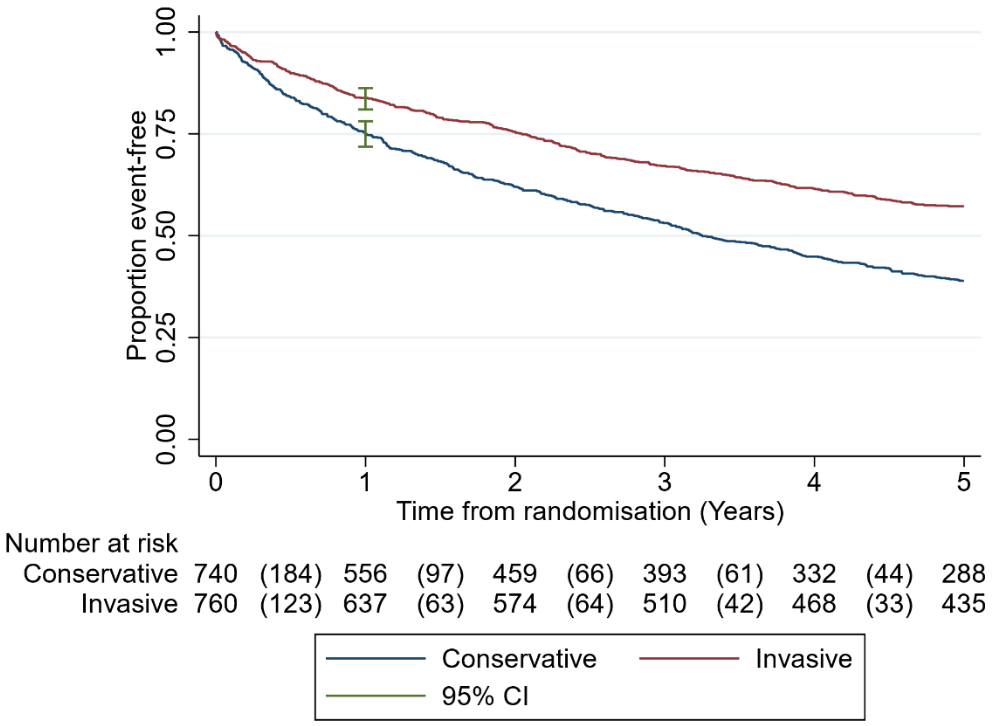

Similar Tables and Figures will be provided for all other time to event endpoints. Tables and Figures will also be provided for cumulative incidence rates and sub-hazard ratios estimated via competing risks methodology.

Recurrent MI, angiography, revascularisation, stroke, TIA, hospitalisation for heart failure

For each outcome measure, the number of participants reporting the event and the total number of events (including multiple events per participant) will be tabulated by randomised group. The crude rate (total number of events or total follow up time) will also be reported alongside 95% CI's.

Example Table: Occurrence of secondary outcome events

	Invasive Strategy (N=)	Conservative Strategy (N=)
**Total follow up**		
Years		
**Recurrent MI**		
Total [#pts (#events)]		
No. per participant		
1		
2		
≥3		
Type [#pts (#events)]		
Type 1		
Type 2		
Type 3		
Type 4-5		
Rate/100 person years (95% CI)		
**Coronary angiography**		
Total [#pts (#events)]		
No. per participant		
1		
2		
≥3		
Rate/100 person years (95% CI)		
**Revascularisation**		
Total [#pts (#events)]		
No. per participant		
1		
2		
≥3		
Rate/100 person years (95% CI)		
**Stroke**		
Total [#pts (#events)]		
No. per participant		
1		
2		
≥3		
Rate/100 person years (95% CI)		
**TIA**		
Total [#pts (#events)]		
No. per participant		
1		
2		
≥3		
Rate/100 person years (95% CI)		
**Hospitalisation for heart failure**		
Total [#pts (#events)]		
No. per participant		
1		
2		
≥3		
Rate/100 person years (95% CI)		

Bleeding

Analysis methods will follow those described above for recurrent MI, etc. Data will also be summarised descriptively by BARC type/grade. A Kaplan-Meier plot will be used to show time to first bleed (BARC ≥ 2) by randomised group. Formal statistical comparisons between treatment groups will not be made.

Frailty scores

The distribution of scores over time will be summarised descriptively for each scoring instrument (Fried and Rockwood). Change in score from baseline may also be summarised descriptively. Data will also be presented graphically.

The mean and standard deviation of the SAUC scores will be presented in each treatment group. The mean SAUC within 12 months will be compared between treatment groups using a t-test.

5.3 Additional and exploratory analyses

5.3.1 Sub-group analyses

Pre-planned sub-group analysis of primary time-to-event endpoints will be based on the following sub-groups:

- Frail versus not frail patients (Rockwood score ≥5 vs. <5).
- Cognitively impaired versus not impaired (Montreal Cognitive Assessment **(**MoCA) score <26 vs. ≥26).
- Frail and cognitively impaired patients versus non-frail, non-cognitively impaired patients
- Co-morbid (Charlson age comorbidity index >5) versus non co-morbid patients (Charlson age comorbidity index ≤5)

Hazard Ratios of the treatment effect will be calculated within identified subgroups and displayed using a Forest plot with associated 95% confidence intervals. Interactions between the levels of each sub-group and the treatment effect will evaluated with Wald tests used to assess the significance of the interaction.

5.3.2 Additional and exploratory analyses

- At a joint meeting of the DMC/TSC on 4th May 2016, prior to the trial opening to recruitment, the committee noted that rates of type 4 and 5 MI may be different early on in the trial and consideration should be given on whether this should be accounted for in the analysis as it might mask the impact of more clinically significant MIs that occur. One approach to account for this is to perform a landmark analysis of the primary outcome as per the FAME 2 trial analysis. For this study a landmark (cut-off) point of 30 days from randomisation would be used to allow time for any CABG procedures to be performed . HR's pre and post the landmark time would be presented. Alternative methods may be explored.

- Each component of the composite primary endpoint will be explored using the win-ratio method[12]. The main analysis of the primary outcome only takes the first event of the composite into account. However, this is often an event of lesser clinical importance, for example when a patient has a recurrent MI, whether they subsequently die is ignored. This method allows for clinical priorities, i.e. that CV deaths are considered more important than a recurrent MI, by first forming patients in the two treatment groups into matched pairs and then for each pair the invasive treatment is labelled a 'winner' or 'loser' depending on who had a CV death first. If that is unknown, then it will depend on who had a recurrent MI first. Otherwise they will be considered ties. The 'win-ratio' is then the total number of winners divided by the total number

of losers. Patients will be ranked and matched based on their risk profile (based on a risk score calculated from the coefficients of the adjusted Cox model for the primary outcome – excluding the treatment coefficient) and time stratified based on yearly intervals of the recruitment duration. The win-ratio will be presented with a 95% CI. This analysis will be strictly exploratory in nature.

5.4 Missing data

Levels of missing data will be summarised. Most primary and secondary outcome measures are time to event endpoints and therefore imputation of missing outcome data is not necessary.

6. SAFETY

6.1 Procedure-related complications

Procedural complications related to the study angiography or PCI will be tabulated. The number and proportion of patients experiencing a complication related to the study procedure will be reported descriptively along-side a 95% binomial confidence interval.

Example Table: Frequency of procedural related complications

	Randomised to invasive management and undergoing angiography and/or PCI (N=)	
	N (%)	95% CI
Perforation		
Coronary Dissection		
Myocardial Infarction (type 4a)		
TIA		
Stroke		
Need for renal replacement therapy		
Death		
>25% increase in serum creatinine concentration		
Aortic Dissection		
Cardiac tamponade		
Emergency repeat angiography or PCI		
Procedure related pulmonary oedema		
Vascular complications needing intervention		
Bleeding (BARC criteria)		
Type 2		
Type 3a		
Type 3b		
Type 3c		
Type 4		
Type 5a		
Type 5b		
Other complication*		
Any complication		

**Other complications will be listed*

6.2 Serious adverse events and reactions

For this study only complications meeting the following criteria will be subject to expedited reporting:

- Those which are serious and *directly related* to the randomised coronary angiography or angioplasty procedure and occur within 7 days
- Those which are serious, *unexpected* and *directly related* to the randomised coronary angiography or angioplasty procedure occurring at any time-point

Expedited safety reporting is not required for patients randomised to receive the conservative treatment strategy.

For each event undergoing expedited reporting the following will be collected, as determined by the Principal Investigator at site or the Chief Investigator if it has not been possible to obtain local medical assessment:

- Severity (mild / moderate /severe)
- Causality (possibly / probably / definitely related)
- Expectedness (expected / unexpected)

The Chief Investigator will also provide an assessment of expectedness for all events. If any doubt about causality exists, the case will be reviewed and adjudicated by the Clinical Events Committee.

Comprehensive detail of each event will be reported as a line listing.

The number of *events* and the number of *patients* reporting an event will be reported by severity.

Example Table: Line listing of all reported SAEs

ID	SAE no.	Procedure date	Onset date	Description	Severity	Causality	Outcome	Outcome date

7. STATISTICAL SOFTWARE

Data will be output directly from MACRO into a STATA format by the NCTU at time-points agreed by the TMG. Statistical analyses will be carried out by the Trial Statistician at the Biostatistics Research Group using Stata version 15 or later. All programs and output will be stored in the School Statistics folder on the IHS server.

References

1. Gamble C, Krishan A, et al. Guidelines for the Content of Statistical Analysis Plans in Clinical Trials. *JAMA*. 2017;318(23):2337-2343

2. Thygesen K, Alpert JS, et al. Fourth universal definition of myocardial infarction (2018). *European Heart Journal*. 2019;40:237-269

3. Mehran R, Rao SV, et al. Standardized bleeding definitions for cardiovascular clinical trials: A consensus report from the Bleeding Academia Research Consortium. Circulation. 2011;123:2736-2747

4. Fried LP, Tangen CM, et al. Frailty in Older Adults: Evidence for a Phenotype. *Journal of Gerontology: MEDICAL SCIENCES* 2001, Vol. 56A, No. 3, M146–M156

5. Rockwood K, Song X et al. A global clinical measure of fitness and frailty in elderly people. *CMAJ*. 2005;173(5):489-495

6. Qian W, Parmar MKB, et al. Analysis of messy longitudinal data from a randomized clinical trial. *Statist*. *Med*. 2000; 19:2657-2674

7. Billingham LJ, Abrhams KR. Simultaneous analysis of quality of life and survival data. *Statistical Methods in Medical Research*. 2002; 11: 25-48

8. Ekerstad N, Swahn E, et al. Frailty is independently associated with 1-year mortality for elderly patients with non-ST-segment elevation myocardial infarction. *Eur J Prev Cardiol*. 2014;21:1216-1224

9. Machin D, Campbell MJ et al. Comparing survival curves. *Sample size tables for clinical studies*. Wiley-Blackwell; 2009:84-101.

10. Barthel, F M-S, Babiker A, Royston P, Parmar M K B (2006) Evaluation of sample size and power for multi-arm survival trials allowing for non-uniform accrual, non-proportional hazards, loss to follow-up and cross-over. *Statistics in Medicine*, 25, 2521-2542.

11. Barthel, F M-S, Royston P, Babiker A (2005). A menu-driven facility for complex sample size calculation in randomized controlled trials with a survival or a binary outcome: Update. *Stata Journal* 5: 123–129.

12. Pocock SJ, Ariti CO et al. The win ratio: a new approach to the analysis of composite endpoints in clinical trials based on clinical priorities. *European Heart Journal.* 2012; 33: 176-182.

Biostatistics Research Group,
Population Health Sciences Institute,
Newcastle University

The British Heart Foundation older patients with non-ST segment elevation myocardial infarction randomised interventional treatment trial

Statistical Analysis Plan
SAP Version number: Version 2.0
SAP Date: 24/11/2023

This statistical analysis plan is based on protocol version 3.0 [02/07/2020]

ISRCTN Number: 11343602
REC Reference: 16/NE/0238

Sponsor: Newcastle upon Tyne Hospitals NHS Foundations Trust
Sponsor protocol number: 7910

Funder: British Heart Foundation
Funder reference number: CS/15/7/31679

Prepared by:

Name	Helen Mossop	Role	Trial Statistician
Signature		Date	24/11/2023

Authorised by:

Name	Professor Dawn Teare	Role	Senior Statistician
Signature		Date	02/12/2023
Name	Professor Vijay Kunadian	Role	Chief Investigator
Signature		Date	8/12/23

This current version of the SAP and all preceding versions will be stored in the Statistical Section of the Trial Master File held by the Biostatistics Research Group.

This statistical analysis plan (SAP) provides a framework and guidelines for the statistical analysis and reporting of the BHF SENIOR-RITA trial. It has regard for published guidelines on the content of statistical analysis plans in clinical trials.[1]

This SAP applies to a clean and validated dataset. Detailed information on data collection tools, data validation, consistency and accuracy checks, and data storage and archiving can be found in the current version of the Data Management Plan and Data Validation Plan.

Any deviation from the methods outlined in this SAP will be documented in the statistical end of trial report. Example Tables, Figures and Listings are for illustrative purposes only and are subject to change.

This SAP, along with all other documents relating to the analysis of this trial, will be stored in the 'Statistical Section' of the Trial Master File (TMF) held and maintained by the Biostatistics Research Group. The final signed SAP will also be stored in the main TMF.

Document history

Version	Date	Major changes made	Justification for change
0.1	20/05/2017	First draft	NA
0.2	15/06/2017	Safety reporting procedures clarified	Discussed and agreed at TMG meeting on 24/05/17. Protocol amendment to be made to clarify SAE reporting procedures.
0.3	03/12/2018	General formatting updates and changes to standard text in line with SAP template [dated 24/01/18]	To incorporate changes in made to the current SAP template
		Updates to stratification factors used for randomisation	In line with protocol amendment (v2.0)
		Analysis methods for safety data updated in line with protocol amendment to version 2.0	Due to protocol amendment (v 2.0) which clarified safety reporting requirements.
		Changes to Example Tables to reflect database updates and DMC suggestions	In line with database updates and DMC suggestions at 30th June 2017 meeting and 4th September 2018 meeting.
0.4	26/09/2019	Removed 'within one year' from the definition of the primary objective	In line with protocol version 3.0 [draft].
		Sample size calculation updated to incorporate events accruing over the whole follow-up period rather than just within 1 year from randomisation	For detailed justification see section 2.5.
		Addition of coronary angiography as an outcome measure and removal of recurrent hospitalisation for MI	Coronary angiography was added to the study database after the start of the trial. Recurrent hospitalisation for MI, specifically, was not collected in the study database. Recurrent MIs are collected.
		Clarified that the primary analysis method will be a stratified log-rank test and Cox regression model adjusted for baseline stratification factors	Primary analysis to account for frailty status (baseline stratification factor) as this should improve the power of the estimated treatment effect.
		Removal of quality of life analyses assessed by EQ-5D-5L	Due to duplication with health economic analyses EQ-5D-5L data will only be analysed by the health economic team. Analysis methods are documented in the health economics analysis plan.
		Changes to study follow-up arrangements as per protocol version 3.0 [draft]	In line with protocol version 3.0 [draft].
		Addition of an exploratory analysis of the composite primary endpoint using the win-ratio method	To explore each component of the composite endpoint taking into account clinical priorities.
0.5	14/04/2021	Addition of statement to clarify how primary outcome events which cannot be adjudicated by the CEC will be handled in the analysis	Discussed and agreed with the TSC
		Definition of MI changed from 3rd to 4th Universal definition	In line with protocol version 3.0
1.0	14/04/2021	Version 0.5 made final version 1.0	NA

Version	Date	Major changes made	Justification for change
2.0	24/11/2023	Amended relevant analyses to be adjusted for frailty status according to Rockwood score rather than Fried score	Fried score was stated in error. Rockwood score is used for stratification in the randomisation system and will be used in analyses.
		Added a supplementary analysis of the primary outcome which will include non-cardiovascular deaths as part of the composite outcome (i.e. non-fatal MI or death from any cause).	The TSC suggested reporting a composite all-cause mortality and MI outcome as a subsidiary primary outcome measure at their meeting on 18//10/2021. Following a review of outcome measures used in other cardiovascular trials, and giving consideration to the Estimand framework, the TMG agreed to add an analysis of all-cause mortality and MI as a supplementary analysis of the primary outcome measure (the term 'supplementary' was chosen to use the same terminology as in the Estimand framework).
		Definition of non-fatal MI added	To provide clarity as this was not previously defined
		Clarified coronary angiography and coronary revascularisation outcome measures will exclude those performed as part of the trial	Clinical interest for this outcome measure is in the need for repeat procedures.
		Added more detail on analyses to be performed if the proportional hazards assumption of the Cox regression model is not found to hold	To further pre-specify technical details of how such analyses are to be performed
		Added reporting of time to event analyses for time to first non-fatal MI	This is to be reported as it is a component of the primary outcome measure
		Analysis methods added for the Length of time spent at home outcome measure	Analysis methods added as these were previously not specified
		Removed SAUC approach for the analysis of frailty scores over time	Due to the higher than expected missing data rates due to COVID-19 the SAUC approach was no longer felt to be a robust method of analysis due to the high level of imputation which would be required.
		Additional subgroup analyses added	Additional subgroups of clinical interest pre-specified
		Sensitivity analyses for the primary outcome using multiple imputation added to explore departures from a censoring at random assumption for participants withdrawing from follow-up.	Added in response to suggestions made by the Data Monitoring Committee at their meeting on 10th May 2023
		Added a description of the estimands of interest for the primary outcome measure as an Appendix	Following release of the ICH E9 Addendum on estimands and sensitivity analyses

Abbreviations

BARC	Bleeding Academic Research Consortium
CABG	Coronary artery bypass surgery
CEC	Clinical Endpoints Committee
CI	Confidence interval
COPD	Chronic obstructive pulmonary disease
CV	Cardiovascular
Cx	Circumflex
DMC	Data monitoring committee
HR	Hazard Ratio
IQR	Interquartile range
LAD	Left anterior descending
MI	Myocardial infarction
MINAP	Myocardial ischaemia national audit project
NSTEMI	Non-ST elevation myocardial infarction
ONS	Office for National Statistics
PCI	Percutaneous coronary intervention
RCA	Right coronary artery
RMST	Restricted mean survival time
SAP	Statistical Analysis Plan
SAUC	Standardised area under the curve
SD	Standard deviation
SVG	Saphenous vein graft
TIA	Transient ischaemic attack
TMF	Trial Master File
TMG	Trial Management Group
TSC	Trial Steering Committee
ULN	Upper limit of normal

CONTENTS

1. INTRODUCTION 7
1.1 Background and rational 7
1.2 Objectives 7

2. STUDY METHODS 8
2.1 Trial design 8
2.2 Study setting and patient population 8
2.3 Randomisation and blinding 9
2.4 Definition of outcome measures 10
2.5 Sample size and power 14

3. STATISTICAL CONSIDERATIONS 17
3.1 Timing of analyses 17
3.2 Interim analyses, data monitoring and stopping guidelines 17
3.3 Analysis populations 17

4. STUDY POPULATION 18
4.1 Participant flow through trial 18
4.1.1 Screening, eligibility and recruitment 19
4.2 Baseline characteristics 19
4.3 Treatment received 23
4.4 Follow-up and withdrawals 26
4.5 Protocol deviations 26

5. ANALYSIS METHODS 27
5.1 Analysis of primary outcome 27
5.2 Analysis of secondary outcomes 28
5.3 Additional and exploratory analyses 32
5.3.1 Sub-group analyses 32
5.3.2 Additional and exploratory analyses 33
5.4 Missing data 34

6. SAFETY 35
6.1 Procedure-related complications 35
6.2 Serious adverse events and reactions 36

7. STATISTICAL SOFTWARE 37

1. INTRODUCTION

1.1 Background and rational

Heart disease, in particular coronary artery disease, remains one of the leading causes of death in the UK and worldwide. As our population ages, increasing numbers of older patients are presenting with coronary artery disease, including non-ST elevation myocardial infarction (NSTEMI).

Older patients admitted after an episode of NSTEMI are often frail, with up to a third of older patients (≥75 years) being severely frail. These frail older patients are often denied routine invasive strategy (coronary angiography with a view to coronary revascularisation by percutaneous coronary intervention (PCI) or coronary artery bypass surgery (CABG)) due to fear of complications and causing harm. Whether these procedures are beneficial in older patients, in particular those with co-morbidities and those who are frail, is unknown.

This study aims to determine the benefit and risks of routine invasive strategy (coronary angiography+/-revascularisation and optimal medical therapy) versus conservative management (optimal medical therapy only) in older patients aged 75 years or over presenting with NSTEMI.

1.2 Objectives

Primary

To determine the impact of a routine invasive strategy on cardiovascular death and non-fatal myocardial infarction (MI) compared with a conservative treatment strategy in older patients (≥75 years) presenting with NSTEMI.

Secondary Objectives

To determine the impact of a routine invasive strategy compared with a conservative strategy on:

- All-cause death
- Cardiovascular death
- Non-cardiovascular death
- Recurrent myocardial infarction
- Coronary angiography and coronary revascularisation
- Hospitalisation for heart failure
- Stroke
- Transient ischaemic attack (TIA)
- Bleeding (Bleeding Academic Research Consortium (BARC) ≥2)
- Procedural and in-hospital complications
- Frailty
- Length of time spent at home
- Quality of life (outside the scope of this analysis plan*).
- Cost-effectiveness (outside the scope of this analysis plan*).

*Methods for the analysis and reporting of quality of life as assessed by EQ-5D-5L and cost-effectiveness will be documented in a Health Economics Analysis Plan (HEAP). See the current version of the HEAP for more details.

2. STUDY METHODS

2.1 Trial design

SENIOR-RITA is a multicentre, prospective, phase III, open-label trial in older patients (aged ≥75 years) presenting with type 1 NSTEMI. The trial aims to assess the impact of a routine invasive strategy on cardiovascular death and non-fatal myocardial infarction (MI) compared with a conservative treatment strategy. All patients will be randomised (1:1) to receive invasive (coronary angiography ± coronary revascularisation plus optimal medical therapy) or conservative strategy (optimal medical therapy alone). For patients randomised to the invasive strategy, invasive coronary angiography will be performed as per local practice with coronary revascularisation by PCI or CABG performed based on angiographic findings at the discretion of the attending cardiologist and the local multidisciplinary team.

Patients will be followed-up for at least one year after the last patient has been recruited, up to a maximum of 5 years. In the future, longer-term follow-up data is planned to be collected up to 10 years using record linkage to electronic medical records (e.g. via Office for National Statistics (ONS), Hospital Episode Statistics and Scottish Health data).

2.2 Study setting and patient population

This is a broad and inclusive trial that includes all-comer older patients (aged ≥75 years) with NSTEMI, including those with co-morbidities and/or cognitive impairment* in whom there is currently a lack of evidence relating to the management of NSTEMI. Patients are being recruited from ~52 NHS centres throughout the UK.

Inclusion Criteria

- Aged ≥75 years
- Type 1 NSTEMI during index hospitalisation

Exclusion Criteria

- Patients presenting with STEMI or unstable angina
- Patients with cardiogenic shock
- Patients with known life expectancy <1 year
- Patients in whom neither the patient nor the consultee are able and willing to provide written informed consent
- Previous inclusion in the BHF SENIOR-RITA trial
- Inability to undergo invasive coronary angiography, such as no vascular access site, or absolute contraindication to coronary revascularisation.

**Note that patients lacking capacity to provide informed consent will not be approached at Scottish sites due to different mental capacity act in Scotland and due to lack of ethics approval in Scotland to approach cognitively impaired patients.*

2.3 Randomisation and blinding

Patients are randomised to invasive or conservative management on a 1:1 basis using a variable-length block stratified method. Block lengths used are documented in the statistical section of the TMF. Randomisation is performed at site using a secure web-based system.

Up to 14th September 2018 stratification was based on three factors:

- Recruiting centre
- Frailty score: frail (Rockwood Frailty Score ≥5) and not frail (Rockwood Frailty Score <5)
- Cognitive impairment: impaired (Montreal Cognitive Assessment (MoCA) score <26) and not impaired (MoCA ≥26).

At the time data were first presented to the data monitoring committee (DMC) on 30th June 2017, a small proportion (~7%) of patients were found to have been randomised to the incorrect cognitive impairment strata. At the time of the next DMC meeting, this had increased to ~13%. On investigation, it became apparent that some sites found it logistically challenging to obtain a complete 30-item MoCA assessment from the participant prior to randomisation. So as not to delay treatment, randomisation was going ahead without a complete MoCA assessment being carried out, with cognitive impairment judged by the treating clinician for the purpose of randomisation and the MoCA assessment completed after randomisation. This was leading to a number of protocol deviations.

The decision was therefore made to remove cognitive impairment as a stratification factor. This was incorporated into protocol version 2.0 [14/12/17]. However due to a delay in updating the randomisation system, this was not implemented in practice until 14th September 2018, following the implementation of all other aspects of protocol version 2.0 on 20th February 2018. In total, 532 participants were recruited under the original allocation strategy.

The update to the randomisation system involved creating new blocks of participants, with variable block lengths as before. Incomplete blocks using the old strata were not used. The overall sample size should ensure this change has minimal or negligible effect on the overall balance between randomised groups at the end of the study.

Due to the nature of the intervention, it is not possible to blind patients or clinicians to trial treatment. The trial statistician (Helen Mossop) has been involved in preparing unblind reports to the DMC. The senior statistician responsible for approving this Statistical Analysis Plan has not reviewed any unblind data by randomised treatment group and will remain blind until the data are locked for the primary analysis.

2.4 Definition of outcome measures

Primary endpoint

The primary endpoint is time to cardiovascular (CV) death or non-fatal MI (of any type, defined by the fourth universal definition[2]). This will be measured as the time from randomisation to date of CV death or non-fatal MI (whichever comes first). Patients alive and free from recurrent non-fatal MI at the time of analysis will be censored at the date they were last seen or assessed (latest date of follow-up / assessment on the Clinical Endpoint eCRF), up to a maximum of 5 years post randomisation. Patients free from recurrent non-fatal MI but experiencing non-CV death will be censored at their date of death.

All death and recurrent MI events will be reviewed and adjudicated, in a blinded fashion, by an independent Clinical Endpoints Committee (CEC) and it will be this assessment which will be used for the main analysis. Further details of the adjudication process can be found in the Clinical Endpoints Committee Charter.

Deaths will be considered cardiovascular in cause if the CEC adjudicates the cause of death as cardiovascular. In addition, while we do not anticipate there will be many deaths where the CEC cannot determine whether the participant died of a cardiovascular or non-cardiovascular cause (and will therefore be reported as undetermined), for the primary analysis these undetermined cases will be conservatively included as cardiovascular deaths. This will include cases where no source data or death certificate are available. Sensitivity analyses will be performed assuming undetermined deaths are non-cardiovascular.

For some MI events, it is possible insufficient source data will be available to allow the CEC to adjudicate the event, however we do not anticipate this to affect many cases. In such cases the event will be considered a MI, as per the local site assessment, and will be assumed to be Type 1. The number of MI events the CEC could not adjudicate will be reported. A fatal MI will be defined as cardiovascular death (as per CEC adjudication) within 30 days of MI (confirmed as per CEC adjudication, or as per local site assessment when insufficient source data is available). All other MIs will be considered non-fatal.

As a supplementary analysis we will also report a composite outcome of all-cause death and non-fatal MI. Time will be measured from randomisation to death from any cause or non-fatal MI, whichever is earliest. Patients alive and free from recurrent non-fatal MI will be censored at the date they were last seen or assessed.

Secondary endpoints

For all secondary endpoints, timing of analyses will coincide with the main analysis of the primary endpoint.

<u>All-cause death</u>

This will be measured as the time from randomisation to the date of death. Patients alive at the time of analysis will be censored at the date they were last seen or assessed.

<u>Cardiovascular death</u>

This will be measured as the time from randomisation to date of cardiovascular death. Patients alive at the time of analysis will be censored at the date they were last seen or assessed. Patients experiencing non-CV death will be censored at their date of death. Cause of death will be as adjudicated by the CEC, with undetermined deaths assumed to be cardiovascular.

<u>Non-cardiovascular death</u>

This will be measured as the time from randomisation to date of non-cardiovascular death. Patients alive at the time of analysis will be censored at the date they were last seen or assessed. Patients

experiencing CV death will be censored at their date of death. Cause of death will be as adjudicated by the CEC.

Recurrent MI

All recurrent MIs (fatal and non-fatal) are reported in the database by site staff and adjudicated by the CEC using the fourth universal definition[2].

As a component of the primary outcome, time to first non-fatal MI will be reported. Time will be measured from randomisation. Patients known to have died and not experienced a recurrent non-fatal MI will be censored at their date of death. Patients alive and free from recurrent non-fatal MI will be censored at the date they were last seen or assessed.

The total number of recurrent MIs (non-fatal, fatal, and overall), and the total number of patients affected by recurrent MIs (non-fatal, fatal, and overall), will be calculated for each randomised group. The number of occurrences and number of patients affected by recurrent MIs of each type (as per the 4th Universal Definition; Type 1 / 2 / 3 / 4a / 4b / 4c / 5) will also be calculated. The number of MIs reported per participant will also be calculated.

A crude rate of all recurrent MI events (both fatal and non-fatal, of any type) will be calculated in each treatment group as the total number of recurrent MI events divided by the total patient follow-up time in each group.

Coronary angiography and coronary revascularisation

All coronary angiography and coronary revascularisation procedures occurring from the time of randomisation will be obtained from medical records and reported by site staff in the study database. Coronary angiography and revascularisation procedures performed as part of the trial intervention will not be included in this outcome measure. This will be instead be reported as described in Section 4.3.

Time to first (non-trial) coronary angiography and time to first (non-trial) coronary revascularisation procedure will be reported. Patients not experiencing the event will be censored at their date of death or date the they were last seen or assessed.

The total number of (non-trial) coronary angiography and (non-trial) coronary revascularisation procedures, and the total number of patients affected, will be calculated for each treatment group. The number of (non-trial) coronary angiography and (non-trial) coronary revascularisation procedures reported per participant will also be calculated. Crude rates will be calculated for each outcome measure as described above for recurrent MIs.

Stroke and TIA

All occurrences of stroke and TIA occurring from the time of randomisation will be obtained from medical records and reported by site staff in the study database. For each event (stroke and TIA), the total number of events and the total number of patients affected will be calculated for each treatment group. The number of events reported per participant will also be calculated. Crude rates will be calculated for each outcome measure as described above for recurrent MIs.

Hospitalisation for heart failure

All hospitalisations for heart failure occurring from the time of randomisation will be obtained from medical records and reported by site staff in the study database. The total number of events and the total number of patients affected will be calculated for each treatment group. The number of events reported per participant will also be calculated. A crude rate will be calculated in each treatment group as described above for recurrent MIs.

Bleeding

All bleeding events occurring from the time of randomisation will be reported if they are Type 2 or higher according to the BARC definition[3].

Time to first BARC Type 2 or higher event will be explored, as per the request of the DMC [at their meeting on 4th September 2018]. This will be measured as the time from randomisation to date of first BARC Type 2 event. Patients who are event-free at the time of analysis will be censored at the date they were last seen or assessed, or their date of death.

The total number of bleeding events, and the total number of patients affected, will be calculated for each treatment group. The number of events reported per patient will also be calculated. A crude rate of bleeding events [Type 2 or higher] will be calculated as described above.

Procedural and in-hospital complications

Serious adverse events which are causally (possible, probable or definitely) related to the study angiography or PCI will be reported in patients randomised to the invasive arm undergoing their allocated coronary angiography procedure. Complications related to CABG will not be reported.

Expected procedure related complications include:

- Death
- Myocardial Infarction
- Minor (BARC<2) and major bleeding (BARC≥2)
- >25% increase in serum creatinine concentration from baseline
- Need for renal replacement therapy
- Coronary Dissection
- Aortic Dissection
- Coronary perforation
- Stroke
- TIA
- Cardiac tamponade
- Emergency repeat angiography or PCI procedure
- Procedure related pulmonary oedema
- Vascular complications needing intervention.

Frailty score

Frailty will be assessed using Fried and Rockwood scores at index hospitalisation, at 6 and 12 months after randomisation and annually up to 5 years. Scores at each time point will be calculated at site according to the developers' instructions, taking values 0-5 (Fried) and 1-7 (Rockwood).

Fried scores will be categorised as[4]

- Frail (score ≥3)
- Non-frail (score <3).

and

- Robust (score=0)
- Pre-frail (score=1, 2)
- Frail (score ≥3)

Similarly, Rockwood scores may be categorised as[5]

- Frail (Rockwood score ≥5)
- Non-frail (Rockwood score <5).

Change from baseline to each follow-up time-point will be calculated as the follow-up score minus the baseline score. Changes in status from baseline will be defined as transitions between frail and non-frail states (i.e. Stable (non-frail): non-frail to non-frail, Stable (frail): frail to frail, Worsening: non-frail to frail, Improving: frail to non-frail).

<u>Length of time spent at home</u>

At index hospitalisation, 6 months and 12 months after randomisation and annually up to 5 years, participants are asked to complete a resource use questionnaire which asks the main place they have lived in the last 6 months:

- In their own home
- A relative or friend's home
- Sheltered housing
- Residential home
- Nursing home.

Length of time spent at home will be defined as the time from trial entry to first report of living in a residential or nursing home. The exact date will not be known so will be taken as the date at which the questionnaire was completed. Patients not known to be living in a residential or nursing home will be censored using the date of the last completed resource use questionnaire.

2.5 Sample size and power

Original calculation

The original recruitment target for this trial was a total of 2300 patients (1150 in each arm).

In previous studies, the all-cause death rates in frail and non-frail patients presenting with NSTEMI was approximately 30% at one year,[8] which equates to an assumed 12-month 'event-free survival' rate on the conservative therapy arm of 70% at 12-months. In our trial, the estimated reduction in event rate due to including only cardiovascular (CV) deaths was anticipated to be counter balanced by an increase in event rate due to the inclusion of non-fatal MI.

A clinically relevant effect size is assumed to be a 20% reduction in the overall event rate. To detect a clinically relevant reduction in CV death and MI from 30% to 24% at 12-months equates to a clinically relevant increase in event-free survival from 70% to 76% and a Hazard Ratio (HR) = 0.77. Standard sample size formula for a log-rank test[9] requires 620 events to be observed to detect a HR of 0.77 with 90% power and two-sided type I error of 5%. To observe 620 events with 12 month follow-up would require 620/(0.3+0.24) = 1149 patients to be recruited to each group[9]. The original recruitment target was therefore 2300 patients (1150 in each group).

Updated sample size calculation

In late 2018, an assessment of study progress was made by the trial management group and the trial steering committee. It was accepted that recruitment was at a much slower rate than originally projected and the study would require a considerable extension to recruitment in order to reach the target sample size of 2300. Furthermore, a blind review of available primary outcome data (unadjudicated) to December 2018 suggested the probability of an event in the first year (aggregated across treatment groups) was lower than originally anticipated at approximately 20%.

The original sample size calculation effectively assumes patients are only followed-up for 12 months. However, in reality, patients are followed up throughout the trial, not just to 12 months, and the analysis will make use of all data available on all patients. This allows events to accrue over a longer period, so fewer patients would need to be recruited to achieve the required number of events.

A more realistic approach to sample size estimation, as detailed in Barthel *et al*., 2006[10], allows events beyond one-year to be incorporated by assuming the event rate follows a piecewise exponential distribution; the recruitment rate across the trial can also be taken into account as this will inform the duration of follow up available for each patient at the time of the primary analysis. This approach can be implemented using the Stata function ARTSURV (version 1.1.0)[11].

Assuming an event rate of 20% in the conservative therapy arm at 12 months and maintaining the original clinically relevant effect size of a 20% reduction equates to an event rate of 16% in the invasive therapy arm and a HR of 0.78. To detect a HR of 0.78 with 90% power and 5% two-sided type I error requires around 700 events to be observed.

If the original recruitment period is extended for 24 months (i.e. a total recruitment period of 5 years) and a recruitment rate of 32 patients/month can be maintained then the study will recruit 1668 patients and have 92.5% power (5% two-sided type I error) to observe a HR of 0.78 assuming an event rate in the control group of 20% with the analysis taking place 1 year after the last patient is recruited and utilising all available follow-up data. This assumes a total of 752 events will have occurred at the time of the primary analysis. While this sample size gives a power higher than that for the original sample size calculation (90%), it will offer some protection against non-proportional hazards, treatment crossover, loss to follow-up and competing risks (non-CV deaths).

The table below gives the power (and expected number of observed events) under various recruitment and minimum follow-up scenarios.

Table: Power (and expected number of events [E]) under various recruitment and follow-up scenarios (assuming a 20% control group event rate at 1 year and HR=0.78)

	**12-month recruitment extension (total recruitment period of 4 years)**			**24-month recruitment extension (total recruitment period of 5 years)**		
**Monthly accrual for remaining trial duration (from Jan 19)**	**28p/m**	**30p/m**	**32p/m**	**28p/m**	**30p/m**	**32p/m**
**Expected sample size achieved**	**1204[a]**	**1244[b]**	**1284[c]**	**1540[d]**	**1604[e]**	**1668[f]**
Follow-up for *at least* 1 year after last patient entered	0.784 (E=491)	0.795 (E=505)	0.804 (E=516)	0.909 (E=705)	0.919 (E=732)	0.925 (E=752)
Follow-up for *at least* 2 years after last patient entered	0.87 (E=619)	0.879 (E=637)	0.887 (E=654)	0.952 (E=854)	0.959 (E=888)	0.964 (E=915)

Proportion of target sample size recruited per year:

[a] (10%; 33%; 29%; 28%)

[b] (10%; 32%; 29%; 29%)

[c] (10%; 30%; 30%; 30%)

[d] (8%; 26%; 22%; 22%; 22%)

[e] (8%; 25%; 23%; 22%; 22%)

[f] (7%; 24%; 23%; 23%; 23%)

The calculations outlined above assume the treatment effect (HR=0.78) persists over time. In this patient population, it may be unrealistic to expect this to be the case and if the treatment effect diminishes over time then this would potentially lead to an under-powered study. An alternative assumption, which serves as a sensitivity analysis to assess the likely effect of a diminishing treatment effect, is to assume that the hazard ratio of 0.78 persists for the first two years after randomisation, but that it is attenuated thereafter to a value intermediate between 0.78 and 1 (see below **figure**). For example, if the HR increased to 0.90 after 2 years, the power reduces to 80% (assuming an event rate of 20% in the control group, a total sample size of 1668 patients and an analysis that takes place 1 year after the last patient is recruited and utilises all available follow-up data).

Figure: Power allowing for non-proportional hazards

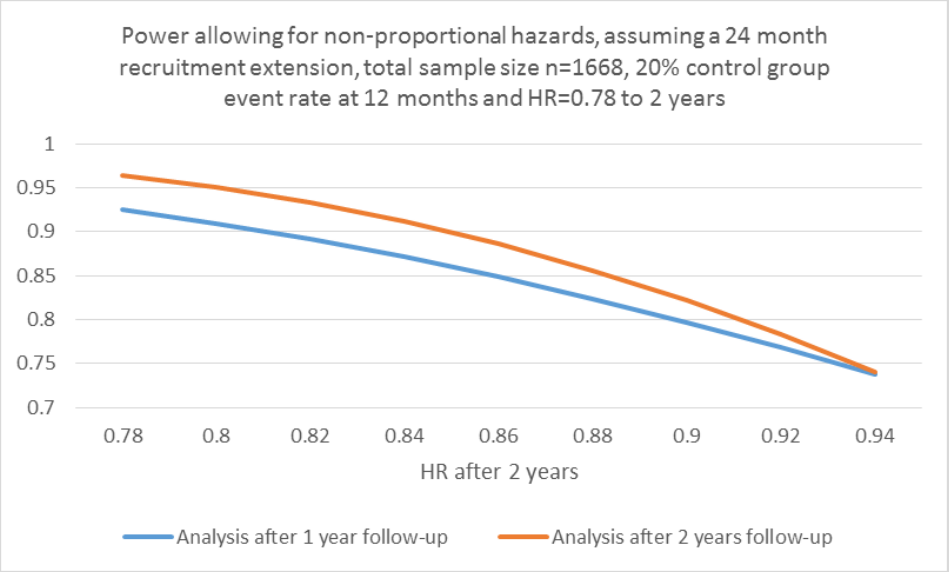

In the event that recruitment cannot be maintained at 32 patients/month, alternative strategies could be employed, such as further reducing the sample size and extending the minimum follow-up to 2 years (see above **table**).

3. STATISTICAL CONSIDERATIONS

3.1 Timing of analyses

The primary analysis is scheduled to take place once all participants have been followed-up for one year and will make use of all available follow-up data. The number of primary endpoint events, both overall and by arm, will be monitored periodically by the independent data monitoring committee (DMC) against the target number. The achieved power based on the final sample size and observed number of events will be calculated for information.

3.2 Interim analyses, data monitoring and stopping guidelines

The trial will be monitored by an independent data monitoring committee (DMC) that will meet at the start of the trial and at least annually thereafter, unless otherwise agreed. The DMC will review data summaries corresponding to the specific roles described in the DMC charter; e.g. recruitment, data quality, adherence to protocol treatment and follow-up and descriptive summaries of safety data and main efficacy outcome measures. Underlying assumptions pertaining to the sample size calculations, including overall numbers of CV deaths and non-fatal MI, will be monitored by the DMC. The DMC will not be blind to treatment allocation.

Accumulating patient data will be reported to the DMC but (in addition to any summaries performed at the requested of the DMC) interim analyses of primary outcome data will not be undertaken until 50% of patients are recruited. While there are no formal stopping rules proposed for this trial, the DMC will make recommendations to the TSC as to whether to stop or continue recruitment based on all available evidence: when interpreting significance levels attention will be paid to Peto-Haybittle boundaries.

3.3 Analysis populations

Intention to treat (ITT): This population contains all patients randomised into the study (regardless of whether they were later found to be ineligible, a protocol violator, not receiving the allocated treatment etc.).

Safety population: This population contains patients in the intervention group undergoing their randomised coronary angiography.

Analysis of the primary endpoint and all secondary endpoints (apart from procedural and in-hospital complications) will be conducted in the ITT population. Procedural and in-hospital complications will be presented in the safety population.

There will be some participants allocated to the invasive strategy who do not undergo their allocated procedure and some participants allocated to conservative management who go on to receive coronary angiography. However, any analysis in a per-protocol population, of participants receiving their allocated treatment strategy as per-protocol, is likely to biased as treatment assignment would no longer be random and there would likely be an association between treatment switching and the risk of a clinical event. Furthermore, treatment switching reflects real-life clinical practice and a per-protocol analysis of participants receiving their allocated treatment as per-protocol would not estimate real-life clinical effectiveness. For these reasons, it is not planned to conduct any analyses in a per-protocol population of participants receiving their allocated treatment strategy.

A per-protocol analysis, excluding any participants found to be ineligible after randomisation, may be conducted as a sensitivity analysis if the number of ineligible patients is found to be excessive (>2.5%).

4. STUDY POPULATION

4.1 Participant flow through trial

Participant flow through the trial will be presented via a CONSORT diagram. Information will be provided on numbers and reasons (where appropriate) for: screened patients not being eligible; eligible patients not being randomised; participants found to be ineligible after randomisation; participants deviating from allocated treatment; withdrawal from follow-up; and any other major protocol deviations.

Example Figure: CONSORT flow diagram

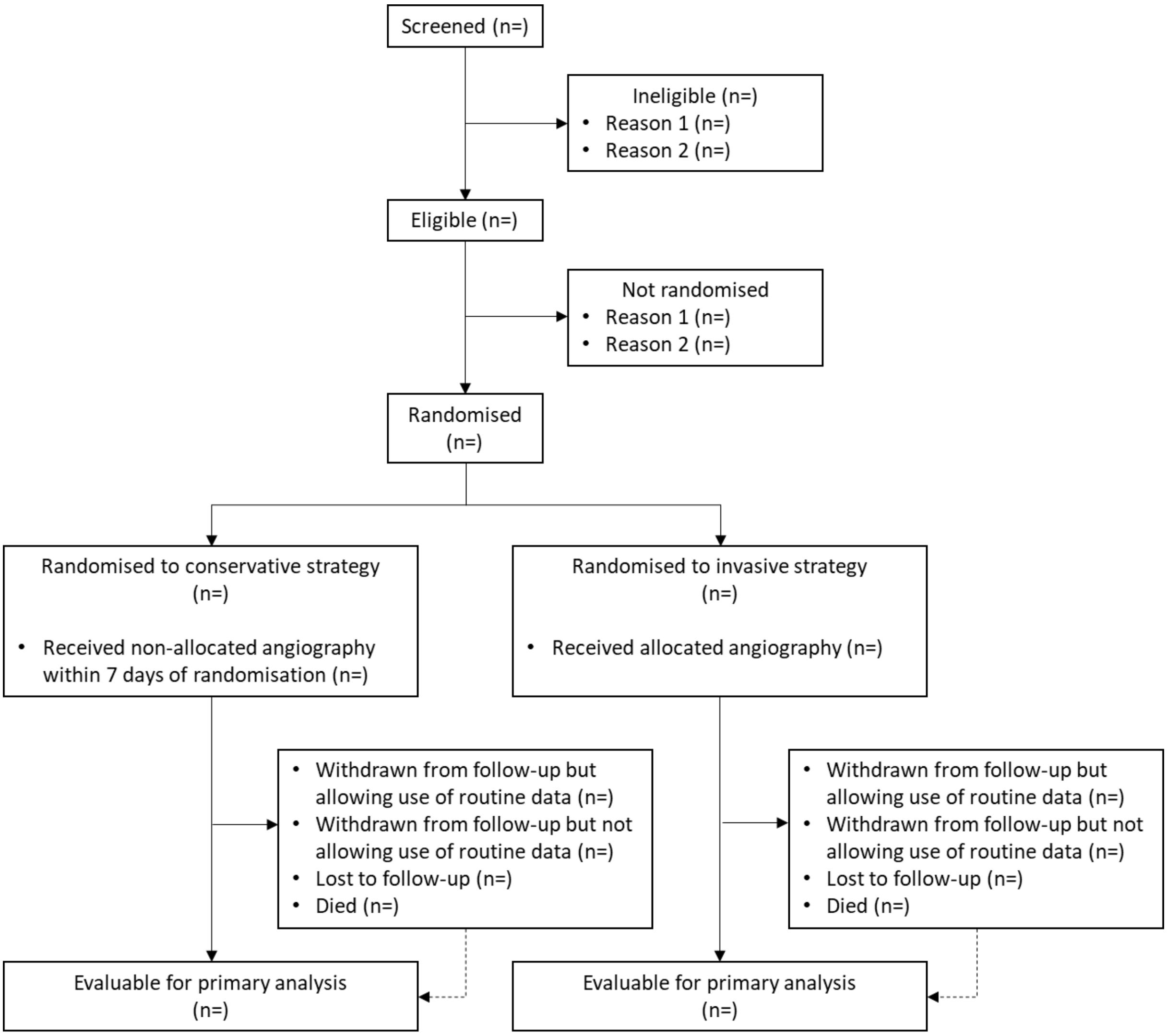

4.1.1 Screening, eligibility and recruitment

The representativeness of the study sample will be assessed using the following data:

- The number of patients identified at screening
- The number of patients excluded at screening due to ineligibility (with reasons)
- The number of eligible patients identified at screening
- The number of eligible patients not taking part in the study (with reasons)
- The treatment strategy chosen for those eligible patients not taking part in the study
- The number of eligible patients randomised into the study.

Observed and predicted cumulative recruitment will be presented graphically over time. Screening and recruitment data will also be tabulated by site.

4.2 Baseline characteristics

Demographic, clinical and baseline characteristics and trial stratification factors at randomisation will be summarised by treatment groups and overall descriptively. For categorical variables, the frequency and percentage in each group will be reported and for continuous variables the mean, standard deviation (SD) and/or median, IQR and range will be reported.

Example Table: Patient demographics during index hospitalisation

Data are n; %, unless otherwise stated

	Conservative strategy (N=)	Invasive strategy (N=)	Overall (N=)
**Demographic**			
Gender<br>Male<br>Female			
Age years<br>Mean (SD)<br>Median (IQR); Range<br>≥75 to <80<br>≥80 to < 85<br>≥85 to < 90<br>≥90 to < 95<br>≥95			
**Stratification factors**			
Rockwood Frailty score<br>Frail (≥5)<br>Non Frail (<5)			
**Vital signs**			
Heart rate (bpm) Mean (SD)			
Systolic blood pressure (mmHg) Mean (SD)			
Diastolic blood pressure (mmHg) Mean (SD)			
Killip Class<br>I<br>II<br>III<br>IV			
Cardiac arrest at presentation*			

	Conservative strategy (N=)	Invasive strategy (N=)	Overall (N=)
GRACE risk score<br>Mean (SD)<br>Median (IQR); Range			
Peak Troponin<br>Troponin T<br>Troponin I<br>*If I, high sensitivity*			
Peak Troponin T (ng/L)‡<br>Mean (SD)<br>Median (IQR); Range			
Peak Troponin I (ng/L) ‡<br>Mean (SD)<br>Median (IQR); Range			
**Cognitive impairment, frailty, co-morbidity**			
MoCA assessment<br>Median (IQR); Range<br>Not impaired (≥26)<br>Impaired (<26)			
Rockwood Frailty score<br>Median (IQR); Range			
Fried Frailty score<br>Median (IQR); Range<br>Frail (≥3)<br>Pre-frail (1 or 2)<br>Robust (0)			
Charlson age-adjusted co-morbidity index<br>Mean (SD)<br>Median (IQR); Range			
**ECG**			
ECG Change Present*<br>ST Depression*<br>T Wave Inversion*<br>Transient ST Elevation*<br>ST Elevation*<br>Bundle Branch Block*<br>Q Waves*			
Echo**cardiogram** performed*			
LV function<br>Normal<br>Mild<br>Moderate<br>Severe			
Presence of severe aortic stenosis*			
Mitral regurgitation*<br>Mild<br>Moderate<br>Severe			

**Number (%) answering yes; ‡Will also be reported by assay type*

Example Table: History of comorbidities at baseline

Data are number (%) answering yes, unless otherwise stated

	Conservative strategy (N=)	Invasive strategy (N=)	Overall (N=)
Hypertension			
Diabetes			
Smoking status<br>Current smoker<br>Ex-smoker<br>Never smoked			
Hypercholesterolaemia			
Family History of Ischaemic Heart Disease			
History of Renal Disease			
Previous Myocardial Infarction			
Previous Angina			
Previous PCI			
Previous CABG			
History of Peripheral Vascular Disease			
History of TIA/Stroke			
COPD			
Malignancy			
History of Congestive Heart Failure			
Dementia			
Liver Disease			
Peptic Ulcer Disease			
Bleeding History			
Anaemia			
Number of comorbidities<br>Mean (SD)<br>Median (IQR); Range			

Example Table: Laboratory assessments at baseline

	Conservative strategy (N=)	Invasive strategy (N=)	Overall (N=)
Haemoglobin (g/L)			
No. (%) with data			
Mean (SD)			
Median (IQR); Range			
White blood cells (10^9/L)			
No. (%) with data			
Mean (SD)			
Median (IQR); Range			
Platelets (10^9/L)			
No. (%) with data			
Mean (SD)			
Median (IQR); Range			
Urea (mmol/L)			
No. (%) with data			
Mean (SD)			
Median (IQR); Range			
Creatinine (µmol/L)			
No. (%) with data			
Mean (SD)			
Median (IQR); Range			
Glucose (mmol/L)			
No. (%) with data			
Mean (SD)			
Median (IQR); Range			
Total cholesterol (mmol/L)			
No. (%) with data			
Mean (SD)			
Median (IQR); Range			

4.3 Treatment received

4.3.1 Invasive treatment

Patients are randomised to receive invasive coronary angiography ± coronary revascularisation plus optimal medical therapy versus optimal medical therapy alone.

Coronary angiography will be performed as per local practice. Based on angiographic findings, revascularisation by PCI or CABG will be performed at the discretion of the attending cardiologist and the multidisciplinary team. All invasive procedures will be performed according to local hospital protocols. It is recommended that patients undergo coronary angiography with a view to revascularisation (PCI/CABG) within 3-7 days after randomisation and during their index hospitalisation. Where possible, coronary revascularisation should be completed within 7 days or as soon as practically possible.

The number and proportion of patients undergoing coronary angiography and subsequent revascularisation by PCI or CABG during index hospitalisation will be reported in the intervention group. Compliance with treatment timelines will also be summarised and may also be explored by centre.

Example Table: Allocated angiography and revascularisation procedures performed in the invasive management group

Data are n; %, unless otherwise stated

	Invasive strategy (N=)
Number who received intended angiography	
Reason angiography not performed<br>Clinician decided against strategy<br>Patient decided against strategy<br>Patient died prior to angiography	
Days from hospital admission	*Median (IQR); Range*
Days from randomisation	*Median (IQR); Range*
If angiography performed	
Arterial access<br>Radial<br>Femoral<br>Brachial<br>Other	
Culprit Vessel, if known<br>LAD<br>Cx<br>RCA<br>SVG<br>Missing/unknown	
Imaging Used (IVUS/OCT)*	
Fractional Flow Reserve Used*	
Contrast Volume (ml)<br>Mean; SD<br>Median (IQR); Range	
Any revascularisation procedure from randomised angiography<br>CABG<br>Days from hospital admission<br>Days from randomisation	 <br><br>*Median (IQR); Range*<br>*Median (IQR); Range*

	Invasive strategy (N=)
PCI<br>Days from hospital admission<br>Days from randomisation	 *Median (IQR); Range*<br>*Median (IQR); Range*
If PCI performed	
Single or multi-vessel PCI<br>Single vessel<br>Multi-vessel	
Drug coated balloon used*	
Rotablation used*	
Balloon Angioplasty only<br>Yes<br>No	
If stent used: type<br>Drug eluting stent<br>Bare metal stent<br>Bio-absorbable stent<br>Other	
Number of stents used<br>1<br>2<br>≥3	

**Number (%) answering yes*

The number and proportion of patients undergoing coronary angiography and subsequent revascularisation (including those performed as part of the trial intervention) within the first year of randomisation will be reported in both randomised treatment groups. Time to first coronary angiography and time to first revascularisation will be calculated and summarised using Kaplan-Meier curves. Patients not undergoing coronary angiography or revascularisation will be censored at their date of death or date last seen or assessed.

Example Table: Cardiac procedures since randomisation

Data are n; %, unless otherwise stated

	Invasive Strategy (N=)	Conservative Strategy (N=)	Overall (N=)
Number receiving coronary angiography<br>Within 7 days<br>Within 28 days<br>Within 6 months<br>Within 1 year			
Number receiving any revascularisation procedure<br>Within 7 days<br>Within 28 days<br>Within 6 months<br>Within 1 year			

4.3.2 Medical therapies

Optimal medical therapy, in the absence of contraindications, will normally include aspirin 75 mg once daily; a P2Y12 receptor antagonist; statin therapy, a beta-blocker; an ACE inhibitor or ARB. Full details on optimal contemporary medical therapy is given in Section 7.2 of the protocol.

The use of optimal medical therapies will be tabulated by treatment group as reported at discharge, and 6-month and 12-month follow-up visits.

Single antiplatelet therapy will be defined as taking either aspirin or a P2Y12 receptor antagonist. Dual antiplatelet therapy is defined as taking both aspirin and a P2Y12 receptor antagonist. Triple therapy is defined as taking aspirin, a P2Y12 receptor antagonist and an anticoagulant.

Example Table: Optimal medical therapies prescribed at discharge

Data are n (%), unless otherwise stated

	Invasive Strategy (N=)	Conservative Strategy (N=)	Overall (N=)
**Antiplatelet therapies**			
Aspirin			
P2Y12 Receptor Antagonist (Total)<br>Clopidogrel<br>Prasugrel<br>Ticagrelor			
Antiplatelet therapy*<br>None<br>Single<br>Dual			
**Anticoagulant* (Total)**<br>Warfarin<br>Rivaroxaban<br>Apixaban<br>Dabigatran<br>Edoxaban<br>Other			
**Triple therapy (Total)**			
**ACE inhibitor or ARB (Total)**<br>Candesartan<br>Lisinopril<br>Perindopril<br>Ramipril<br>Other			
**Beta-blocker (Total)**<br>Atenolol<br>Bisoprolol<br>Other			
**Statin (Total)**<br>Atorvastatin<br>Simvastatin<br>Rosuvastatin<br>Other			

**A cross-tabulation of antiplatelet (none, single, dual) and anticoagulant (yes, no) therapies will also be presented as number (%), as requested by the DMC*

4.4 Follow-up and withdrawals

Availability of follow-up data will be presented descriptively as frequency and percentages at each time-point and for each assessment in each randomised group.

Withdrawals from follow-up will be tabulated, noting whether the participant allowed continued use of routinely collected data or not and the time-point of withdrawal. Time to withdrawal will also be summarised using Kaplan-Meier curves.

Example Table: Withdrawals from follow-up

Data are n; %, unless otherwise stated

	Conservative strategy (N=)	Invasive strategy (N=)	Overall (N=)
Withdrawn from follow up but allow the use of routinely collected data			
< 12 months follow up			
≥ 12 months follow up			
Withdrawn from follow up and not allowing the use of routinely collected data			
< 12 months follow up			
≥ 12 months follow up			

Duration of follow-up will be calculated using the reverse Kaplan-Meier method (the failure event will be 1 for those remaining in the study and 0 for those who have died or withdrawn from any further use of data), patients who have died will be censored at date of death and patients withdrawn from follow up and not allowing the use of routinely collected data censored at their last assessment date. Data will be presented as median (with 95% confidence interval) and interquartile range (IQR), both overall and within each randomised treatment group.

4.5 Protocol deviations

Protocol deviations will be reported overall and by randomised group. Details of protocol deviations will be reported in a line listing, sorted by type or summarised by frequency and percentage of patients reporting a particular type of deviation by randomised group if the number of that type of deviations is high and the impact low (e.g. for study visits taking place outside of allowable time-windows). Note that protocol deviations which are administrative in nature (e.g. patient identifiable data sent to an unsecure email address, incorrect versions of consent forms being signed etc.) will not be included as part of the statistical reporting.

5. ANALYSIS METHODS

5.1 Analysis of primary outcome

5.1.1 Main analysis methods

Event rates will be estimated using Kaplan-Meier methods and will be presented using Kaplan-Meier survival curves. Analyses will utilise all data collected, up to a maximum of 5 years post randomisation. Kaplan-Meier estimates of 6-month and 1-year event rates will be reported in each treatment group alongside 95% CI's.

The primary analysis will be conducted in the ITT population. The difference in survival between treatment groups will be compared using a non-parametric stratified log-rank test (stratified by Rockwood frailty status at randomisation*). An adjusted Cox proportional hazards regression model (adjusted by Rockwood frailty status at randomisation*) will be used to estimate treatment effect. A hazard ratio (HR) <1 will indicate a reduction in the event rate in the invasive treatment group and will be presented alongside a 95% CI.

A further multivariable model will also be used, as a sensitivity analysis, to also adjust for sex, age at trial entry, MoCA status at baseline and recruiting centre (which will be added to the model as a shared frailty term).

**in the event of an error at randomisation, the correct frailty strata will be used, rather than that entered into the randomisation system.*

5.1.2 Non-proportional hazards

The proportional hazards assumption of the Cox model will be investigated using Schoenfeld residuals (graphically and using the Grambsch-Therneau test – implemented using the estat phtest command in Stata) and by testing the interaction of treatment with log(survival time), i.e. including treatment as a time dependent covariate. In the event that the statistical assumptions of the Cox model are not found to hold then a flexible parametric survival model, adjusted for Rockwood frailty status, will be used to estimate the time-dependent treatment effect (HR) and calculate the restricted mean survival time (RMST). Where there is strong evidence of non-proportional hazards the HR cannot be reliably interpreted and the RMST will take precedence as the primary estimate of the treatment effect. The choice of degrees of freedom, or number of knots, for the restricted cubic spline function and time-dependent effects in the flexible parametric survival model will be guided by model selection criteria, i.e. Akaike's Information Criteria (AIC) and Bayesian Information Criteria (BIC). The fitted survival curves from the model will be overlaid on the Kaplan-Meier curves to graphically assess goodness of fit. The time-dependent HR will be plotted against time with 95% CI's. The RMST is measured as the area under the survival curve up to a specific time, t*, and quantifies the average survival (in this case time free from recurrent non-fatal MI or cardiovascular death) from randomisation up to a specific time, t*. The value for t* will be guided by the last observed event time, as suggested by Royston and Parmar[12], up to a maximum of 5 years. The RMST in each treatment group, and the absolute difference between treatment groups, will be presented with 95% CI's. The RMST will also be plotted over time for a range of alternative values for t*.

The RMST will be calculated and reported regardless of whether the proportional hazards assumption is violated as it can provide a useful summary measure; particularly as a measure of absolute treatment effect (in contrast to the relative treatment effect provided by Hazard Ratios).

The RMST will also be estimated using a flexible parametric survival model, as described above, but without including a time-dependent treatment effect. This estimate will be compared with that

obtained when time-dependent treatment effects are incorporated as another means of assessing the proportional hazards assumption.

5.1.3 Competing risks

Given that deaths from other non-CV causes would prevent the occurrence of the primary outcome events, a competing risk analysis with the competing event being non-CV death will be performed as a supplementary analysis. Cumulative incidence of non-fatal MI or CV-death will be estimated, taking into account competing risks, using the Aalen-Johansen method and summarised by cumulative incidence curves. Estimates of 6-month and 1-year cumulative incidence will be reported in each treatment group (with 95% CI's). Estimates of the treatment effect would be made using Fine and Gray's proportional regression model, adjusted for Rockwood frailty status, and presented as the sub-distribution hazard ratio (sHR), with a 95% CI. A potential limitation of the Fine and Gray approach is the sum of the cause-specific cumulative incidence functions can sum to more than one for some covariate patterns[13]. We will investigate the impact of this potential limitation by calculating total failure probabilities for all subjects at 1, 3 and 5 year follow-up.

As above, if the proportional hazards assumption is not found to hold, flexible parametric models would be used to estimate the time-dependent sHR and the difference in RMST between treatment groups[14]. Cause-specific cumulative incidence curves will also be provided.

An additional supplementary analysis will incorporate non-CV deaths into the composite outcome measure. Time to all-cause death or non-fatal MI will be measured as the time from randomisation to death from any cause or non-fatal MI, whichever is earliest. Patients alive and free from recurrent non-fatal MI at the time of analysis will be censored at the date they were last seen or assessed. A Cox proportional hazards model, adjusted for Rockwood frailty status, will be used to estimate the treatment effect and a stratified log-rank test will be used to compare the difference in survival between treatment groups. As described in section 5.1.2, the proportional hazards assumption will be investigated, and a flexible parametric survival model used to estimate the time-dependent HR and RMST if the proportional hazards assumption is violated.

5.2 Analysis of secondary outcomes

<u>All-cause death, CV death and non-CV death</u>

Event rates will be estimated using Kaplan-Meier methods, estimates of the treatment effect (HR) will be made using Cox regression models and reported with 95% CI's, as described above. The proportional hazards assumption will be assessed, and alternatives explored as previously described.

Competing risks analyses will be performed for the analysis of CV death, with non-CV death as the competing event, as per section 5.1.3. Cause-specific cumulative incidence curves will be provided for CV and non-CV death. In the case of non-proportional hazards alternative approaches will be explored, as previously described.

Example Table: Primary and secondary time to event outcomes

Endpoint	Conservative strategy	Invasive strategy	Hazard Ratio‡ (95% CI)	p-value*
	no. events / no. patients (%)			
CV death or non-fatal MI				
All-cause death or non-fatal MI				
All-cause death				
CV death				
Non-CV death				
Non-fatal MI				
Coronary angiography†				
Coronary revascularisation†				
Bleeding (BARC ≥2)				

†Not including trial angiography and revascularisation procedures
‡Adjusted for frailty
**p-values will only be reported for the primary outcome*

Example Figure (simulated data): Kaplan-Meier estimates of the incidence of CV death or non-fatal MI

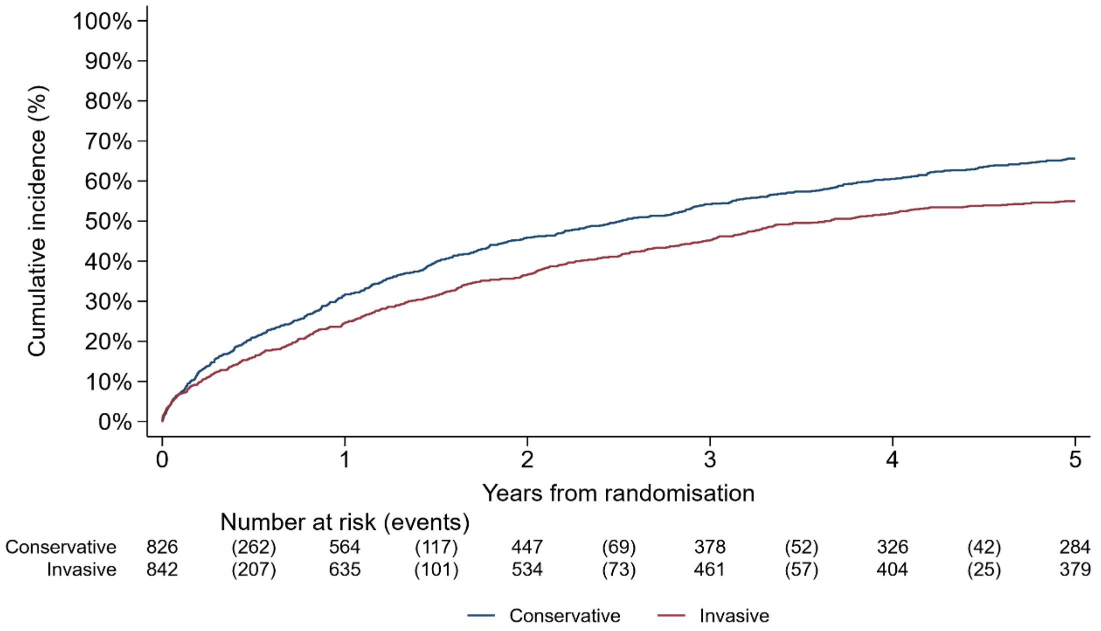

Similar Tables and Figures will be provided for all other time to event endpoints. Tables and Figures will also be provided to show cumulative incidence rates and sub-hazard ratios estimated via competing risks methodology.

<u>Recurrent MI, coronary angiography, coronary revascularisation, stroke, TIA, hospitalisation for heart failure</u>

For each outcome measure, the number of participants reporting the event, the number of events per participant, and the total number of events (including multiple events per participant) will be tabulated by randomised group. The crude rate (total number of events or total follow up time) will also be reported alongside 95% CIs.

Time to non-fatal MI, time to first (non-trial) coronary angiography and time to first (non-trial) revascularisation will be estimated using Kaplan-Meier methods. Estimates of the treatment effect (HR) will be made using Cox regression models, as described above and reported with 95% CI's. The proportional hazards assumption will be assessed, and alternatives explored as previously described. Competing risks analyses will also be performed with death as the competing event, as per section 5.1.3.

Example Table: Occurrence of secondary outcome events

	Invasive Strategy (N=)	Conservative Strategy (N=)
**Total follow up**		
Years		
**Recurrent MI**		
Total [#pts (#events)]		
Non-fatal		
Fatal		
Total		
No. per participant		
1		
2		
≥3		
Type [#pts (#events)]		
Type 1		
Type 2		
Type 3		
Type 4-5		
Rate/100 person years (95% CI)		
**Coronary angiography†**		
Total [#pts (#events)]		
No. per participant		
1		
2		
≥3		
Rate/100 person years (95% CI)		
**Coronary revascularisation†**		
Total [#pts (#events)]		
No. per participant		
1		
2		
≥3		
Rate/100 person years (95% CI)		
**Stroke**		
Total [#pts (#events)]		
No. per participant		
1		
2		
≥3		
Rate/100 person years (95% CI)		
**TIA**		
Total [#pts (#events)]		
No. per participant		
1		
2		
≥3		

	Invasive Strategy (N=)	Conservative Strategy (N=)
Rate/100 person years (95% CI)		
**Hospitalisation for heart failure**		
Total [#pts (#events)]		
No. per participant		
1		
2		
≥3		
Rate/100 person years (95% CI)		
**Bleeding (BARC ≥2)**		
Total [#pts (#events)]		
No. per participant		
1		
2		
≥3		
Rate/100 person years (95% CI)		
BARC Type [#pts (#events)]		
Type 2		
Type 3a		
Type 3b		
Type 3c		
Type 4		
Type 5a		
Type 5b		

†Not including trial angiography and revascularisation procedures

Bleeding

The number of participants reporting a bleeding event, the number of events per participant, and the total number of events (including multiple events per participant) will be tabulated by randomised group. The crude rate (total number of events or total follow up time) will also be reported alongside 95% CIs. Data will also be summarised descriptively by BARC type/grade. A Kaplan-Meier plot will be used to show time to first bleed (BARC ≥ 2) by randomised group.

Frailty scores

The distribution of scores over time will be summarised descriptively for each scoring instrument (Fried and Rockwood). Change in score from baseline and change in status (i.e. non-frail to frail) may also be summarised descriptively. Data will also be presented graphically.

Length of time spent at home

The main place of residence will be tabulated in each treatment group at each visit.

A Kaplan-Meier plot will be used to show the time to moving to a residential or nursing home by randomised group.

5.3 Additional and exploratory analyses

5.3.1 Sub-group analyses

We will explore whether the treatment effect for the primary outcome measure is consistent across the following sub-groups:

- Frail versus not frail patients (Rockwood score ≥5 vs. <5)
- Cognitively impaired versus not impaired (MoCA score <26 vs. ≥26)
- Co-morbid (Charlson age comorbidity index >5) versus non co-morbid patients (Charlson age comorbidity index ≤5)
- Age ≥80 vs <80 years
- Male versus female
- Diabetes versus no diabetes;
- Hypertension vs. no hypertension
- Grace >140 vs ≤140
- Renal disease vs. no renal disease
- Prior MI vs. no prior MI
- Never smoked vs smoked

Hazard Ratios of the treatment effect will be calculated within identified subgroups and displayed using a Forest plot with associated 95% confidence intervals. Interactions between the levels of each sub-group and the treatment effect will evaluated with Wald tests used to assess the significance of the interaction.

As the trial has not been powered to detect subgroup effects, and no adjustment for multiplicity is planned, results from these analyses should be considered exploratory and not used to infer definitive treatment effects.

5.3.2 Additional and exploratory analyses

- At a joint meeting of the DMC/TSC on 4th May 2016, prior to the trial opening to recruitment, the committee noted that rates of type 4 and 5 MI may be different early in the follow-up period and consideration should be given on whether this should be accounted for in the analysis as it might mask the impact of more clinically significant MIs that occur. One approach to account for this is to perform a landmark analysis of the primary outcome as per the FAME 2 trial analysis. For this study a landmark (cut-off) point of 30 days from randomisation would be used to allow time for any CABG procedures to be performed. HRs pre and post the landmark time would be presented. Alternative methods may be explored.
- Each component of the composite primary endpoint will be explored using the win-ratio method[15]. The main analysis of the primary outcome only takes the first event of the composite into account. However, this is often an event of lesser clinical importance, for example when a patient has a recurrent MI, whether they subsequently die is ignored. This method allows for clinical priorities, i.e. that CV deaths are considered more important than a recurrent MI, by first forming patients in the two treatment groups into matched pairs and then for each pair the invasive treatment is labelled a 'winner' or 'loser' depending on who had a CV death first. If that is unknown, then it will depend on who had a recurrent MI first. Otherwise, they will be considered ties. The 'win-ratio' is then the total number of winners divided by the total number of losers. Patients will be ranked and matched based on their risk profile (based on a risk score calculated from the coefficients of the adjusted Cox model for the primary outcome – excluding the treatment coefficient) and time stratified based on yearly intervals of the recruitment duration. The win-ratio will be presented with a 95% CI. This analysis will be strictly exploratory in nature.
- Further analyses may explore treatment effects in following additional subgroups, however it is not anticipated this will form part of the main trial report;
  - Fried frail vs prefrail vs robust
  - Frail and cognitively impaired patients versus non-frail, non-cognitively impaired patients
  - Diabetes and/or renal disease vs no diabetes and no renal disease
  - Impaired LV function vs. normal LV function (only in those with echocardiogram)
  - Severe (>50% stenosis in any epicardial vessel) vs. non-severe (<50%) coronary disease – based on angiographic analysis
  - Obstructive vs non-obstructive disease – based on angiographic analysis. This may also be explored by frailty and co-morbidity status

5.4 Missing data

Levels of missing data will be summarised. Withdrawals and losses to follow-up will be presented as described in section 4.4. Key baseline characteristics will be presented for the sub-population of participants who withdraw from the trial without allowing use of routinely collected data.

Most primary and secondary outcome measures are time to event endpoints. All participants will be included in the analysis of time-to-event outcomes, with participants who withdraw from the trial without allowing use of routinely collected data (and without prior non-fatal MI) censored at their last known follow-up time. We will primarily assume unobserved event times are censored at random, conditional on covariates included in the analysis model. However, for the primary outcome measure, if the proportion of participants (out of total randomised) censored due to withdrawal without use of routinely collected data is more than 5%, either overall or in either treatment group, we will perform sensitivity analyses using multiple imputation. We will follow the approach described by Jackson et al[16] which will be implemented using the R package *InformativeCensoring*. Briefly, sensitivity analyses will be performed by assuming that, at the point of censoring, the hazard function is multiplied by a constant, e^{γ}. Where $\gamma > 0$ there is an increased risk of failure after censoring, where $\gamma < 0$ there is a reduced risk of failure after censoring, and $\gamma = 0$ corresponds to the independent censoring assumption. By varying the value for γ we can assess the assess the robustness of the censored at random assumption and find a 'tipping point' such that the conclusions of the trial would be altered.

The variables included in the imputation model will be the same as those included in the primary analysis model. We initially plan to impute 50 datasets, however this number may be increased if 50 imputations are not felt to provide adequate precision. Each imputed dataset will be analysed using the primary analysis model and Rubin's rules used to combine treatment effect estimates.

Event times will only be imputed for participants who withdraw from follow-up without allowing continued use of routinely collected data (and have not experienced a prior non-fatal MI). Event times will be capped at each participants maximum possible follow-up time, had they not withdrawn.

We initially plan to allow γ to vary between -3 and +3 in increments of 0.5, and find a 'tipping point' value for γ such that the conclusions of the trial would be altered. Extreme cases may also be considered, e.g. by assuming all censored participants in the conservative management arm would have an event immediately after censoring and all censored participants in the invasive arm would remain event-free until the end of the trial, and vice-versa. Sensitivity analyses using multiple imputation will only be considered for the analysis of the primary outcome measure.

6. SAFETY

6.1 Procedure-related complications

Procedural complications related to the study angiography or PCI will be tabulated. The number and proportion of patients experiencing a complication related to the study procedure will be reported descriptively alongside a 95% binomial confidence interval.

Example Table: Frequency of procedural related complications

	Randomised to invasive management and undergoing angiography and/or PCI (N=)	
	N (%)	95% CI
Perforation		
Coronary Dissection		
Myocardial Infarction (type 4a)		
TIA		
Stroke		
Need for renal replacement therapy		
Death		
>25% increase in serum creatinine concentration		
Aortic Dissection		
Cardiac tamponade		
Emergency repeat angiography or PCI		
Procedure related pulmonary oedema		
Vascular complications needing intervention		
Bleeding (BARC criteria)		
Type 2		
Type 3a		
Type 3b		
Type 3c		
Type 4		
Type 5a		
Type 5b		
Other complication*		
Any complication		

**Other complications will be listed*

6.2 Serious adverse events and reactions

For this study only complications meeting the following criteria will be subject to expedited reporting:

- Those which are serious and *directly related* to the randomised coronary angiography or angioplasty procedure and occur within 7 days
- Those which are serious, *unexpected* and *directly related* to the randomised coronary angiography or angioplasty procedure occurring at any time-point

Expedited safety reporting is not required for patients randomised to receive the conservative treatment strategy.

For each event undergoing expedited reporting the following will be collected, as determined by the Principal Investigator at site or the Chief Investigator if it has not been possible to obtain local medical assessment:

- Severity (mild / moderate /severe)
- Causality (possibly / probably / definitely related)
- Expectedness (expected / unexpected)

The Chief Investigator will also provide an assessment of expectedness for all events.

Comprehensive detail of each event will be reported as a line listing.

The number of *events* and the number of *patients* reporting an event will be reported by severity.

Example Table: Line listing of all reported SAEs

ID	SAE no.	Procedure date	Onset date	Description	Severity	Causality	Outcome	Outcome date

7. STATISTICAL SOFTWARE

Data will be output directly from MACRO into a STATA format by the NCTU at time-points agreed by the TMG. Statistical analyses will be carried out by the Trial Statistician at the Biostatistics Research Group using Stata version 16 or later. Some analyses may be performed using R software. All programs and output will be stored in the School Statistics folder on the IHS server.

Appendix

The analyses of the primary outcome measure are described here using the estimand framework[17].

1. **Primary outcome**

1.1. Main analysis

Our primary objective is to estimate the relative effect of treatment on the rate of non-fatal MI or cardiovascular death. The main estimand will use a hypothetical strategy to account for the intercurrent event of death due to non-cardiovascular causes. Using this strategy, participants who die of non-cardiovascular causes (without a prior non-fatal MI) are censored in the analysis at their date of death. This assumes these participants are still at risk of the primary outcome event(s). The time to non-fatal MI or cardiovascular death is assumed to follow a similar distribution to that of other participants with the same covariates who experienced the event at a later time. If non-cardiovascular deaths give rise to independent censoring an unbiased estimate of the treatment effect can be obtained.

Other intercurrent events are handled using a treatment policy strategy, in keeping with the intention-to-treat principle.

The attributes of the main estimand for the primary endpoint are described in the table below:

**Primary Estimand**	
Estimand attribute	Description
Population	Adults aged 75 and over with Type I NSTEMI
Treatment	Invasive strategy (coronary angiography, with revascularisation where indicated, plus optimal medical treatment) or conservative strategy (optimal medical treatment alone)
Outcome variable	Time from randomisation to non-fatal MI or cardiovascular death, whichever is earliest.<br>Events will be as adjudicated by the CEC. Where a cause of death cannot be determined a cardiovascular cause will be assumed. MIs where insufficient source data is available to allow for adjudication will be assumed to be an MI as per the local site assessment.
Population-level summary measure	Hazard Ratio (Invasive vs Conservative strategy) estimated from a Cox regression model
Strategies used to handle intercurrent events	• Not undergoing allocated angiography – treatment policy<br>• Undergoing non-allocated angiography – treatment policy<br>• Non-cardiovascular death – hypothetical

1.2. Sensitivity analyses

Two sensitivity analysis are planned which target the same estimand as described above;

1. The analysis will be repeated using a multivariate Cox regression model to explore the impact of important baseline covariates on the estimated treatment effect.
2. This analysis will be repeated assuming deaths where a cause could not be determined by the CEC are non-cardiovascular.

1.3. Supplementary analyses

1.3.1. Competing risks

A supplementary analyses of the primary endpoint will consider the intercurrent event of non-cardiovascular death as a competing event. This can be considered as a "while alive" strategy using the estimand framework.

A Fine and Gray regression model will be used to estimate the relative effect of treatment on the rate of the primary outcome via the sub-distribution hazard ratio. Here we are considering the rate of the primary outcome in those who are event-free or who have experienced a competing event (non-cardiovascular death, without prior non-fatal MI). Participants who experience the competing event are kept in the risk set so they can be counted as having no risk of the primary event.

The attributes of this supplementary estimand are described in the table below:

**Supplementary Estimand 1**	
Estimand attribute	Description
Population	Adults aged 75 and over with Type I NSTEMI
Treatment	Invasive strategy (coronary angiography, with revascularisation where indicated, plus optimal medical treatment) or conservative strategy (optimal medical treatment alone)
Outcome variable	Time from randomisation to non-fatal MI or cardiovascular death, whichever is earliest.<br>Events will be as adjudicated by the CEC. Where a cause of death cannot be determined a cardiovascular cause will be assumed. MIs where insufficient source data is available to allow for adjudication will be assumed to MIs as per the local site assessment.
Population-level summary measure	Sub-distribution Hazard Ratio (Invasive vs Conservative strategy) estimated from a Fine and Gray regression model
Strategies used to handle intercurrent events	• Not undergoing allocated angiography – treatment policy<br>• Undergoing non-allocated angiography – treatment policy<br>• Non-cardiovascular death – while alive

An additional supplementary analysis will incorporate non-cardiovascular deaths into the outcome measure. This will modify the outcome variable to be time to non-fatal MI or death due to any cause and can be considered a "composite" strategy using the estimand framework.

The attributes of this supplementary estimand are described in the table below:

**Supplementary Estimand 2**	
Estimand attribute	Description
Population	Adults aged 75 and over with Type I NSTEMI
Treatment	Invasive strategy (coronary angiography with revascularisation where indicated) plus optimal medical treatment or conservative strategy (optimal medical treatment alone)
Outcome variable	Time from randomisation to non-fatal MI or death from any cause, whichever is earliest
Population-level summary measure	Hazard Ratio (Invasive vs Conservative strategy)
Strategies used to handle intercurrent events	• Not undergoing allocated angiography – treatment policy<br>• Undergoing non-allocated angiography – treatment policy

1.3.2. Non-proportional hazards

If, for any of the analyses specified above, the proportional hazards assumption of the Cox regression model (or Fine and Gray regression model) is found to be violated our focus will be on estimating the absolute difference in the restricted mean survival time (RMST) between randomised treatment groups.

Alternative estimands to be considered, corresponding to the Primary Estimand and Supplementary Estimands 1 and 2 respectively, are described below:

**Main Estimand in event of non-proportional hazards**	
Estimand attribute	Description
Population	Adults aged 75 and over with Type I NSTEMI
Treatment	Invasive strategy (coronary angiography, with revascularisation where indicated, plus optimal medical treatment) or conservative strategy (optimal medical treatment alone)
Outcome variable	Time from randomisation to non-fatal MI or cardiovascular death, whichever is earliest.<br>Events will be as adjudicated by the CEC. Where a cause of death cannot be determined a cardiovascular cause will be assumed. MIs where insufficient source data is available to allow for adjudication will be assumed to be an MI as per the local site assessment.
Population-level summary measure	Absolute difference (Invasive – Control) in the RMST between randomised treatment groups. The RMST is measured as the area under the survival curve up to a specific time, t* and can be interpreted as the average time free from recurrent MI or cardiovascular death from randomisation up to a specific time, t*.
Strategies used to handle intercurrent events	• Not undergoing allocated angiography – treatment policy<br>• Undergoing non-allocated angiography – treatment policy<br>• Non-cardiovascular death – hypothetical

**Supplementary Estimand 1 in event of non-proportional hazards**	
Estimand attribute	Description
Population	Adults aged 75 and over with Type I NSTEMI
Treatment	Invasive strategy (coronary angiography, with revascularisation where indicated, plus optimal medical treatment) or conservative strategy (optimal medical treatment alone)
Outcome variable	Time from randomisation to non-fatal MI or cardiovascular death, whichever is earliest.<br>Events will be as adjudicated by the CEC. Where a cause of death cannot be determined a cardiovascular cause will be assumed. MI’s where insufficient source data is available to allow for adjudication will be assumed to be an MI as per the local site assessment.
Population-level summary measure	Absolute difference (Invasive – Control) in the RMST between randomised treatment groups. In this case the RMST will be measured based on cause-specific cumulative incidence curves.
Strategies used to handle intercurrent events	• Not undergoing allocated angiography – treatment policy<br>• Undergoing non-allocated angiography – treatment policy<br>• Non-cardiovascular death – while alive

**Supplementary Estimand 2 in event of non-proportional hazards**	
Estimand attribute	Description
Population	Adults aged 75 and over with Type I NSTEMI
Treatment	Invasive strategy (coronary angiography, with revascularisation where indicated, plus optimal medical treatment) or conservative strategy (optimal medical treatment alone)
Outcome variable	Time from randomisation to non-fatal MI or death from any cause, whichever is earliest.<br>Events will be as adjudicated by the CEC. Where a cause of death cannot be determined a cardiovascular cause will be assumed. MIs where insufficient source data is available to allow for adjudication will be assumed to be an MI as per the local site assessment.
Population-level summary measure	Absolute difference (Invasive – Control) in the RMST between randomised treatment groups. The RMST is measured as the area under the survival curve up to a specific time, t* and can be interpreted as the average time free from recurrent MI or death from randomisation up to a specific time, t*.
Strategies used to handle intercurrent events	• Not undergoing allocated angiography – treatment policy<br>• Undergoing non-allocated angiography – treatment policy<br>• Non-cardiovascular death – composite

References

1. Gamble C, Krishan A, et al. Guidelines for the Content of Statistical Analysis Plans in Clinical Trials. *JAMA*. 2017;318(23):2337-2343

2. Thygesen K, Alpert JS, et al. Fourth universal definition of myocardial infarction (2018). *European Heart Journal*. 2019;40:237-269

3. Mehran R, Rao SV, et al. Standardized bleeding definitions for cardiovascular clinical trials: A consensus report from the Bleeding Academia Research Consortium. Circulation. 2011;123:2736-2747

4. Fried LP, Tangen CM, et al. Frailty in Older Adults: Evidence for a Phenotype. *Journal of Gerontology: MEDICAL SCIENCES* 2001, Vol. 56A, No. 3, M146–M156

5. Rockwood K, Song X et al. A global clinical measure of fitness and frailty in elderly people. *CMAJ*. 2005;173(5):489-495

6. Qian W, Parmar MKB, et al. Analysis of messy longitudinal data from a randomized clinical trial. *Statist*. *Med*. 2000; 19:2657-2674

7. Billingham LJ, Abrhams KR. Simultaneous analysis of quality of life and survival data. *Statistical Methods in Medical Research*. 2002; 11: 25-48

8. Ekerstad N, Swahn E, et al. Frailty is independently associated with 1-year mortality for elderly patients with non-ST-segment elevation myocardial infarction. *Eur J Prev Cardiol*. 2014;21:1216-1224

9. Machin D, Campbell MJ et al. Comparing survival curves. *Sample size tables for clinical studies*. Wiley-Blackwell; 2009:84-101.

10. Barthel F M-S, Babiker A, Royston P, Parmar MKB (2006) Evaluation of sample size and power for multi-arm survival trials allowing for non-uniform accrual, non-proportional hazards, loss to follow-up and cross-over. *Statistics in Medicine*, 25, 2521-2542.

11. Barthel, F M-S, Royston P, Babiker A (2005). A menu-driven facility for complex sample size calculation in randomized controlled trials with a survival or a binary outcome: Update. *Stata Journal* 5: 123–129.

12. Royston P, Parmar MKB. The use of restricted mean survival time to estimate the treatment effect in randomized clinical trials when the proportional hazards assumption is in doubt. *Stat Med. 2011;* **30**: 2409-2421

13. Austin PC, Steyerberg EW, Putter H. Fine-Gray subdistribution hazard models to simultaneously estimate the absolute risk of different event types: Cumulative total failure probability may exceed 1. *Stat Med. 2021;* **40**: 4200-4212

14. Mozumder SI, Rutherford MJ, Lambert PC. Estimating restricted mean survival time and expected life-years lost in the presence of competing risks within flexible parametric survival models. *BMC Medical Research Methodology.* 2021; 21:52

15. Pocock SJ, Ariti CO et al. The win ratio: a new approach to the analysis of composite endpoints in clinical trials based on clinical priorities. *European Heart Journal.* 2012; 33: 176-182.

16. Jackson D, White I, et al. Relaxing the independent censoring assumption in the Cox proportional hazards model using multiple imputation. *Statistics in Medicine*. 2014; 33(27):4681-4694

17. ICH E9 (R1) addendum on estimands and sensitivity analysis in clinical trials to the guideline on statistical principles for clinical trials. EMA/CHMP/ICH/436221/2017.

SAP Summary of changes

Version	Date	Major changes made	Justification for change
0.1	20/05/2017	First draft	NA
0.2	15/06/2017	Safety reporting procedures clarified	Discussed and agreed at TMG meeting on 24/05/17. Protocol amendment to be made to clarify SAE reporting procedures.
0.3	03/12/2018	General formatting updates and changes to standard text in line with SAP template [dated 24/01/18]	To incorporate changes in made to the current SAP template
		Updates to stratification factors used for randomisation	In line with protocol amendment (v2.0)
		Analysis methods for safety data updated in line with protocol amendment to version 2.0	Due to protocol amendment (v 2.0) which clarified safety reporting requirements.
		Changes to Example Tables to reflect database updates and DMC suggestions	In line with database updates and DMC suggestions at 30th June 2017 meeting and 4th September 2018 meeting.
0.4	26/09/2019	Removed 'within one year' from the definition of the primary objective	In line with protocol version 3.0 [draft].
		Sample size calculation updated to incorporate events accruing over the whole follow-up period rather than just within 1 year from randomisation	For detailed justification see section 2.5.
		Addition of coronary angiography as an outcome measure and removal of recurrent hospitalisation for MI	Coronary angiography was added to the study database after the start of the trial. Recurrent hospitalisation for MI, specifically, was not collected in the study database. Recurrent MIs are collected.
		Clarified that the primary analysis method will be a stratified log-rank test and Cox regression model adjusted for baseline stratification factors	Primary analysis to account for frailty status (baseline stratification factor) as this should improve the power of the estimated treatment effect.
		Removal of quality of life analyses assessed by EQ-5D-5L	Due to duplication with health economic analyses EQ-5D-5L data will only be analysed by the health economic team. Analysis methods are documented in the health economics analysis plan.
		Changes to study follow-up arrangements as per protocol version 3.0 [draft]	In line with protocol version 3.0 [draft].
		Addition of an exploratory analysis of the composite primary endpoint using the win-ratio method	To explore each component of the composite endpoint taking into account clinical priorities.
0.5	14/04/2021	Addition of statement to clarify how primary outcome events which cannot be adjudicated by the CEC will be handled in the analysis	Discussed and agreed with the TSC
		Definition of MI changed from 3rd to 4th Universal definition	In line with protocol version 3.0
1.0	14/04/2021	Version 0.5 made final version 1.0	NA

Version	Date	Major changes made	Justification for change
2.0	24/11/2023	Amended relevant analyses to be adjusted for frailty status according to Rockwood score rather than Fried score	Fried score was stated in error. Rockwood score is used for stratification in the randomisation system and will be used in analyses.
		Added a supplementary analysis of the primary outcome which will include non-cardiovascular deaths as part of the composite outcome (i.e. non-fatal MI or death from any cause).	The TSC suggested reporting a composite all-cause mortality and MI outcome as a subsidiary primary outcome measure at their meeting on 18//10/2021. Following a review of outcome measures used in other cardiovascular trials, and giving consideration to the Estimand framework, the TMG agreed to add an analysis of all-cause mortality and MI as a supplementary analysis of the primary outcome measure (the term 'supplementary' was chosen to use the same terminology as in the Estimand framework).
		Definition of non-fatal MI added	To provide clarity as this was not previously defined
		Clarified coronary angiography and coronary revascularisation outcome measures will exclude those performed as part of the trial	Clinical interest for this outcome measure is in the need for repeat procedures.
		Added more detail on analyses to be performed if the proportional hazards assumption of the Cox regression model is not found to hold	To further pre-specify technical details of how such analyses are to be performed
		Added reporting of time to event analyses for time to first non-fatal MI	This is to be reported as it is a component of the primary outcome measure
		Analysis methods added for the Length of time spent at home outcome measure	Analysis methods added as these were previously not specified
		Removed SAUC approach for the analysis of frailty scores over time	Due to the higher than expected missing data rates due to COVID-19 the SAUC approach was no longer felt to be a robust method of analysis due to the high level of imputation which would be required.
		Additional subgroup analyses added	Additional subgroups of clinical interest pre-specified
		Sensitivity analyses for the primary outcome using multiple imputation added to explore departures from a censoring at random assumption for participants withdrawing from follow-up.	Added in response to suggestions made by the Data Monitoring Committee at their meeting on 10th May 2023
		Added a description of the estimands of interest for the primary outcome measure as an Appendix	Following release of the ICH E9 Addendum on estimands and sensitivity analyses
