# Supplementary material for: Invasive vs Conservative Strategy for Frail Older Patients With Myocardial Infarction: A Secondary Analysis of the SENIOR-RITA Randomized Clinical Trial
Source: JAMA Netw Open. 2026 Apr 21;9(4):e267316. doi: 10.1001/jamanetworkopen.2026.7316 (PMC13100840; doi:10.1001/jamanetworkopen.2026.7316)
Supplement: Supplement 2. — eMethods. eTable 1. Baseline Demographics, Clinical Characteristics and Medical Therapy of Frail, Pre-Frail and Robust Patients as Per Randomized Strategy eTable 2. Restricted Mean Event-Free Time to Account for Non-Proportional Hazards in Frail and Pre-Frail Patients eTable 3. Primary Outcome Accounting for Competing Risk In Frail, Pre-Frail and Robust Patients—Fine and Gray Regression Model eFigure 1. Subgroup-Study Flow Chart eFigure 2. Cumulative Incidence of Cardiovascular Death in Frail, Pre-Frail and Robust Patients eFigure 3. Cumulative Incidence of Non-Fatal Myocardial Infarction in Frail, Pre-Frail and Robust Patients eFigure 4. Treatment Effect for Primary Outcome and components of Primary Outcome With Interaction Between Frailty and Strategy Arm in Robust, Pre-Frail and Frail Patients [file jamanetwopen-e267316-s002.pdf]

Supplementary Online Content

Rubino F, Mossop H, Ripley DP, et al. Invasive vs conservative strategy for frail older patients with myocardial infarction: a secondary analysis of the SENIOR-RITA randomized clinical trial. *JAMA Netw Open.* 2026;9(4):e267316. doi:10.1001/jamanetworkopen.2026.7316

eMethods.
eTable 1. Baseline Demographics, Clinical Characteristics and Medical Therapy of Frail, Pre-Frail and Robust Patients as Per Randomized Strategy
eTable 2. Restricted Mean Event-Free Time to Account for Non-Proportional Hazards in Frail and Pre-Frail Patients
eTable 3. Primary Outcome Accounting for Competing Risk In Frail, Pre-Frail and Robust Patients—Fine and Gray Regression Model
eFigure 1. Subgroup-Study Flow Chart
eFigure 2. Cumulative Incidence of Cardiovascular Death in Frail, Pre-Frail and Robust Patients
eFigure 3. Cumulative Incidence of Non-Fatal Myocardial Infarction in Frail, Pre-Frail and Robust Patients
eFigure 4. Treatment Effect for Primary Outcome and components of Primary Outcome With Interaction Between Frailty and Strategy Arm in Robust, Pre-Frail and Frail Patients

This supplementary material has been provided by the authors to give readers additional information about their work.

© 2026 Rubino F et al. *JAMA Network Open.*

SUPPLEMENTARY METHODS

Inclusion and exclusion criteria SENIOR-RITA Trial.

**Inclusion criteria (all the following)**
○ Age ≥75 years old<br>○ Type 1 NSTEMI during index hospitalization
**Exclusion criteria (any of the following)**
○ Patients presenting with STEMI or unstable angina
○ Patients with cardiogenic shock
○ Patients with a known life expectancy of <1 year
○ Patients in whom neither the patient nor the consultee are able and willing to provide written informed consent
○ Previous randomization in the SENIOR RITA trial
○ Inability to undergo invasive coronary angiography, such as no vascular access site, or absolute contraindication to coronary revascularization

© 2026 Rubino F et al. *JAMA Network Open.*

Fried Frailty Criteria

Criterion Frailty Status	Criterion Frailty Status
**Shrinking**	**Frailty cut point:**<br>**Baseline:** Self-reported unintentional weight loss ≥10lb in previous year.<br>**Follow-up:** Unintentional weight loss ≥5% of previous year's body weight OR BMI <18.5kg/m^2.
**Physical endurance/energy**	**Geriatric Depression Scale:**<br>Do you feel full of energy?<br>During the last 4 weeks how often you rested in bed during day? *Response options: Every day, every week, once, not at all.*<br>**Frailty cut point:** No to 1 and every day or every week to 2.
**Low physical activity**	Frequency of mildly energetic, moderately energetic and very energetic physical activity. *Response options: ≥3 times per week, 1-2 times per week, 1-3 times per month, hardly ever/never.*<br>**Frailty cut point:** Hardly ever/never for very energetic physical activity AND for moderately energetic physical activity.
**Weakness**	Hand grip strength in Kg: GRIP-D handheld dynamometer, dominant hand, average of 3 measures.<br>**Frailty cut point:**<br>**Grip strength:** lowest 20% (by gender, body mass index)<br>Men<br>BMI ≤24 ≤29<br>BMI 24.1–26 ≤30<br>BMI 26.1–28 ≤30<br>BMI >28 ≤32<br>Women<br>BMI ≤23 ≤17<br>BMI 23.1–26 ≤17.3<br>BMI 26.1–29 ≤18<br>BMI >29 ≤21
**Slow walking speed**	Walking time in seconds (usual pace) over 15 feet.<br>**Frailty cut point:** Slowest 20%, stratified by gender and median standing height. Men Height ≤173 cm Height >173 cm Women Height ≤159 cm Height >159 cm ≥7 seconds ≥6 seconds ≥7 seconds ≥6 seconds. OR<br>Time to complete "timed up and go test" (TUG).<br>**Frailty cut point:** TUG time ≥19 seconds.
**Frail:** ≥3 criteria present. **Intermediate or Pre-Frail:**1 or 2 criteria present. **Robust:** 0 criteria present.	

© 2026 Rubino F et al. *JAMA Network Open.*

Charlson Comorbidity Index

**Each condition is assigned a score of 1, 2, 3, or 6.**	
**1**	Myocardial infarction, congestive heart failure, peripheral vascular disease, cerebrovascular disease, dementia, chronic pulmonary disease, rheumatologic disease, peptic ulcer disease, liver disease (if mild, or 3 if moderate/severe), diabetes (if controlled, or 2 if uncontrolled)
**2**	Hemiplegia or paraplegia, renal disease, malignancy (if localized, or 6 if metastatic tumor), leukemia, lymphoma
**6**	AIDS
**Age:**	
**+1**	50-59 years old
**+2**	60-69 years old
**+3**	70-79 years old
**+4**	80 years old or more

© 2026 Rubino F et al. *JAMA Network Open.*

Montreal Cognitive Assessment (MoCA)

**Domain**	**Maximum Score**
Executive/visuospatial function	5 points
Naming	3 points
Attention	6 points
Language	3 points
Abstraction	2 points
Recall	5 points
Orientation	6 points
TOTAL	30 points
**1 point** is added to the total score if a person has 12 years or less of formal education	
Scores ≥26 is classified as normal, and <26 as cognitively impaired	

© 2026 Rubino F et al. *JAMA Network Open.*

eTable 1. Baseline Demographics, Clinical Characteristics and Medical Therapy of Frail, Pre-Frail and Robust Patients as Per Randomized Strategy

	Frail patients		Pre-frail patients		Robust patients	
**Characteristics** No./total No. (%)	**Invasive strategy (N =231)**	**Conservative strategy (N =238)**	**Invasive strategy (N=335)**	**Conservative strategy (N=339)**	**Invasive strategy (N=150)**	**Conservative strategy (N=153)**
Female	115/231 (49.8)	125/238 (52.5)	145/335 (43.3)	146 /339 (43.1)	58/150 (38.7)	59/153 (38.6)
Male	116/231 (50.2)	113/238 (47.5)	190/335 (56.7)	193/339 (56.9)	100/150 (66.7)	93/153 (60.8)
Age – year- median (IQR)	83 (80-87)	83 (80-86)	82 (79-86)	82 (79-85)	80 (78-83)	80 (78-83)
Median days from admission to randomisation (IQR)	2 (1-3)	2 (1-3)	2 (1-3)*	2 (1-3)*	2 (1-2)	2 (1-3)
Median MoCA (IQR)	23.0 (20.0-26.0)	23.0 (18.0- 25.2)	25.0 (22-27)	24.0 (21-26)	26.0 (23-28)	25.5 (23-27)
Median Charlson age-adjusted co-morbidity index (IQR)	6 (5-7)	6 (5-7)	5 (4-6)	5 (4-6)	5 (4-6)	5 (4-6)
Current Smoker	9/231 (3.9)	16/234 (6.8)	15/331 (4.5)	19/337 (5.6)	7/149 (4.7)	8/152 (5.3)
Hypertension	157/231 (68.0)	158/238 (66.4)	207/335 (61.8)	224/337 (66.1)	102/150 (68.0)	96/153 (62.7)
Diabetes	75/231 (32.5)	82/238 (34.5)	111/335 (33.1)	101/337 (29.8)	35/150 (23.3)	36/153 (23.5)
Hypercholesterolemia-	61/231 (26.4)	78/238 (32.8)	119/334 (35.4)	104/ 336 (30.9)	50/150 (33.3)	46/153 (30.1)
History of renal disease	61/231 (26.4)	60/238 (25.2)	62/335 (18.5)	61/336 (18.0)	24/150 (16.0)	25/153 (16.3)
Previous myocardial infarction	91/231 (39.4)	86/238 (36.1)	106/335 (31.6)	92/336 (27.1)	38/150 (25.3)	37/153 (24.2)
Previous PCI	60/230 (26.1)	51/238 (21.4)	63/335 (18.8)	57/336 (16.8)	30/150 (20.0)	24/153 (15.7)
Previous CABG	37/230 (16.0)	27/238 (11.3)	41/335 (12.2)	35/336 (10.3)	19/150 (12.7)	13/153 (8.5)
History of peripheral vascular disease	26/230 (11.3)	21/238 (8.8)	19/335 (5.7)	25/336 (7.4)	8/150 (5.3)	9/153 (5.9)
History of TIA/Stroke	41/230 (17.7)	41/238 (17.2)	58/335 (17.3)*	36/336 (10.6)*	23/150 (15.3)	19/153 (12.4)
History of COPD	48/230 (20.8)	45/238 (18.9)	44/335 (13.1)	51/336 (15.0)	16/150 (10.7)	17/153 (11.1)
History of Congestive Heart Failure	35/230 (15.2)	40/238 (16.8)	20/335 (6.0)	20/336 (5.9)	14/150 (9.3)	5/153 (3.3)
**Discharge medical therapy**						
Aspirin	208/231 (90.0)*	192/238 (80.7)*	300/334 (89.8)	302/338 (89.3)	138/150 (92.0)	140/153 (91.5)
$P2Y_{12}$ Receptor Antagonist (Total)	209/231 (90.5)	217/238 (91.2)	298/334 (89.2)*	327/338 (96.7)*	136/150 (90.7)	144/153 (94.1)
Anticoagulant (Total)	53/231 (22.9)	67/238 (28.2)	79/334 (23.7)	77/338 (22.8)	31/150 (20.7)	32/153 (21.0)
Lipid-lowering therapy	204/231 (88.3)	207/238 (87.0)	311/334 (93.1)	307/338 (90.8)	137/150 (91.3)	143/153 (93.5)

ACE, angiotensin converting enzyme; ARB, Angiotensin receptor blocker CABG, coronary artery bypass graft; COPD, chronic obstructive pulmonary disease; IQR, interquartile range; MoCA, Montreal Cognitive Assessment; PCI, percutaneous coronary intervention; TIA, transient ischemic attack. *P value<0.05.

© 2026 Rubino F et al. *JAMA Network Open.*

eTable 2. Restricted Mean Event-Free Time to Account for Non-Proportional Hazards in Frail and Pre-Frail Patients

	**Frail patients**		
Outcome variables	Invasive Strategy* (N= 231)	Conservative Strategy* (N=238)	Mean difference† (95% CI)
Cardiovascular death and non-fatal MI	3.64 (3.39-3.89)	3.80 (3.55-4.04)	–57 days (–186 days to +72 days)
Coronary angiography	3.90 (3.82-3.97)	3.90 (3.82-3.97)	+244 days (+169 days to 318 days)
	**Pre-frail patients**		
	Invasive Strategy* (N= 335)	Conservative Strategy* (N=339	
Coronary angiography	3.93 (3.85-4.00)	3.27 (3.11-3.43)	+239 days (+174 days to 304 days)

MI, Myocardial infarction.

*Data shown are the restricted mean event-free time at 5 years post-randomization (95% confidence intervals).
†Mean difference for invasive strategy compared to conservative strategy, estimated in days.
The widths of the confidence intervals have not been adjusted for multiplicity and should not be used in place of hypothesis testing.

© 2026 Rubino F et al. *JAMA Network Open.*

eTable 3. Primary Outcome Accounting for Competing Risk In Frail, Pre-Frail and Robust Patients—Fine and Gray Regression Model

	**Frail patients**		
Outcome variables	Invasive Strategy (N= 231)	Conservative Strategy (N=238)	Sub- distribution Hazard Ratio* (95% CI)
Cardiovascular death and non-fatal MI	87 (37.7)	70 (29.4)	1.32 (0.96 -1.81)
Cardiovascular death	59 (25.5)	44 (18.5)	1.23 (0.875 -1.73)
Non-fatal MI	34 (14.7)	33 (13.9)	1.09 (0.68 -1.76)
	**Pre-frail patients**		
Cardiovascular death and non-fatal MI	72 (21.5)	86 (25.4)	0.81 (0.59-1.11)
Cardiovascular death	42 (12.5)	46 (13.6)	0.83 (0.60-1.16)
Non-fatal MI	37 (11.0)	51 (15.0)	0.71 (0.47-1.09)
	**Robust patients**		
Cardiovascular death and non-fatal MI	30 (20.0)	31(20.3)	0.94 (0.57 -1.55)
Cardiovascular death	15 (10.0)	10 (6.5)	0.72 (1.39-1.23)
Non-fatal MI	16 (10.7)	24 (15.7)	0.67 (0.36-1.26)

MI, Myocardial infarction.

*Non-cardiovascular death as competing risk.

© 2026 Rubino F et al. *JAMA Network Open.*

eFigure 1. Subgroup-Study Flow Chart

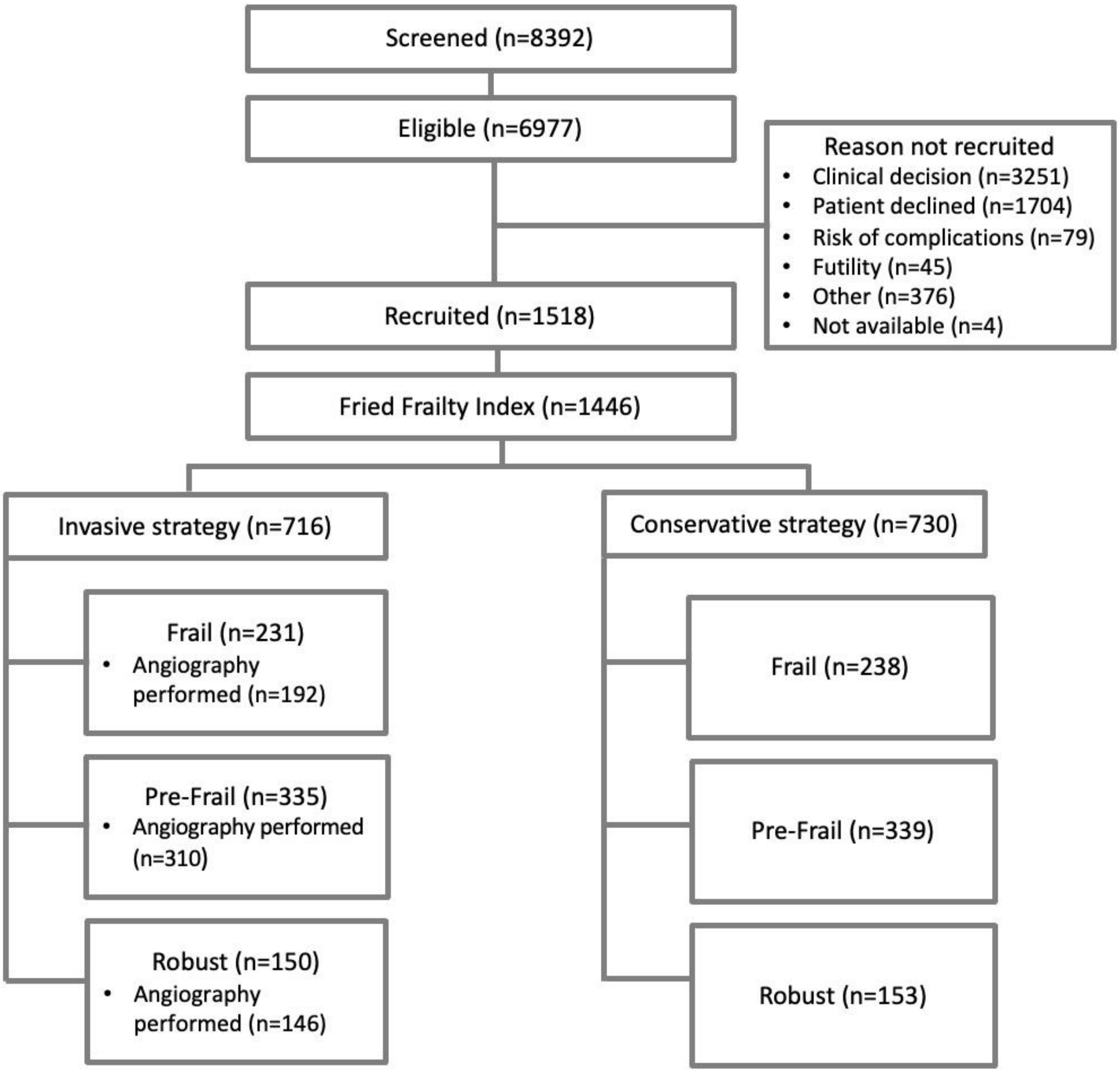

© 2026 Rubino F et al. *JAMA Network Open.*

eFigure 2. Cumulative Incidence of Cardiovascular Death in Frail, Pre-Frail and Robust Patients

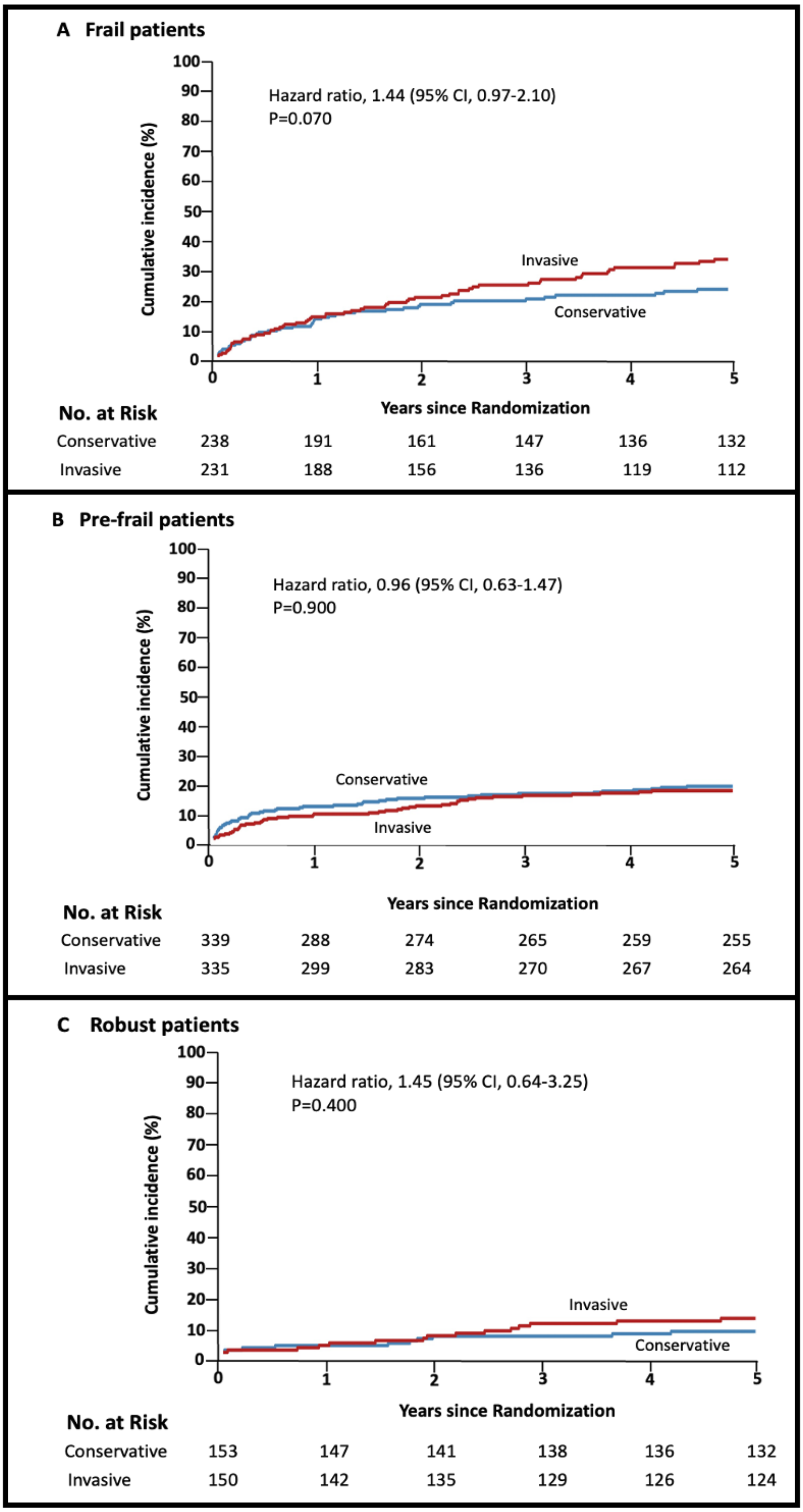

© 2026 Rubino F et al. *JAMA Network Open.*

eFigure 3. Cumulative Incidence of Non-Fatal Myocardial Infarction in Frail, Pre-Frail and Robust Patients

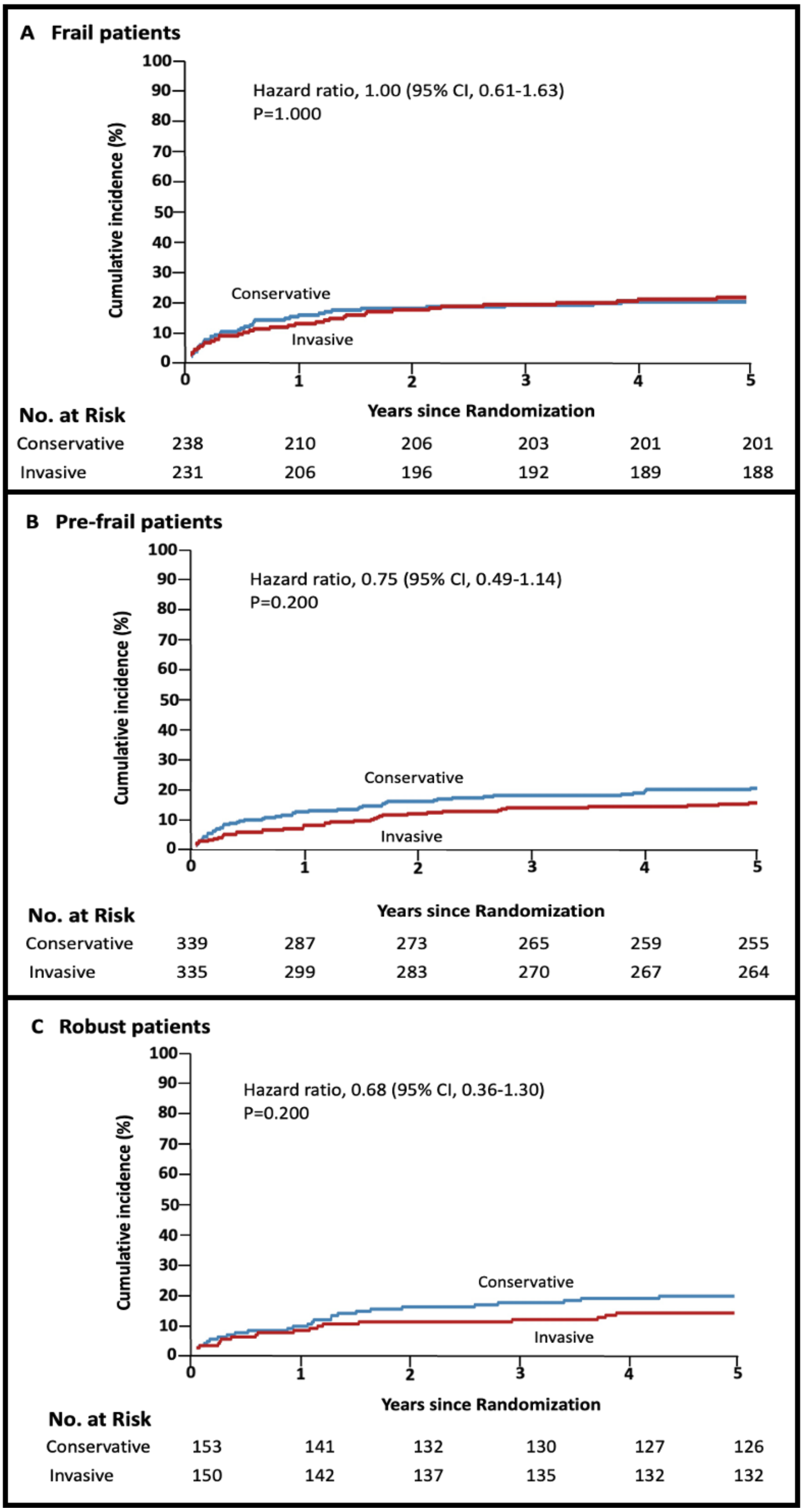

© 2026 Rubino F et al. *JAMA Network Open.*

eFigure 4. Treatment Effect for Primary Outcome and components of Primary Outcome With Interaction Between Frailty and Strategy Arm in Robust, Pre-Frail and Frail Patients

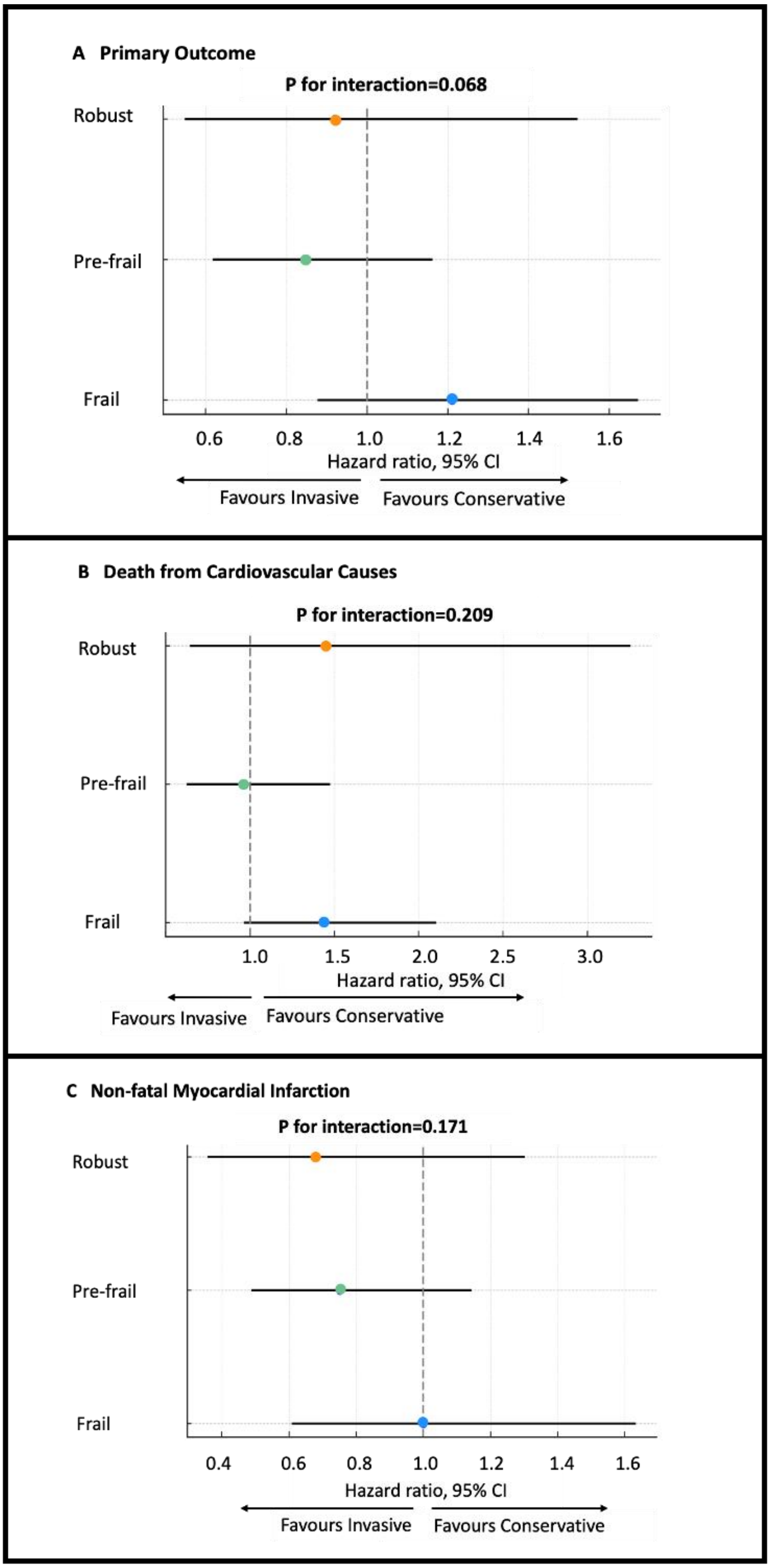

© 2026 Rubino F et al. *JAMA Network Open.*
