# Supplementary material for: Invasive vs Conservative Strategy for Frail Older Patients With Myocardial Infarction: A Secondary Analysis of the SENIOR-RITA Randomized Clinical Trial
Source: JAMA Netw Open. 2026 Apr 21;9(4):e267316. doi: 10.1001/jamanetworkopen.2026.7316 (PMC13100840; doi:10.1001/jamanetworkopen.2026.7316)
Supplement: Supplement 3. — Data Sharing Statement [file jamanetwopen-e267316-s003.pdf]

Data Sharing Statement

Rubino. Invasive vs Conservative Strategy for Frail Older Patients With Myocardial Infarction.
JAMA Netw Open. Published April 21, 2026. doi:10.1001/jamanetworkopen.2026.7316

Data
Additional Information: ISRCTN 11343602
Data available: No

Additional Information
Explanation for why data not available: Long term follow-up still ongoing
